# Supplementary figures and images for: Comparative SIR/SEIR modeling of the Antonine Plague in Rome
Source: PLoS One. 2025 Feb 13;20(2):e0313684. doi: 10.1371/journal.pone.0313684 (PMC11824979; doi:10.1371/journal.pone.0313684)

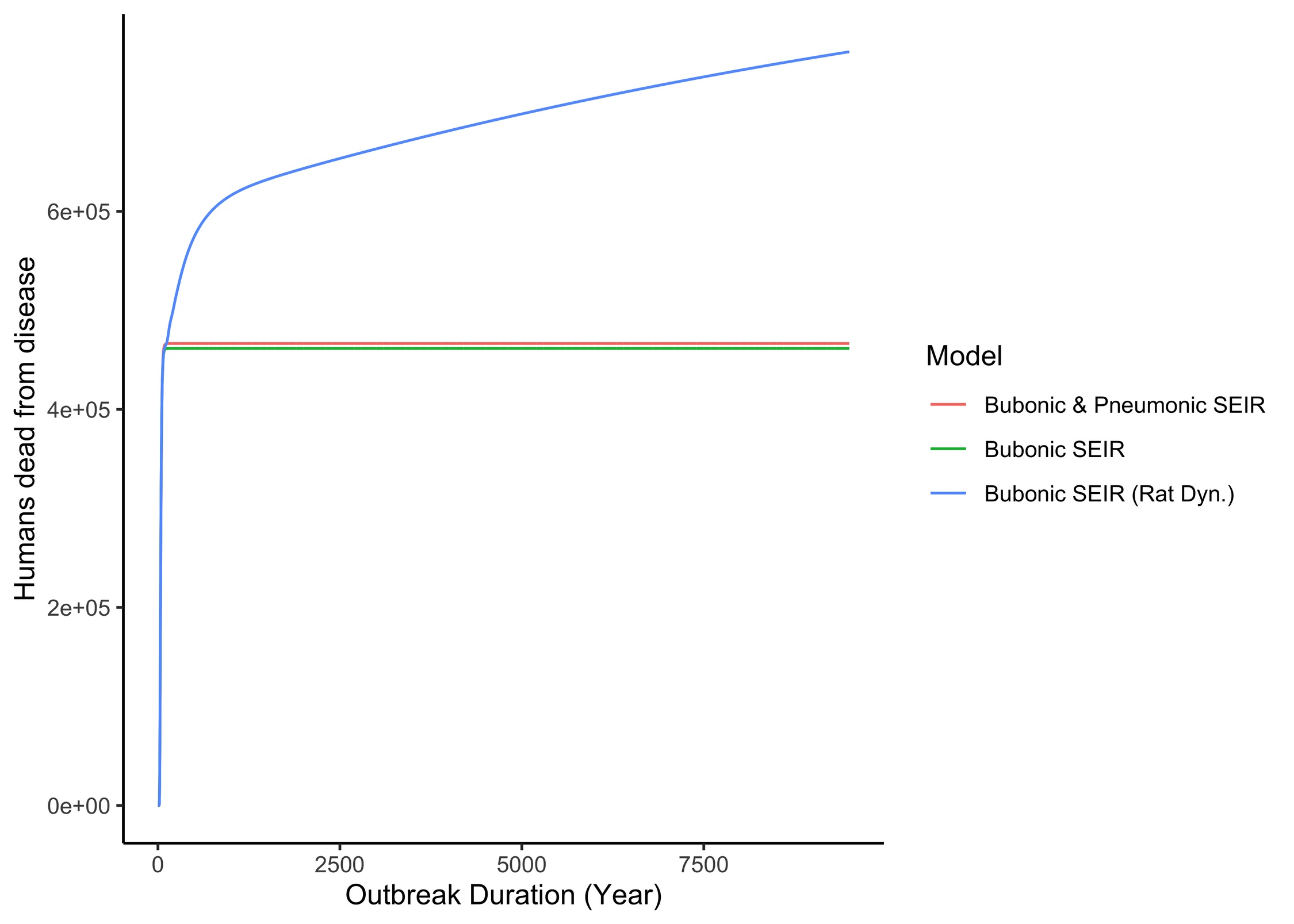

Supplement: S1 Fig — Created with rat to human ratio of 1:2 and using expected epidemiological features from Table 1. Initial conditions: number of susceptible humans, Sh(t = 0) = 923,406; number of susceptible rats, Sr(t = 0) = 461,702; and number of infected rats, Ir(t = 0) = 1. (TIF) [file pone.0313684.s001.tif]

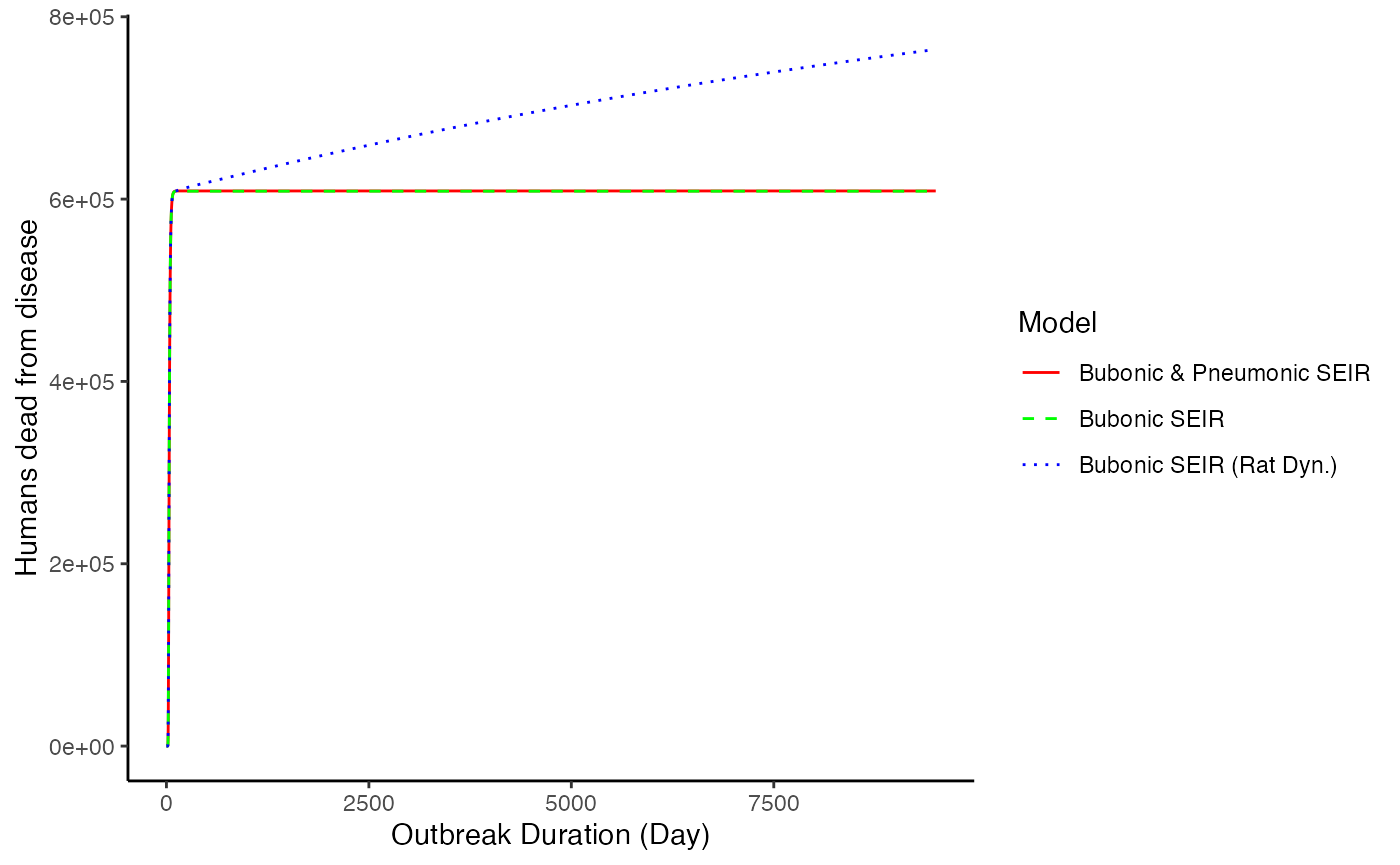

Supplement: S2 Fig — Created with a rat to human ratio of 2:1 and using expected epidemiological features from Table 1. Initial conditions: number of susceptible humans, Sh(t = 0) = 923,406; number of susceptible rats, Sr(t = 0) = 1,846,811; and number of infected rats, Ir(t = 0) = 1. (TIF) [file pone.0313684.s002.tif]

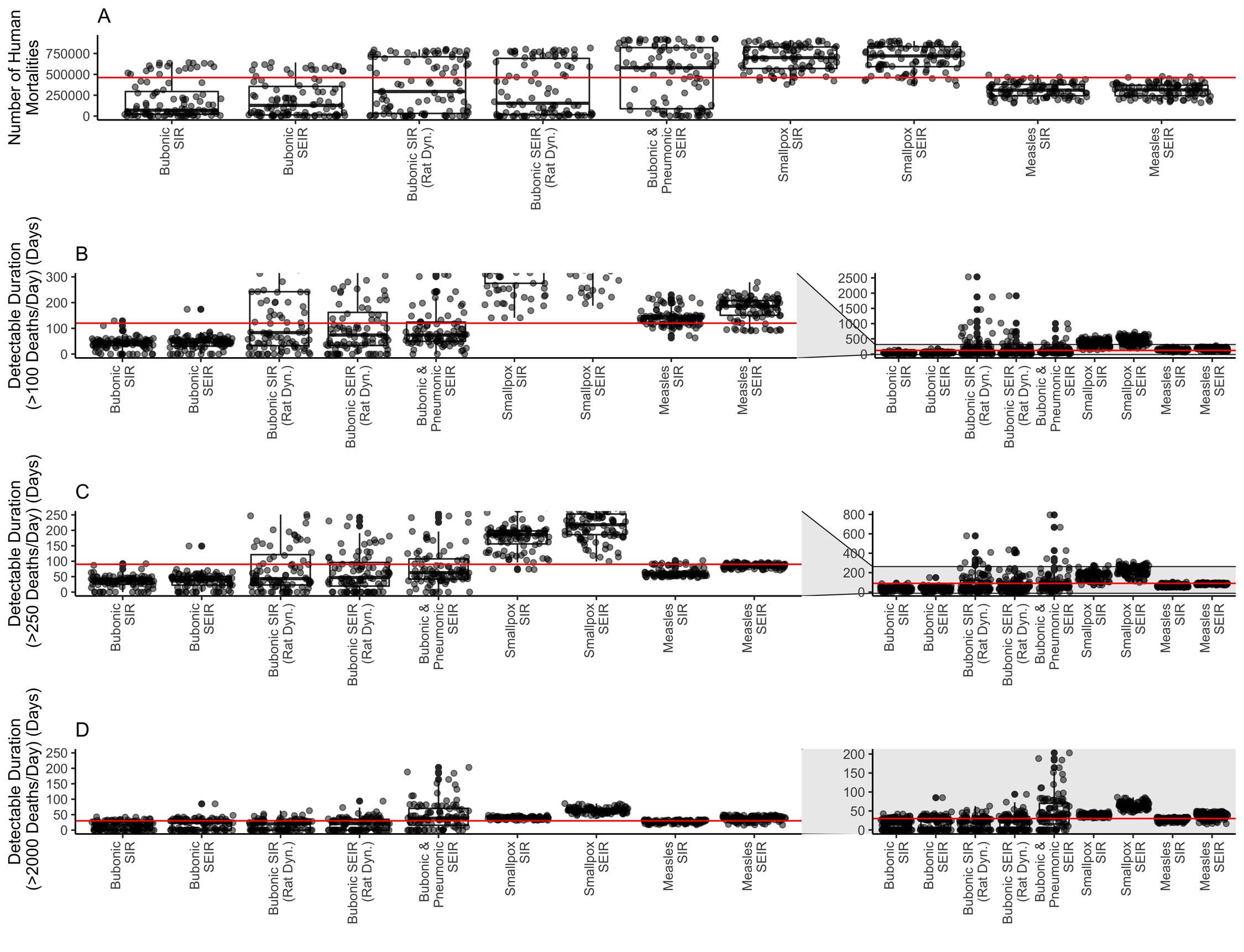

Supplement: S3 Fig — (A) Number of human mortalities; (B) detectable outbreak duration (>100 deaths per day, nonconsecutive) with inset including outliers; (C) detectable outbreak duration (>250 deaths per day); (D) detectable outbreak duration (>2,000 deaths per day). Red lines indicate half of the initial population of susceptible humans and the outbreak duration in months for contextualization of the panels: (A) 461,703 mortalities; (B) 4 months (120 days) at more than 100 deaths per day due to disease; (C) 3 months (90 days) at >250 deaths per day; (D) 1 month (30 days) at >2000 deaths per day. (TIF) [file pone.0313684.s003.tif]

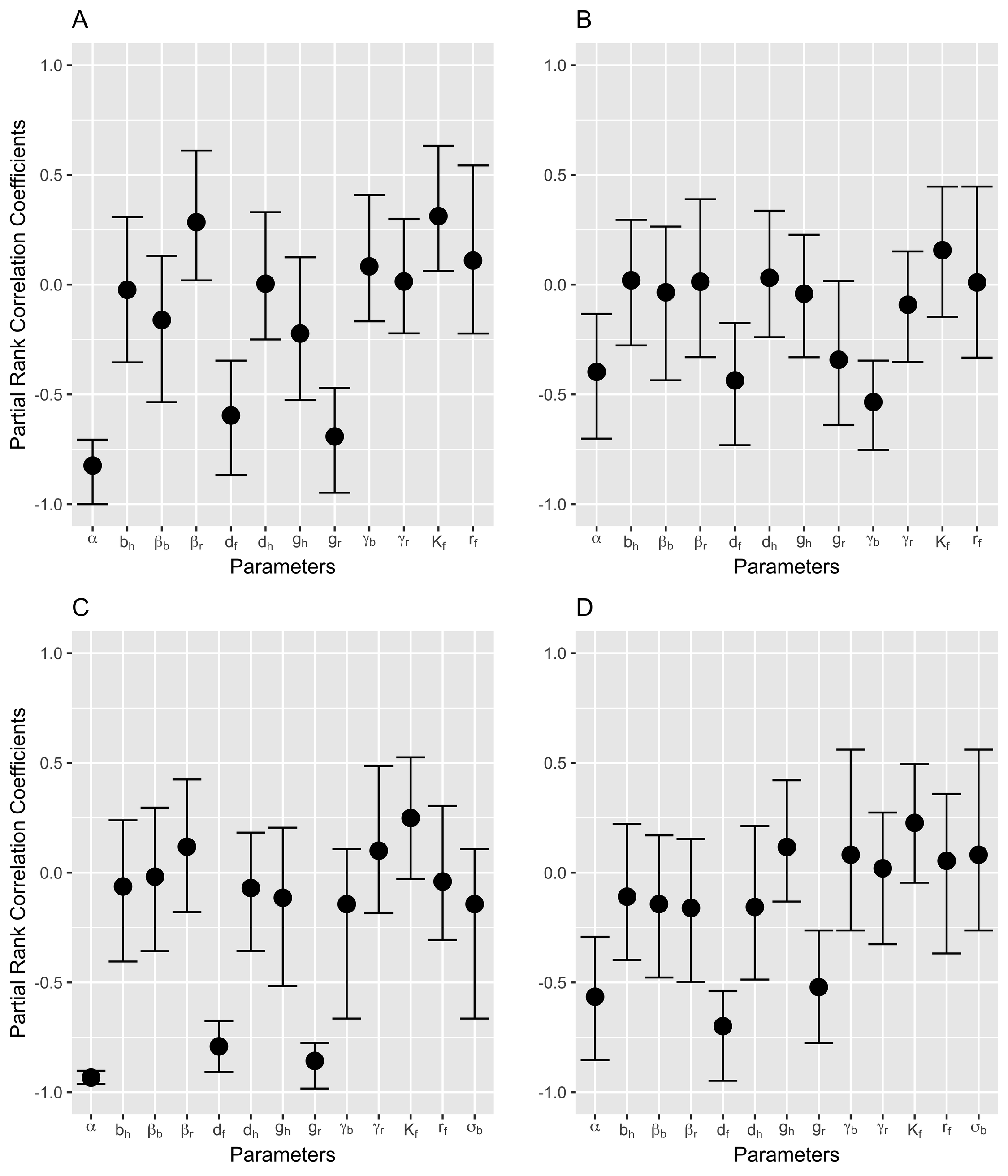

Supplement: S4 Fig — (A) Outbreak size in the SIR model (number of deaths due to disease); (B) outbreak duration in the SIR model (days); (C) outbreak size in SEIR; (D) outbreak duration in SEIR. (TIF) [file pone.0313684.s004.tif]

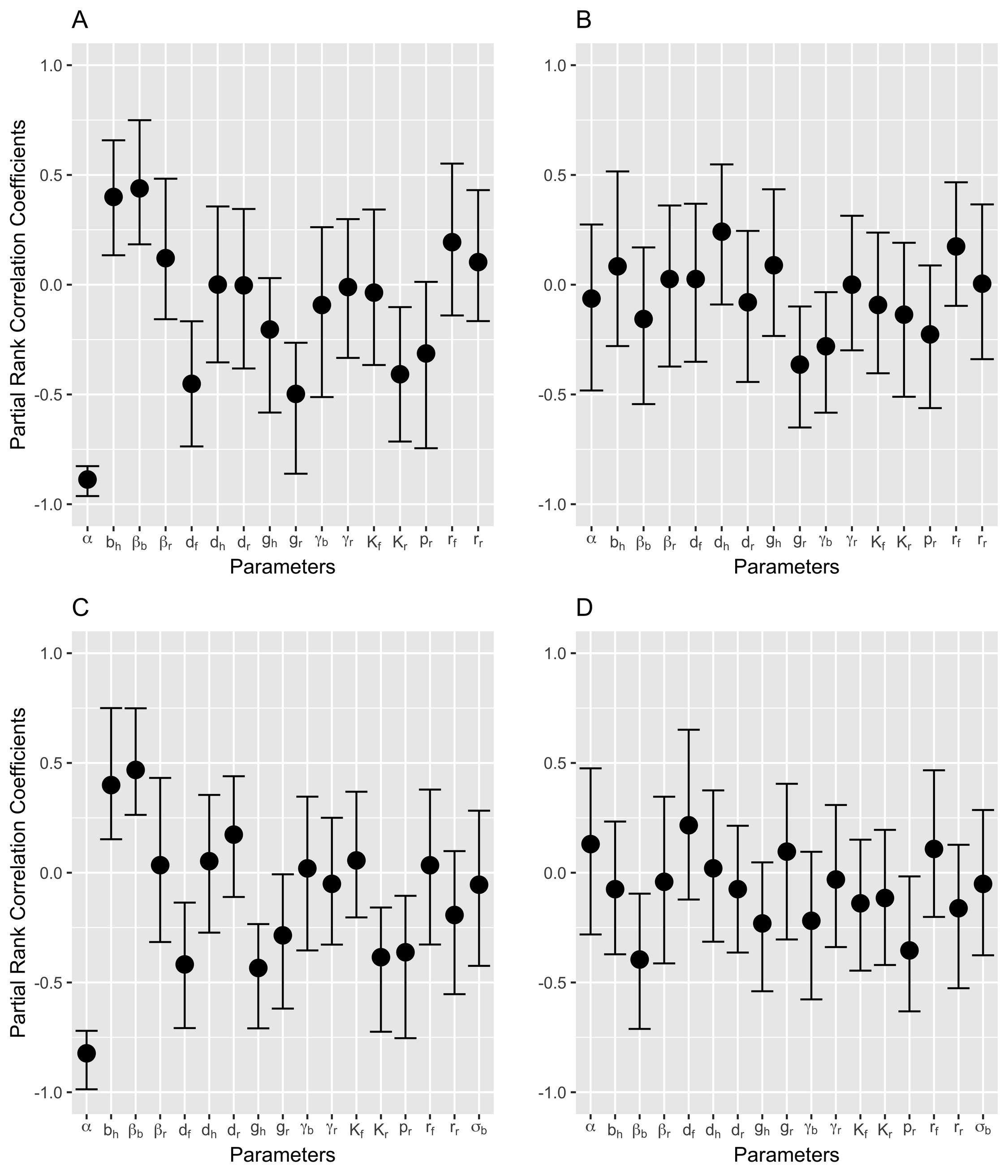

Supplement: S5 Fig — (A) Outbreak size in the SIR model (number of deaths due to disease); (B) outbreak duration in the SIR model (days); (C) outbreak size in SEIR; (D) outbreak duration in SEIR. (TIF) [file pone.0313684.s005.tif]

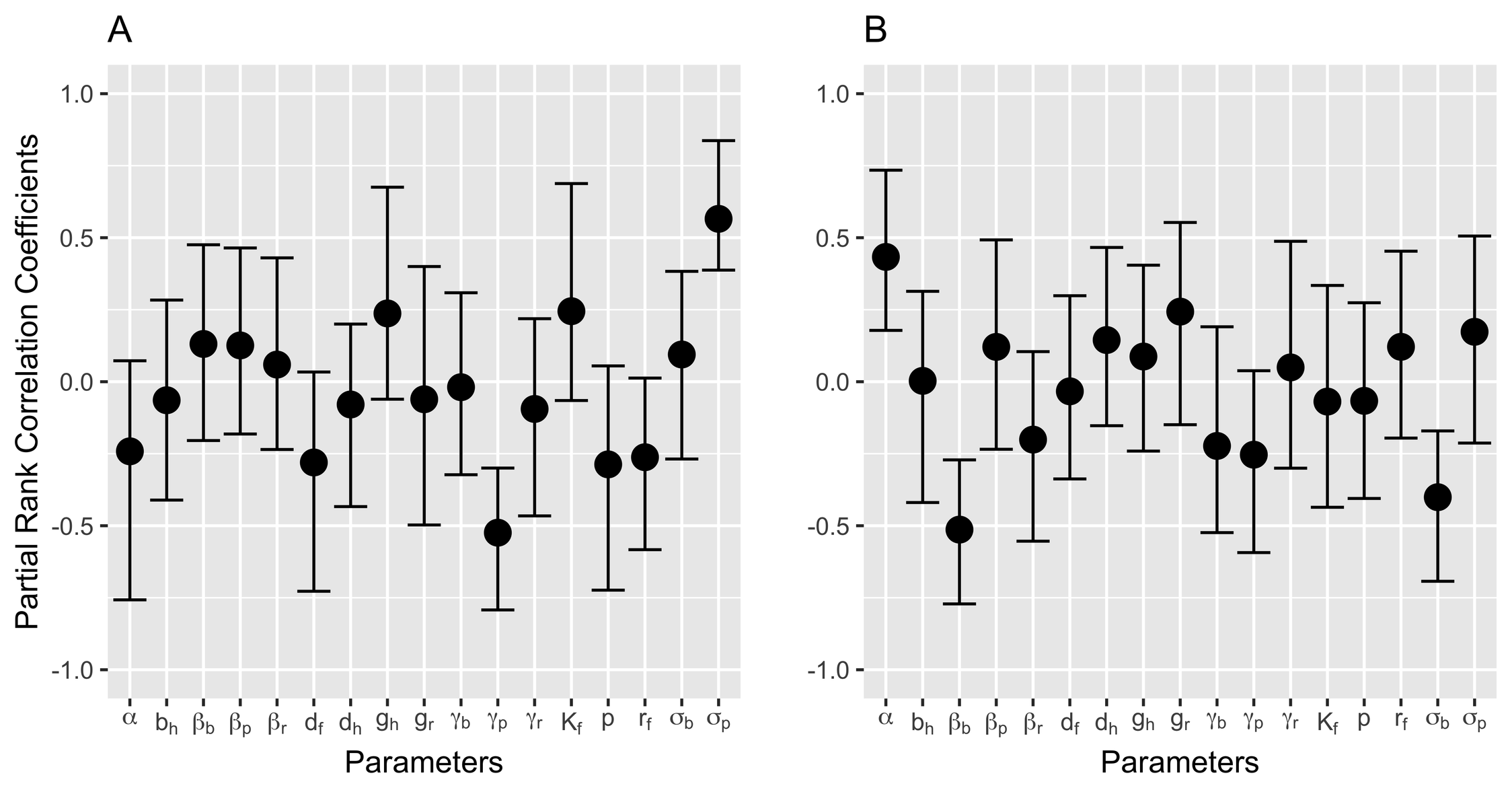

Supplement: S6 Fig — (A) Outbreak size (number of deaths due to disease); (B) outbreak duration (days). (TIF) [file pone.0313684.s006.tif]

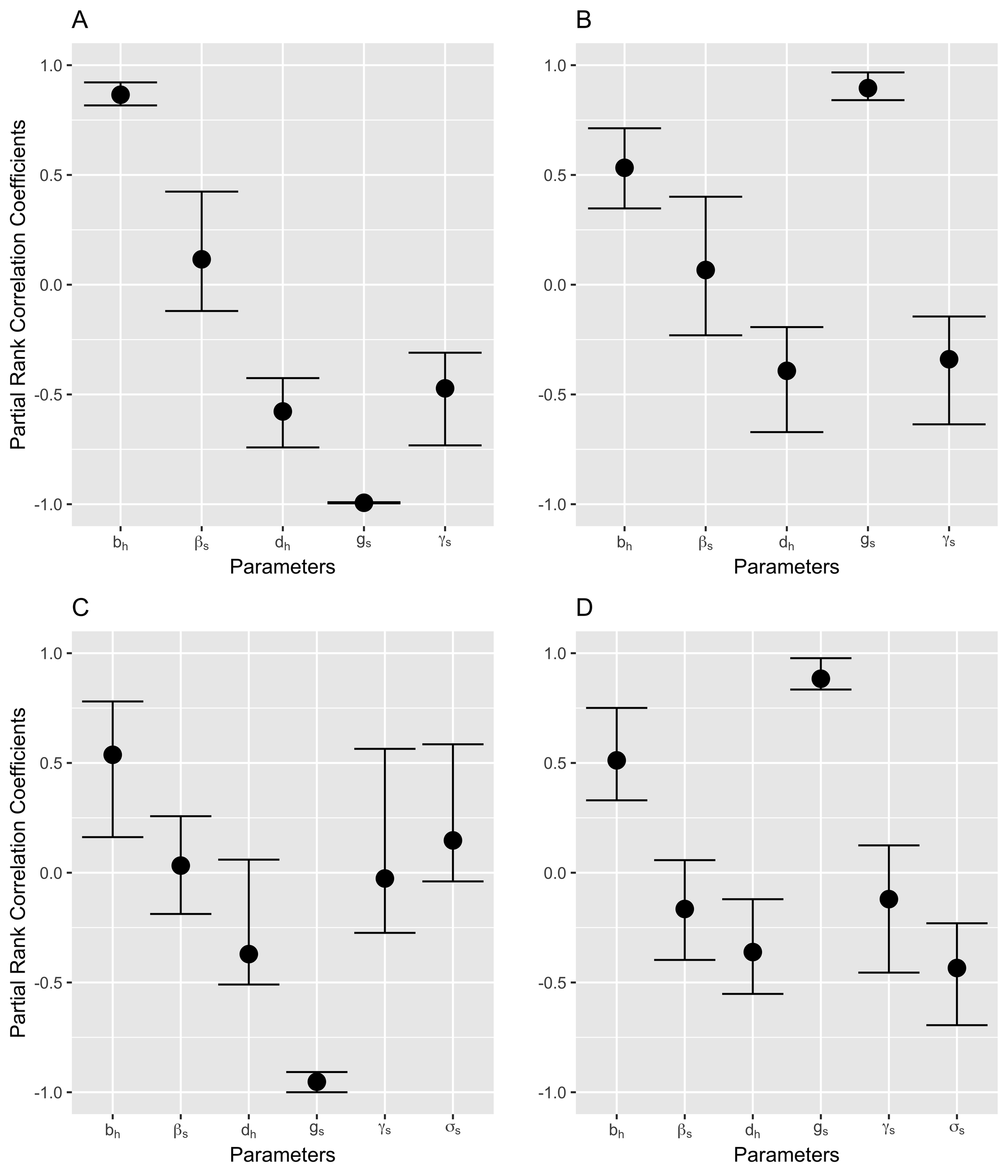

Supplement: S7 Fig — (A) Outbreak size in the SIR model (number of deaths due to disease); (B) outbreak duration in the SIR model (days); (C) outbreak size in SEIR; (D) outbreak duration in SEIR. (TIF) [file pone.0313684.s007.tif]

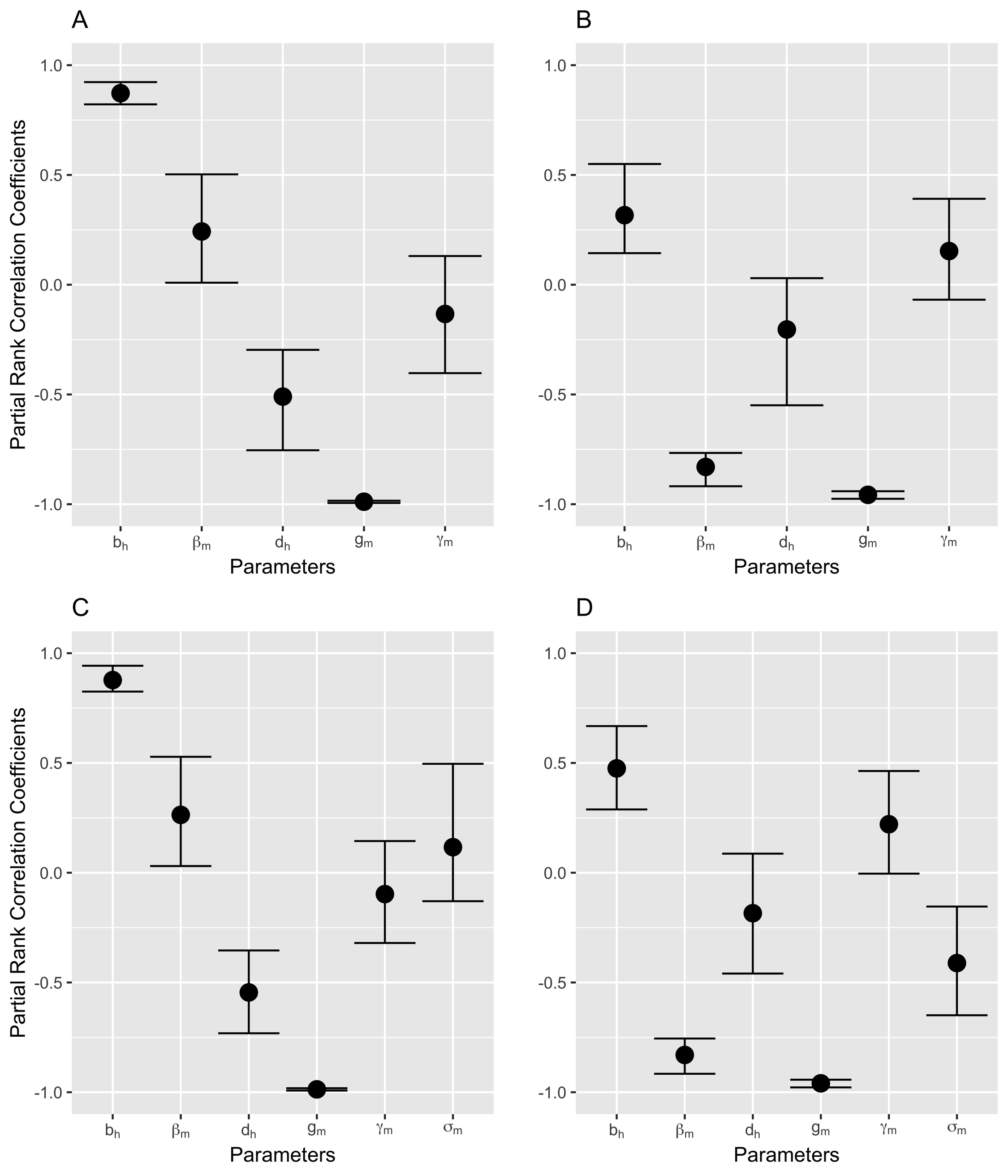

Supplement: S8 Fig — (A) Outbreak size in the SIR model (number of deaths due to disease); (B) outbreak duration in the SIR model (days); (C) outbreak size in SEIR; (D) outbreak duration in SEIR. (TIF) [file pone.0313684.s008.tif]
