# Supplementary material for: Comparative SIR/SEIR modeling of the Antonine Plague in Rome
Source: PLoS One. 2025 Feb 13;20(2):e0313684. doi: 10.1371/journal.pone.0313684 (PMC11824979; doi:10.1371/journal.pone.0313684)
Supplement: S1 Appendix — Contains scatter plots of parameters vs. model outcomes and PRCC plots. (PDF) [file pone.0313684.s009.pdf]

S1 Appendix: Uniform Sensitivity Analysis

Anestis Karasaridis (file adapted from an original by Lauren White)

January 11th, 2022

Sensitivity Analysis with Uniform Parameter Distributions

- Begin by creating LHS sampling space with `LHSnonuniform.R`

```
source("./Plague_model_functions.R")

require(lhs)  #add the lhs library
library(sensitivity)
require(ggplot2)
library(tidyverse)
library(ggforce)
require(deSolve)

set.seed(2718)  #set random seed
times <- seq(0, 9500, by = 1)
h <- 100  #choose number of parameter sets/subdivisions to sample to sample
N_r0 <- 923406  #initial conditions for ODE model- number of rats
niter <- 500  #number of times to bootstrap CI for PRCC

#' load uniform and non uniform LHS distributions
source("./LHSnonuniform.R")
uniform <- TRUE  #choose uniform (TRUE) or non-uniform (FALSE) distributions
```

Latin Hypercube Sampling and Partial Ranked Correlation Coefficients (LHS-PRCC)

- Use `GlobalSensitivityAnalysis.R` to run ODE models on LHS parameter sets produced in `LHSnonuniform.R`
- Plot scatter plots of each parameter vs. outbreak size and dectable outbreak duration (days)
- Calculate and plot PRCC values for each parameter for outbreak size and detectable outbreak duration (days)

```
source("./GlobalSensitivityAnalysis.R")
```

```
## DLSODA-  At current T (=R1), MXSTEP (=I1) steps
##       taken on this call before reaching TOUT
## In above message, I1 = 5000
```

```
##
## In above message, R1 = 7467.6
##
```

Define multiplot function

Use to produce multipaneled ggplot2 figures

```
#' Multiplot function
# ggplot objects can be passed in ..., or to plotlist (as a list of ggplot
# objects) - cols: Number of columns in layout - layout: A matrix specifying
# the layout. If present, 'cols' is ignored.  If the layout is something like
# matrix(c(1,2,3,3), nrow=2, byrow=TRUE), then plot 1 will go in the upper
# left, 2 will go in the upper right, and 3 will go all the way across the
# bottom.
multiplot <- function(..., plotlist = NULL, file, cols = 1, layout = NULL) {
    library(grid)

    # Make a list from the ... arguments and plotlist
    plots <- c(list(...), plotlist)

    numPlots = length(plots)

    # If layout is NULL, then use 'cols' to determine layout
    if (is.null(layout)) {
        # Make the panel ncol: Number of columns of plots nrow: Number of rows
        # needed, calculated from # of cols
        layout <- matrix(seq(1, cols * ceiling(numPlots/cols)), ncol = cols, nrow = ceiling(numPlots/col
            byrow = TRUE)
    }

    if (numPlots == 1) {
        print(plots[[1]])

    } else {
        # Set up the page
        grid.newpage()
        pushViewport(viewport(layout = grid.layout(nrow(layout), ncol(layout))))

        # Make each plot, in the correct location
        for (i in 1:numPlots) {
            # Get the i,j matrix positions of the regions that contain this
            # subplot
            matchidx <- as.data.frame(which(layout == i, arr.ind = TRUE))

            print(plots[[i]], vp = viewport(layout.pos.row = matchidx$row, layout.pos.col = matchidx$col
        }
    }
}
```

Comparative Figure Across Models

```
# Comparative Figure for All Models ---------------------------------------

comp_size <- data.frame(bSIR = bSIR$MaxInf, bSEIR = bSEIR$MaxInf, bSIRrK = bSIRrK$MaxInf,
    bSEIRrK = bSEIRrK$MaxInf, bpSEIR = bpSEIR$MaxInf, sSIR = sSIR$MaxInf, sSEIR = sSEIR$MaxInf,
    mSIR = mSIR$MaxInf, mSEIR = mSEIR$MaxInf)
comp_dur <- data.frame(bSIR = bSIR$Thresh100, bSEIR = bSEIR$Thresh100, bSIRrK = bSIRrK$Thresh100,
    bSEIRrK = bSEIRrK$Thresh100, bpSEIR = bpSEIR$Thresh100, sSIR = sSIR$Thresh100,
    sSEIR = sSEIR$Thresh100, mSIR = mSIR$Thresh100, mSEIR = mSEIR$Thresh100)
comp_dur250 <- data.frame(bSIR = bSIR$Thresh250, bSEIR = bSEIR$Thresh250, bSIRrK = bSIRrK$Thresh250,
    bSEIRrK = bSEIRrK$Thresh250, bpSEIR = bpSEIR$Thresh250, sSIR = sSIR$Thresh250,
    sSEIR = sSEIR$Thresh250, mSIR = mSIR$Thresh250, mSEIR = mSEIR$Thresh250)
comp_dur2000 <- data.frame(bSIR = bSIR$Thresh2000, bSEIR = bSEIR$Thresh2000, bSIRrK = bSIRrK$Thresh2000
    bSEIRrK = bSEIRrK$Thresh2000, bpSEIR = bpSEIR$Thresh2000, sSIR = sSIR$Thresh2000,
    sSEIR = sSEIR$Thresh2000, mSIR = mSIR$Thresh2000, mSEIR = mSEIR$Thresh2000)

long_DFsize <- comp_size %>%
    gather(Model, NumberDead, c(bSIR, bSEIR, bSIRrK, bSEIRrK, bpSEIR, sSIR, sSEIR,
        mSIR, mSEIR))
long_DFdur <- comp_dur %>%
    gather(Model, Duration, c(bSIR, bSEIR, bSIRrK, bSEIRrK, bpSEIR, sSIR, sSEIR,
        mSIR, mSEIR))
long_DFdur250 <- comp_dur250 %>%
    gather(Model, Duration, c(bSIR, bSEIR, bSIRrK, bSEIRrK, bpSEIR, sSIR, sSEIR,
        mSIR, mSEIR))
long_DFdur2000 <- comp_dur2000 %>%
    gather(Model, Duration, c(bSIR, bSEIR, bSIRrK, bSEIRrK, bpSEIR, sSIR, sSEIR,
        mSIR, mSEIR))

long_DFsize$Model <- as.character(long_DFsize$Model)
long_DFsize$Model <- factor(long_DFsize$Model, levels = c("bSIR", "bSEIR", "bSIRrK",
    "bSEIRrK", "bpSEIR", "sSIR", "sSEIR", "mSIR", "mSEIR"))

long_DFdur$Model <- as.character(long_DFdur$Model)
long_DFdur$Model <- factor(long_DFdur$Model, levels = c("bSIR", "bSEIR", "bSIRrK",
    "bSEIRrK", "bpSEIR", "sSIR", "sSEIR", "mSIR", "mSEIR"))

long_DFdur250$Model <- as.character(long_DFdur250$Model)
long_DFdur250$Model <- factor(long_DFdur250$Model, levels = c("bSIR", "bSEIR", "bSIRrK",
    "bSEIRrK", "bpSEIR", "sSIR", "sSEIR", "mSIR", "mSEIR"))

long_DFdur2000$Model <- as.character(long_DFdur2000$Model)
long_DFdur2000$Model <- factor(long_DFdur2000$Model, levels = c("bSIR", "bSEIR",
    "bSIRrK", "bSEIRrK", "bpSEIR", "sSIR", "sSEIR", "mSIR", "mSEIR"))

# Plot the first chart about size. The yintercept corresponds with half the
# cities population as in White's charts. The yintercept in the B, C and D
# charts indicate days of duration (4, 3 and 1 month, respectively).
A <- ggplot(long_DFsize, aes(Model, NumberDead)) + geom_boxplot() + geom_jitter(alpha = 0.5) +
    ylab("Number of Human\nMortalities") + xlab("") + scale_x_discrete(labels = c(bSIR = "Bubonic\nSIR"
    bSEIR = "Bubonic\nSEIR", bSIRrK = "Bubonic SIR\n(Rat Dyn.)", bpSEIR = "Bubonic &\nPneumonic\nSEIR",
    bSEIRrK = "Bubonic SEIR\n(Rat Dyn.)", sSIR = "Smallpox\nSIR", sSEIR = "Smallpox\nSEIR",
```

```
    mSIR = "Measles\nSIR", mSEIR = "Measles\nSEIR")) + geom_hline(yintercept = 923406/2,
    color = "red") + ggtitle("A") + theme_bw() + theme(panel.border = element_blank(),
    panel.grid.major = element_blank(), panel.grid.minor = element_blank(), axis.line = element_line(co
    axis.text.x = element_text(angle = 90, hjust = 1, vjust = 0.5))

B <- ggplot(long_DFdur, aes(Model, Duration)) + geom_boxplot() + geom_jitter(alpha = 0.5) +
    ylab("Detectable Duration\n (>100 Deaths/Day) (Days)") + xlab("") + scale_x_discrete(labels = c(bSI
    bSEIR = "Bubonic\nSEIR", bSIRrK = "Bubonic SIR\n(Rat Dyn.)", bpSEIR = "Bubonic &\nPneumonic\nSEIR",
    bSEIRrK = "Bubonic SEIR\n(Rat Dyn.)", sSIR = "Smallpox\nSIR", sSEIR = "Smallpox\nSEIR",
    mSIR = "Measles\nSIR", mSEIR = "Measles\nSEIR")) + theme(axis.text.x = element_text(angle = 90,
    hjust = 1)) + geom_hline(yintercept = 120, color = "red") + ggtitle("B") + theme_bw() +
    theme(panel.border = element_blank(), panel.grid.major = element_blank(), panel.grid.minor = elemen
        axis.line = element_line(colour = "black"), axis.text.x = element_text(angle = 90,
            hjust = 1, vjust = 0.5)) + facet_zoom(ylim = c(0, 300))

C <- ggplot(long_DFdur250, aes(Model, Duration)) + geom_boxplot() + geom_jitter(alpha = 0.5) +
    ylab("Detectable Duration\n (>250 Deaths/Day) (Days)") + xlab("") + scale_x_discrete(labels = c(bSI
    bSEIR = "Bubonic\nSEIR", bSIRrK = "Bubonic SIR\n(Rat Dyn.)", bpSEIR = "Bubonic &\nPneumonic\nSEIR",
    bSEIRrK = "Bubonic SEIR\n(Rat Dyn.)", sSIR = "Smallpox\nSIR", sSEIR = "Smallpox\nSEIR",
    mSIR = "Measles\nSIR", mSEIR = "Measles\nSEIR")) + theme(axis.text.x = element_text(angle = 90,
    hjust = 1)) + geom_hline(yintercept = 90, color = "red") + ggtitle("C") + theme_bw() +
    theme(panel.border = element_blank(), panel.grid.major = element_blank(), panel.grid.minor = elemen
        axis.line = element_line(colour = "black"), axis.text.x = element_text(angle = 90,
            hjust = 1, vjust = 0.5)) + facet_zoom(ylim = c(0, 250))

D <- ggplot(long_DFdur2000, aes(Model, Duration)) + geom_boxplot() + geom_jitter(alpha = 0.5) +
    ylab("Detectable Duration\n (>2000 Deaths/Day) (Days)") + xlab("") + scale_x_discrete(labels = c(bS
    bSEIR = "Bubonic\nSEIR", bSIRrK = "Bubonic SIR\n(Rat Dyn.)", bpSEIR = "Bubonic &\nPneumonic\nSEIR",
    bSEIRrK = "Bubonic SEIR\n(Rat Dyn.)", sSIR = "Smallpox\nSIR", sSEIR = "Smallpox\nSEIR",
    mSIR = "Measles\nSIR", mSEIR = "Measles\nSEIR")) + theme(axis.text.x = element_text(angle = 90,
    hjust = 1)) + geom_hline(yintercept = 30, color = "red") + ggtitle("D") + theme_bw() +
    theme(panel.border = element_blank(), panel.grid.major = element_blank(), panel.grid.minor = elemen
        axis.line = element_line(colour = "black"), axis.text.x = element_text(angle = 90,
            hjust = 1, vjust = 0.5)) + facet_zoom(ylim = c(0, 250))

tiff("S3_Fig.tiff", height = 22.23, width = 19.05, units = "cm", compression = "lzw",
    res = 600)
multiplot(A, B, C, D, cols = 1)
dev.off()
```

```
## pdf
##   2
```

Bubonic SIR model

```
parameters <- c(beta_r = 0.09, alpha = 3/923406, gamma_r = 1/5.15, g_r = 0.1, r_f = 0.0084,
    K_f = 6, d_f = 1/5, beta_h = 0.19, gamma_h = 1/10, g_h = 0.34, b_h = 1/(25 *
        365), d_h = 1/(25 * 365))  #you can play with transmission and recovery rates here

par(mfrow = c(1, 2))
plot(bSIR$MaxInf ~ bSIR$beta_r, main = expression(paste("Effect of ", beta[r], " on Size")),
```

```
    xlab = expression(beta[r]), ylab = "Outbreak Size")
plot(bSIR$Thresh100 ~ bSIR$beta_r, main = expression(paste("Effect of ", beta[r],
    " on Duration")), xlab = expression(beta[r]), ylab = "Detectable Duration (days)")
```

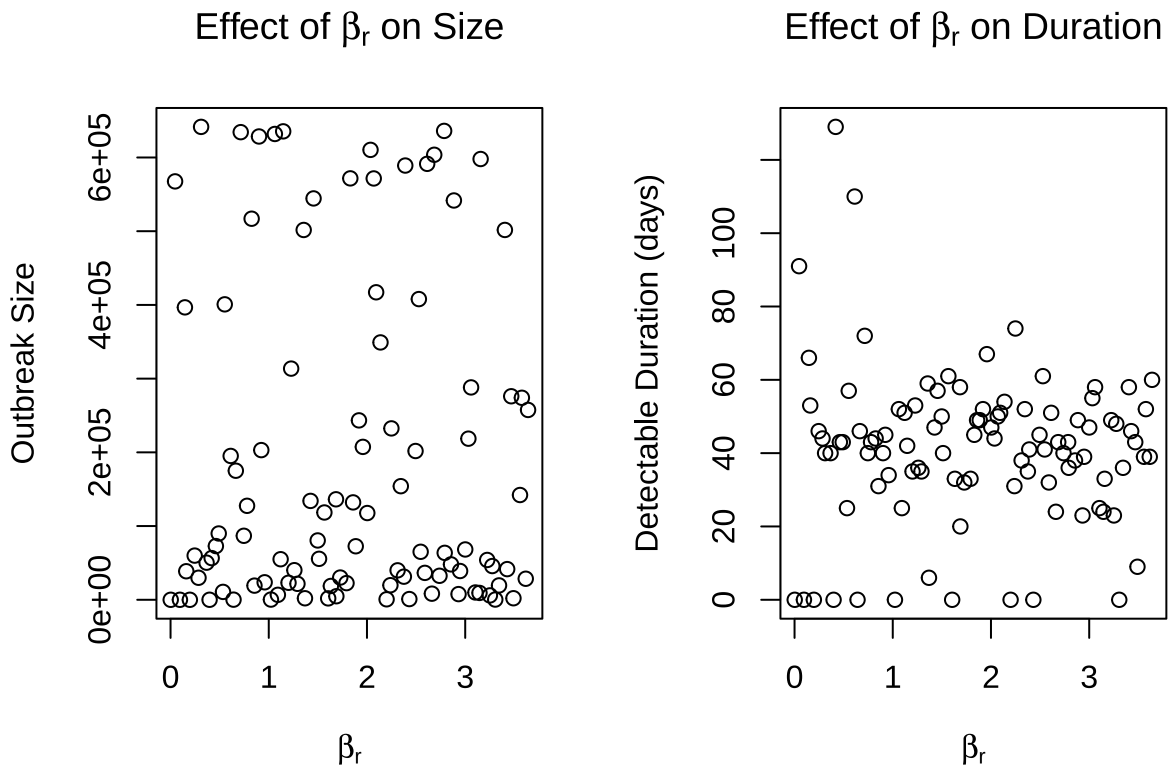


```
plot(bSIR$MaxInf ~ bSIR$alpha, main = expression(paste("Effect of ", alpha, " on Size")),
    xlab = expression(alpha), ylab = "Outbreak Size")
plot(bSIR$Thresh100 ~ bSIR$alpha, main = expression(paste("Effect of ", alpha, " on Duration")),
    xlab = expression(alpha), ylab = "Detectable Duration (days)")
```

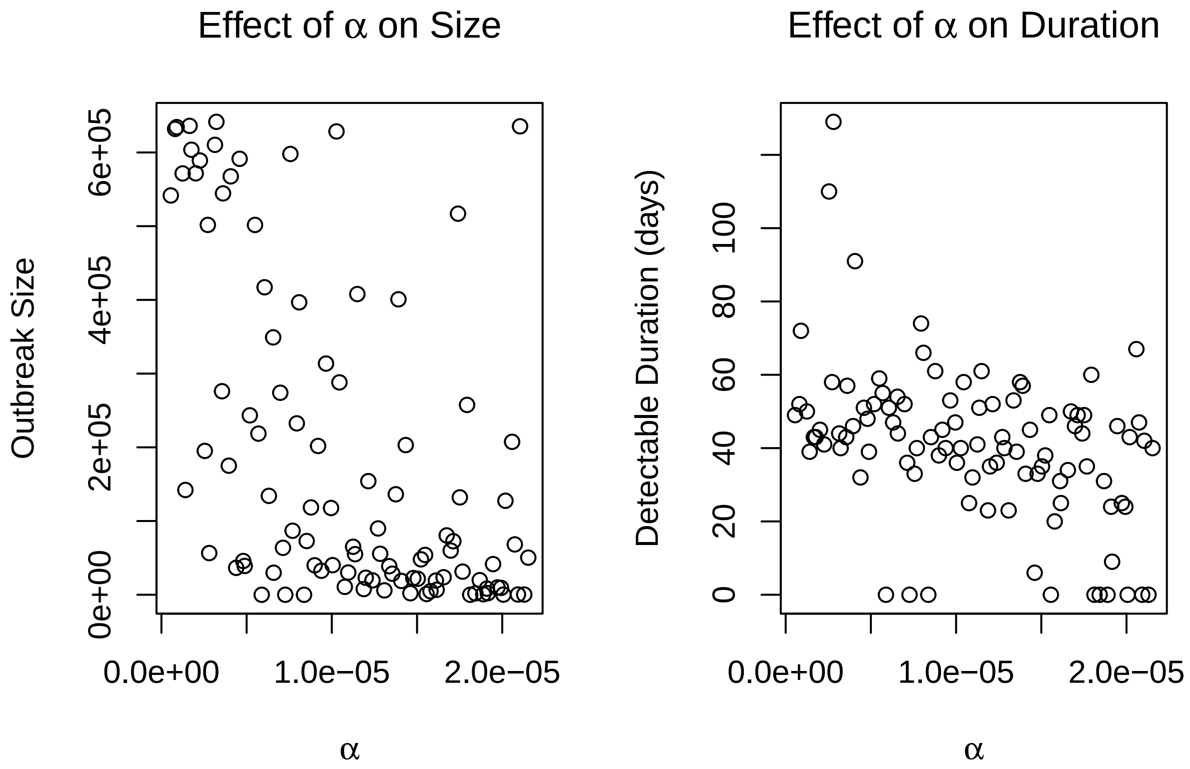


```r
plot(bSIR$MaxInf ~ bSIR$gamma_r, main = expression(paste("Effect of ", gamma[r],
    " on Size")), xlab = expression(gamma[r]), ylab = "Outbreak Size")
plot(bSIR$Thresh100 ~ bSIR$gamma_r, main = expression(paste("Effect of ", gamma[r],
    " on Duration")), xlab = expression(gamma[r]), ylab = "Detectable Duration (days)")
```

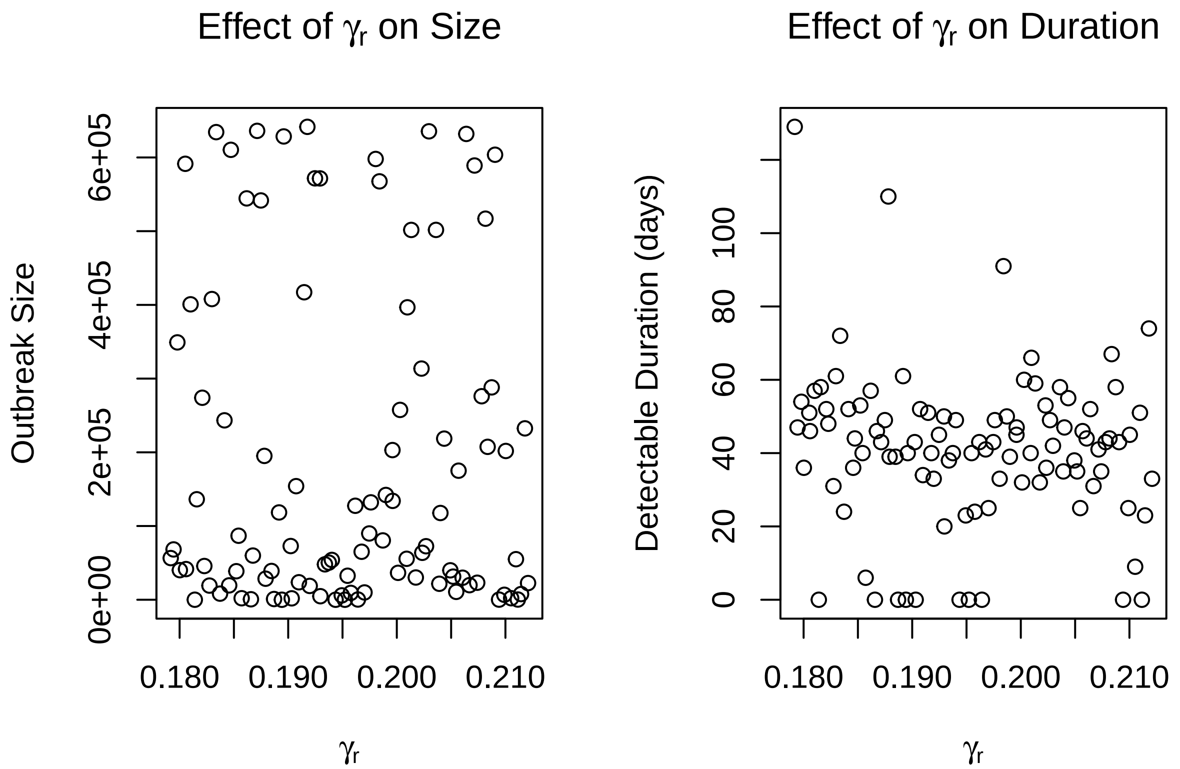


```
plot(bSIR$MaxInf ~ bSIR$g_r, main = expression(paste("Effect of ", g[r], " on Size")),
    xlab = expression(g[r]), ylab = "Outbreak Size")
plot(bSIR$Thresh100 ~ bSIR$g_r, main = expression(paste("Effect of ", g[r], " on Duration")),
    xlab = expression(g[r]), ylab = "Detectable Duration (days)")
```

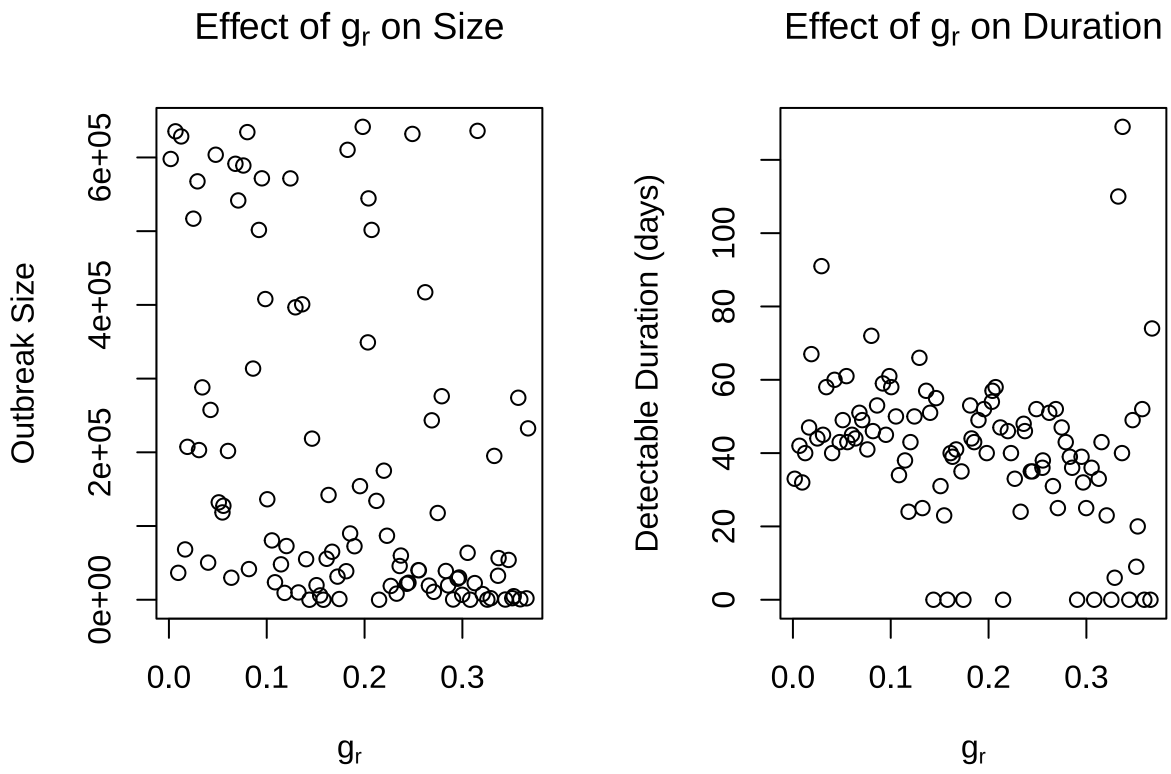


```
plot(bSIR$MaxInf ~ bSIR$r_f, main = expression(paste("Effect of ", r[f], " on Size")),
    xlab = expression(r[f]), ylab = "Outbreak Size")
plot(bSIR$Thresh100 ~ bSIR$r_f, main = expression(paste("Effect of ", r[f], " on Duration")),
    xlab = expression(r[f]), ylab = "Detectable Duration (days)")
```

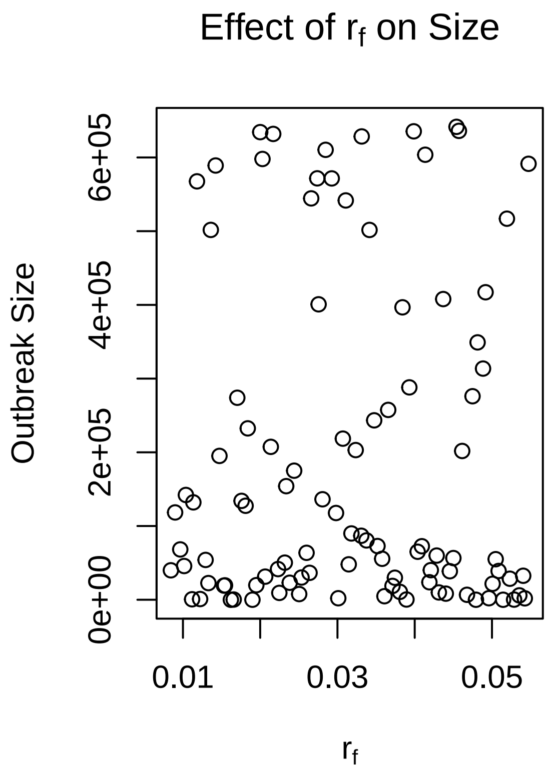

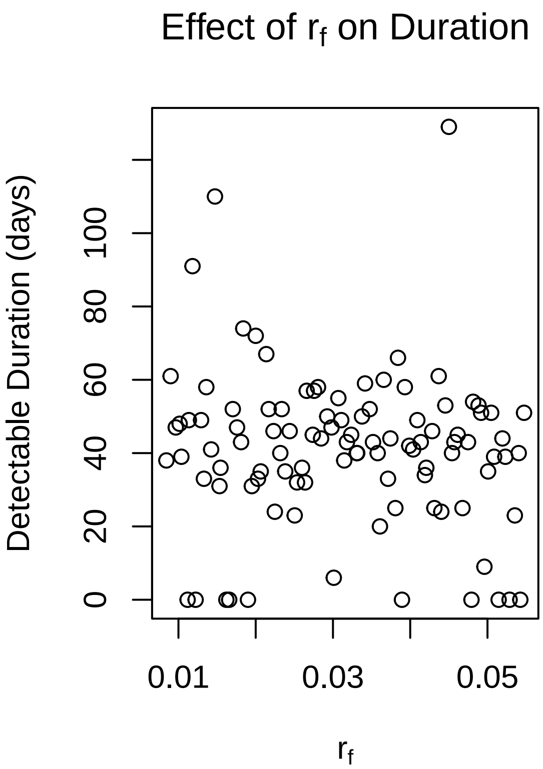


```
plot(bSIR$MaxInf ~ bSIR$K_f, main = expression(paste("Effect of ", K[f], " on Size")),
    xlab = expression(K[f]), ylab = "Outbreak Size")
plot(bSIR$Thresh100 ~ bSIR$K_f, main = expression(paste("Effect of ", K[f], " on Duration")),
    xlab = expression(K[f]), ylab = "Detectable Duration (days)")
```

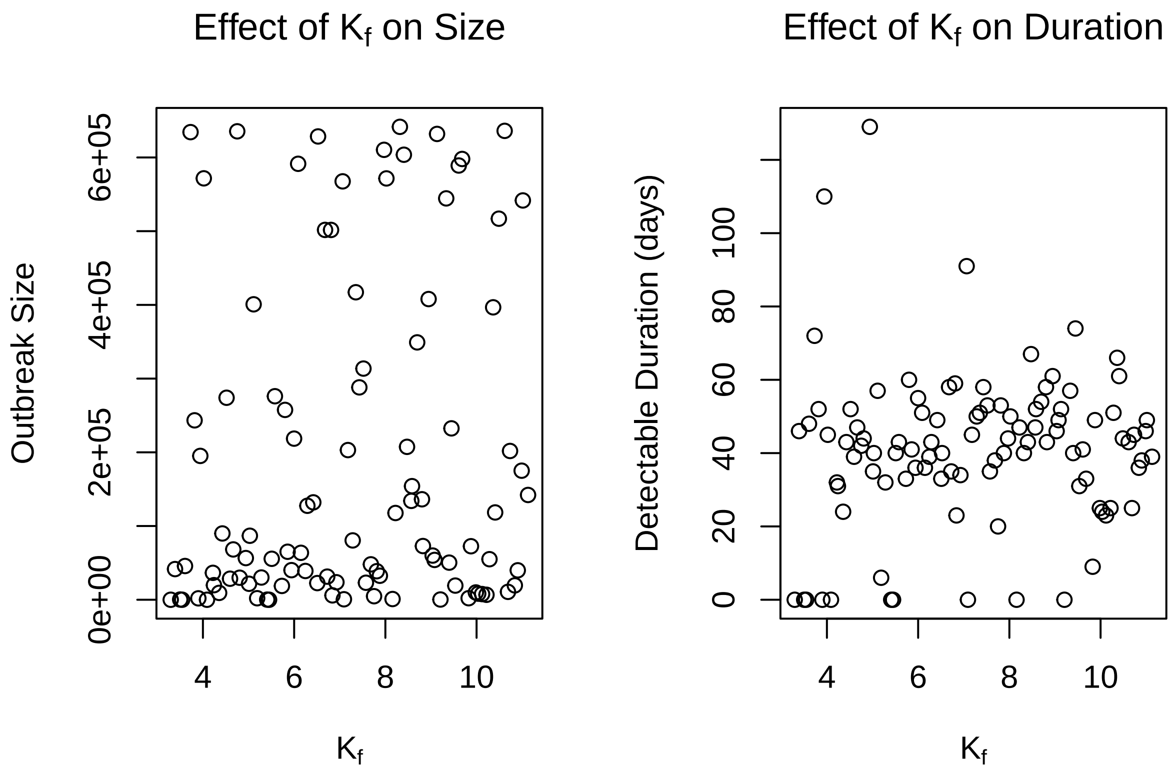


```
plot(bSIR$MaxInf ~ bSIR$d_f, main = expression(paste("Effect of ", d[f], " on Size")),
    xlab = expression(d[f]), ylab = "Outbreak Size")
plot(bSIR$Thresh100 ~ bSIR$d_f, main = expression(paste("Effect of ", d[f], " on Duration")),
    xlab = expression(d[f]), ylab = "Detectable Duration (days)")
```

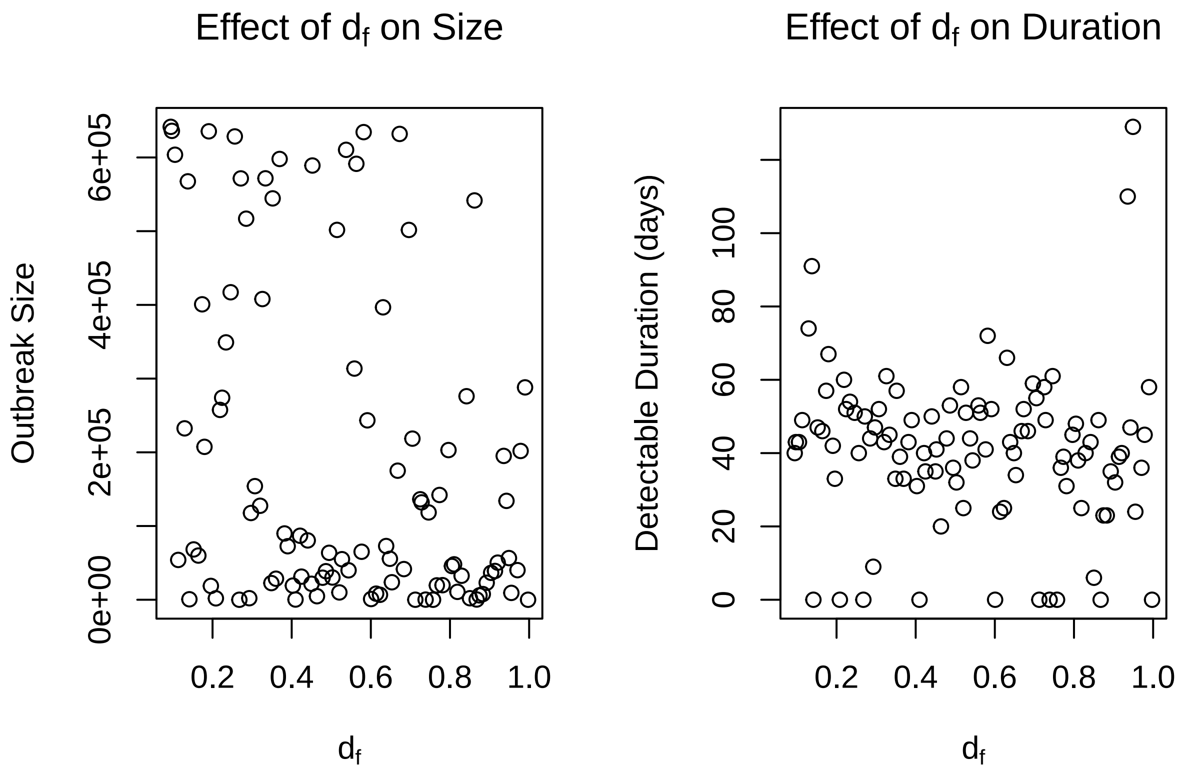


```
plot(bSIR$MaxInf ~ bSIR$beta_h, main = expression(paste("Effect of ", beta[b], " on Size")),
    xlab = expression(beta[b]), ylab = "Outbreak Size")
plot(bSIR$Thresh100 ~ bSIR$beta_h, main = expression(paste("Effect of ", beta[b],
    " on Duration")), xlab = expression(beta[b]), ylab = "Detectable Duration (days)")
```

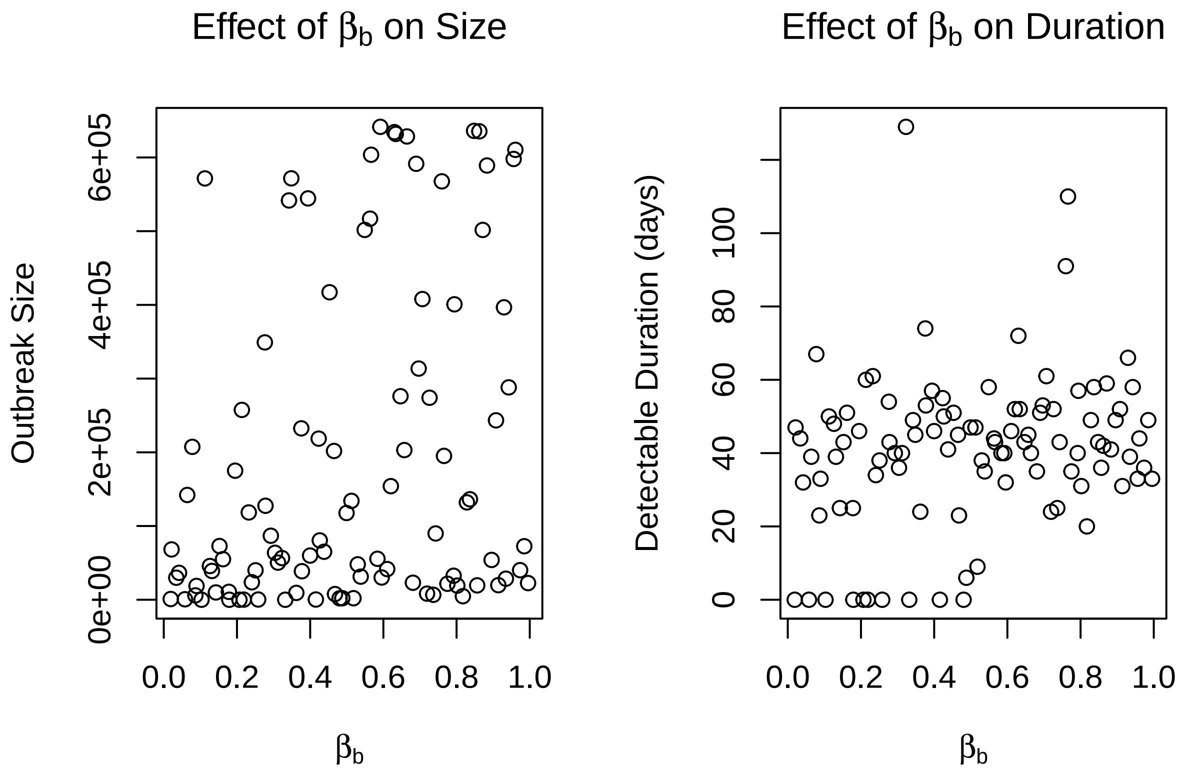


```
plot(bSIR$MaxInf ~ bSIR$gamma_h, main = expression(paste("Effect of ", gamma[b],
    " on Size")), xlab = expression(gamma[b]), ylab = "Outbreak Size")
plot(bSIR$Thresh100 ~ bSIR$gamma_h, main = expression(paste("Effect of ", gamma[b],
    " on Duration")), xlab = expression(gamma[b]), ylab = "Detectable Duration (days)")
```

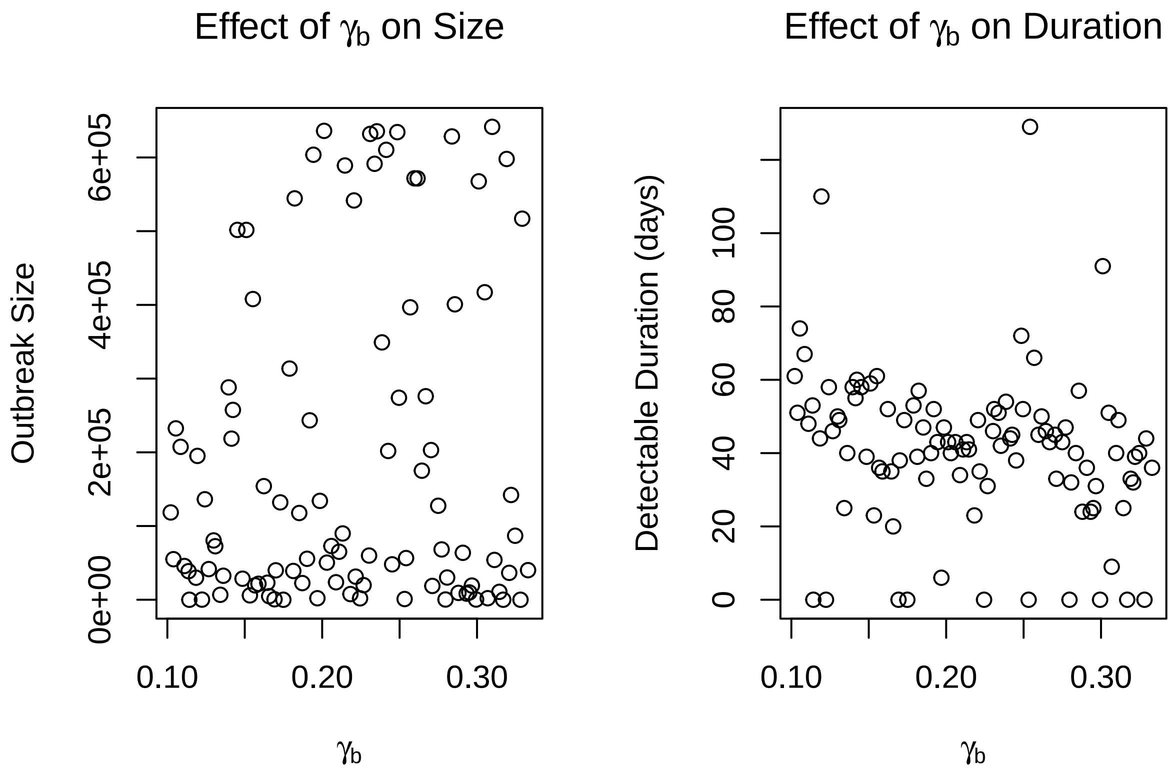


```
plot(bSIR$MaxInf ~ bSIR$g_h, main = expression(paste("Effect of ", g[h], " on Size")),
    xlab = expression(g[h]), ylab = "Outbreak Size")
plot(bSIR$Thresh100 ~ bSIR$g_h, main = expression(paste("Effect of ", g[h], " on Duration")),
    xlab = expression(g[h]), ylab = "Detectable Duration (days)")
```

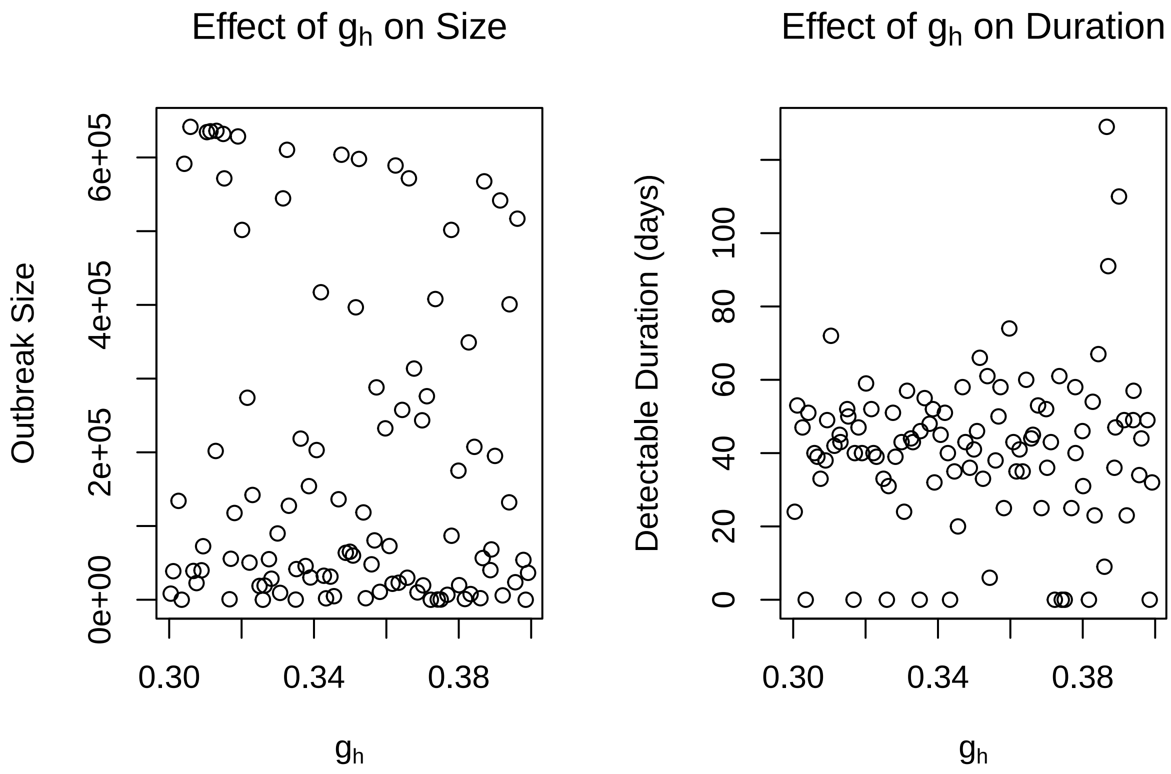


```
plot(bSIR$MaxInf ~ bSIR$b_h, main = expression(paste("Effect of ", b[h], " on Size")),
    xlab = expression(b[h]), ylab = "Outbreak Size")
plot(bSIR$Thresh100 ~ bSIR$b_h, main = expression(paste("Effect of ", b[h], " on Duration")),
    xlab = expression(b[h]), ylab = "Detectable Duration (days)")
```

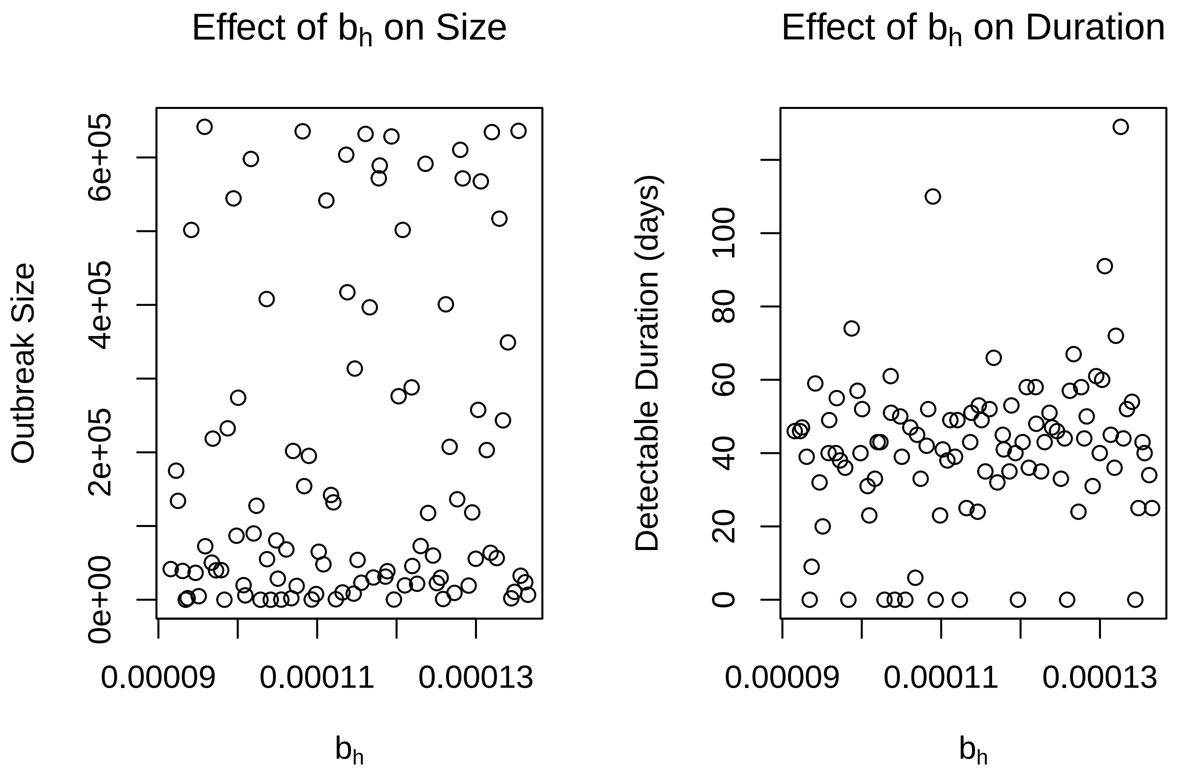


```
plot(bSIR$MaxInf ~ bSIR$d_h, main = expression(paste("Effect of ", d[h], " on Size")),
    xlab = expression(d[h]), ylab = "Outbreak Size")
plot(bSIR$Thresh100 ~ bSIR$d_h, main = expression(paste("Effect of ", d[h], " on Duration")),
    xlab = expression(d[h]), ylab = "Detectable Duration (days)")
```

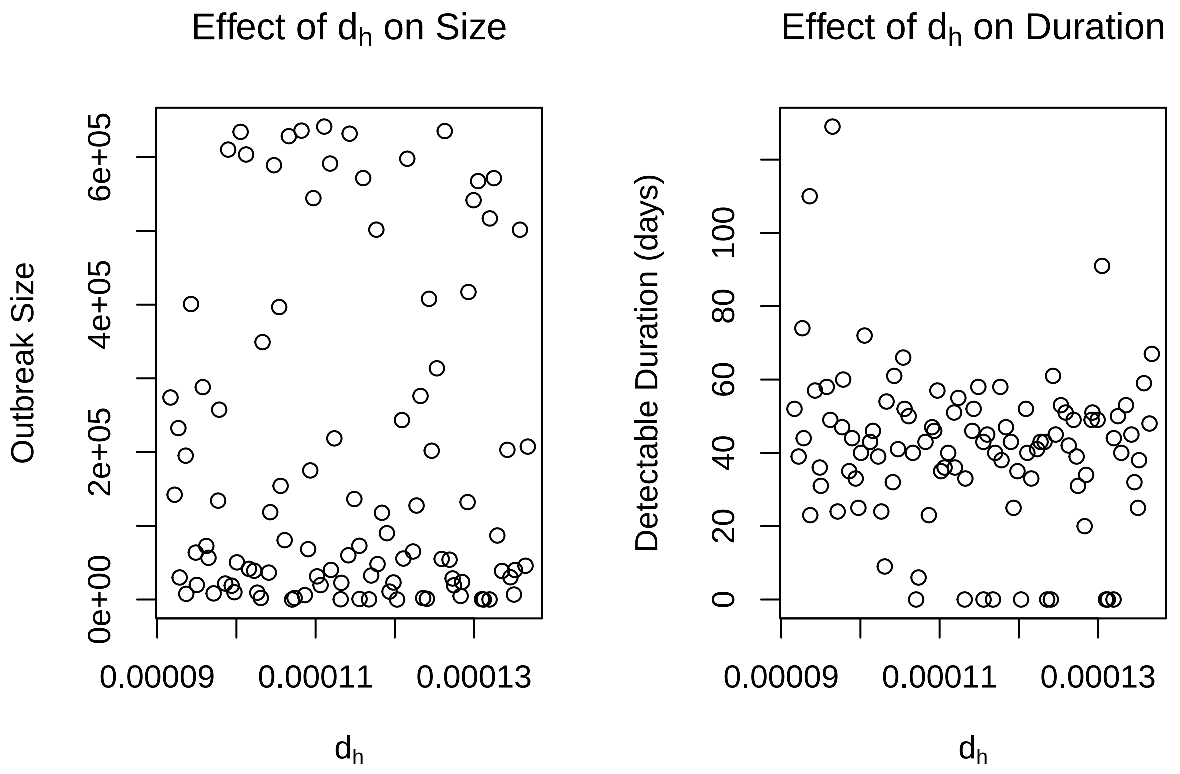


```
par(mfrow = c(1, 2))
boxplot(bSIR$MaxInf, main = "Outbreak Size", ylab = "Number of Dead Humans", ylim = c(0,
    923406))
boxplot(bSIR$Thresh100, main = "Outbreak Duration", ylab = "Time (Days)")
```

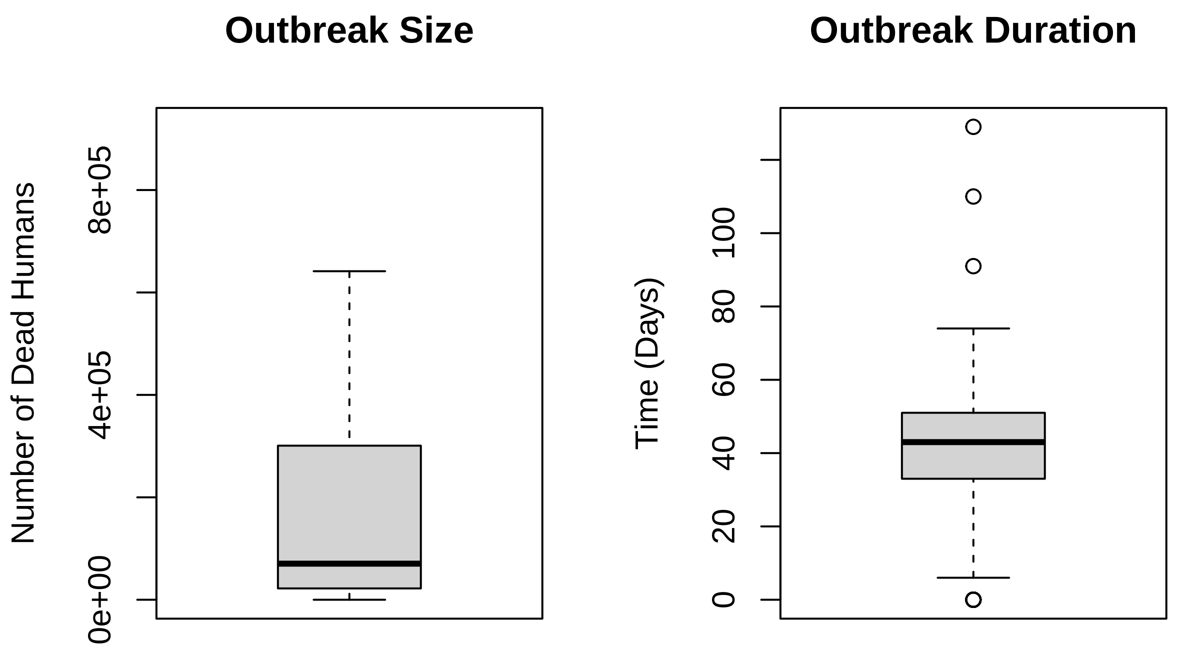


```r
bonferroni.alpha <- 0.05/length(parameters)
prcc_size <- pcc(bSIR[, 1:length(parameters)], bSIR$MaxInf, nboot = niter, rank = TRUE,
    conf = 1 - bonferroni.alpha)
prcc_duration <- pcc(bSIR[, 1:length(parameters)], bSIR$Thresh100, nboot = niter,
    rank = TRUE, conf = 1 - bonferroni.alpha)

# plot correlation coefficients and confidence intervals for epidemic size and
# duration
size <- prcc_size$PRCC
size$param <- rownames(size)
colnames(size)[4:5] <- c("maxCI", "minCI")
size$maxCI[which(size$maxCI > 1)] <- 1
size$maxCI[which(size$maxCI < -1)] <- -1
size$minCI[which(size$minCI > 1)] <- 1
size$minCI[which(size$minCI < -1)] <- -1

duration <- prcc_duration$PRCC
duration$param <- rownames(duration)
colnames(duration)[4:5] <- c("maxCI", "minCI")
duration$maxCI[which(duration$maxCI > 1)] <- 1
duration$maxCI[which(duration$maxCI < -1)] <- -1
duration$minCI[which(duration$minCI > 1)] <- 1
duration$minCI[which(duration$minCI < -1)] <- -1


A <- ggplot(size, aes(x = param, y = original)) + geom_point(size = 4) + geom_errorbar(aes(ymax = maxCI
```

```
    ymin = minCI)) + ggtitle("A") + xlab("Parameters") + ylab("Partial Rank Correlation Coefficients") +
    scale_x_discrete(labels = c(alpha = expression(alpha), beta_h = expression(beta[b]),
        beta_r = expression(beta[r]), b_h = expression(b[h]), d_h = expression(d[h]),
        d_f = expression(d[f]), gamma_h = expression(gamma[b]), gamma_r = expression(gamma[r]),
        g_h = expression(g[h]), g_r = expression(g[r]), K_f = expression(K[f]), r_f = expression(r[f])))
    ylim(-1, 1)

B <- ggplot(duration, aes(x = param, y = original)) + geom_point(size = 4) + geom_errorbar(aes(ymax = ma
    ymin = minCI)) + ggtitle("B") + xlab("Parameters") + ylab(" ") + scale_x_discrete(labels = c(alpha =
    beta_h = expression(beta[b]), beta_r = expression(beta[r]), b_h = expression(b[h]),
    d_h = expression(d[h]), d_f = expression(d[f]), gamma_h = expression(gamma[b]),
    gamma_r = expression(gamma[r]), g_h = expression(g[h]), g_r = expression(g[r]),
    K_f = expression(K[f]), r_f = expression(r[f]))) + ylim(-1, 1)

multiplot(A, B, cols = 2)
```

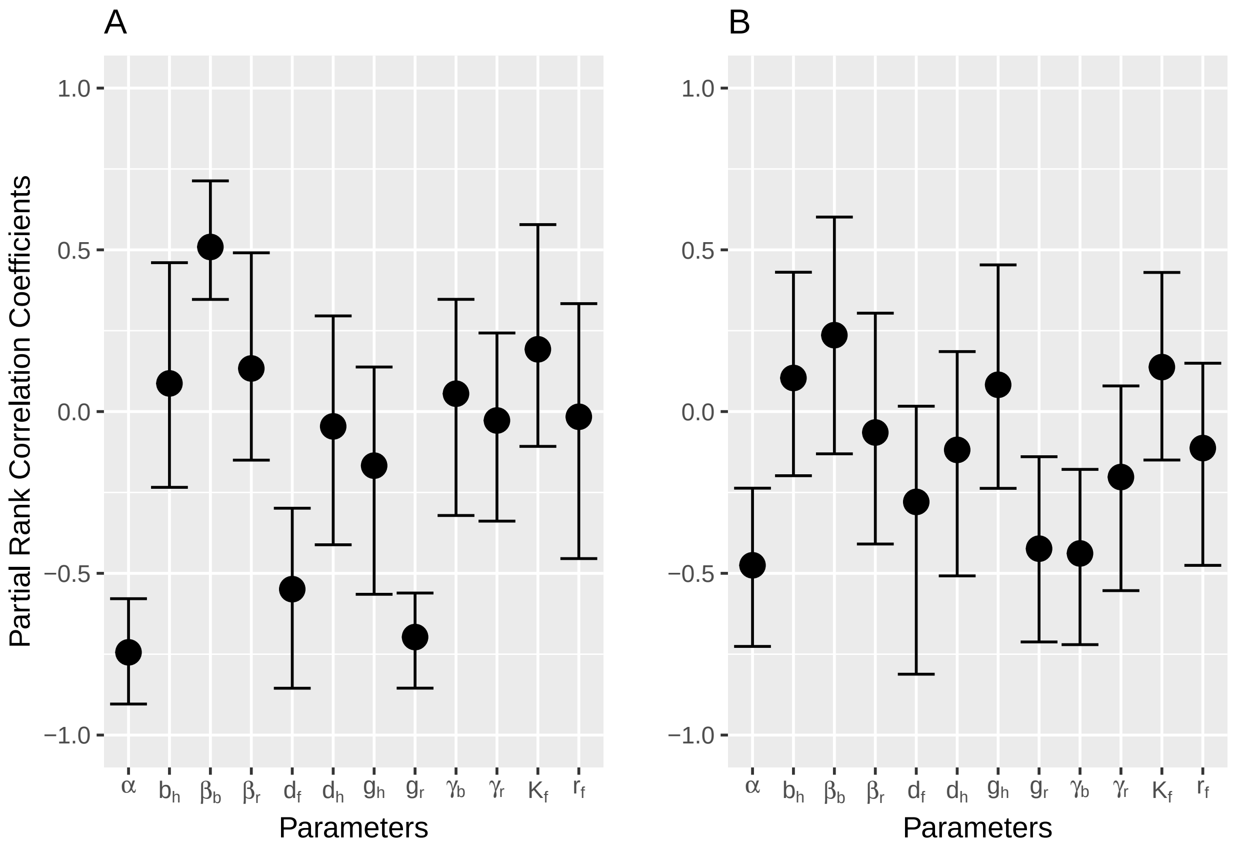


```
# tiff('FigurS13BubonicSIR_PRCCuniform.tiff', height =8.7 , width =10, units =
# 'cm', compression = 'lzw', res = 1200) multiplot(A, B, cols=2) dev.off()
```

Bubonic SEIR

```
parameters <- c(beta_r = 0.09, alpha = 3/923406, gamma_r = 1/5.15, g_r = 0.1, r_f = 0.0084,
    K_f = 6, d_f = 1/5, beta_h = 0.19, sigma_h = 1/4, gamma_h = 1/10, g_h = 0.34,
```

```
    b_h = 1/(25 * 365), d_h = 1/(25 * 365))  #you can play with transmission and recovery rates here

par(mfrow = c(1, 2))
plot(bSEIR$MaxInf ~ bSEIR$beta_r, main = expression(paste("Effect of ", beta[r],
    " on Size")), xlab = expression(beta[r]), ylab = "Outbreak Size")
plot(bSEIR$Thresh100 ~ bSEIR$beta_r, main = expression(paste("Effect of ", beta[r],
    " on Duration")), xlab = expression(beta[r]), ylab = "Detectable Duration (days)")
```

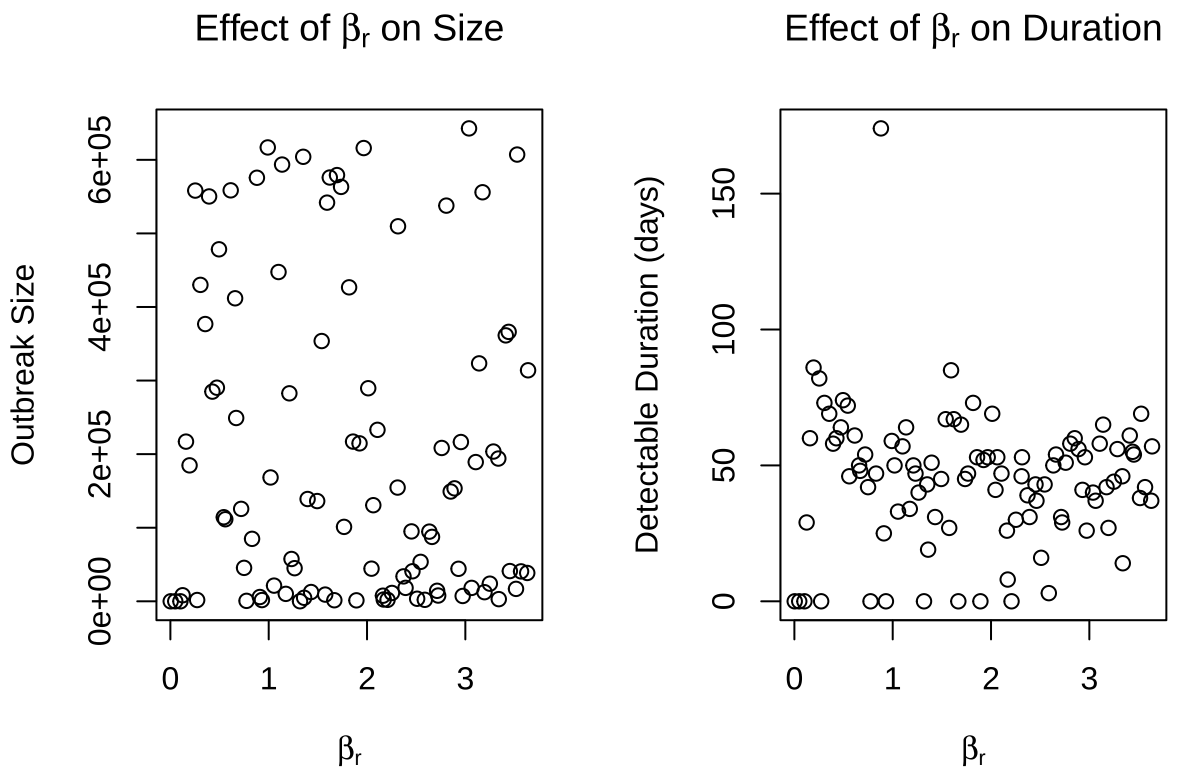


```
plot(bSEIR$MaxInf ~ bSEIR$alpha, main = expression(paste("Effect of ", alpha, " on Size")),
    xlab = expression(alpha), ylab = "Outbreak Size")
plot(bSEIR$Thresh100 ~ bSEIR$alpha, main = expression(paste("Effect of ", alpha,
    " on Duration")), xlab = expression(alpha), ylab = "Detectable Duration (days)")
```

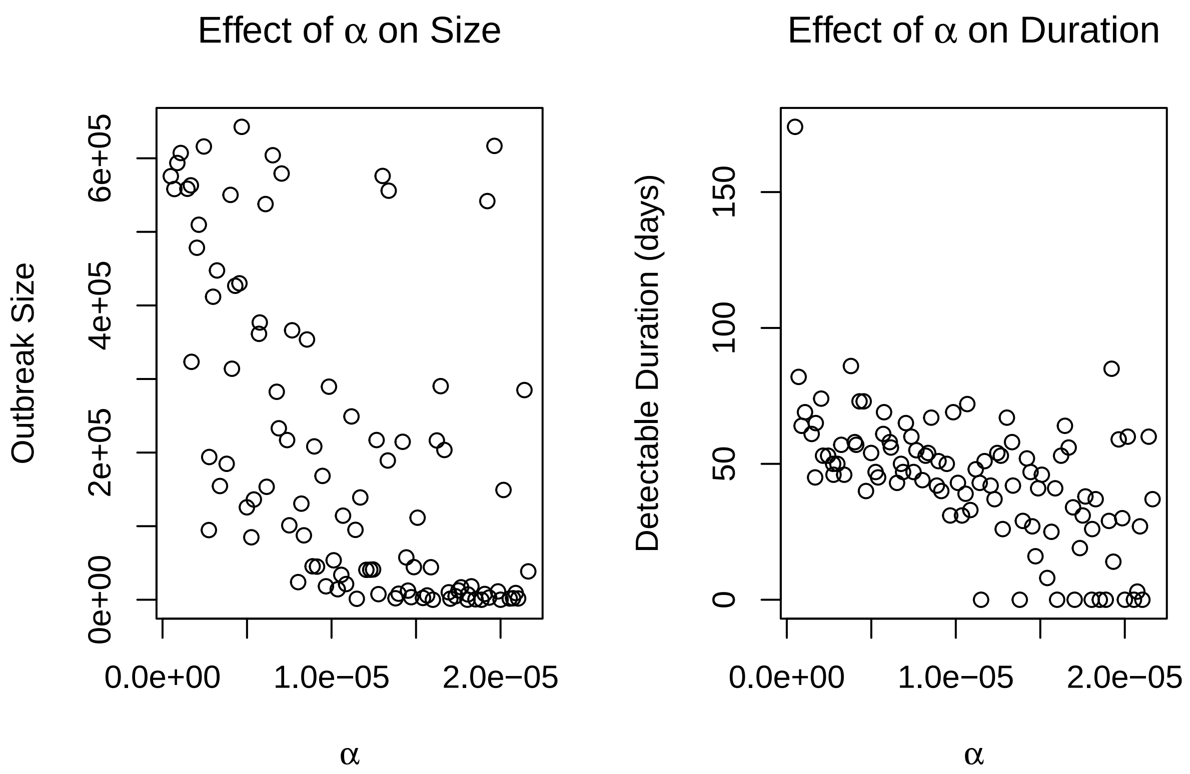


```
plot(bSEIR$MaxInf ~ bSEIR$gamma_r, main = expression(paste("Effect of ", gamma[r],
    " on Size")), xlab = expression(gamma[r]), ylab = "Outbreak Size")
plot(bSEIR$Thresh100 ~ bSEIR$gamma_r, main = expression(paste("Effect of ", gamma[r],
    " on Duration")), xlab = expression(gamma[r]), ylab = "Detectable Duration (days)")
```

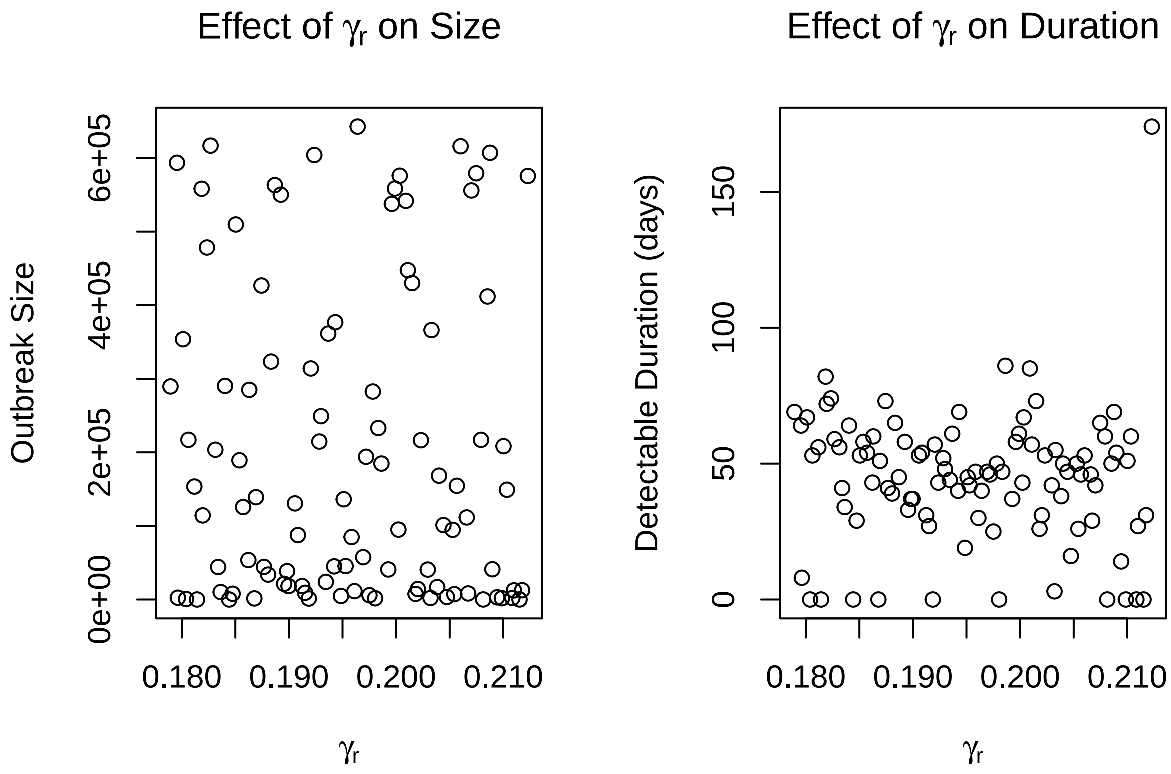


```
plot(bSEIR$MaxInf ~ bSEIR$g_r, main = expression(paste("Effect of ", g[r], " on Size")),
    xlab = expression(g[r]), ylab = "Outbreak Size")
plot(bSEIR$Thresh100 ~ bSEIR$g_r, main = expression(paste("Effect of ", g[r], " on Duration")),
    xlab = expression(g[r]), ylab = "Detectable Duration (days)")
```

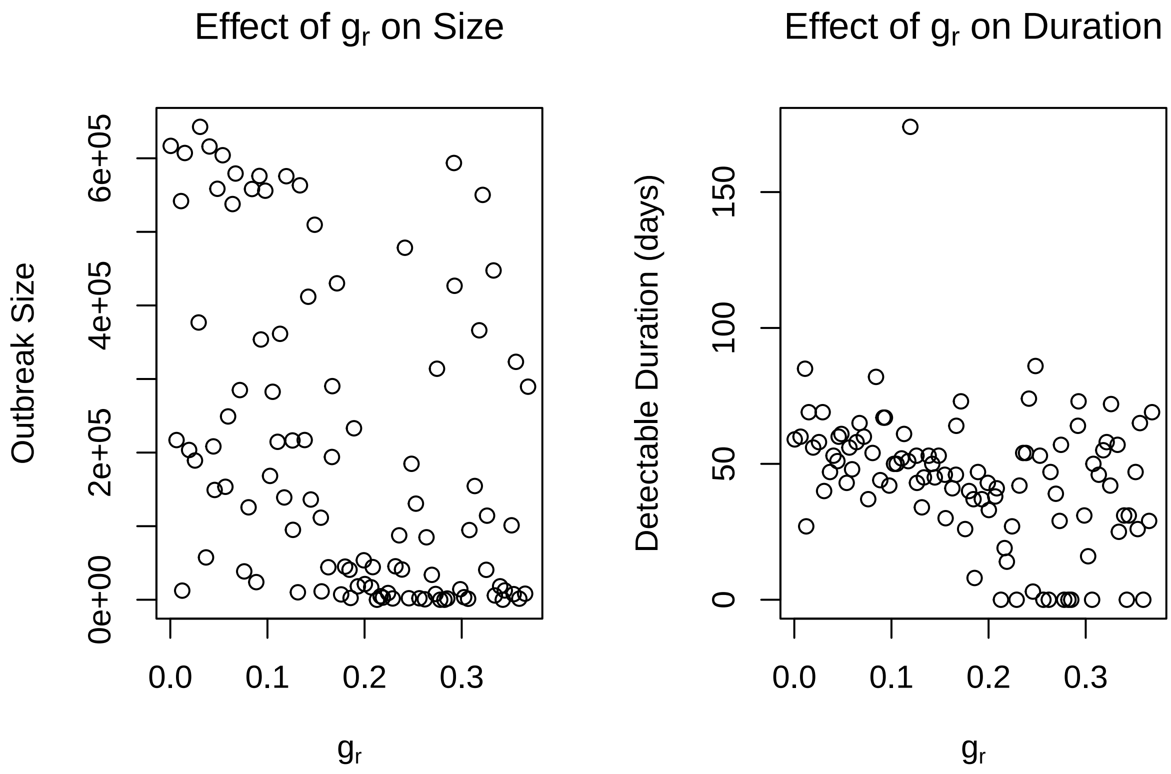


```
plot(bSEIR$MaxInf ~ bSEIR$r_f, main = expression(paste("Effect of ", r[f], " on Size")),
    xlab = expression(r[f]), ylab = "Outbreak Size")
plot(bSEIR$Thresh100 ~ bSEIR$r_f, main = expression(paste("Effect of ", r[f], " on Duration")),
    xlab = expression(r[f]), ylab = "Detectable Duration (days)")
```

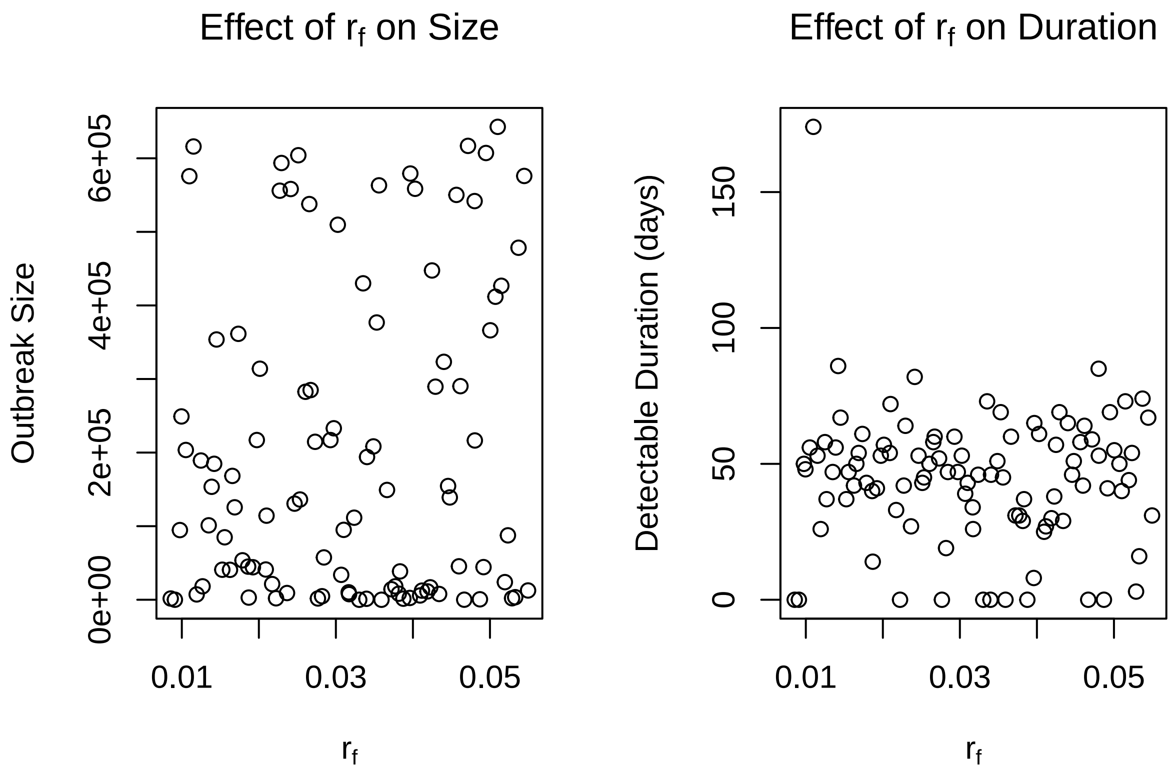


```
plot(bSEIR$MaxInf ~ bSEIR$K_f, main = expression(paste("Effect of ", K[f], " on Size")),
    xlab = expression(K[f]), ylab = "Outbreak Size")
plot(bSEIR$Thresh100 ~ bSEIR$K_f, main = expression(paste("Effect of ", K[f], " on Duration")),
    xlab = expression(K[f]), ylab = "Detectable Duration (days)")
```

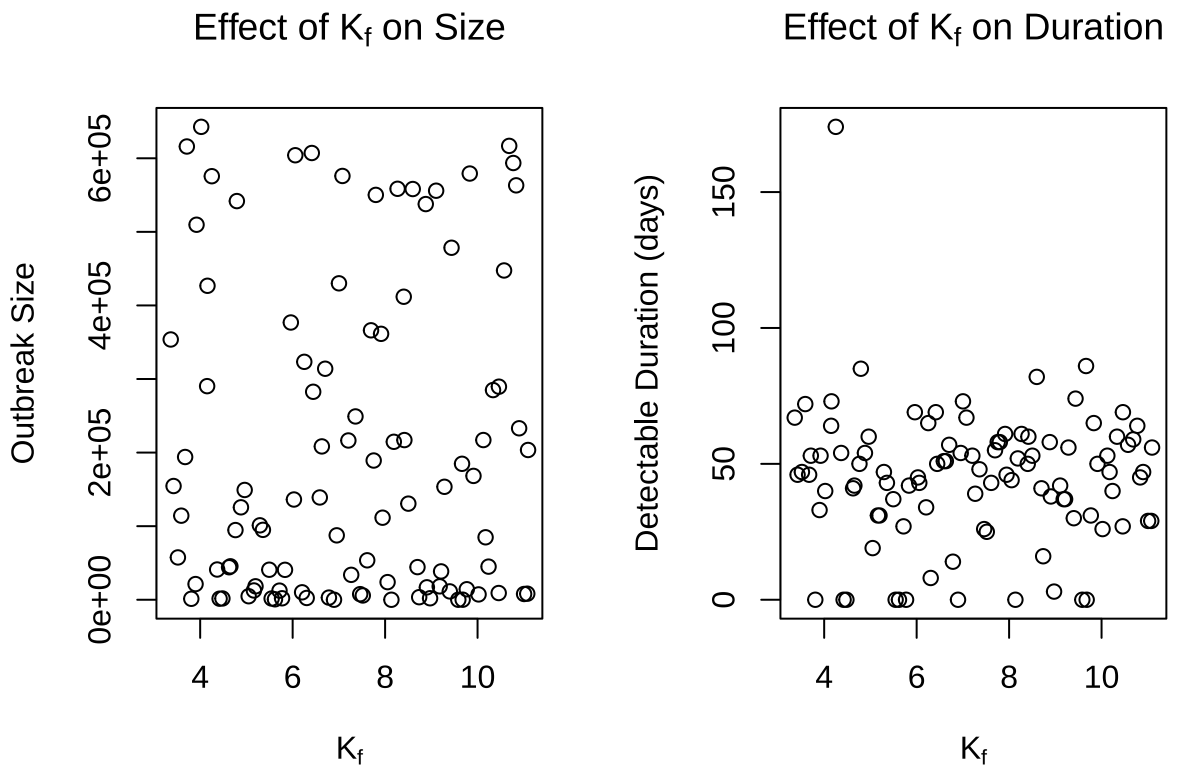


```
plot(bSEIR$MaxInf ~ bSEIR$d_f, main = expression(paste("Effect of ", d[f], " on Size")),
    xlab = expression(d[f]), ylab = "Outbreak Size")
plot(bSEIR$Thresh100 ~ bSEIR$d_f, main = expression(paste("Effect of ", d[f], " on Duration")),
    xlab = expression(d[f]), ylab = "Detectable Duration (days)")
```

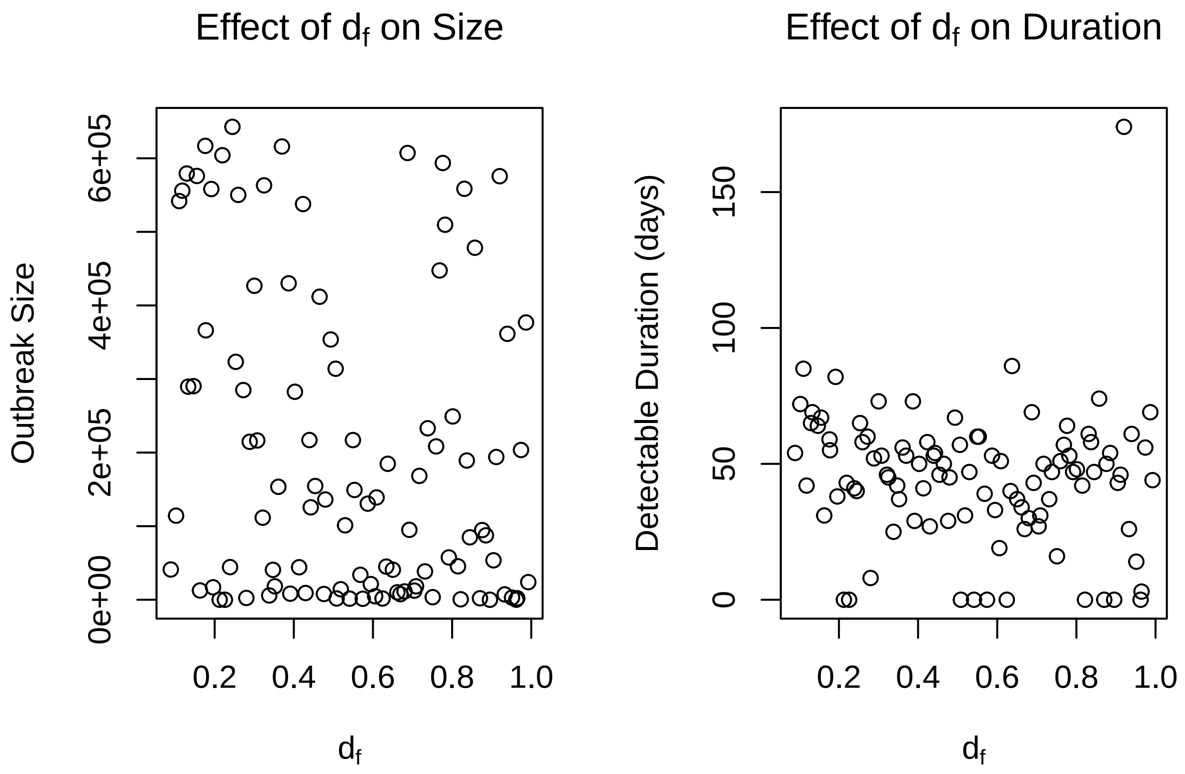


```
plot(bSEIR$MaxInf ~ bSEIR$beta_h, main = expression(paste("Effect of ", beta[b],
    " on Size")), xlab = expression(beta[b]), ylab = "Outbreak Size")
plot(bSEIR$Thresh100 ~ bSEIR$beta_h, main = expression(paste("Effect of ", beta[b],
    " on Duration")), xlab = expression(beta[b]), ylab = "Detectable Duration (days)")
```

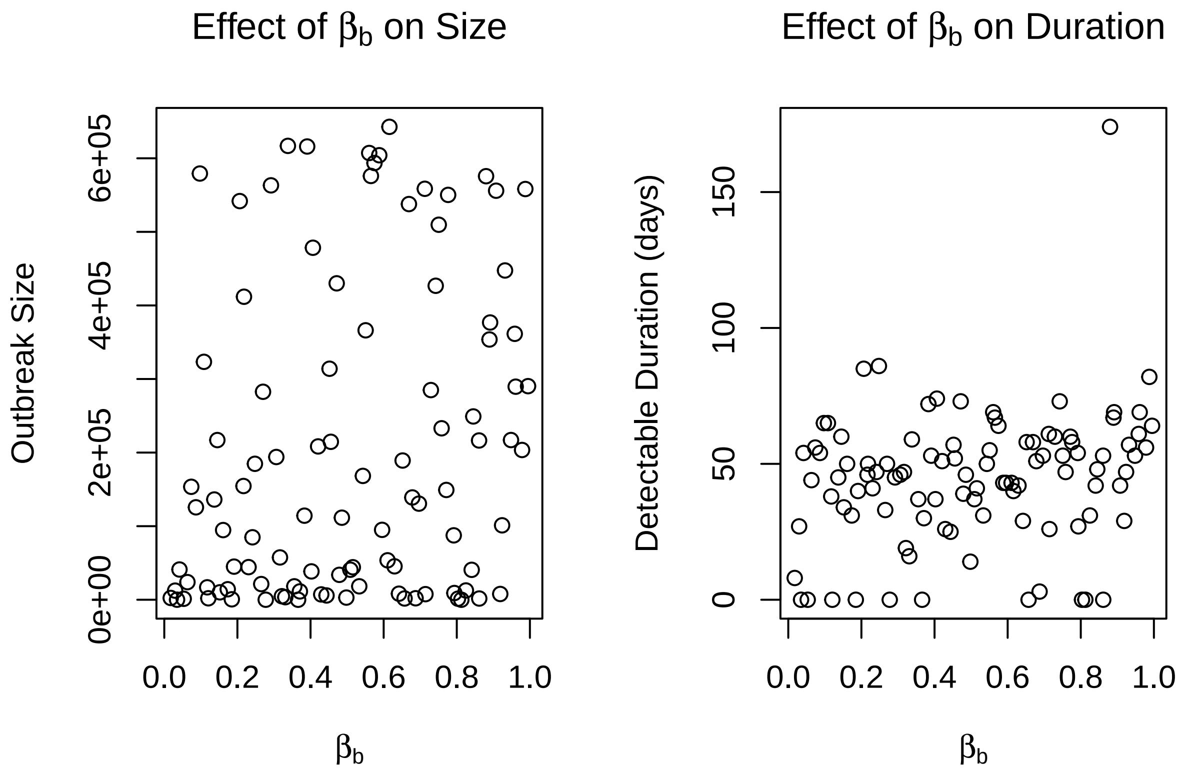


```
plot(bSEIR$MaxInf ~ bSEIR$gamma_h, main = expression(paste("Effect of ", gamma[b],
    " on Size")), xlab = expression(gamma[b]), ylab = "Outbreak Size")
plot(bSEIR$Thresh100 ~ bSEIR$gamma_h, main = expression(paste("Effect of ", gamma[b],
    " on Duration")), xlab = expression(gamma[b]), ylab = "Detectable Duration (days)")
```

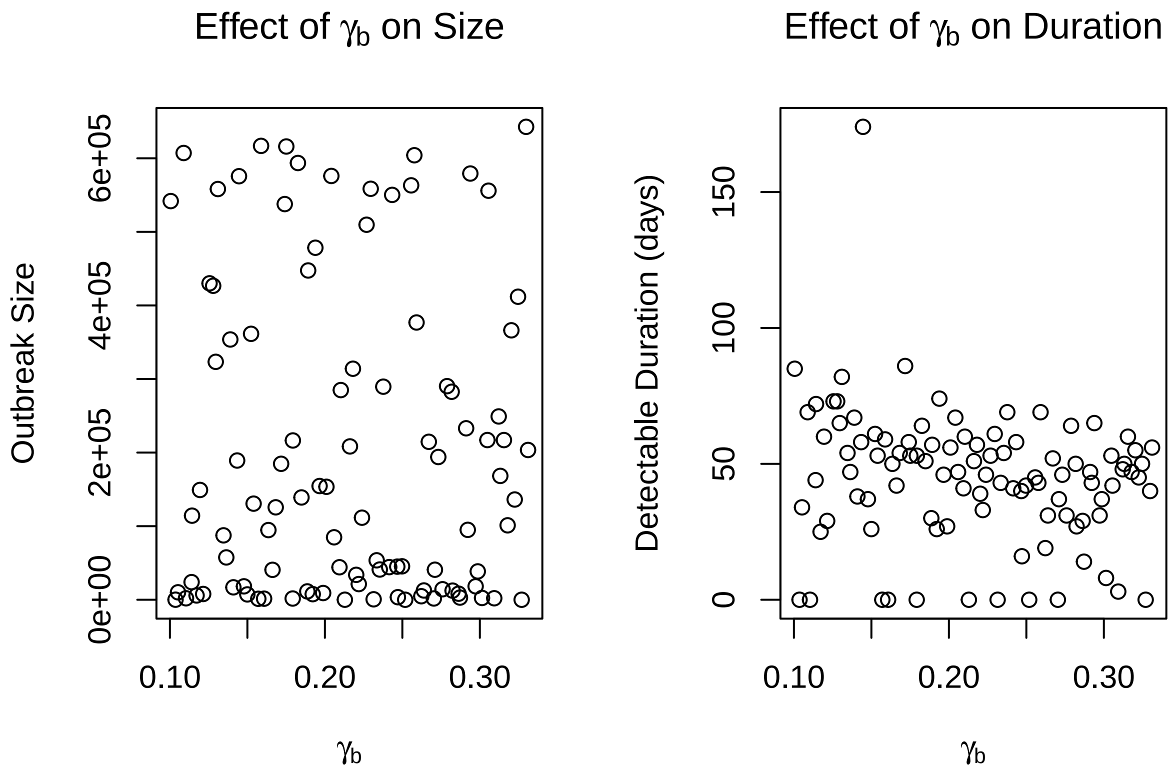


```
plot(bSEIR$MaxInf ~ bSEIR$g_h, main = expression(paste("Effect of ", g[h], " on Size")),
    xlab = expression(g[h]), ylab = "Outbreak Size")
plot(bSEIR$Thresh100 ~ bSEIR$g_h, main = expression(paste("Effect of ", g[h], " on Duration")),
    xlab = expression(g[h]), ylab = "Detectable Duration (days)")
```

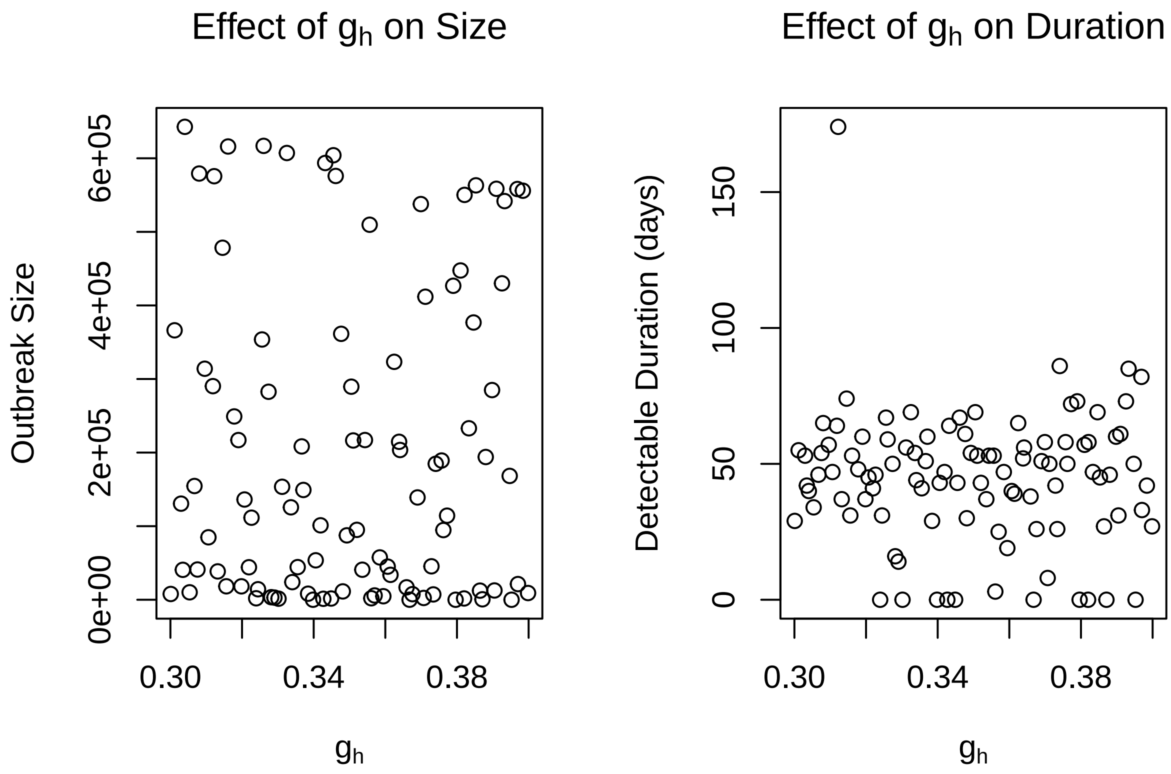


```
plot(bSEIR$MaxInf ~ bSEIR$b_h, main = expression(paste("Effect of ", b[h], " on Size")),
    xlab = expression(b[h]), ylab = "Outbreak Size")
plot(bSEIR$Thresh100 ~ bSEIR$b_h, main = expression(paste("Effect of ", b[h], " on Duration")),
    xlab = expression(b[h]), ylab = "Detectable Duration (days)")
```

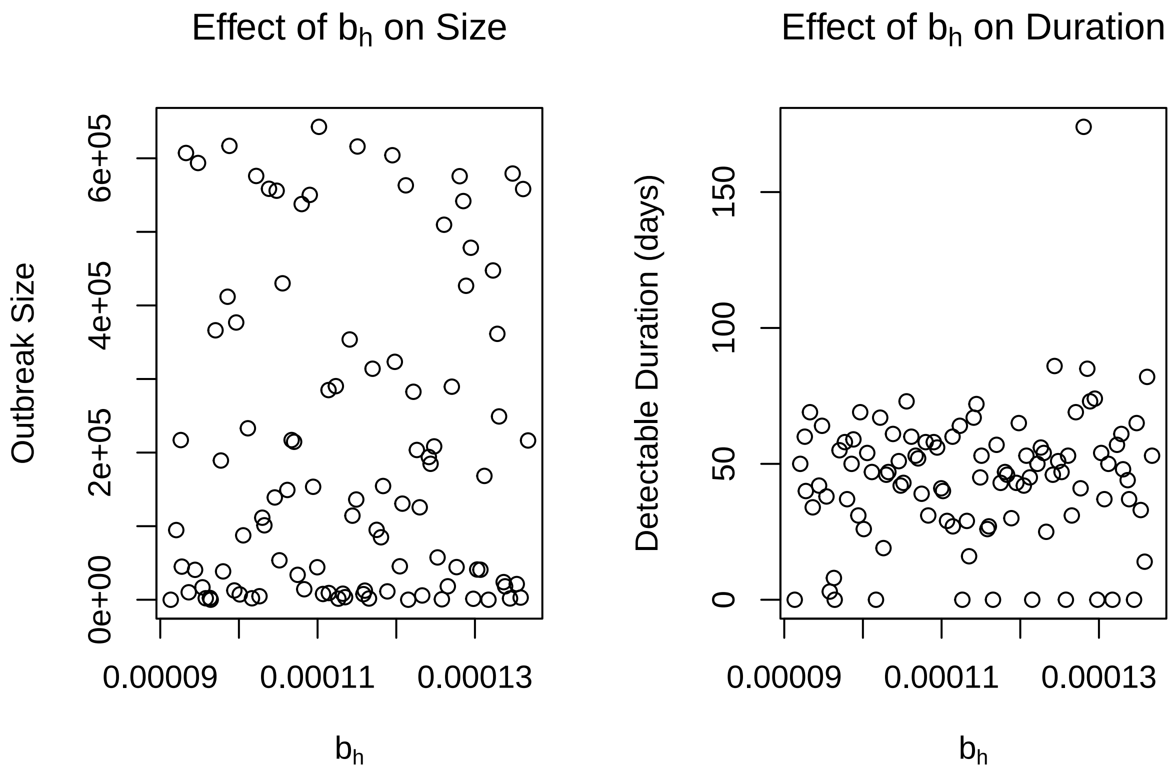


```
plot(bSEIR$MaxInf ~ bSEIR$d_h, main = expression(paste("Effect of ", d[h], " on Size")),
    xlab = expression(d[h]), ylab = "Outbreak Size")
plot(bSEIR$Thresh100 ~ bSEIR$d_h, main = expression(paste("Effect of ", d[h], " on Duration")),
    xlab = expression(d[h]), ylab = "Detectable Duration (days)")
```

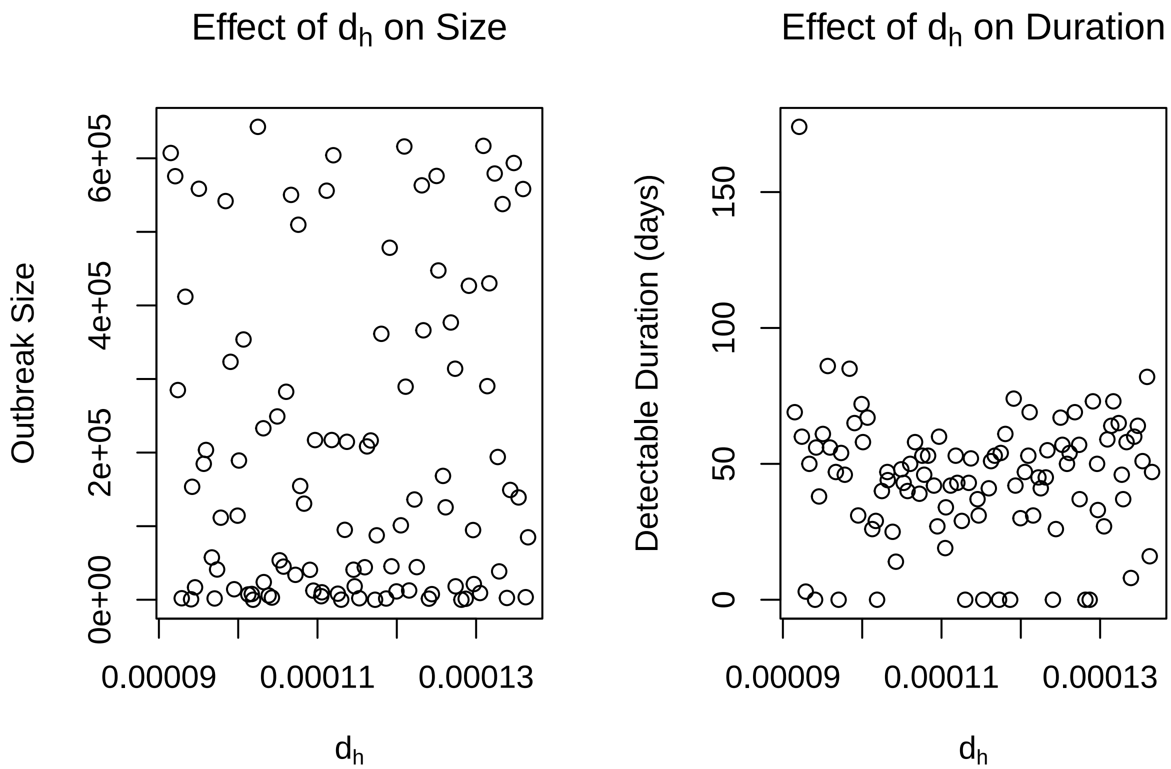


```
plot(bSEIR$MaxInf ~ bSEIR$sigma_h, main = expression(paste("Effect of ", sigma[b],
    " on Size")), xlab = expression(sigma[b]), ylab = "Outbreak Size")
plot(bSEIR$Thresh100 ~ bSEIR$sigma_h, main = expression(paste("Effect of ", sigma[b],
    " on Duration")), xlab = expression(sigma[b]), ylab = "Detectable Duration (days)")
```

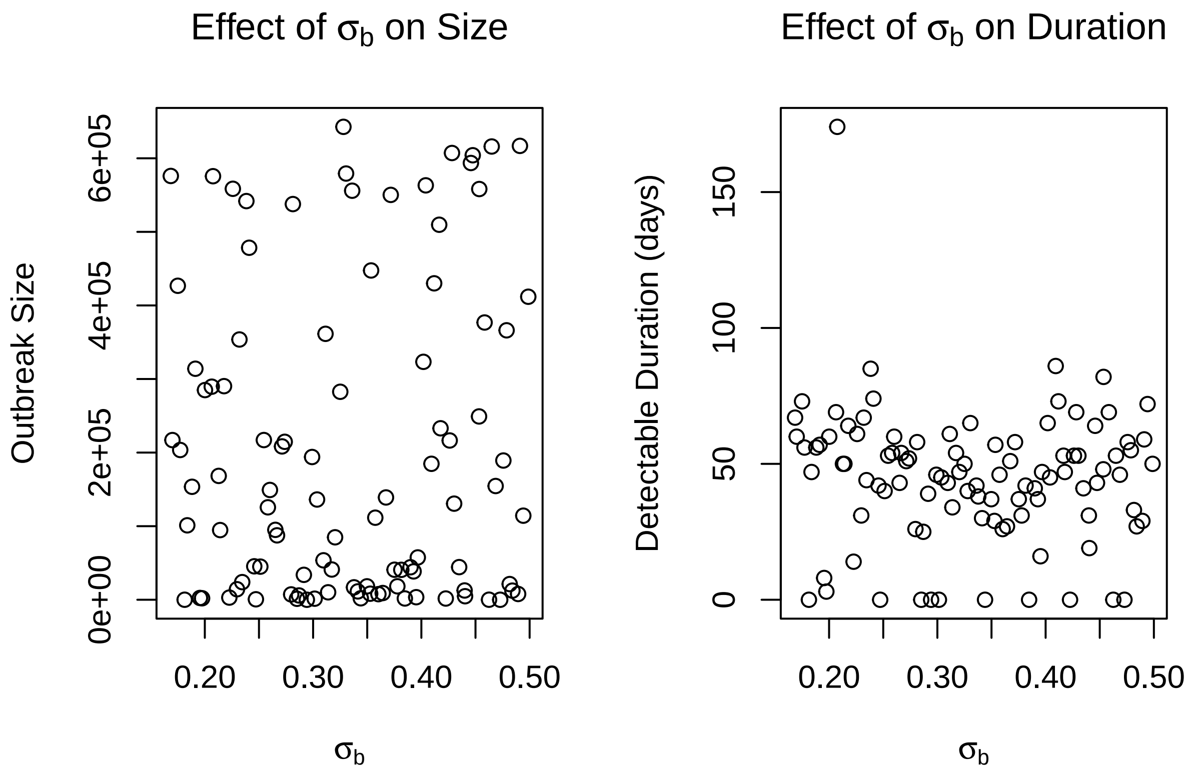


```
par(mfrow = c(1, 2))
boxplot(bSEIR$MaxInf, main = "Outbreak Size", ylab = "Number of Dead Humans", ylim = c(0,
    923406))
boxplot(bSEIR$Thresh100, main = "Outbreak Duration", ylab = "Time (Days)")
```

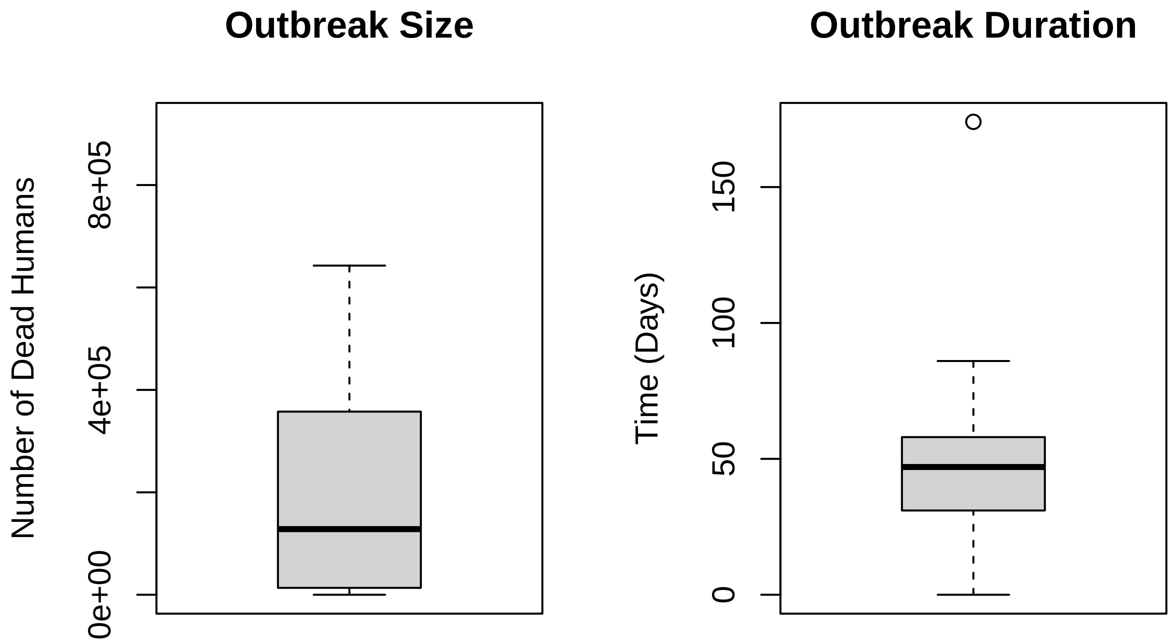


```
bonferroni.alpha <- 0.05/length(parameters)
prcc_size <- pcc(bSEIR[, 1:length(parameters)], bSEIR$MaxInf, nboot = niter, rank = TRUE,
    conf = 1 - bonferroni.alpha)
prcc_duration <- pcc(bSEIR[, 1:length(parameters)], bSEIR$Thresh100, nboot = niter,
    rank = TRUE, conf = 1 - bonferroni.alpha)

# plot correlation coefficients and confidence intervals for epidemic size and
# duration
size <- prcc_size$PRCC
size$param <- rownames(size)
colnames(size)[4:5] <- c("maxCI", "minCI")
size$maxCI[which(size$maxCI > 1)] <- 1
size$maxCI[which(size$maxCI < -1)] <- -1
size$minCI[which(size$minCI > 1)] <- 1
size$minCI[which(size$minCI < -1)] <- -1

duration <- prcc_duration$PRCC
duration$param <- rownames(duration)
colnames(duration)[4:5] <- c("maxCI", "minCI")
duration$maxCI[which(duration$maxCI > 1)] <- 1
duration$maxCI[which(duration$maxCI < -1)] <- -1
duration$minCI[which(duration$minCI > 1)] <- 1
duration$minCI[which(duration$minCI < -1)] <- -1

A <- ggplot(size, aes(x = param, y = original)) + geom_point(size = 4) + geom_errorbar(aes(ymax = maxCI
    ymin = minCI)) + ggtitle("A") + xlab("Parameters") + ylab("Partial Rank Correlation Coefficients") +
```

```
    scale_x_discrete(labels = c(alpha = expression(alpha), beta_h = expression(beta[b]),
        beta_r = expression(beta[r]), b_h = expression(b[h]), d_h = expression(d[h]),
        d_f = expression(d[f]), sigma_h = expression(sigma[b]), gamma_h = expression(gamma[b]),
        gamma_r = expression(gamma[r]), g_h = expression(g[h]), g_r = expression(g[r]),
        K_f = expression(K[f]), r_f = expression(r[f]))) + ylim(-1, 1)

B <- ggplot(duration, aes(x = param, y = original)) + geom_point(size = 4) + geom_errorbar(aes(ymax = ma
    ymin = minCI)) + ggtitle("B") + xlab("Parameters") + ylab(" ") + scale_x_discrete(labels = c(alpha =
    beta_h = expression(beta[b]), beta_r = expression(beta[r]), b_h = expression(b[h]),
    d_h = expression(d[h]), d_f = expression(d[f]), sigma_h = expression(sigma[b]),
    gamma_h = expression(gamma[b]), gamma_r = expression(gamma[r]), g_h = expression(g[h]),
    g_r = expression(g[r]), K_f = expression(K[f]), r_f = expression(r[f]))) + ylim(-1,
    1)

multiplot(A, B, cols = 2)
```

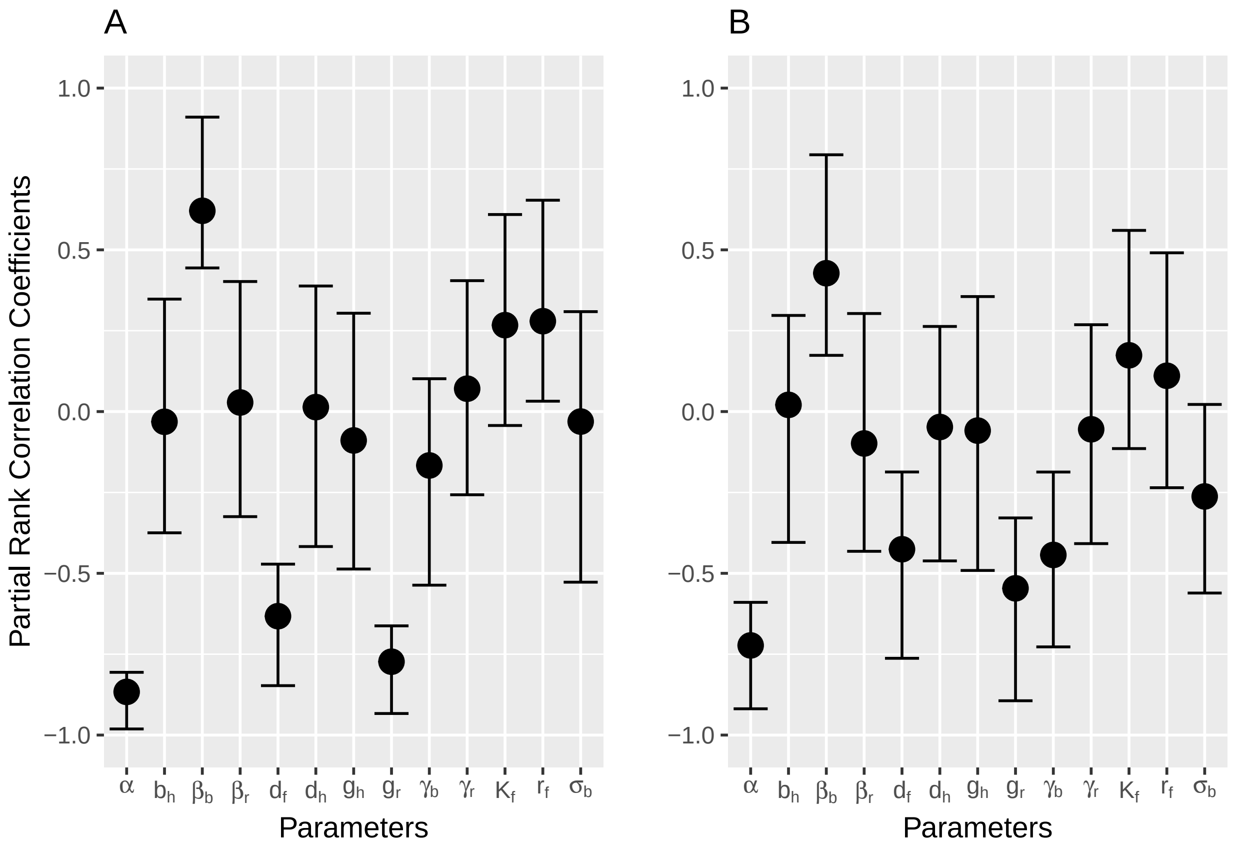


```
# tiff('FigureS14BubonicSEIR_PRCCuniform.tiff', height =8.7 , width =10, units
# = 'cm', compression = 'lzw', res = 1200) multiplot(A, B, cols=2) dev.off()
```

Bubonic SIR with rat carrying capacity and resistance

```
# parameters <- c(r_r=0.014, K_r=499999, p_r=0.975, d_r=0.00055, beta_r = 0.09,
# alpha=3/500000, gamma_r = 1/5.15, g_r=0.1, r_f=0.0084, K_f=6, d_f=1/5,
```

```r
# beta_h=0.19, gamma_h=1/10, g_h=0.34, b_h=1/(25*365), d_h=1/(25*365)) #you can
# play with transmission and recovery rates here
parameters <- c(r_r = 0.014, K_r = 923405, p_r = 0.975, d_r = 0.00055, beta_r = 0.09,
    alpha = 3/923406, gamma_r = 1/5.15, g_r = 0.1, r_f = 0.0084, K_f = 6, d_f = 1/5,
    beta_h = 0.19, gamma_h = 1/10, g_h = 0.34, b_h = 1/(25 * 365), d_h = 1/(25 *
        365))  #you can play with transmission and recovery rates here

par(mfrow = c(1, 2))
plot(bSIRrK$MaxInf ~ bSIRrK$r_r, main = expression(paste("Effect of ", r[r], " on Size")),
    xlab = expression(r[r]), ylab = "Outbreak Size")
plot(bSIRrK$Thresh100 ~ bSIRrK$r_r, main = expression(paste("Effect of ", r[r], " on Duration")),
    xlab = expression(r[r]), ylab = "Detectable Duration (days)")
```

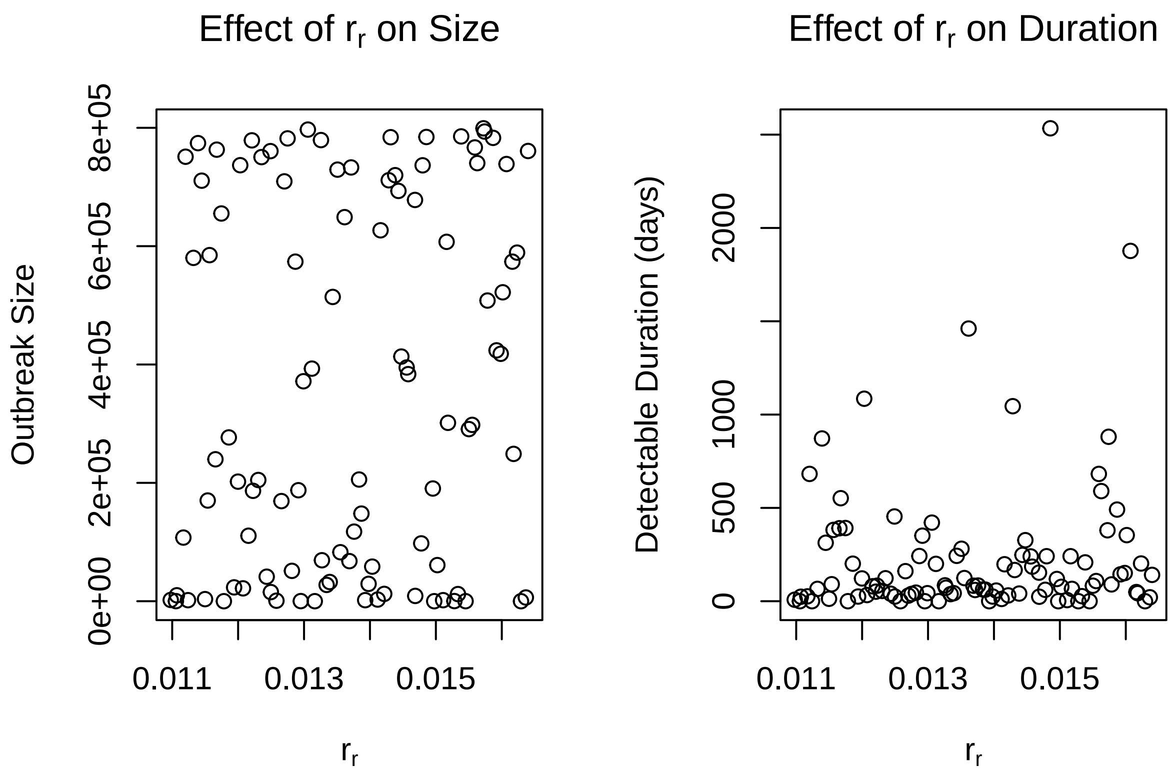


```r
plot(bSIRrK$MaxInf ~ bSIRrK$K_r, main = expression(paste("Effect of ", K[r], " on Size")),
    xlab = expression(K[r]), ylab = "Outbreak Size")
plot(bSIRrK$Thresh100 ~ bSIRrK$K_r, main = expression(paste("Effect of ", K[r], " on Duration")),
    xlab = expression(K[r]), ylab = "Detectable Duration (days)")
```

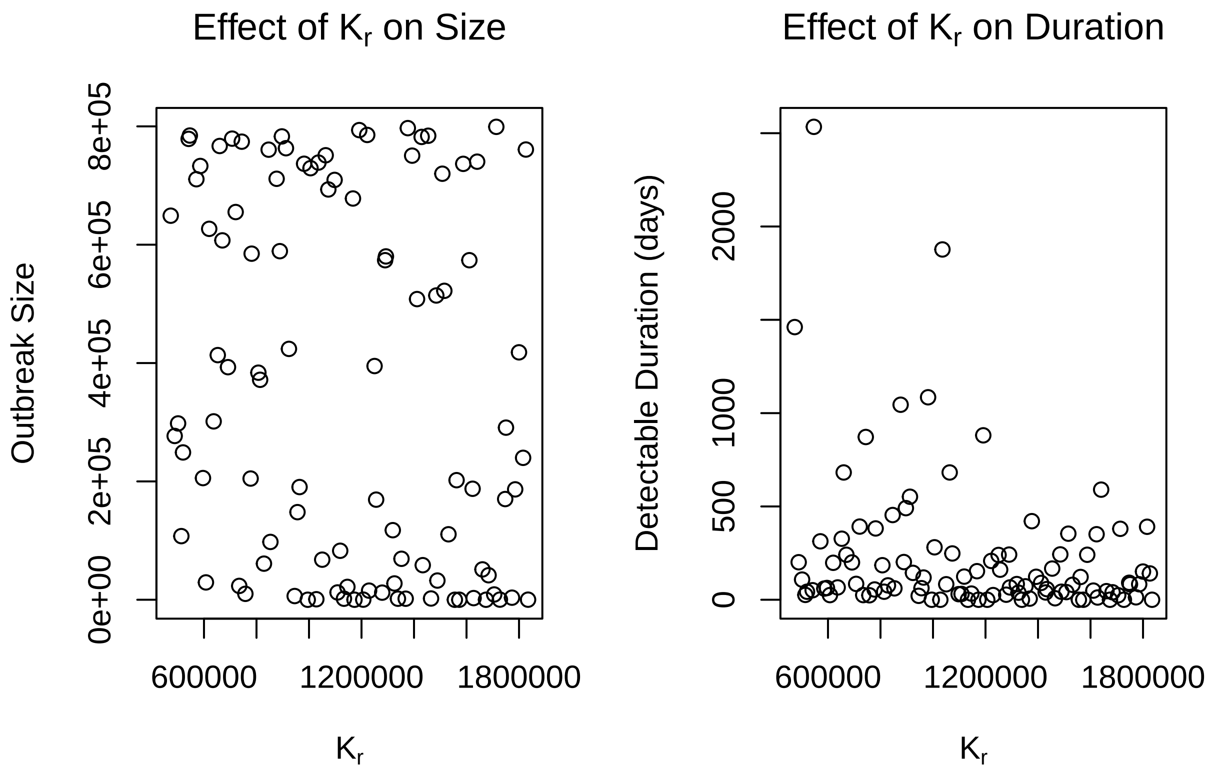


```
plot(bSIRrK$MaxInf ~ bSIRrK$p_r, main = expression(paste("Effect of ", p[r], " on Size")),
    xlab = expression(p[r]), ylab = "Outbreak Size")
plot(bSIRrK$Thresh100 ~ bSIRrK$p_r, main = expression(paste("Effect of ", p[r], " on Duration")),
    xlab = expression(p[r]), ylab = "Detectable Duration (days)")
```

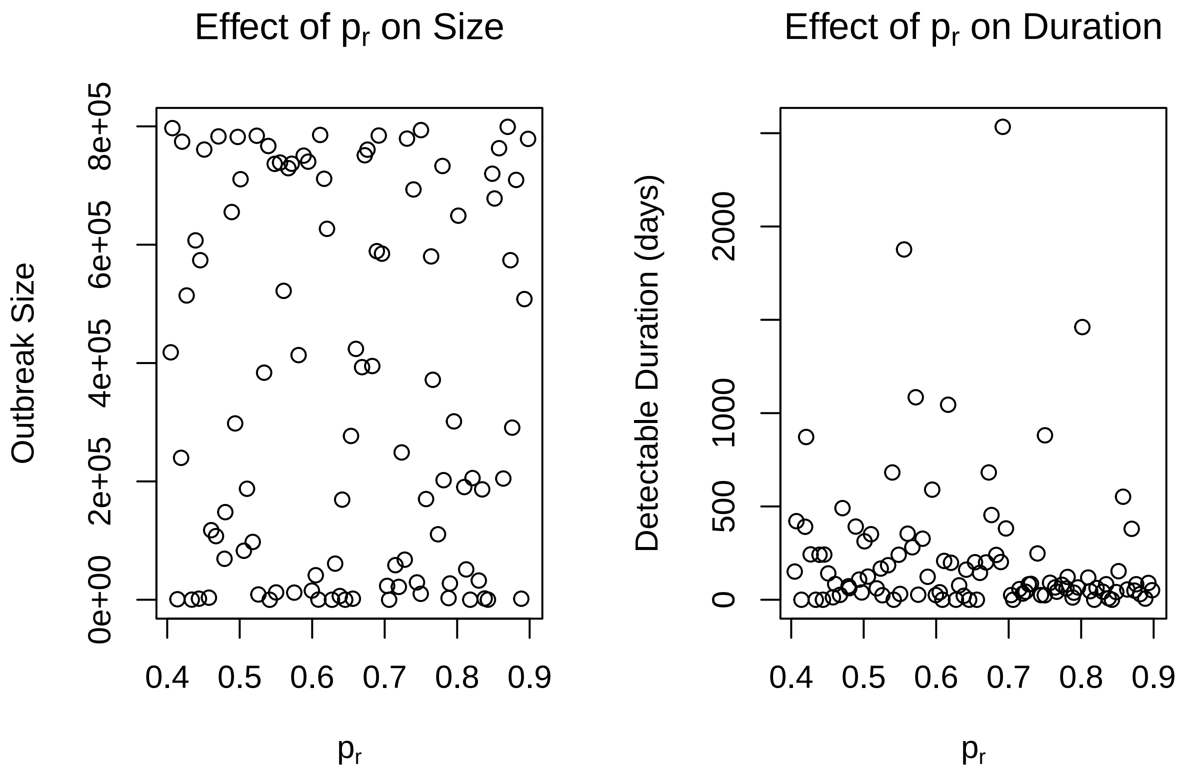


```
plot(bSIRrK$MaxInf ~ bSIRrK$d_r, main = expression(paste("Effect of ", d[r], " on Size")),
    xlab = expression(d[r]), ylab = "Outbreak Size")
plot(bSIRrK$Thresh100 ~ bSIRrK$d_r, main = expression(paste("Effect of ", d[r], " on Duration")),
    xlab = expression(d[r]), ylab = "Detectable Duration (days)")
```

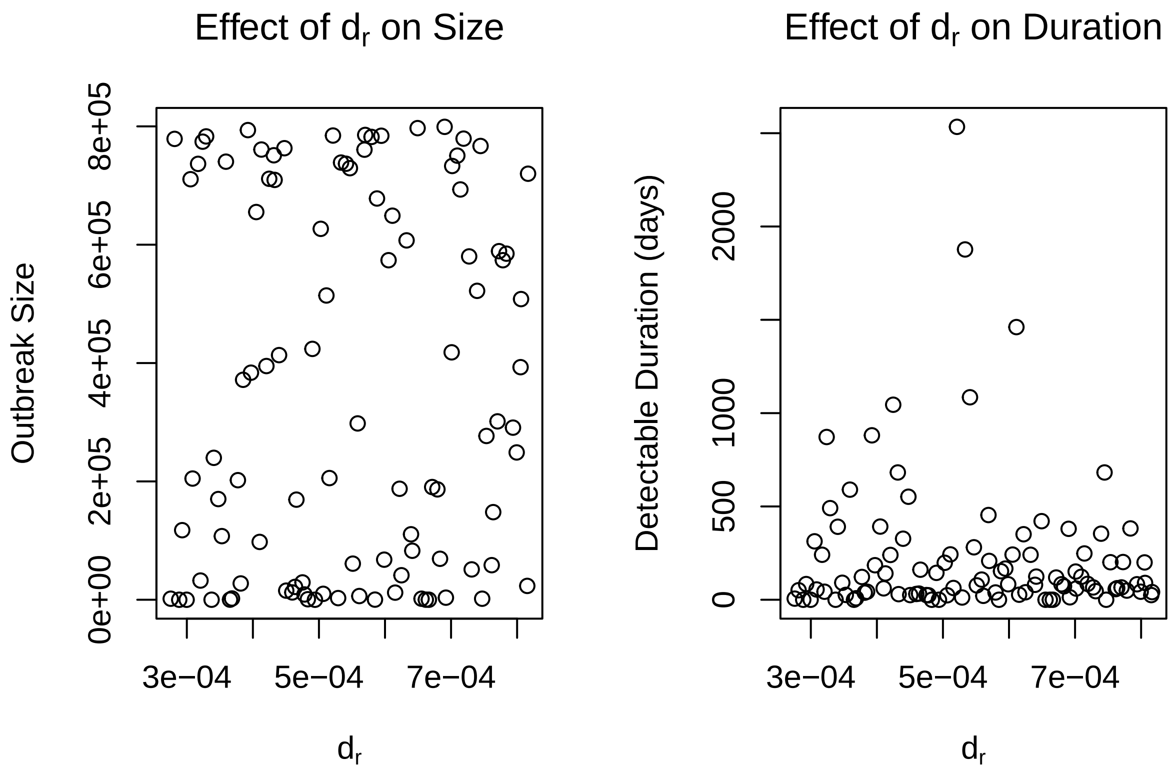


```
plot(bSIRrK$MaxInf ~ bSIRrK$beta_r, main = expression(paste("Effect of ", beta[r],
    " on Size")), xlab = expression(beta[r]), ylab = "Outbreak Size")
plot(bSIRrK$Thresh100 ~ bSIRrK$beta_r, main = expression(paste("Effect of ", beta[r],
    " on Duration")), xlab = expression(beta[r]), ylab = "Detectable Duration (days)")
```

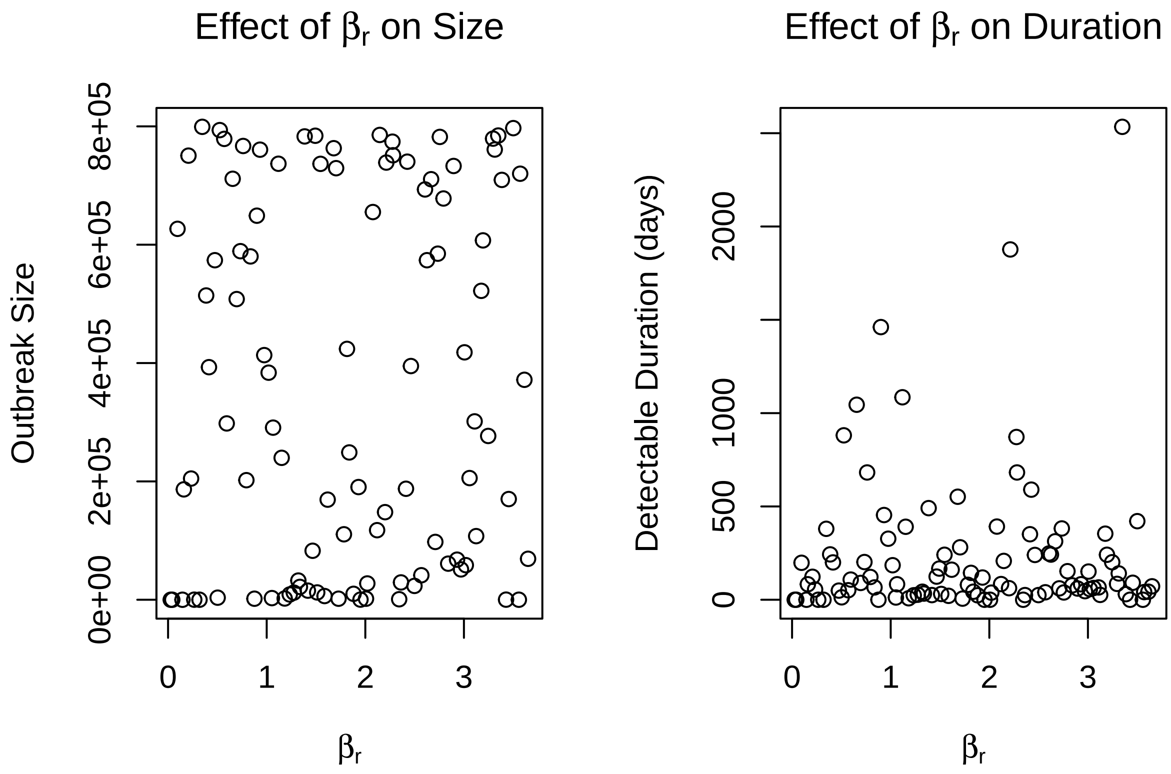


```
plot(bSIRrK$MaxInf ~ bSIRrK$alpha, main = expression(paste("Effect of ", alpha, " on Size")),
    xlab = expression(alpha), ylab = "Outbreak Size")
plot(bSIRrK$Thresh100 ~ bSIRrK$alpha, main = expression(paste("Effect of ", alpha,
    " on Duration")), xlab = expression(alpha), ylab = "Detectable Duration (days)")
```

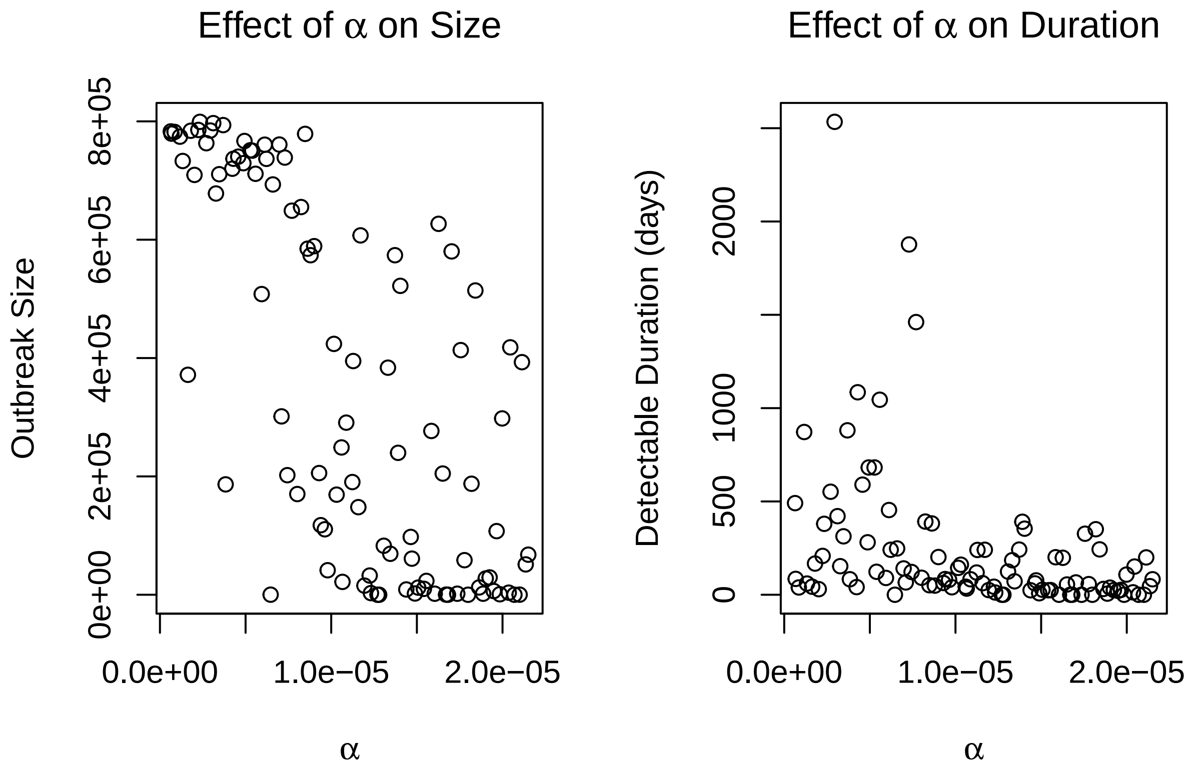


```
plot(bSIRrK$MaxInf ~ bSIRrK$gamma_r, main = expression(paste("Effect of ", gamma[r],
    " on Size")), xlab = expression(gamma[r]), ylab = "Outbreak Size")
plot(bSIRrK$Thresh100 ~ bSIRrK$gamma_r, main = expression(paste("Effect of ", gamma[r],
    " on Duration")), xlab = expression(gamma[r]), ylab = "Detectable Duration (days)")
```

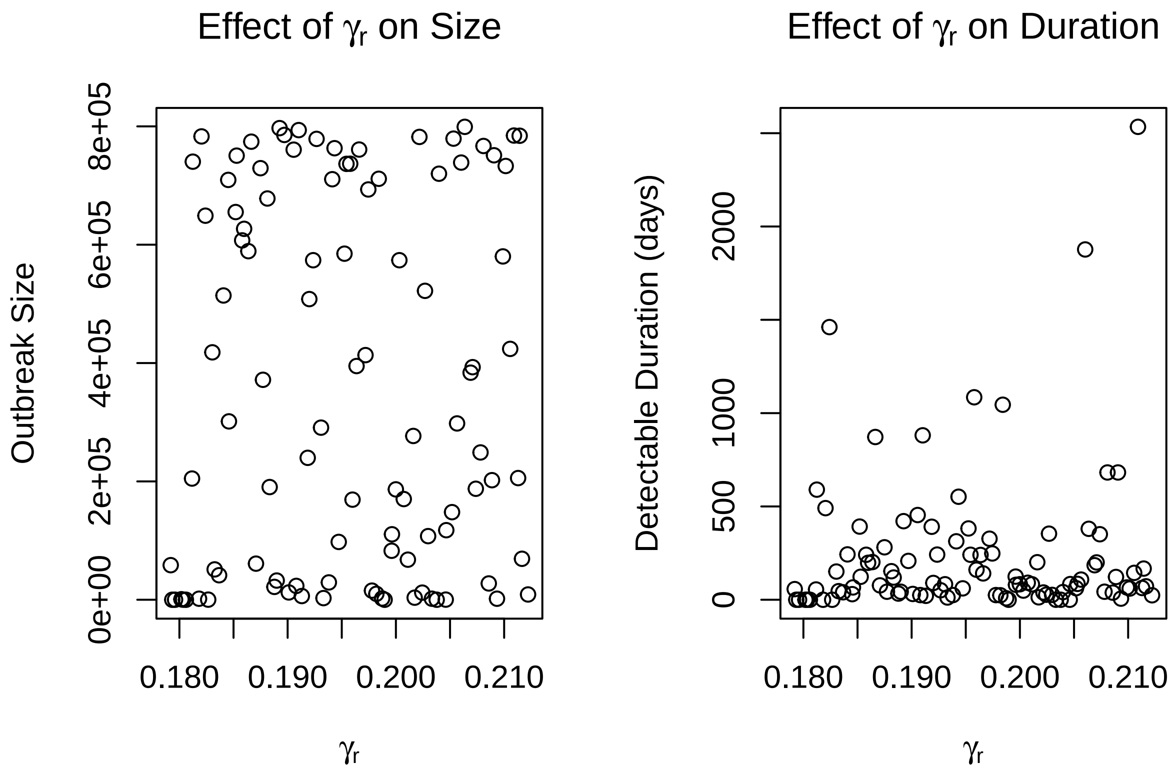


```
plot(bSIRrK$MaxInf ~ bSIRrK$g_r, main = expression(paste("Effect of ", g[r], " on Size")),
    xlab = expression(g[r]), ylab = "Outbreak Size")
plot(bSIRrK$Thresh100 ~ bSIRrK$g_r, main = expression(paste("Effect of ", g[r], " on Duration")),
    xlab = expression(g[r]), ylab = "Detectable Duration (days)")
```

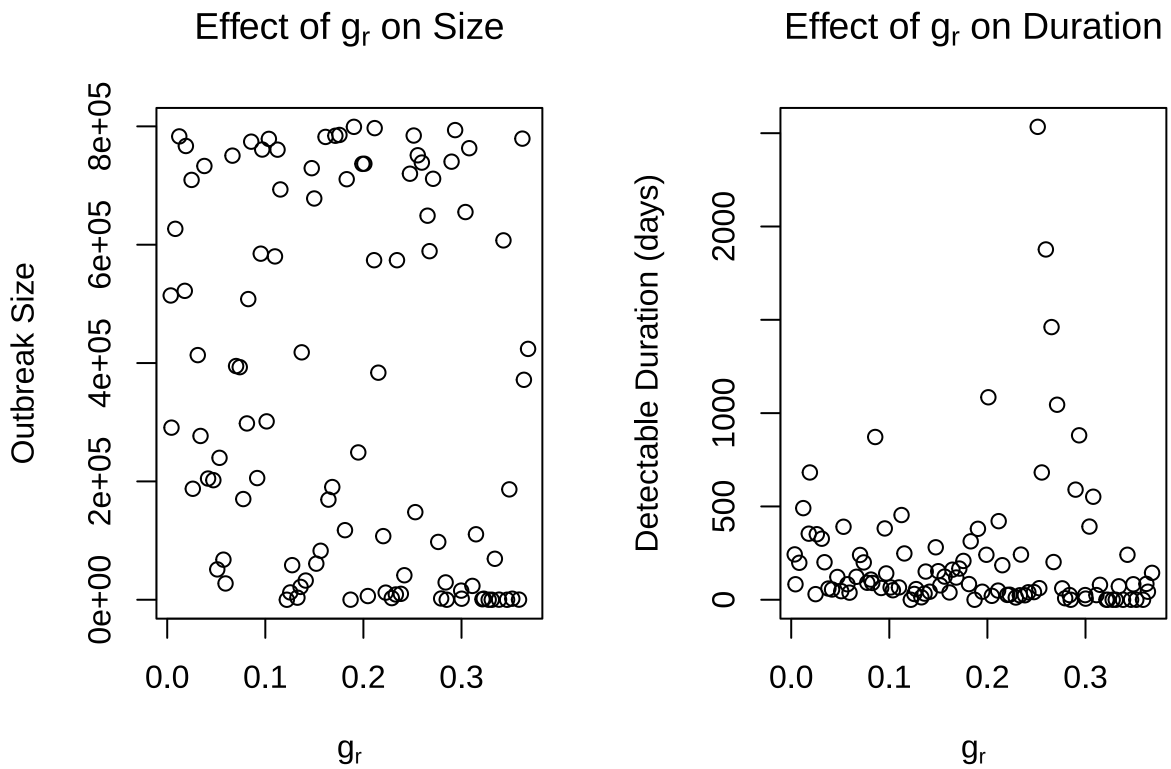


```
plot(bSIRrK$MaxInf ~ bSIRrK$r_f, main = expression(paste("Effect of ", r[f], " on Size")),
    xlab = expression(r[f]), ylab = "Outbreak Size")
plot(bSIRrK$Thresh100 ~ bSIRrK$r_f, main = expression(paste("Effect of ", r[f], " on Duration")),
    xlab = expression(r[f]), ylab = "Detectable Duration (days)")
```

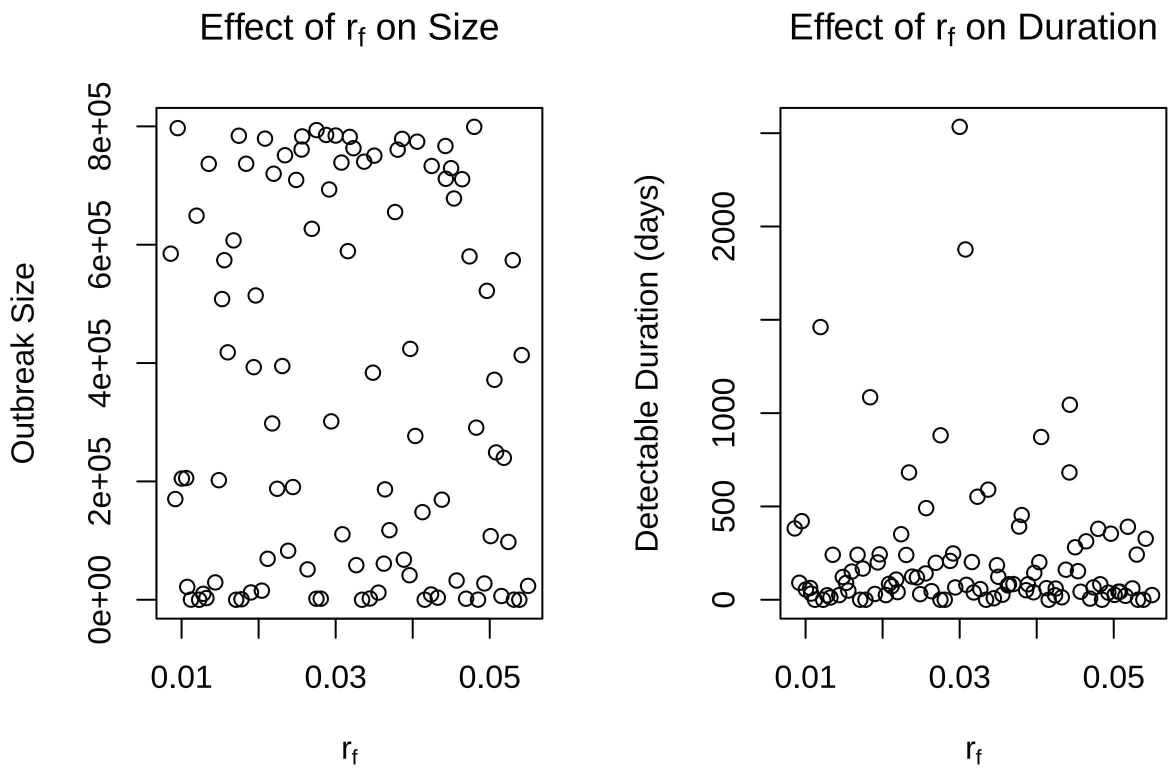


```
plot(bSIRrK$MaxInf ~ bSIRrK$K_f, main = expression(paste("Effect of ", K[f], " on Size")),
    xlab = expression(K[f]), ylab = "Outbreak Size")
plot(bSIRrK$Thresh100 ~ bSIRrK$K_f, main = expression(paste("Effect of ", K[f], " on Duration")),
    xlab = expression(K[f]), ylab = "Detectable Duration (days)")
```

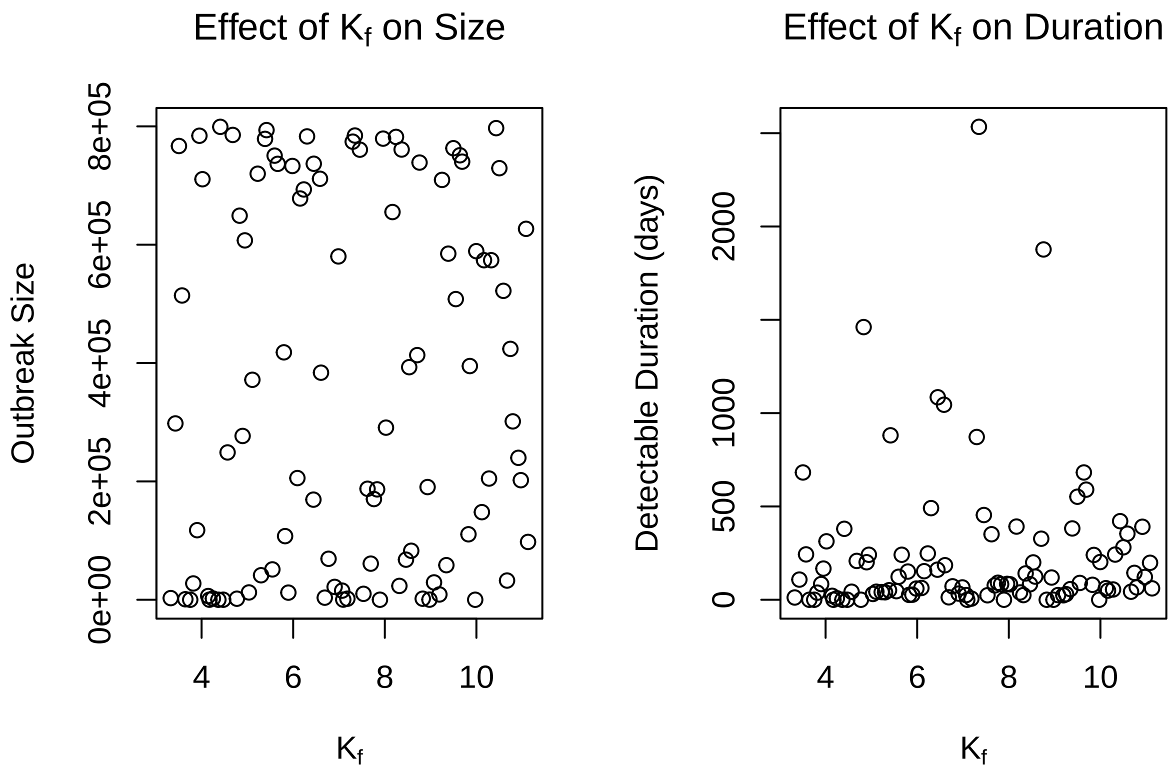


```
plot(bSIRrK$MaxInf ~ bSIRrK$d_f, main = expression(paste("Effect of ", d[f], " on Size")),
    xlab = expression(d[f]), ylab = "Outbreak Size")
plot(bSIRrK$Thresh100 ~ bSIRrK$d_f, main = expression(paste("Effect of ", d[f], " on Duration")),
    xlab = expression(d[f]), ylab = "Detectable Duration (days)")
```

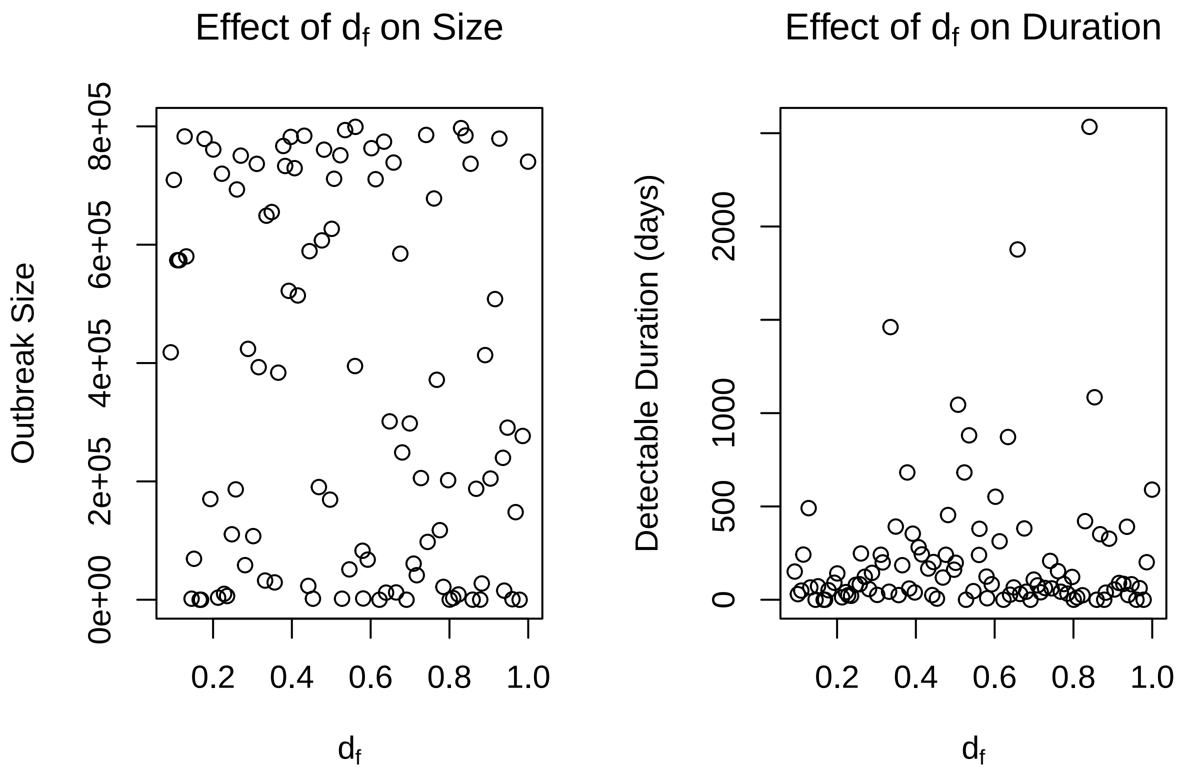


```
plot(bSIRrK$MaxInf ~ bSIRrK$beta_h, main = expression(paste("Effect of ", beta[b],
    " on Size")), xlab = expression(beta[b]), ylab = "Outbreak Size")
plot(bSIRrK$Thresh100 ~ bSIRrK$beta_h, main = expression(paste("Effect of ", beta[b],
    " on Duration")), xlab = expression(beta[b]), ylab = "Detectable Duration (days)")
```

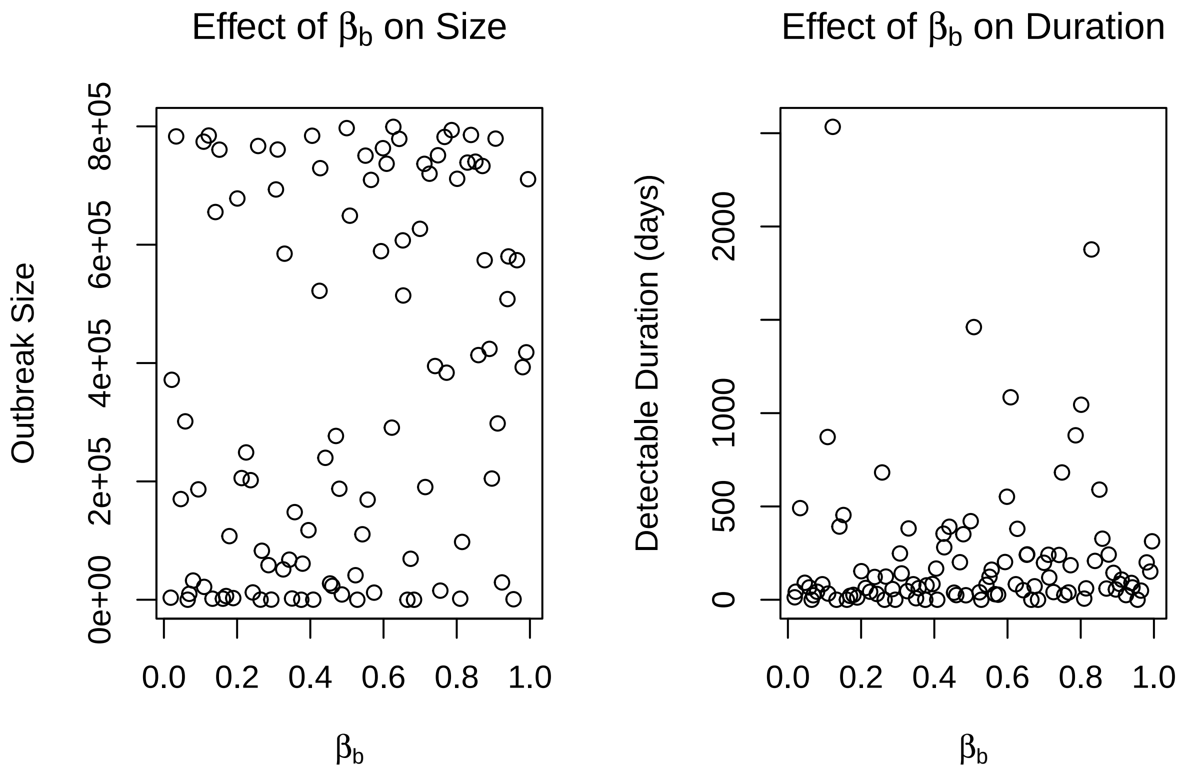


```
plot(bSIRrK$MaxInf ~ bSIRrK$gamma_h, main = expression(paste("Effect of ", gamma[b],
    " on Size")), xlab = expression(gamma[b]), ylab = "Outbreak Size")
plot(bSIRrK$Thresh100 ~ bSIRrK$gamma_h, main = expression(paste("Effect of ", gamma[b],
    " on Duration")), xlab = expression(gamma[b]), ylab = "Detectable Duration (days)")
```

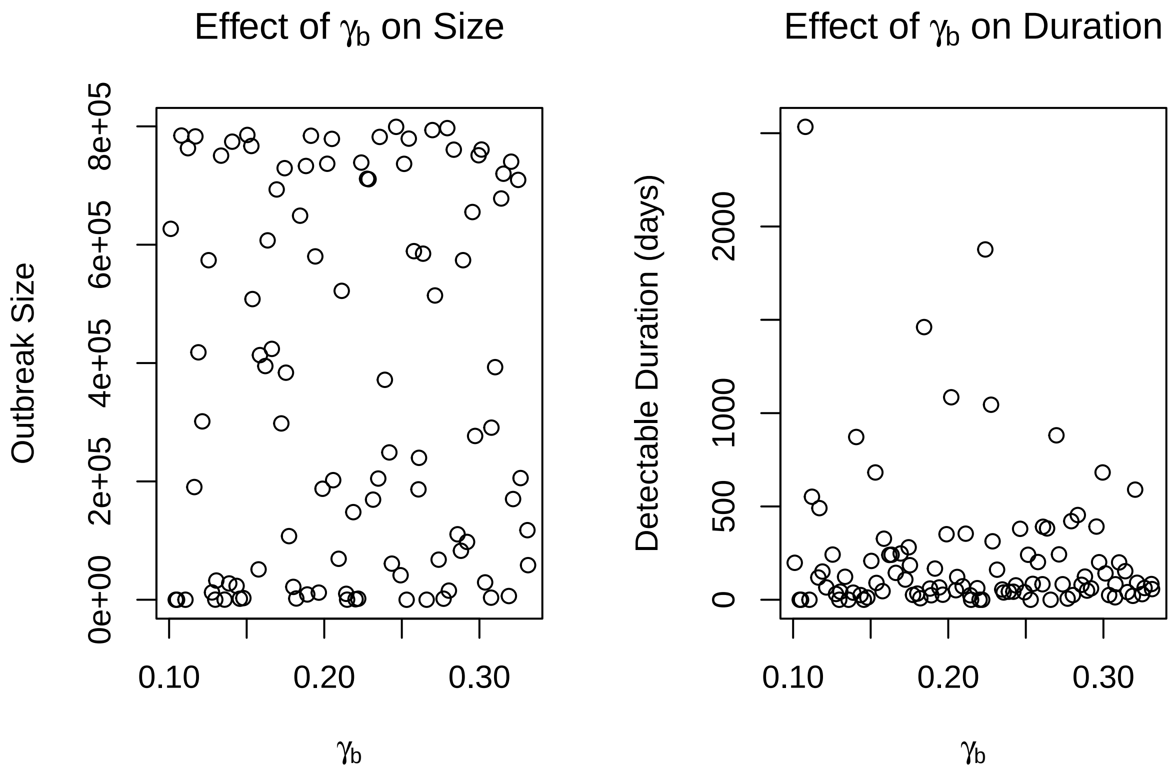


```
plot(bSIRrK$MaxInf ~ bSIRrK$g_h, main = expression(paste("Effect of ", g[h], " on Size")),
    xlab = expression(g[h]), ylab = "Outbreak Size")
plot(bSIRrK$Thresh100 ~ bSIRrK$g_h, main = expression(paste("Effect of ", g[h], " on Duration")),
    xlab = expression(g[h]), ylab = "Detectable Duration (days)")
```

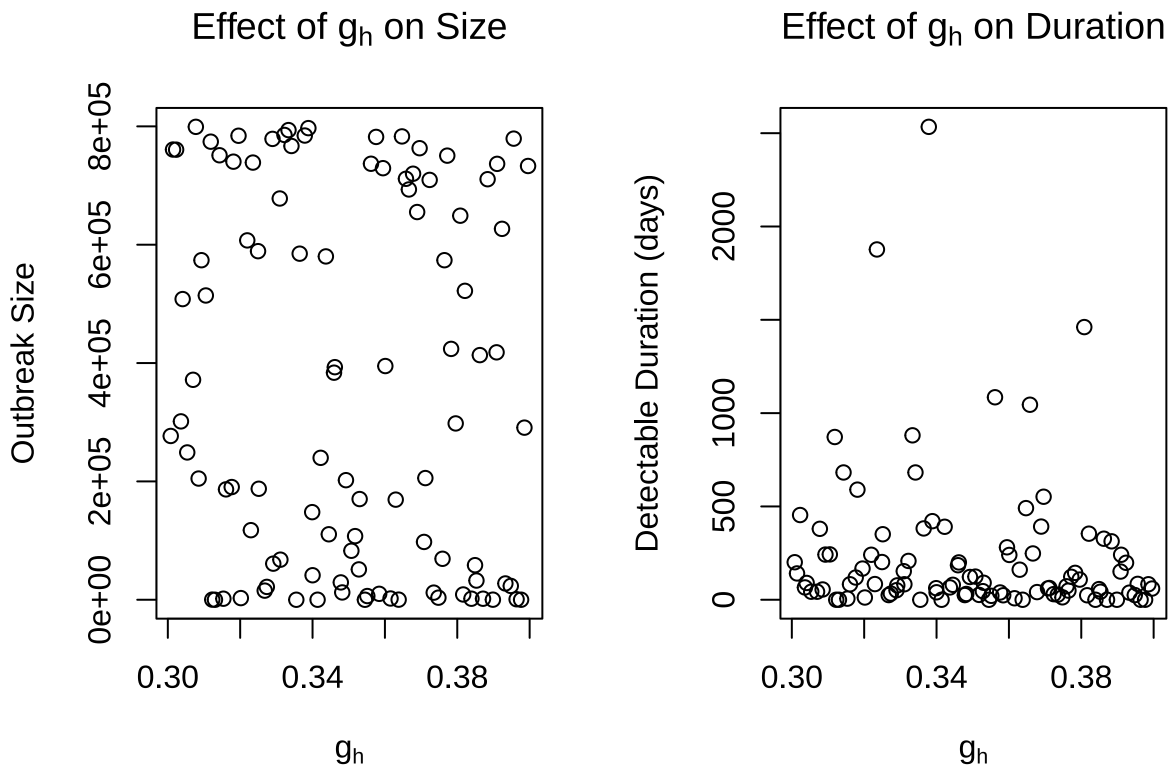


```
plot(bSIRrK$MaxInf ~ bSIRrK$b_h, main = expression(paste("Effect of ", b[h], " on Size")),
    xlab = expression(b[h]), ylab = "Outbreak Size")
plot(bSIRrK$Thresh100 ~ bSIRrK$b_h, main = expression(paste("Effect of ", b[h], " on Duration")),
    xlab = expression(b[h]), ylab = "Detectable Duration (days)")
```

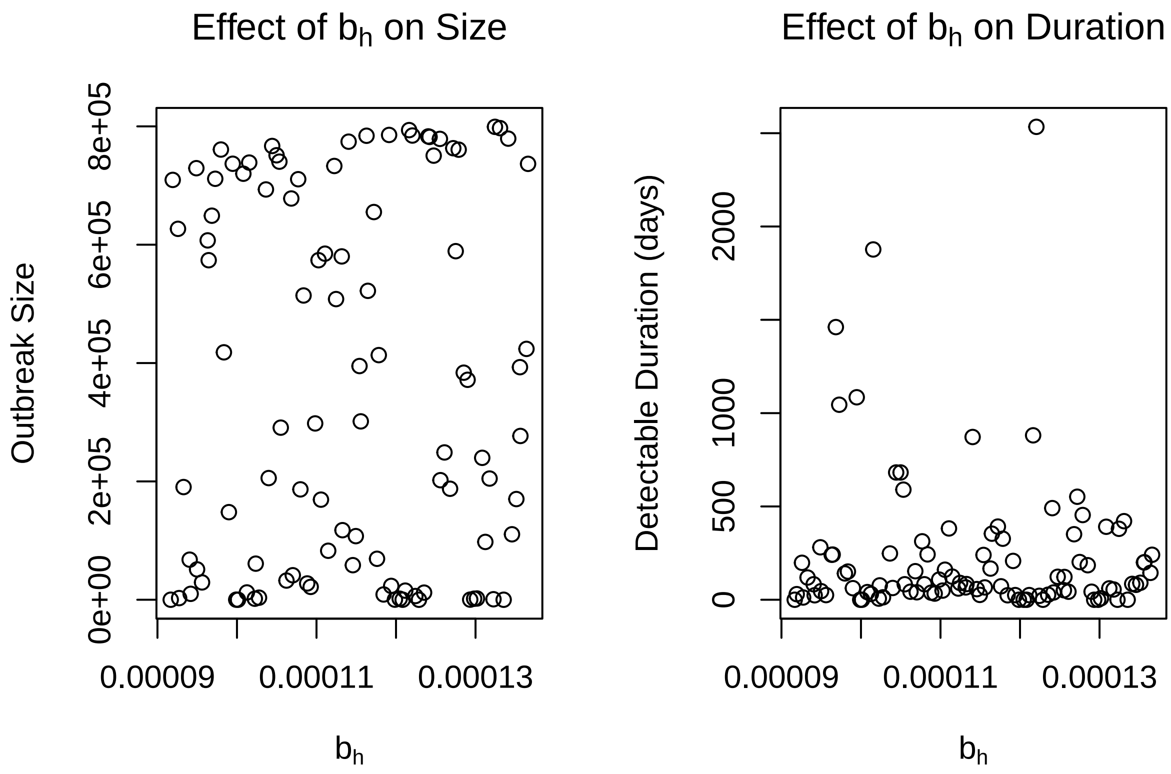


```
plot(bSIRrK$MaxInf ~ bSIRrK$d_h, main = expression(paste("Effect of ", d[h], " on Size")),
    xlab = expression(d[h]), ylab = "Outbreak Size")
plot(bSIRrK$Thresh100 ~ bSIRrK$d_h, main = expression(paste("Effect of ", d[h], " on Duration")),
    xlab = expression(d[h]), ylab = "Detectable Duration (days)")
```

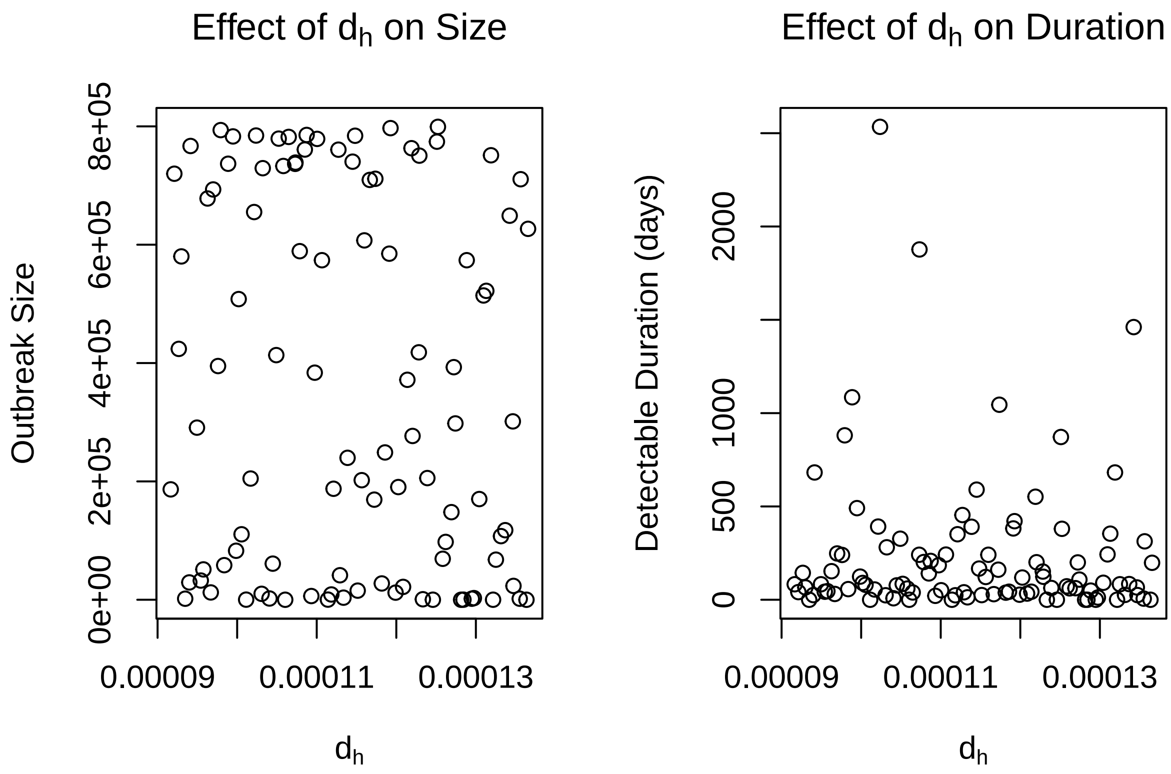


```
par(mfrow = c(1, 2))
boxplot(bSIRrK$MaxInf, main = "Outbreak Size", ylab = "Number of Dead Humans", ylim = c(0,
    923406))
boxplot(bSIRrK$Thresh100, main = "Outbreak Duration", ylab = "Time (Days)")
```

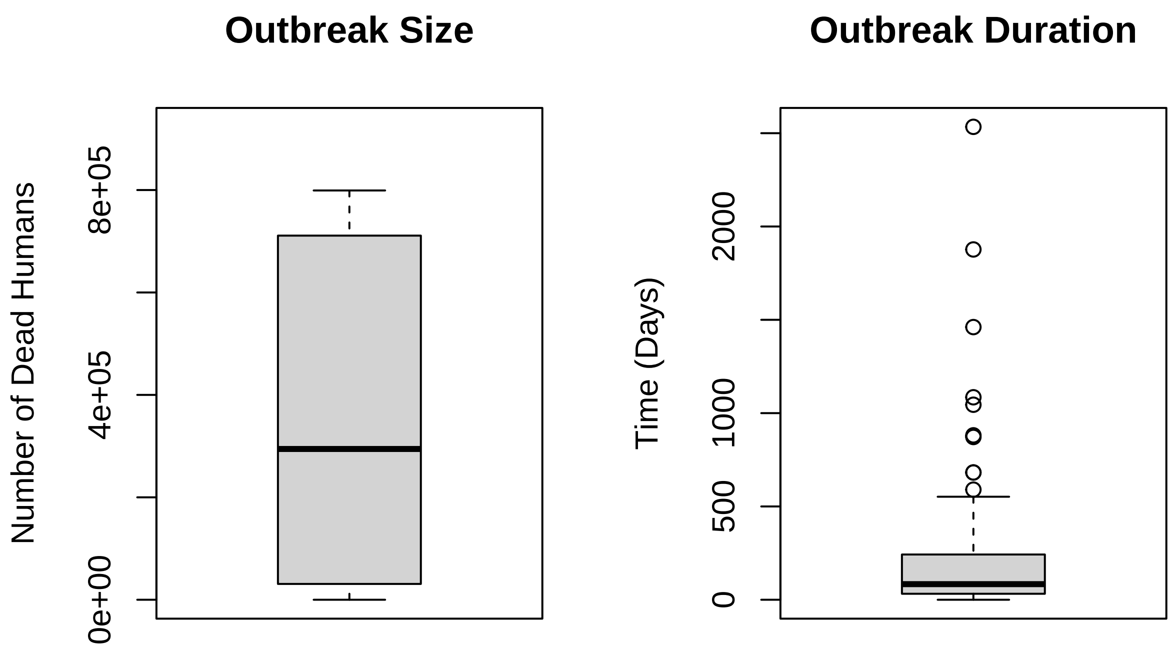


```
bonferroni.alpha <- 0.05/length(parameters)
prcc_size <- pcc(bSIRrK[, 1:length(parameters)], bSIRrK$MaxInf, nboot = niter, rank = TRUE,
    conf = 1 - bonferroni.alpha)
prcc_duration <- pcc(bSIRrK[, 1:length(parameters)], bSIRrK$Thresh100, nboot = niter,
    rank = TRUE, conf = 1 - bonferroni.alpha)

# plot correlation coefficients and confidence intervals for epidemic size and
# duration
size <- prcc_size$PRCC
size$param <- rownames(size)
colnames(size)[4:5] <- c("maxCI", "minCI")
size$maxCI[which(size$maxCI > 1)] <- 1
size$maxCI[which(size$maxCI < -1)] <- -1
size$minCI[which(size$minCI > 1)] <- 1
size$minCI[which(size$minCI < -1)] <- -1

duration <- prcc_duration$PRCC
duration$param <- rownames(duration)
colnames(duration)[4:5] <- c("maxCI", "minCI")
duration$maxCI[which(duration$maxCI > 1)] <- 1
duration$maxCI[which(duration$maxCI < -1)] <- -1
duration$minCI[which(duration$minCI > 1)] <- 1
duration$minCI[which(duration$minCI < -1)] <- -1

A <- ggplot(size, aes(x = param, y = original)) + geom_point(size = 4) + geom_errorbar(aes(ymax = maxCI
    ymin = minCI)) + ggtitle("A") + xlab("Parameters") + ylab("Partial Rank Correlation Coefficients")
```

```
    scale_x_discrete(labels = c(r_r = expression(r[r]), d_r = expression(d[r]), K_r = expression(K[r]),
        p_r = expression(p[r]), alpha = expression(alpha), beta_h = expression(beta[b]),
        beta_r = expression(beta[r]), b_h = expression(b[h]), d_h = expression(d[h]),
        d_f = expression(d[f]), gamma_h = expression(gamma[b]), gamma_r = expression(gamma[r]),
        g_h = expression(g[h]), g_r = expression(g[r]), K_f = expression(K[f]), r_f = expression(r[f]))
    ylim(-1, 1)

B <- ggplot(duration, aes(x = param, y = original)) + geom_point(size = 4) + geom_errorbar(aes(ymax = ma
    ymin = minCI)) + ggtitle("B") + xlab("Parameters") + ylab(" ") + scale_x_discrete(labels = c(r_r = e
    d_r = expression(d[r]), K_r = expression(K[r]), p_r = expression(p[r]), alpha = expression(alpha),
    beta_h = expression(beta[b]), beta_r = expression(beta[r]), b_h = expression(b[h]),
    d_h = expression(d[h]), d_f = expression(d[f]), gamma_h = expression(gamma[b]),
    gamma_r = expression(gamma[r]), g_h = expression(g[h]), g_r = expression(g[r]),
    K_f = expression(K[f]), r_f = expression(r[f]))) + ylim(-1, 1)

multiplot(A, B, cols = 2)
```

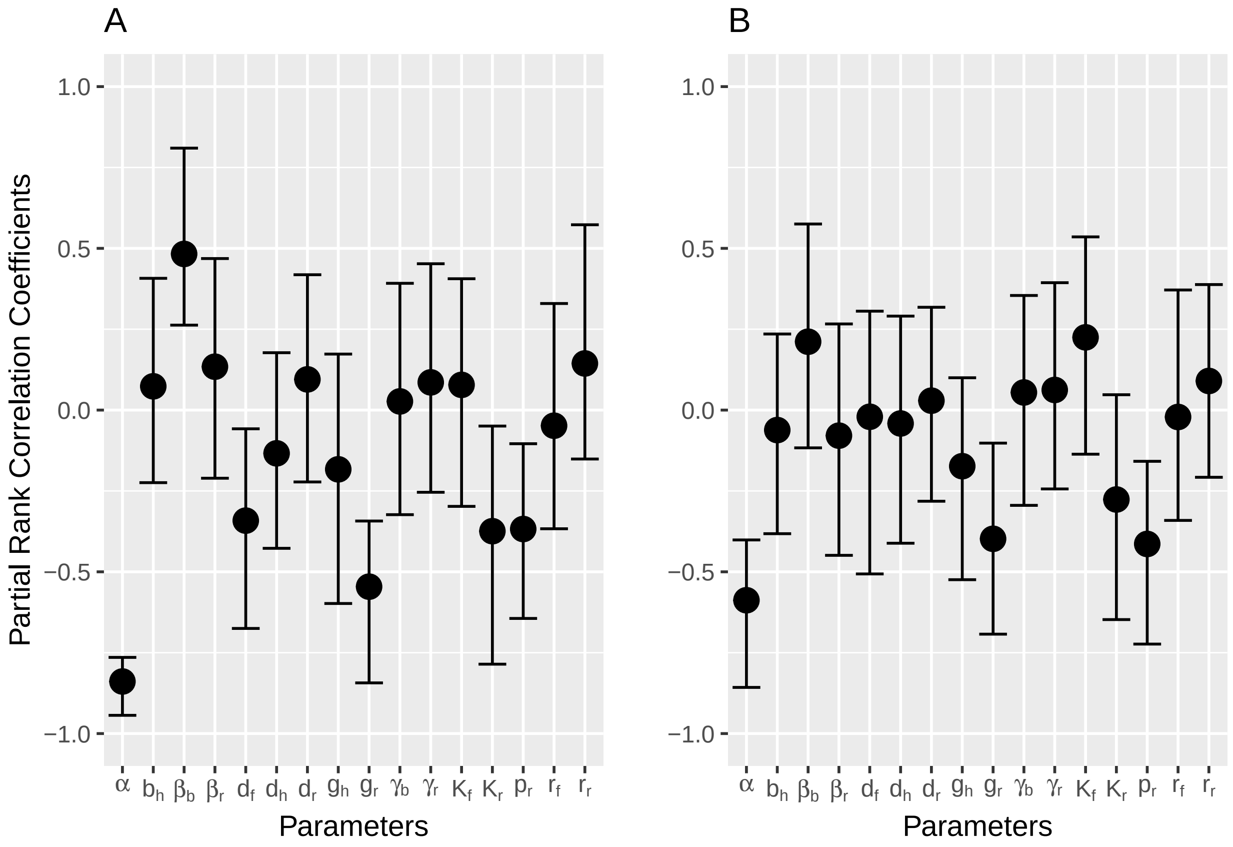


```
# tiff('FigureS15BubonicSIRrK_PRCCuniform.tiff', height =8.7 , width =10, units
# = 'cm', compression = 'lzw', res = 1200) multiplot(A, B, cols=2) dev.off()
```

Bubonic SEIR with rat carrying capacity and resistance

```r
parameters <- c(r_r = 0.014, K_r = 923405, p_r = 0.975, d_r = 0.00055, beta_r = 0.09,
    alpha = 3/923406, gamma_r = 1/5.15, g_r = 0.1, r_f = 0.0084, K_f = 6, d_f = 1/5,
    beta_h = 0.19, sigma_h = 1/4, gamma_h = 1/10, g_h = 0.34, b_h = 1/(25 * 365),
    d_h = 1/(25 * 365))  #you can play with transmission and recovery rates here

par(mfrow = c(1, 2))
plot(bSEIRrK$MaxInf ~ bSEIRrK$r_r, main = expression(paste("Effect of ", r[r], " on Size")),
    xlab = expression(r[r]), ylab = "Outbreak Size")
plot(bSEIRrK$Thresh100 ~ bSEIRrK$r_r, main = expression(paste("Effect of ", r[r],
    " on Duration")), xlab = expression(r[r]), ylab = "Detectable Duration (days)")
```

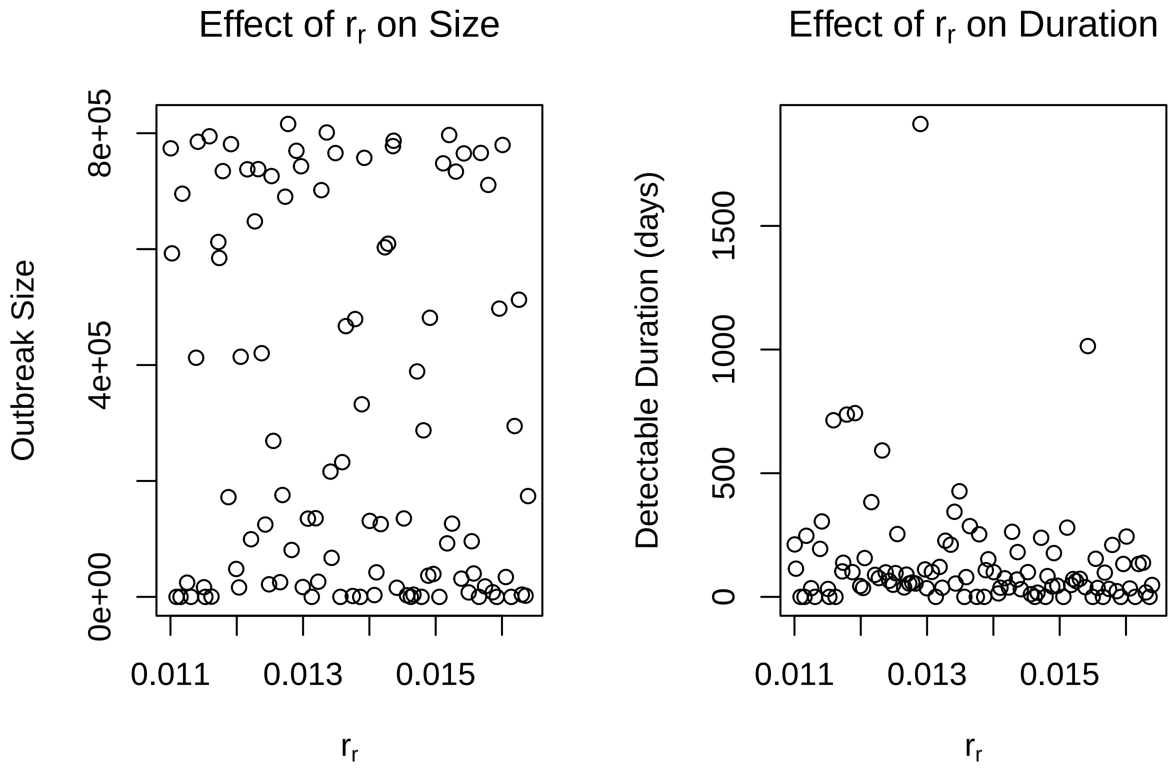


```r
plot(bSEIRrK$MaxInf ~ bSEIRrK$K_r, main = expression(paste("Effect of ", K[r], " on Size")),
    xlab = expression(K[r]), ylab = "Outbreak Size")
plot(bSEIRrK$Thresh100 ~ bSEIRrK$K_r, main = expression(paste("Effect of ", K[r],
    " on Duration")), xlab = expression(K[r]), ylab = "Detectable Duration (days)")
```

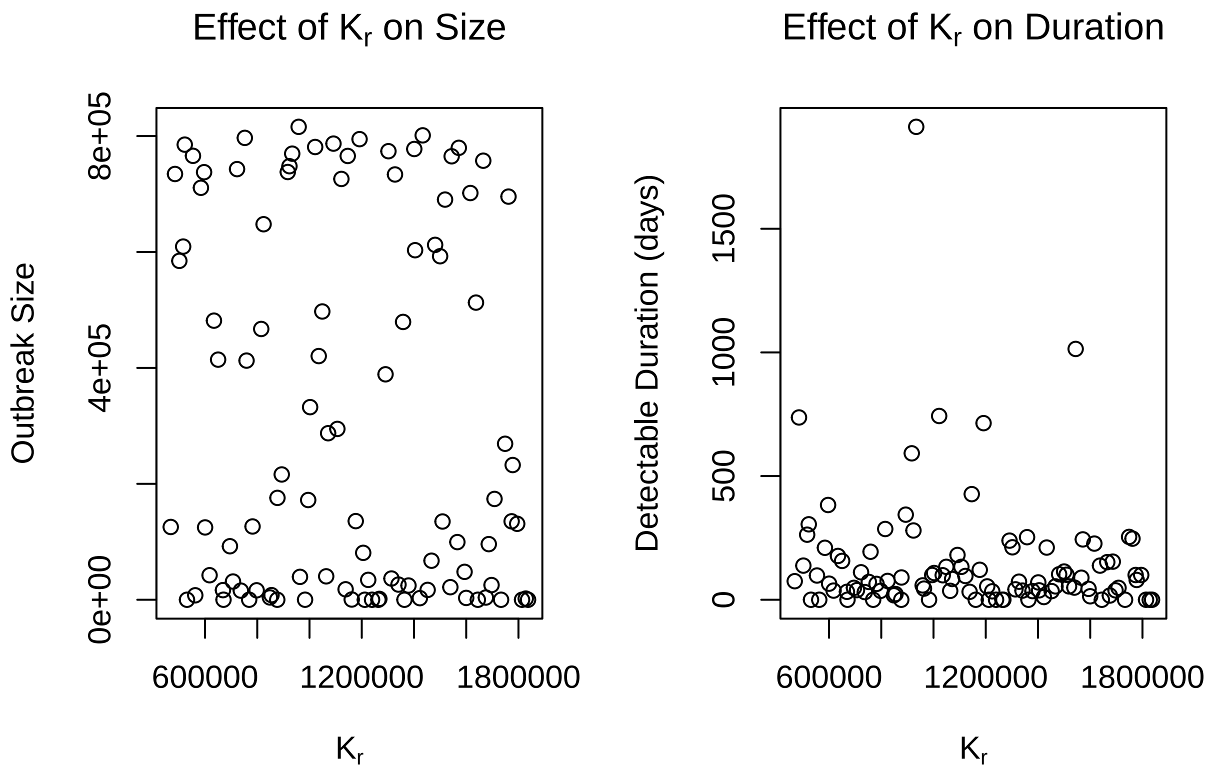


```
plot(bSEIRrK$MaxInf ~ bSEIRrK$p_r, main = expression(paste("Effect of ", p[r], " on Size")),
    xlab = expression(p[r]), ylab = "Outbreak Size")
plot(bSEIRrK$Thresh100 ~ bSEIRrK$p_r, main = expression(paste("Effect of ", p[r],
    " on Duration")), xlab = expression(p[r]), ylab = "Detectable Duration (days)")
```

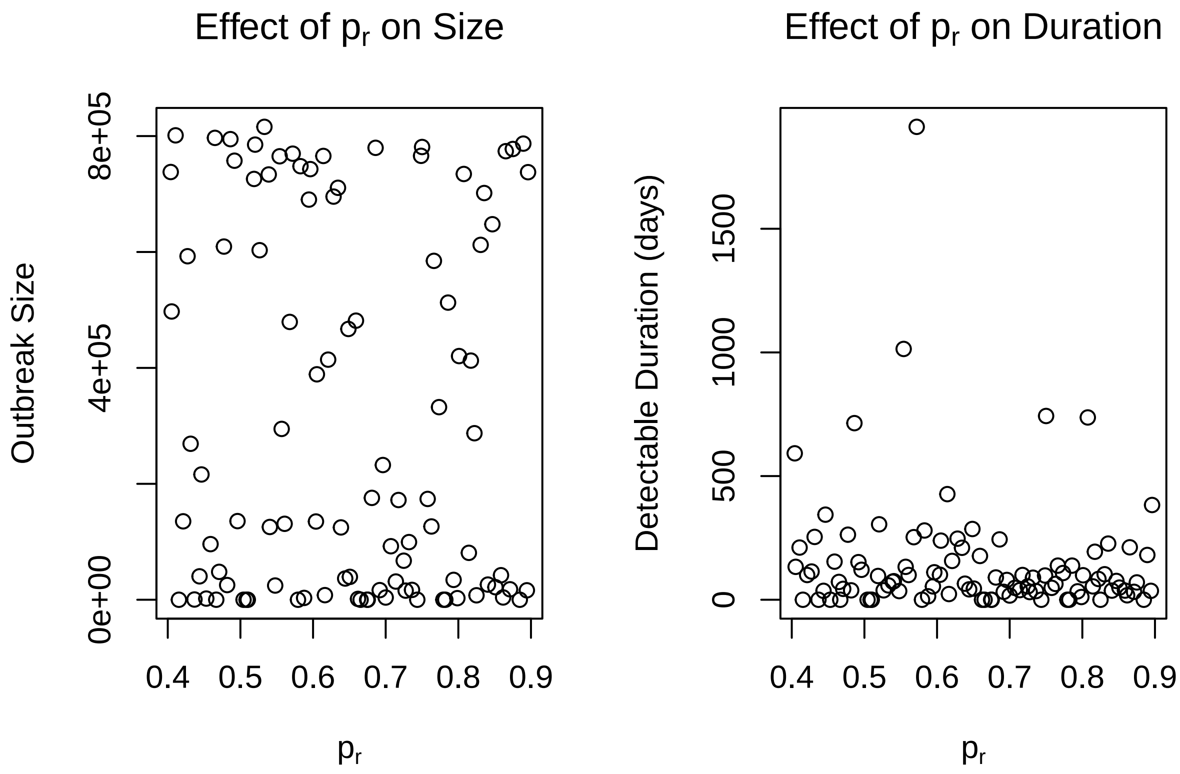


```
plot(bSEIRrK$MaxInf ~ bSEIRrK$d_r, main = expression(paste("Effect of ", d[r], " on Size")),
    xlab = expression(d[r]), ylab = "Outbreak Size")
plot(bSEIRrK$Thresh100 ~ bSEIRrK$d_r, main = expression(paste("Effect of ", d[r],
    " on Duration")), xlab = expression(d[r]), ylab = "Detectable Duration (days)")
```

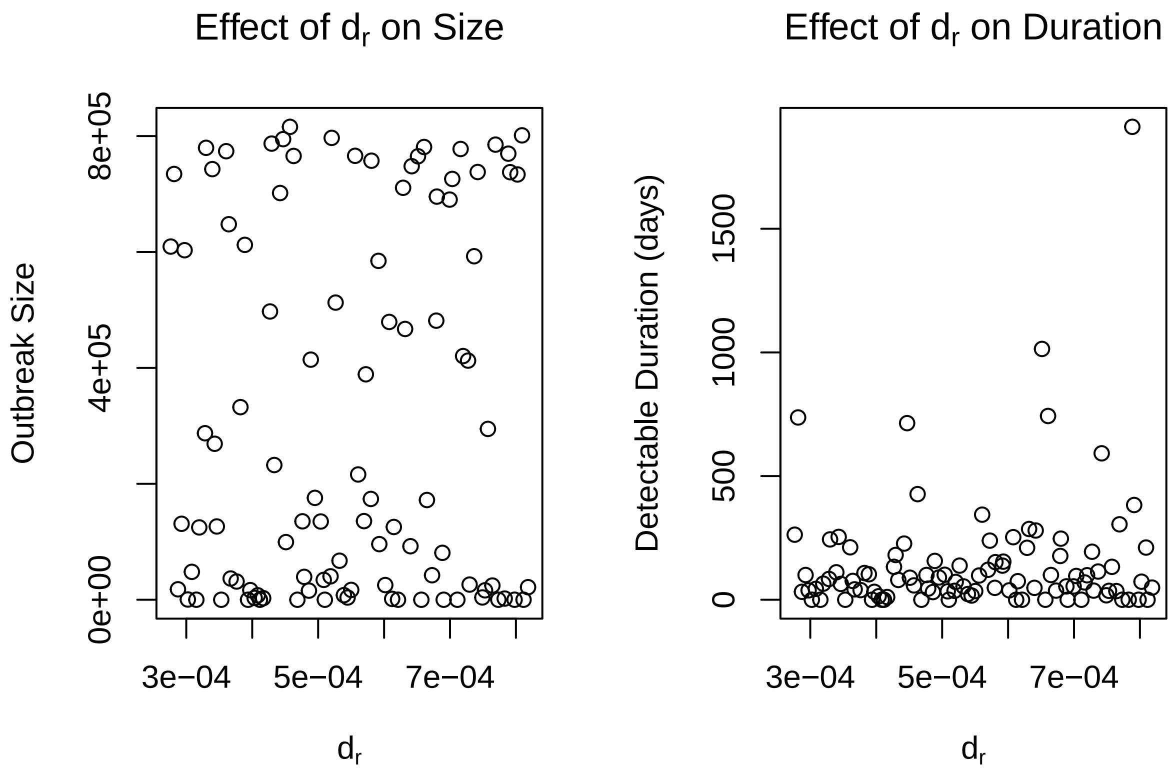


```
plot(bSEIRrK$MaxInf ~ bSEIRrK$beta_r, main = expression(paste("Effect of ", beta[r],
    " on Size")), xlab = expression(beta[r]), ylab = "Outbreak Size")
plot(bSEIRrK$Thresh100 ~ bSEIRrK$beta_r, main = expression(paste("Effect of ", beta[r],
    " on Duration")), xlab = expression(beta[r]), ylab = "Detectable Duration (days)")
```

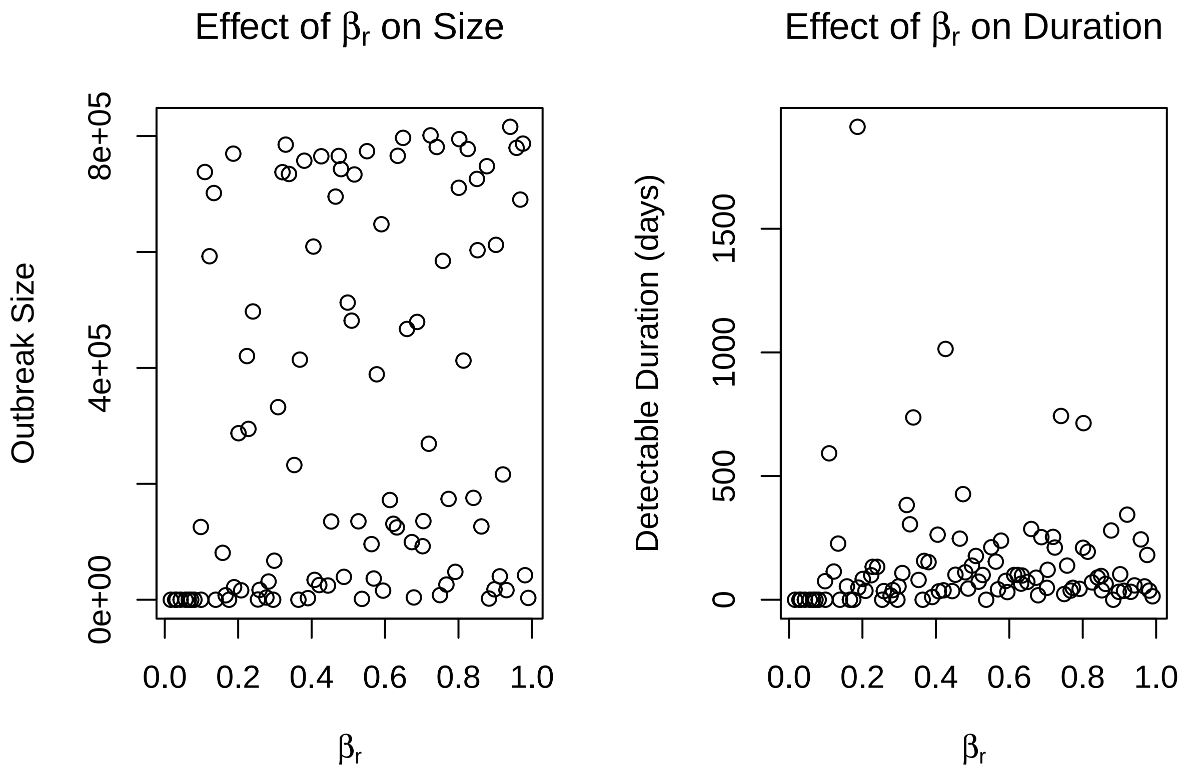


```r
plot(bSEIRrK$MaxInf ~ bSEIRrK$alpha, main = expression(paste("Effect of ", alpha,
    " on Size")), xlab = expression(alpha), ylab = "Outbreak Size")
plot(bSEIRrK$Thresh100 ~ bSEIRrK$alpha, main = expression(paste("Effect of ", alpha,
    " on Duration")), xlab = expression(alpha), ylab = "Detectable Duration (days)")
```

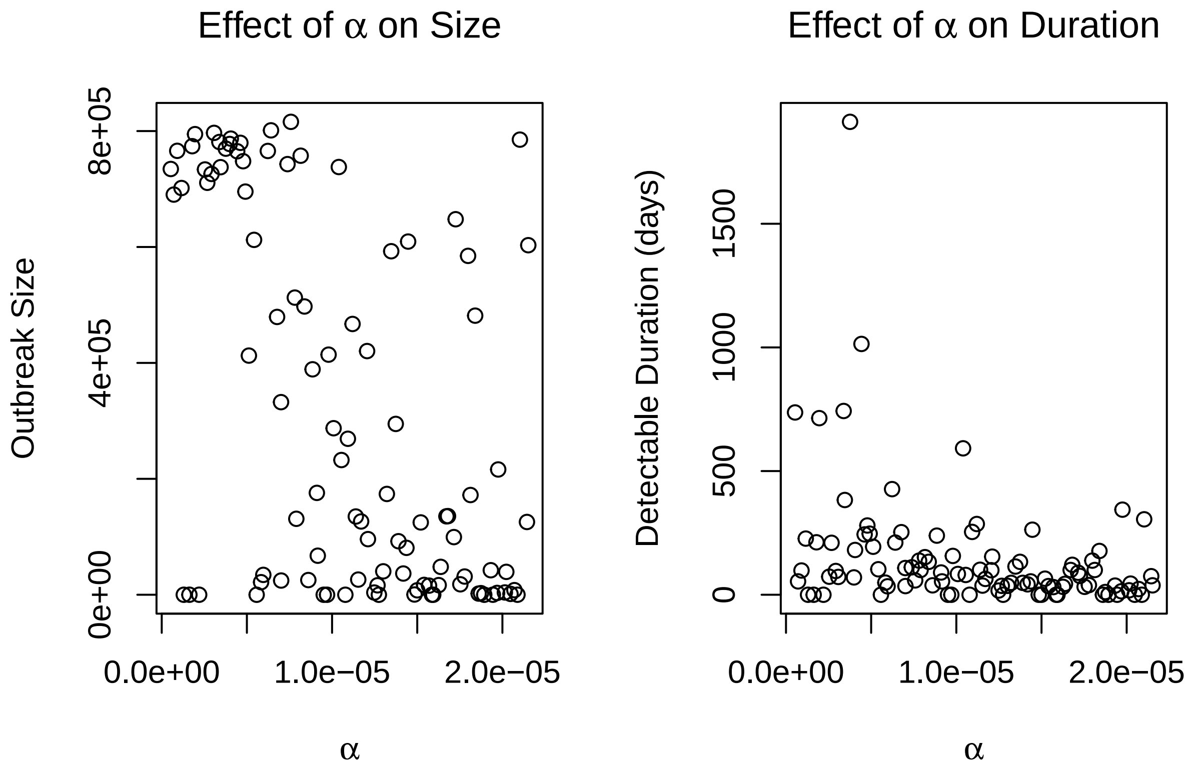


```
plot(bSEIRrK$MaxInf ~ bSEIRrK$gamma_r, main = expression(paste("Effect of ", gamma[r],
    " on Size")), xlab = expression(gamma[r]), ylab = "Outbreak Size")
plot(bSEIRrK$Thresh100 ~ bSEIRrK$gamma_r, main = expression(paste("Effect of ", gamma[r],
    " on Duration")), xlab = expression(gamma[r]), ylab = "Detectable Duration (days)")
```

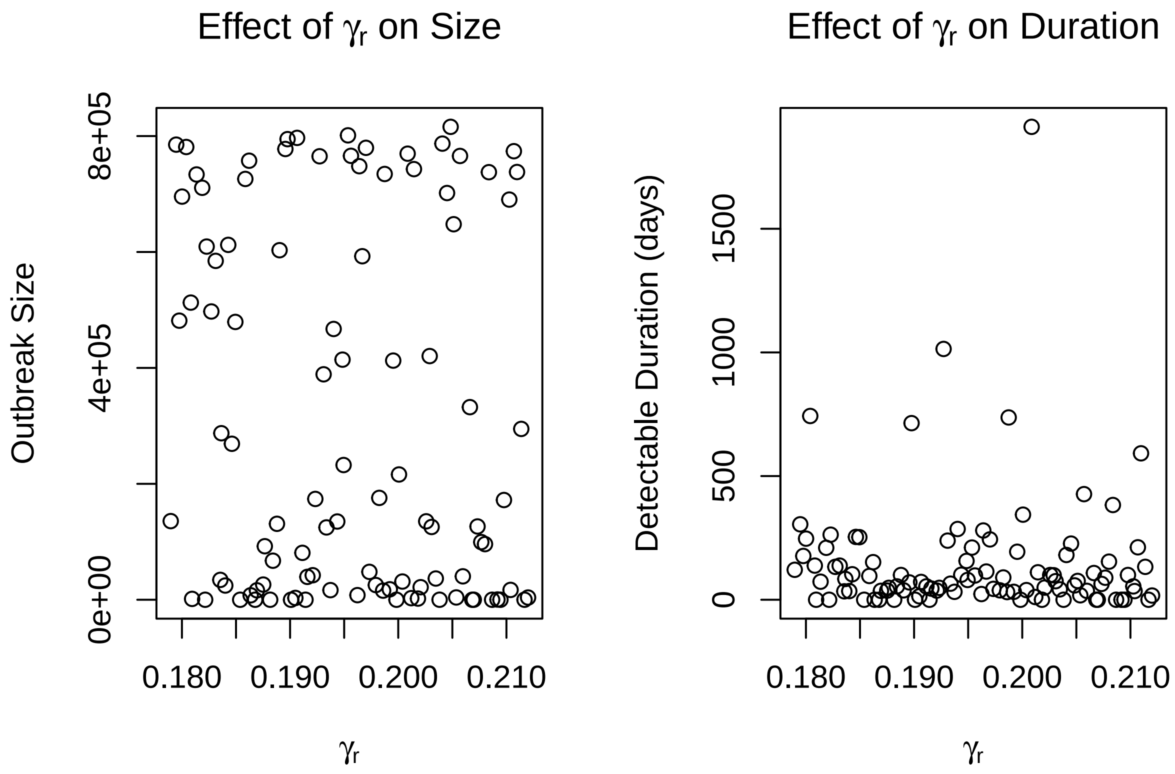


```
plot(bSEIRrK$MaxInf ~ bSEIRrK$g_r, main = expression(paste("Effect of ", g[r], " on Size")),
    xlab = expression(g[r]), ylab = "Outbreak Size")
plot(bSEIRrK$Thresh100 ~ bSEIRrK$g_r, main = expression(paste("Effect of ", g[r],
    " on Duration")), xlab = expression(g[r]), ylab = "Detectable Duration (days)")
```

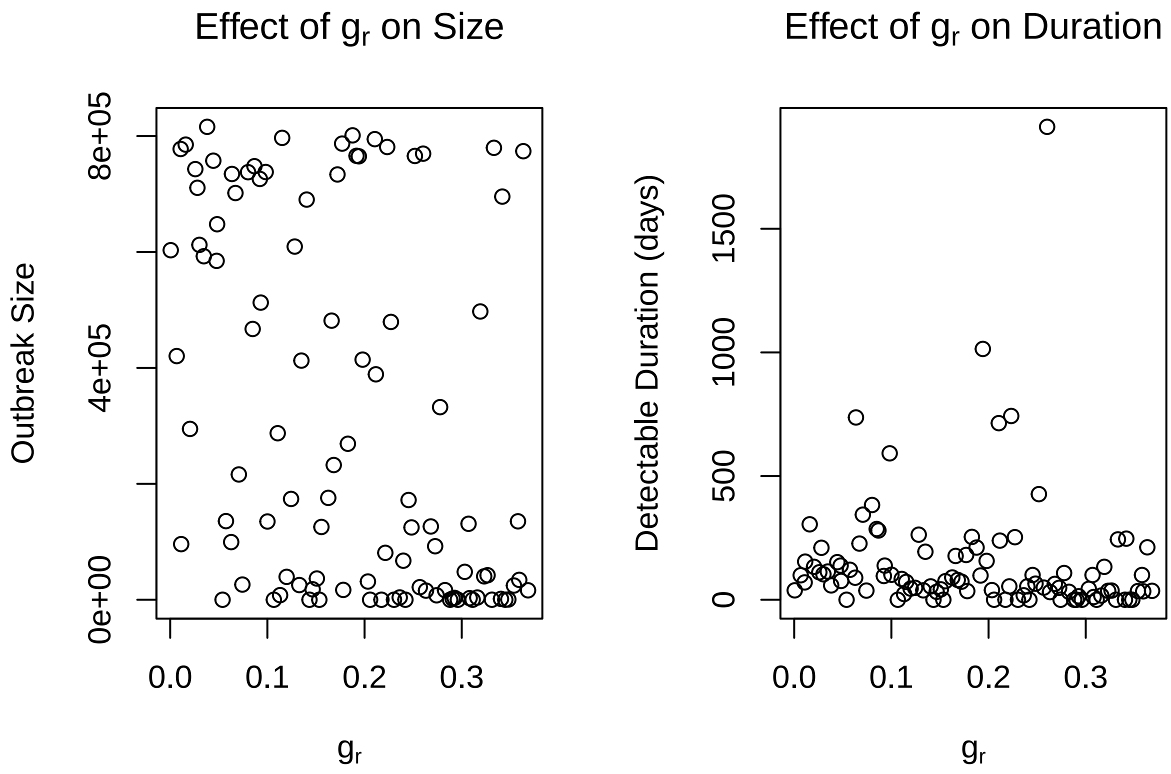


```
plot(bSEIRrK$MaxInf ~ bSEIRrK$r_f, main = expression(paste("Effect of ", r[f], " on Size")),
    xlab = expression(r[f]), ylab = "Outbreak Size")
plot(bSEIRrK$Thresh100 ~ bSEIRrK$r_f, main = expression(paste("Effect of ", r[f],
    " on Duration")), xlab = expression(r[f]), ylab = "Detectable Duration (days)")
```

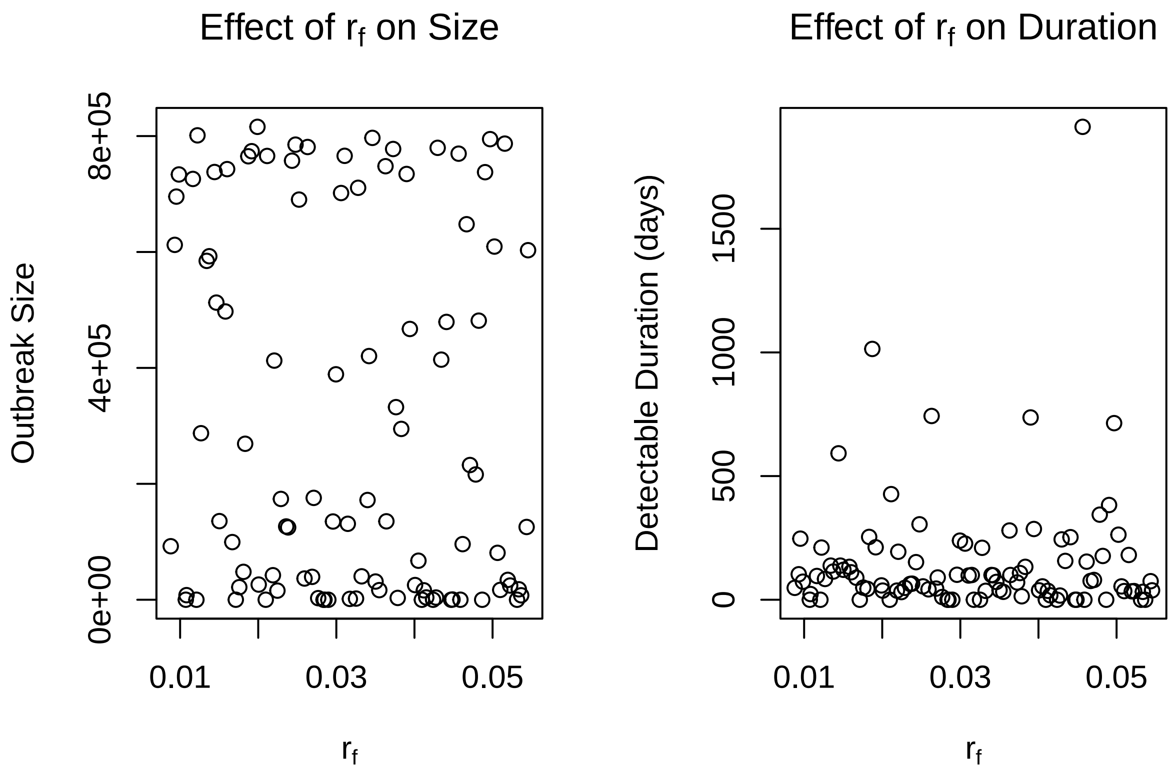


```
plot(bSEIRrK$MaxInf ~ bSEIRrK$K_f, main = expression(paste("Effect of ", K[f], " on Size")),
    xlab = expression(K[f]), ylab = "Outbreak Size")
plot(bSEIRrK$Thresh100 ~ bSEIRrK$K_f, main = expression(paste("Effect of ", K[f],
    " on Duration")), xlab = expression(K[f]), ylab = "Detectable Duration (days)")
```

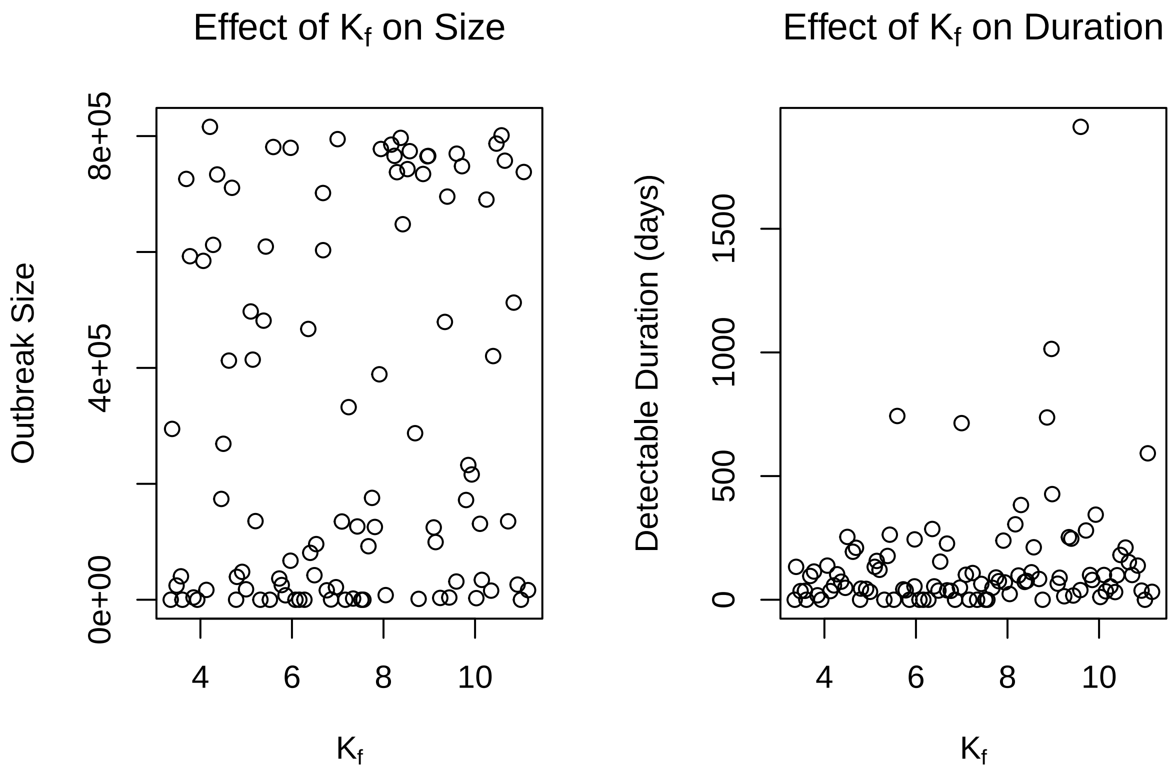


```
plot(bSEIRrK$MaxInf ~ bSEIRrK$d_f, main = expression(paste("Effect of ", d[f], " on Size")),
    xlab = expression(d[f]), ylab = "Outbreak Size")
plot(bSEIRrK$Thresh100 ~ bSEIRrK$d_f, main = expression(paste("Effect of ", d[f],
    " on Duration")), xlab = expression(d[f]), ylab = "Detectable Duration (days)")
```

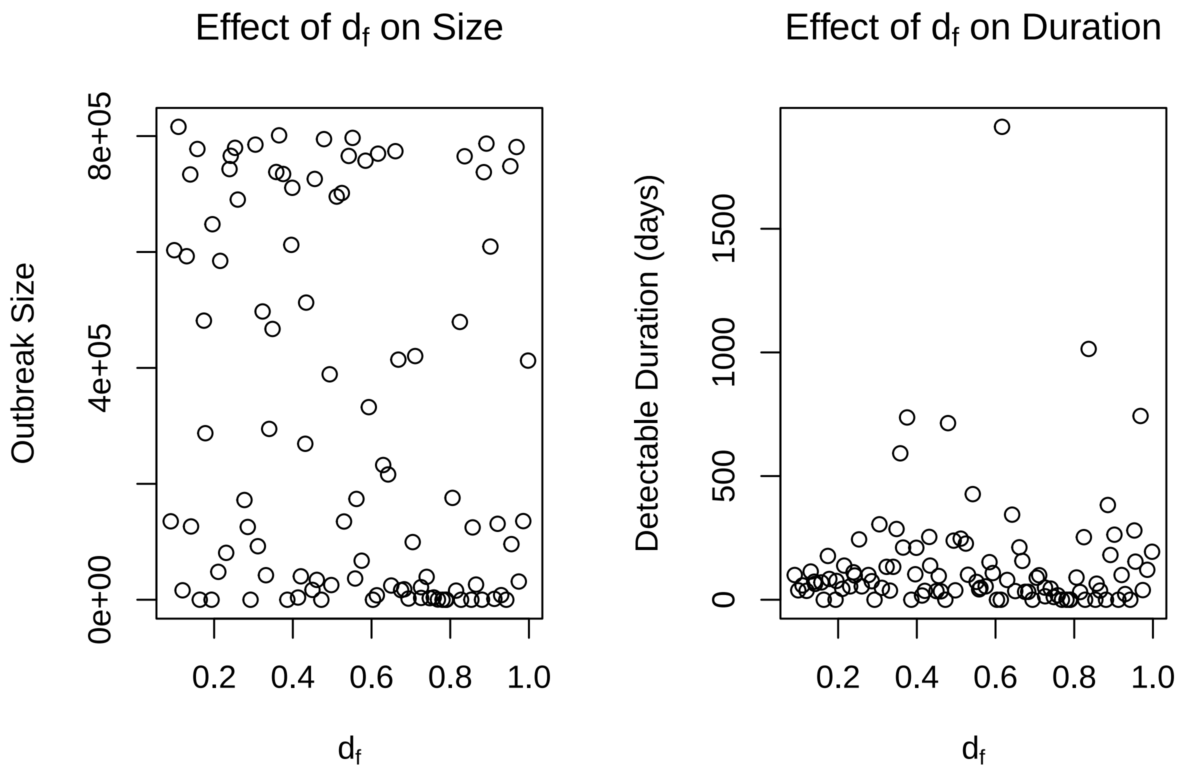


```
plot(bSEIRrK$MaxInf ~ bSEIRrK$beta_h, main = expression(paste("Effect of ", beta[b],
     " on Size")), xlab = expression(beta[b]), ylab = "Outbreak Size")
plot(bSEIRrK$Thresh100 ~ bSEIRrK$beta_h, main = expression(paste("Effect of ", beta[b],
     " on Duration")), xlab = expression(beta[b]), ylab = "Detectable Duration (days)")
```

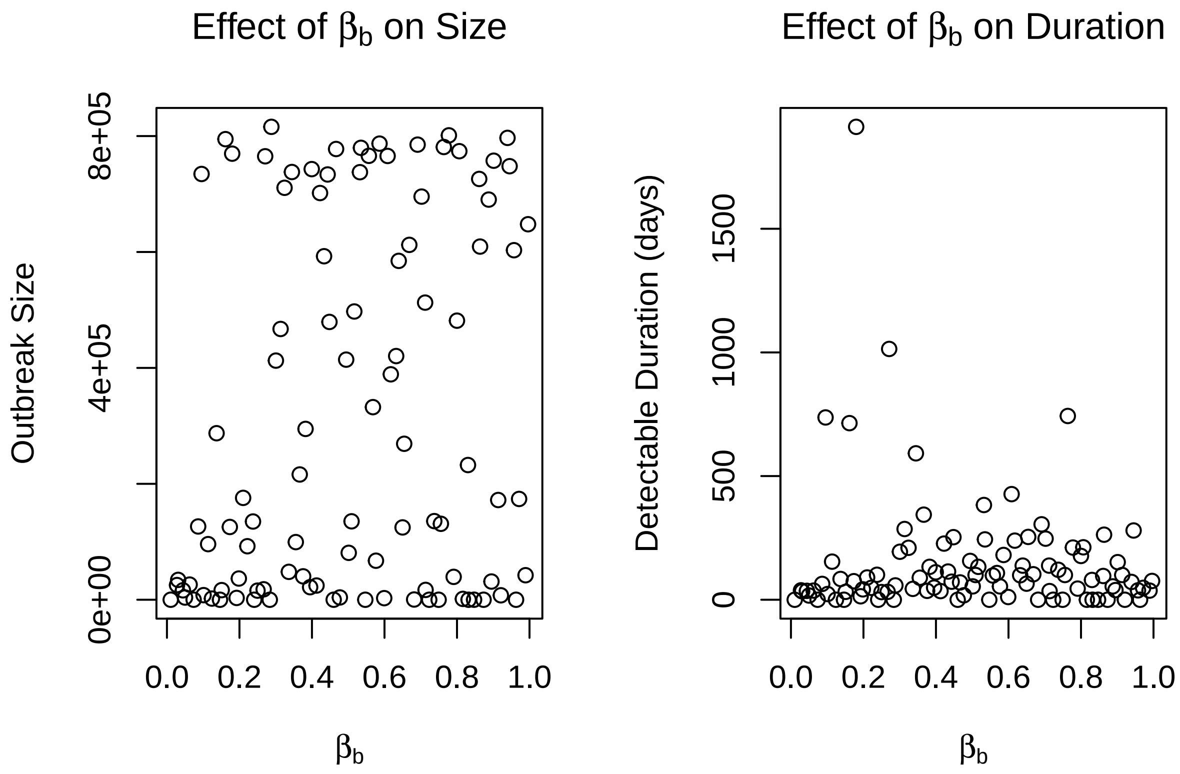


```
plot(bSEIRrK$MaxInf ~ bSEIRrK$sigma_h, main = expression(paste("Effect of ", sigma[b],
    " on Size")), xlab = expression(sigma[b]), ylab = "Outbreak Size")
plot(bSEIRrK$Thresh100 ~ bSEIRrK$sigma_h, main = expression(paste("Effect of ", sigma[b],
    " on Duration")), xlab = expression(sigma[b]), ylab = "Detectable Duration (days)")
```

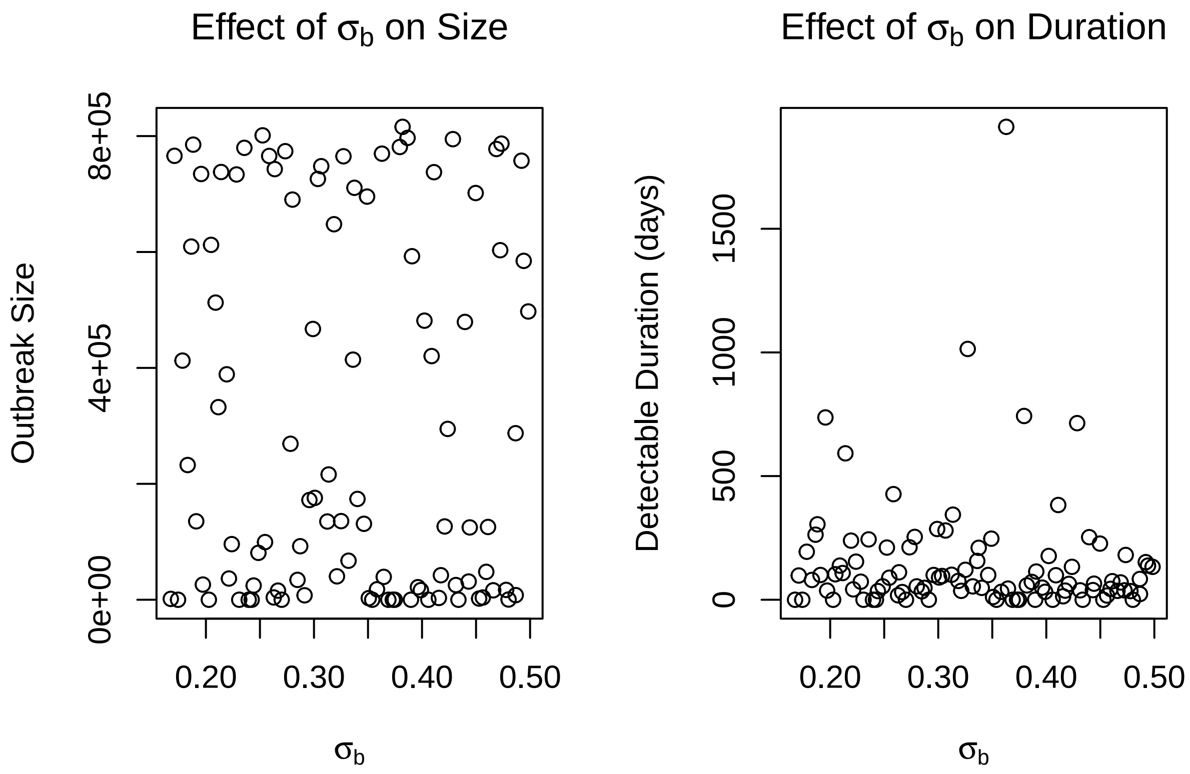


```
plot(bSEIRrK$MaxInf ~ bSEIRrK$gamma_h, main = expression(paste("Effect of ", gamma[b],
    " on Size")), xlab = expression(gamma[b]), ylab = "Outbreak Size")
plot(bSEIRrK$Thresh100 ~ bSEIRrK$gamma_h, main = expression(paste("Effect of ", gamma[b],
    " on Duration")), xlab = expression(gamma[b]), ylab = "Detectable Duration (days)")
```

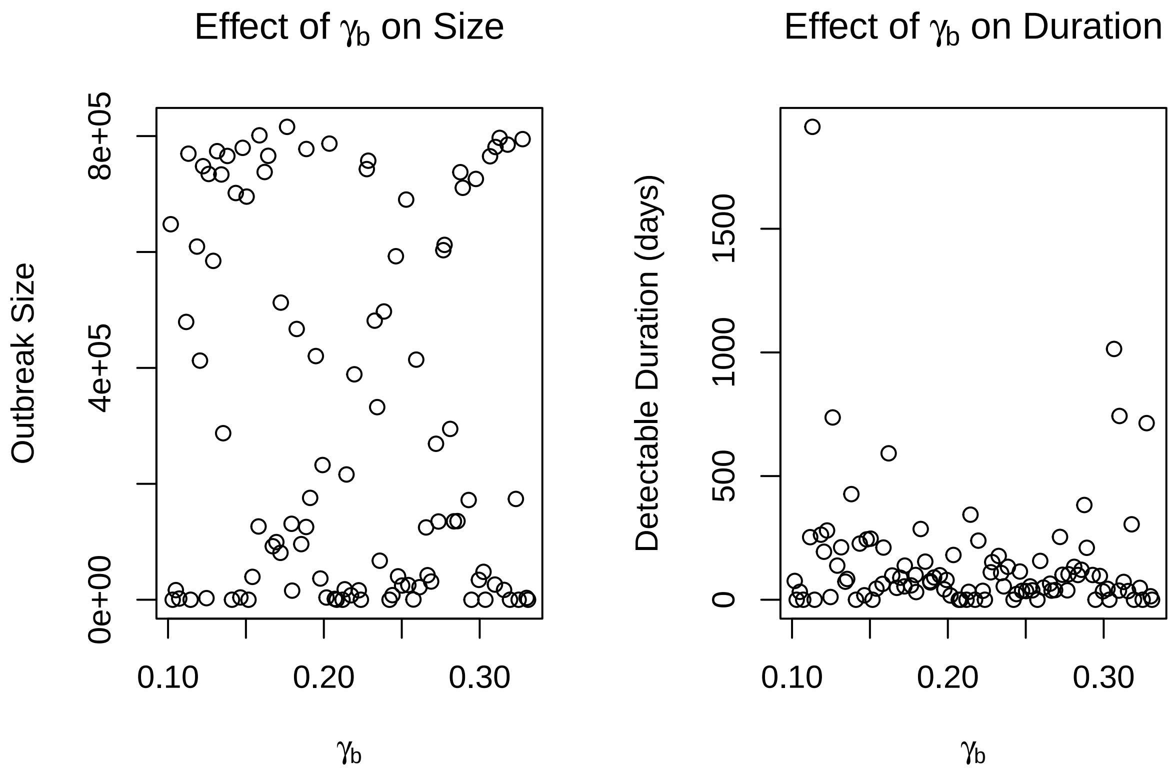


```r
plot(bSEIRrK$MaxInf ~ bSEIRrK$g_h, main = expression(paste("Effect of ", g[h], " on Size")),
    xlab = expression(g[h]), ylab = "Outbreak Size")
plot(bSEIRrK$Thresh100 ~ bSEIRrK$g_h, main = expression(paste("Effect of ", g[h],
    " on Duration")), xlab = expression(g[h]), ylab = "Detectable Duration (days)")
```

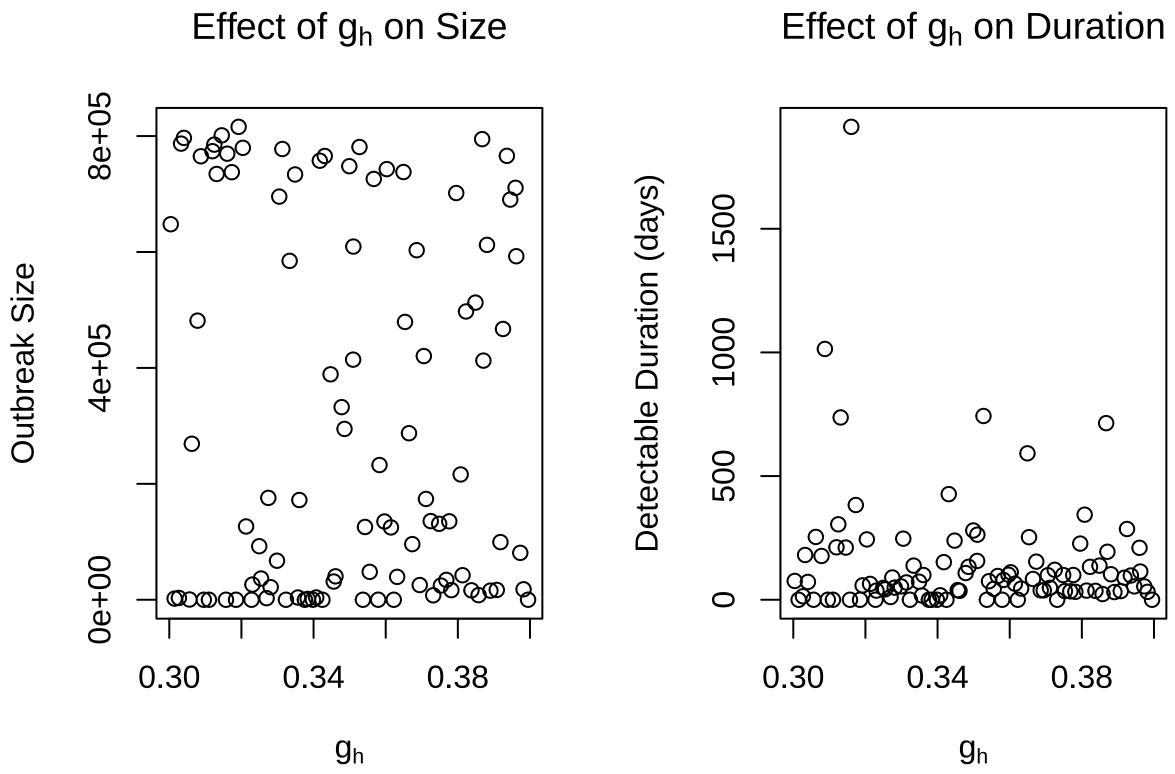


```
plot(bSEIRrK$MaxInf ~ bSEIRrK$b_h, main = expression(paste("Effect of ", b[h], " on Size")),
    xlab = expression(b[h]), ylab = "Outbreak Size")
plot(bSEIRrK$Thresh100 ~ bSEIRrK$b_h, main = expression(paste("Effect of ", b[h],
    " on Duration")), xlab = expression(b[h]), ylab = "Detectable Duration (days)")
```

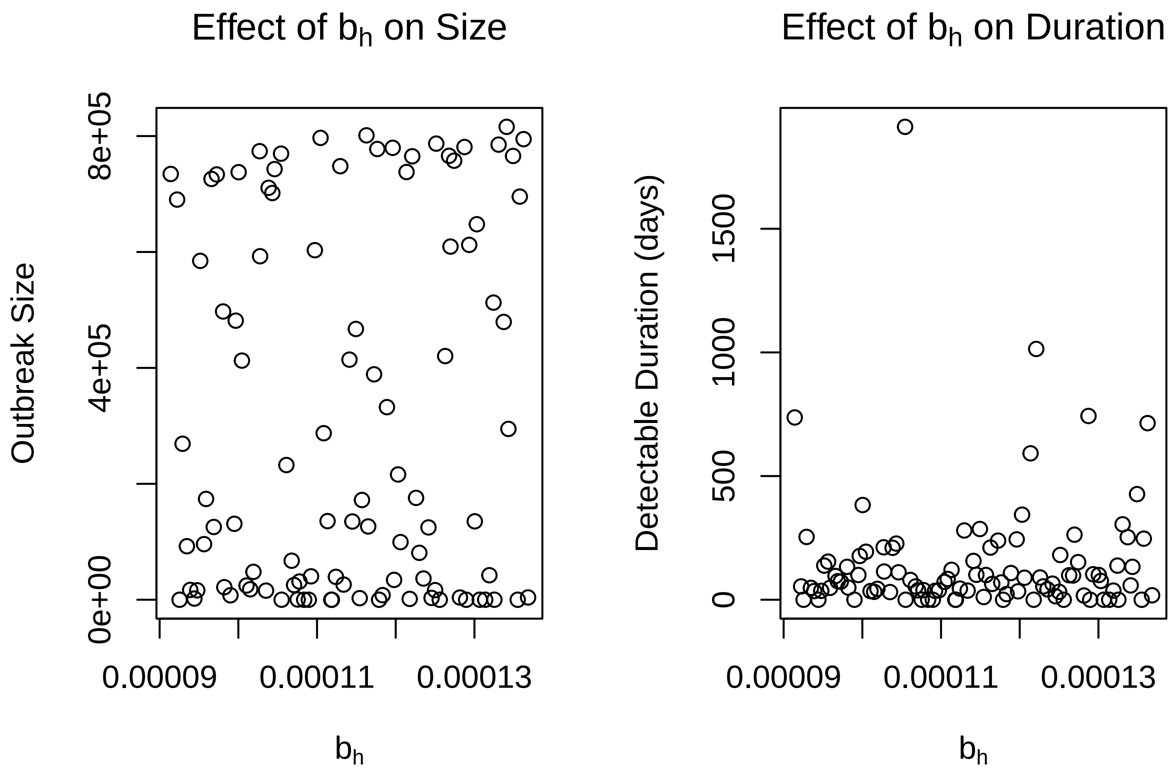


```
plot(bSEIRrK$MaxInf ~ bSEIRrK$d_h, main = expression(paste("Effect of ", d[h], " on Size")),
    xlab = expression(d[h]), ylab = "Outbreak Size")
plot(bSEIRrK$Thresh100 ~ bSEIRrK$d_h, main = expression(paste("Effect of ", d[h],
    " on Duration")), xlab = expression(d[h]), ylab = "Detectable Duration (days)")
```

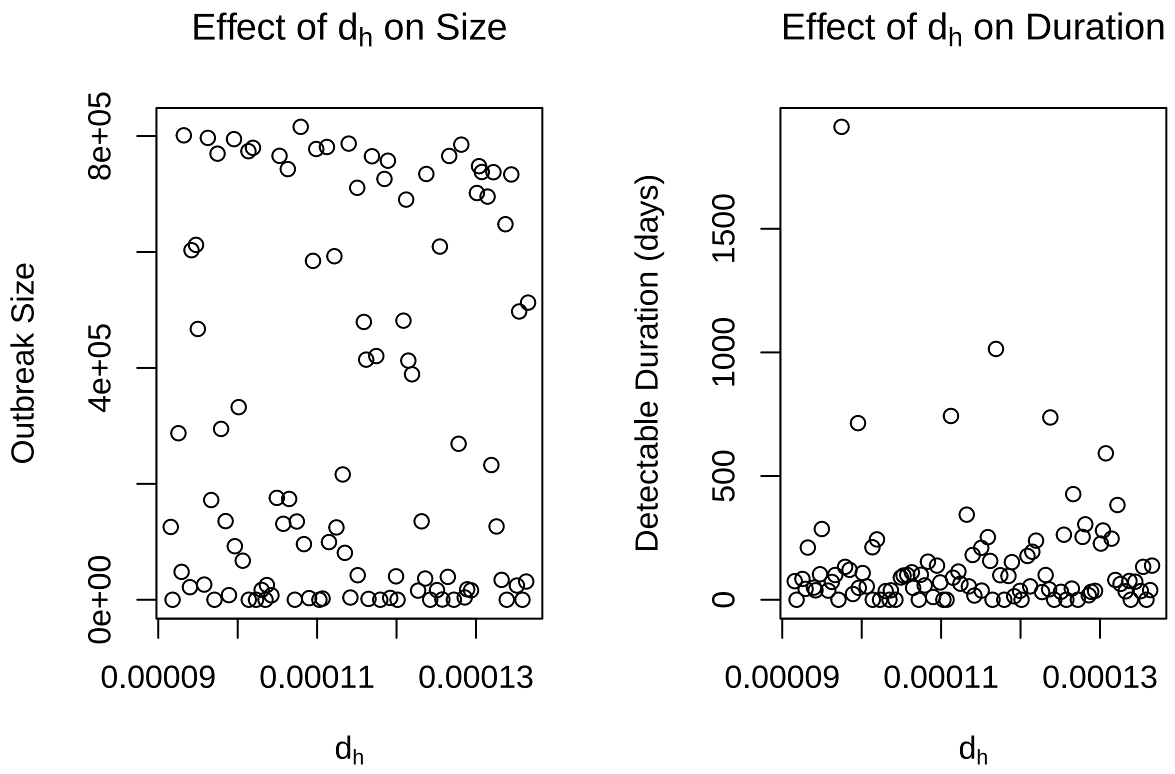


```
par(mfrow = c(1, 2))
boxplot(bSEIRrK$MaxInf, main = "Outbreak Size", ylab = "Number of Dead Humans", ylim = c(0,
    923406))
boxplot(bSEIRrK$Thresh100, main = "Outbreak Duration", ylab = "Time (Days)")
```

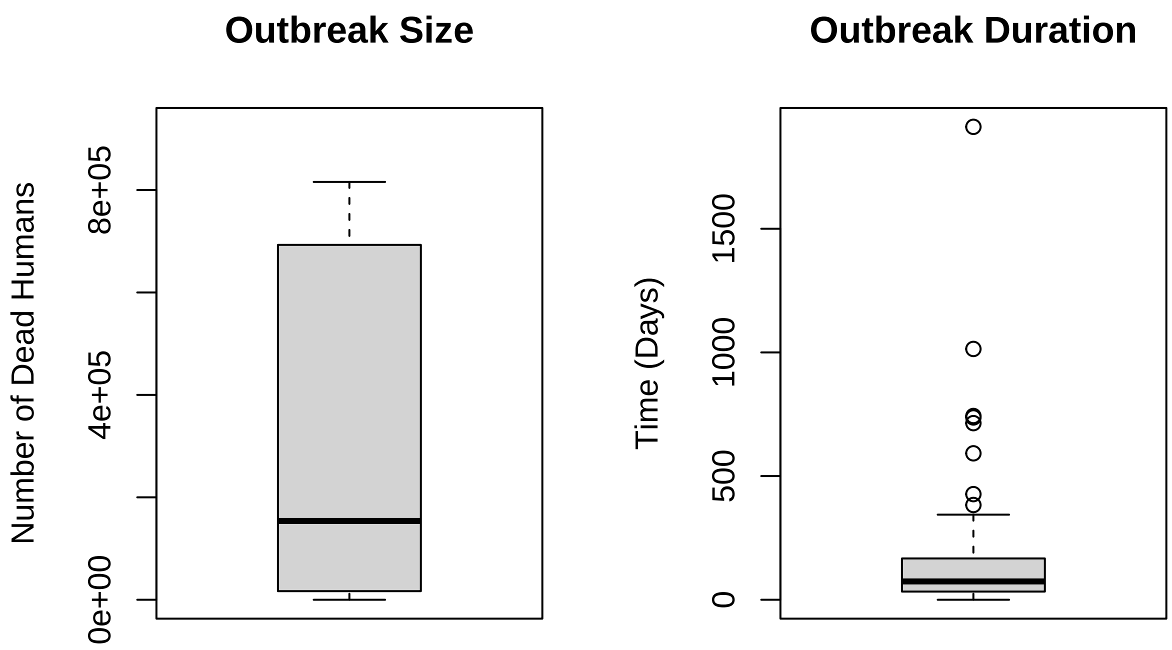


```r
bonferroni.alpha <- 0.05/length(parameters)
prcc_size <- pcc(bSEIRrK[, 1:length(parameters)], bSEIRrK$MaxInf, nboot = niter,
    rank = TRUE, conf = 1 - bonferroni.alpha)
prcc_duration <- pcc(bSEIRrK[, 1:length(parameters)], bSEIRrK$Thresh100, nboot = niter,
    rank = TRUE, conf = 1 - bonferroni.alpha)

# plot correlation coefficients and confidence intervals for epidemic size and
# duration
size <- prcc_size$PRCC
size$param <- rownames(size)
colnames(size)[4:5] <- c("maxCI", "minCI")
size$maxCI[which(size$maxCI > 1)] <- 1
size$maxCI[which(size$maxCI < -1)] <- -1
size$minCI[which(size$minCI > 1)] <- 1
size$minCI[which(size$minCI < -1)] <- -1

duration <- prcc_duration$PRCC
duration$param <- rownames(duration)
colnames(duration)[4:5] <- c("maxCI", "minCI")
duration$maxCI[which(duration$maxCI > 1)] <- 1
duration$maxCI[which(duration$maxCI < -1)] <- -1
duration$minCI[which(duration$minCI > 1)] <- 1
duration$minCI[which(duration$minCI < -1)] <- -1


A <- ggplot(size, aes(x = param, y = original)) + geom_point(size = 4) + geom_errorbar(aes(ymax = maxCI
```

```
    ymin = minCI)) + ggtitle("A") + xlab("Parameters") + ylab("Partial Rank Correlation Coefficients") +
    scale_x_discrete(labels = c(r_r = expression(r[r]), d_r = expression(d[r]), K_r = expression(K[r]),
        p_r = expression(p[r]), alpha = expression(alpha), beta_h = expression(beta[b]),
        beta_r = expression(beta[r]), sigma_h = expression(sigma[b]), b_h = expression(b[h]),
        d_h = expression(d[h]), d_f = expression(d[f]), gamma_h = expression(gamma[b]),
        gamma_r = expression(gamma[r]), g_h = expression(g[h]), g_r = expression(g[r]),
        K_f = expression(K[f]), r_f = expression(r[f]))) + ylim(-1, 1)

B <- ggplot(duration, aes(x = param, y = original)) + geom_point(size = 4) + geom_errorbar(aes(ymax = ma
    ymin = minCI)) + ggtitle("B") + xlab("Parameters") + ylab(" ") + scale_x_discrete(labels = c(r_r = e
    d_r = expression(d[r]), K_r = expression(K[r]), p_r = expression(p[r]), alpha = expression(alpha),
    beta_h = expression(beta[b]), beta_r = expression(beta[r]), sigma_h = expression(sigma[b]),
    b_h = expression(b[h]), d_h = expression(d[h]), d_f = expression(d[f]), gamma_h = expression(gamma[l
    gamma_r = expression(gamma[r]), g_h = expression(g[h]), g_r = expression(g[r]),
    K_f = expression(K[f]), r_f = expression(r[f]))) + ylim(-1, 1)

multiplot(A, B, cols = 2)
```

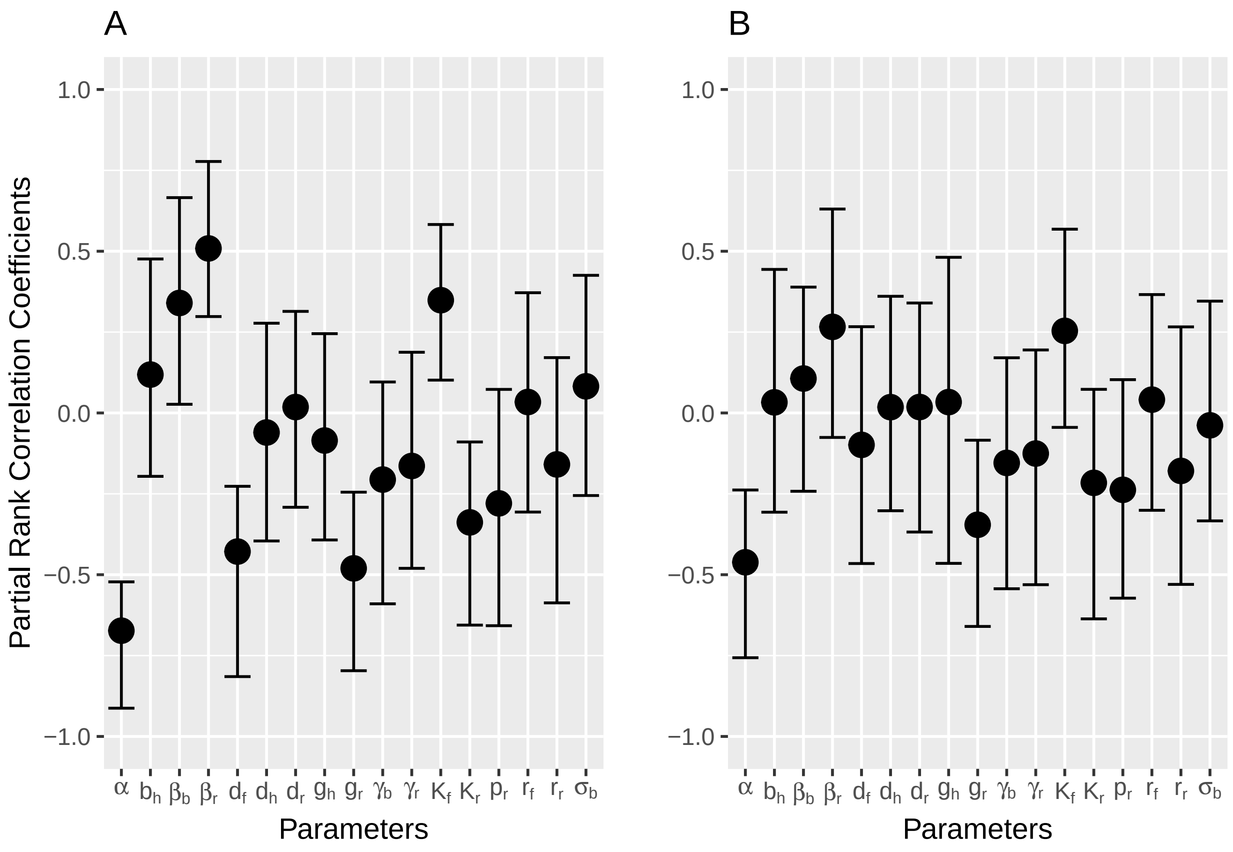


```
# tiff('FigureS16BubonicSEIRrK_PRCCuniform.tiff', height =8.7 , width =10,
# units = 'cm', compression = 'lzw', res = 1200) multiplot(A, B, cols=2)
# dev.off()
```

Bubonic/Pneumonic SEIR

```
parameters <- c(beta_r = 0.09, alpha = 3/923406, gamma_r = 1/5.15, g_r = 0.1, r_f = 0.0084,
    K_f = 6, d_f = 1/5, beta_b = 0.19, beta_p = 0.45, sigma_b = 1/6, sigma_p = 1/4.3,
    gamma_b = 1/10, gamma_p = 1/2.5, p = 0.2, g_h = 0.34, b_h = 1/(25 * 365), d_h = 1/(25 *
        365))  #you can play with transmission and recovery rates here

par(mfrow = c(1, 2))
plot(bpSEIR$MaxInf ~ bpSEIR$beta_r, main = expression(paste("Effect of ", beta[r],
    " on Size")), xlab = expression(beta[r]), ylab = "Outbreak Size")
plot(bpSEIR$Thresh100 ~ bpSEIR$beta_r, main = expression(paste("Effect of ", beta[r],
    " on Duration")), xlab = expression(beta[r]), ylab = "Detectable Duration (days)")
```

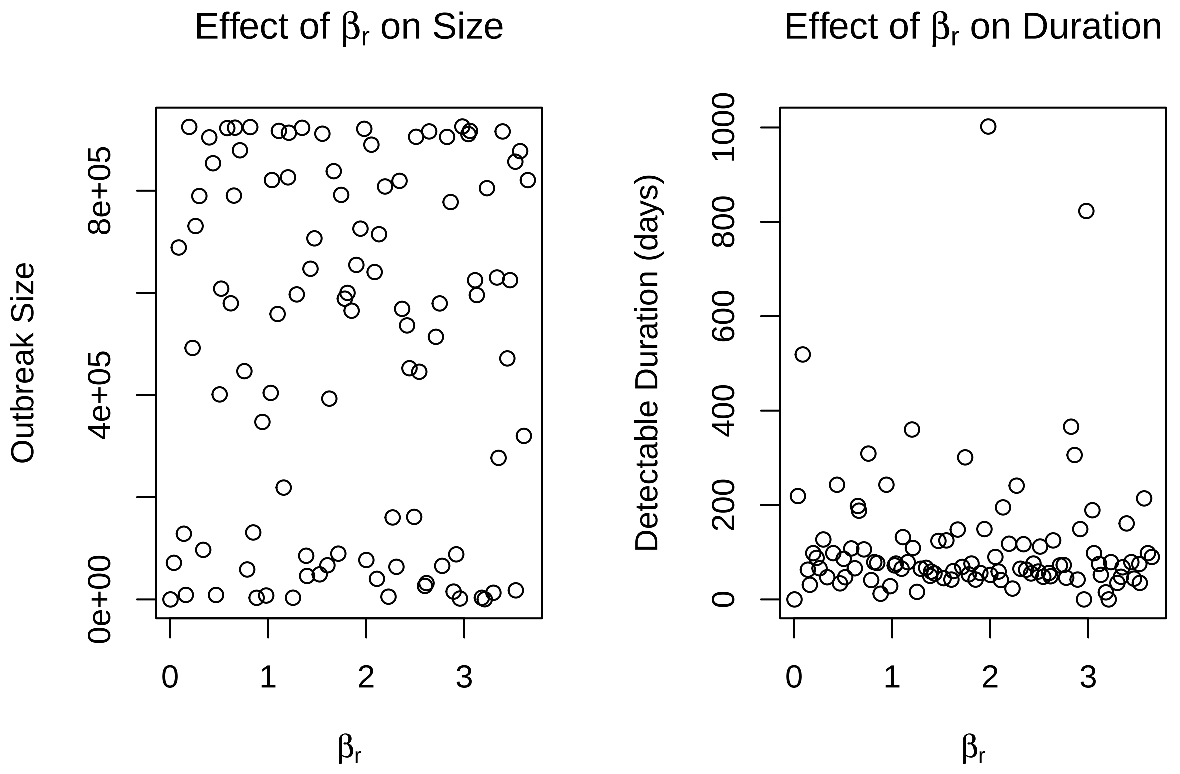


```
plot(bpSEIR$MaxInf ~ bpSEIR$alpha, main = expression(paste("Effect of ", alpha, " on Size")),
    xlab = expression(alpha), ylab = "Outbreak Size")
plot(bpSEIR$Thresh100 ~ bpSEIR$alpha, main = expression(paste("Effect of ", alpha,
    " on Duration")), xlab = expression(alpha), ylab = "Detectable Duration (days)")
```

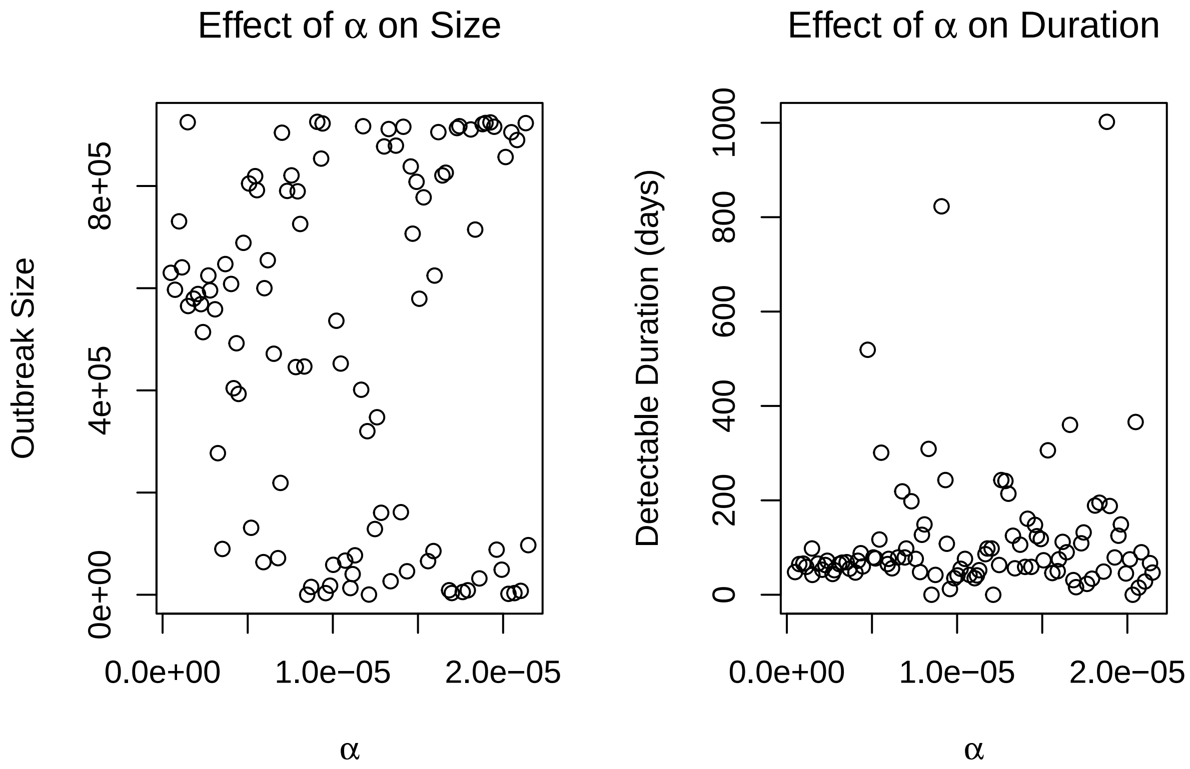


```
plot(bpSEIR$MaxInf ~ bpSEIR$gamma_r, main = expression(paste("Effect of ", gamma[r],
    " on Size")), xlab = expression(gamma[r]), ylab = "Outbreak Size")
plot(bpSEIR$Thresh100 ~ bpSEIR$gamma_r, main = expression(paste("Effect of ", gamma[r],
    " on Duration")), xlab = expression(gamma[r]), ylab = "Detectable Duration (days)")
```

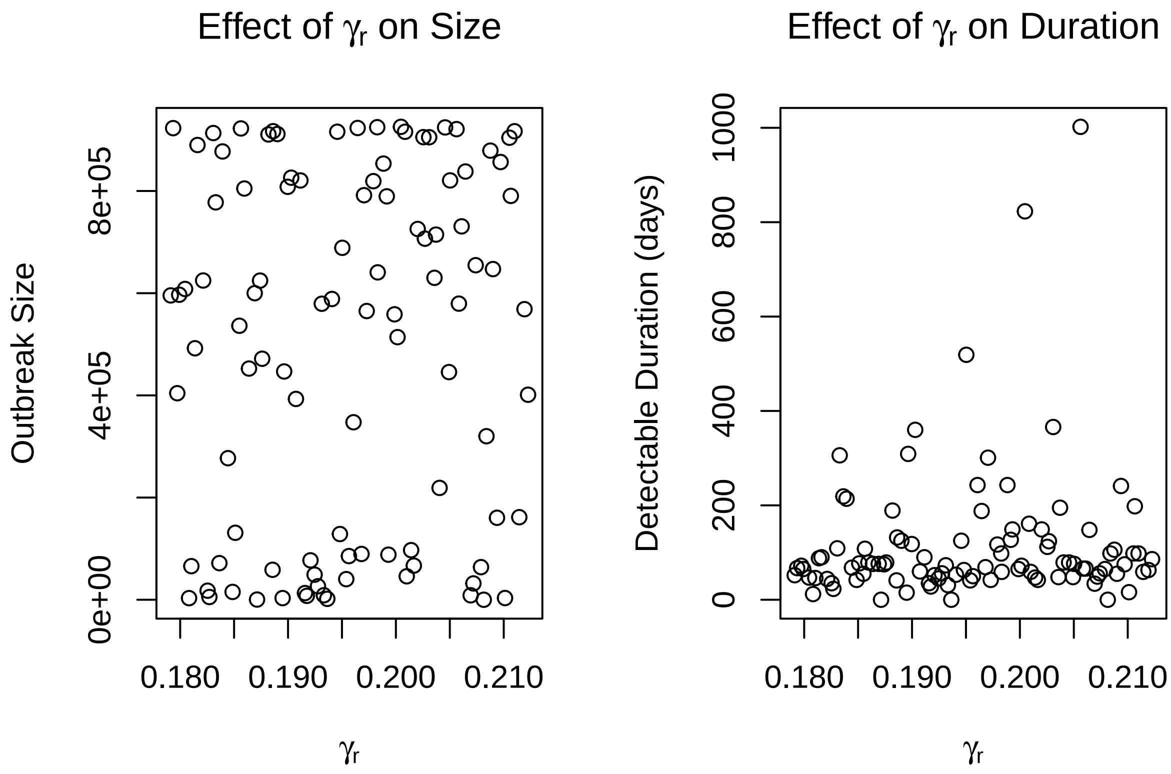


```
plot(bpSEIR$MaxInf ~ bpSEIR$g_r, main = expression(paste("Effect of ", g[r], " on Size")),
    xlab = expression(g[r]), ylab = "Outbreak Size")
plot(bpSEIR$Thresh100 ~ bpSEIR$g_r, main = expression(paste("Effect of ", g[r], " on Duration")),
    xlab = expression(g[r]), ylab = "Detectable Duration (days)")
```

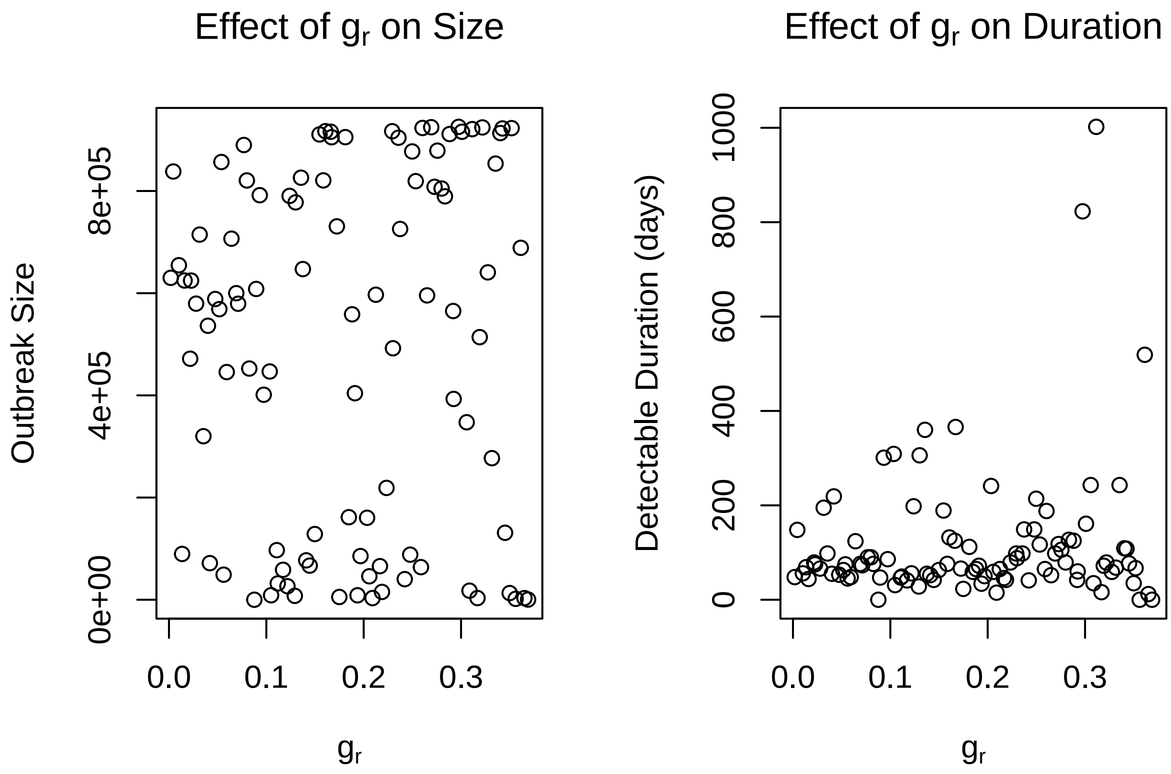


```
plot(bpSEIR$MaxInf ~ bpSEIR$r_f, main = expression(paste("Effect of ", r[f], " on Size")),
    xlab = expression(r[f]), ylab = "Outbreak Size")
plot(bpSEIR$Thresh100 ~ bpSEIR$r_f, main = expression(paste("Effect of ", r[f], " on Duration")),
    xlab = expression(r[f]), ylab = "Detectable Duration (days)")
```

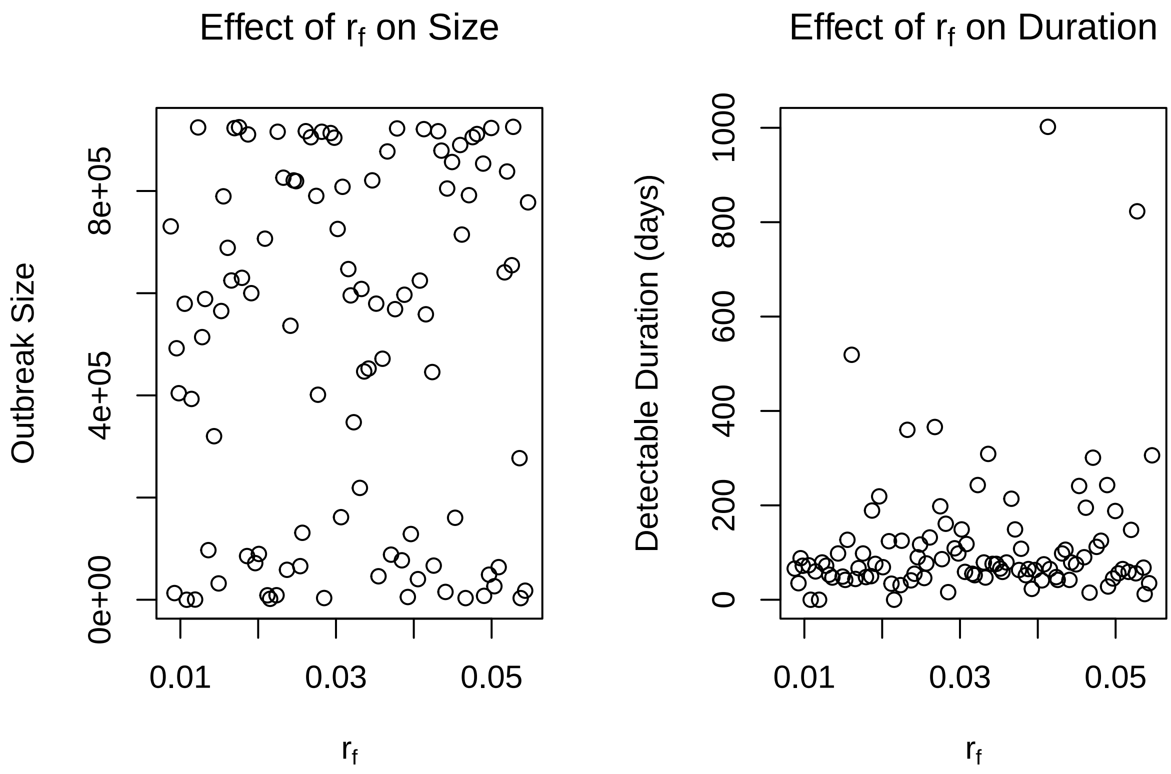


```
plot(bpSEIR$MaxInf ~ bpSEIR$K_f, main = expression(paste("Effect of ", K[f], " on Size")),
    xlab = expression(K[f]), ylab = "Outbreak Size")
plot(bpSEIR$Thresh100 ~ bpSEIR$K_f, main = expression(paste("Effect of ", K[f], " on Duration")),
    xlab = expression(K[f]), ylab = "Detectable Duration (days)")
```

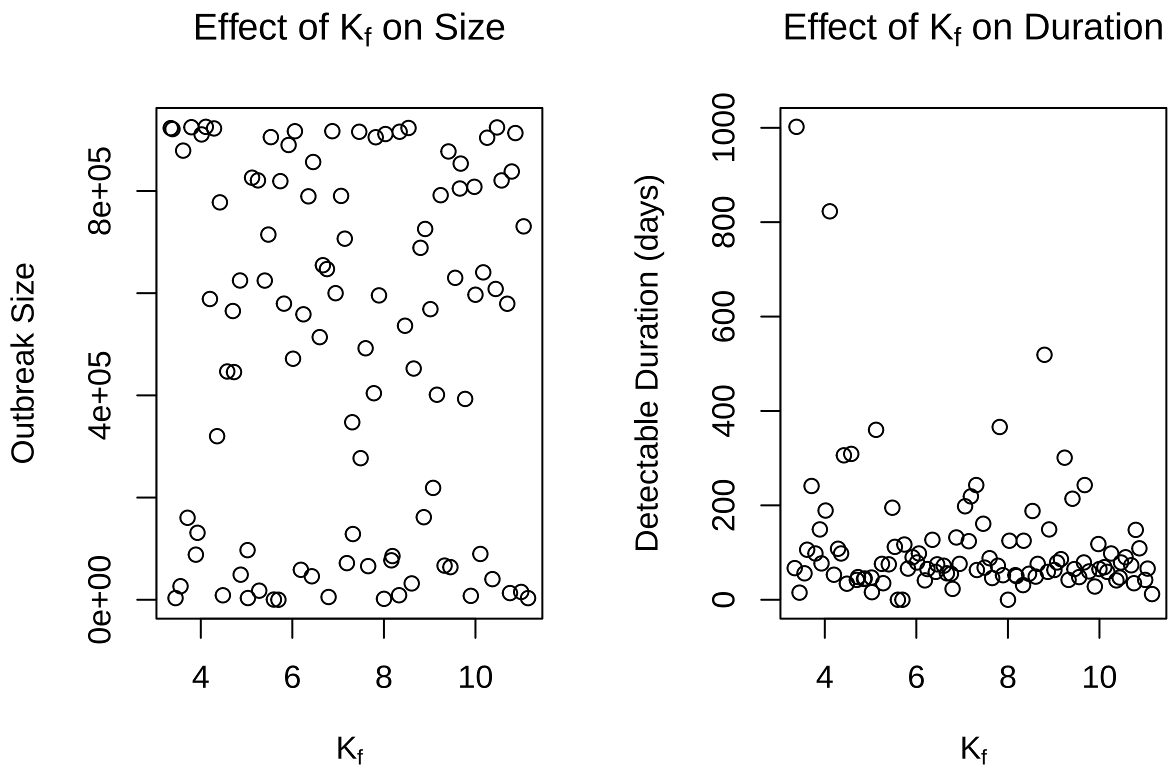


```
plot(bpSEIR$MaxInf ~ bpSEIR$d_f, main = expression(paste("Effect of ", d[f], " on Size")),
    xlab = expression(d[f]), ylab = "Outbreak Size")
plot(bpSEIR$Thresh100 ~ bpSEIR$d_f, main = expression(paste("Effect of ", d[f], " on Duration")),
    xlab = expression(d[f]), ylab = "Detectable Duration (days)")
```

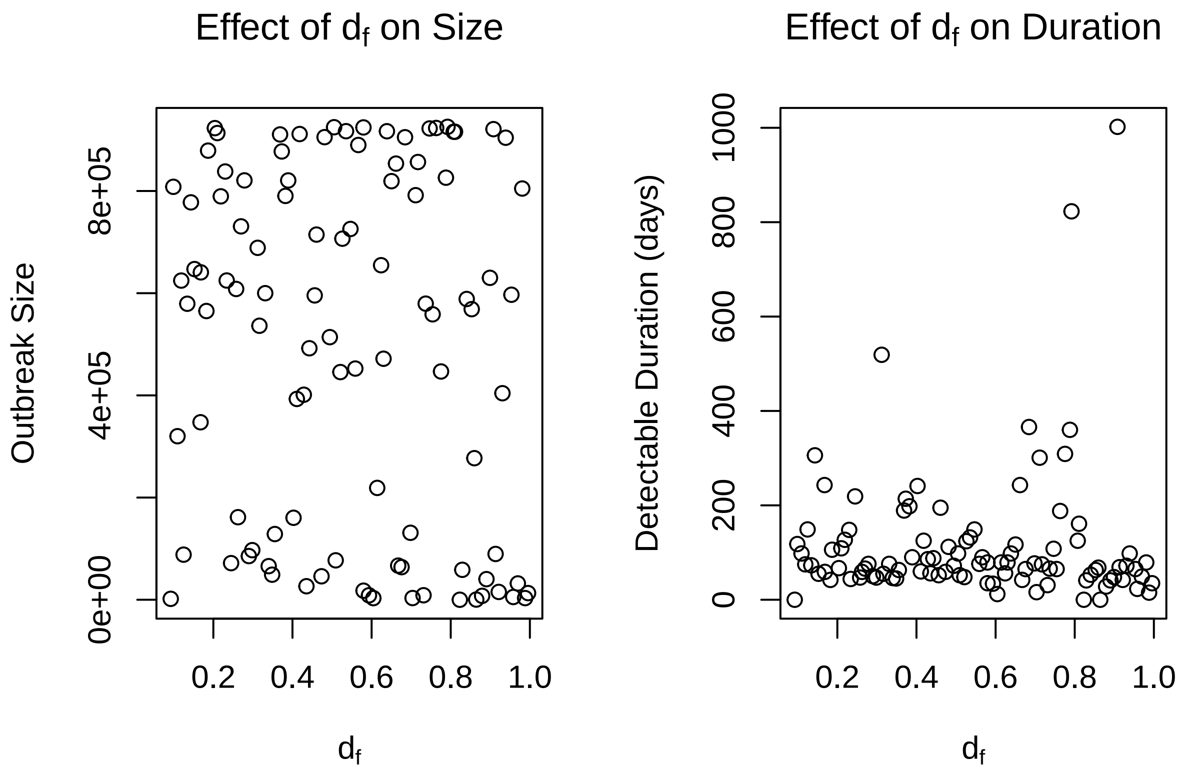


```
plot(bpSEIR$MaxInf ~ bpSEIR$beta_b, main = expression(paste("Effect of ", beta[b],
    " on Size")), xlab = expression(beta[b]), ylab = "Outbreak Size")
plot(bpSEIR$Thresh100 ~ bpSEIR$beta_b, main = expression(paste("Effect of ", beta[b],
    " on Duration")), xlab = expression(beta[b]), ylab = "Detectable Duration (days)")
```

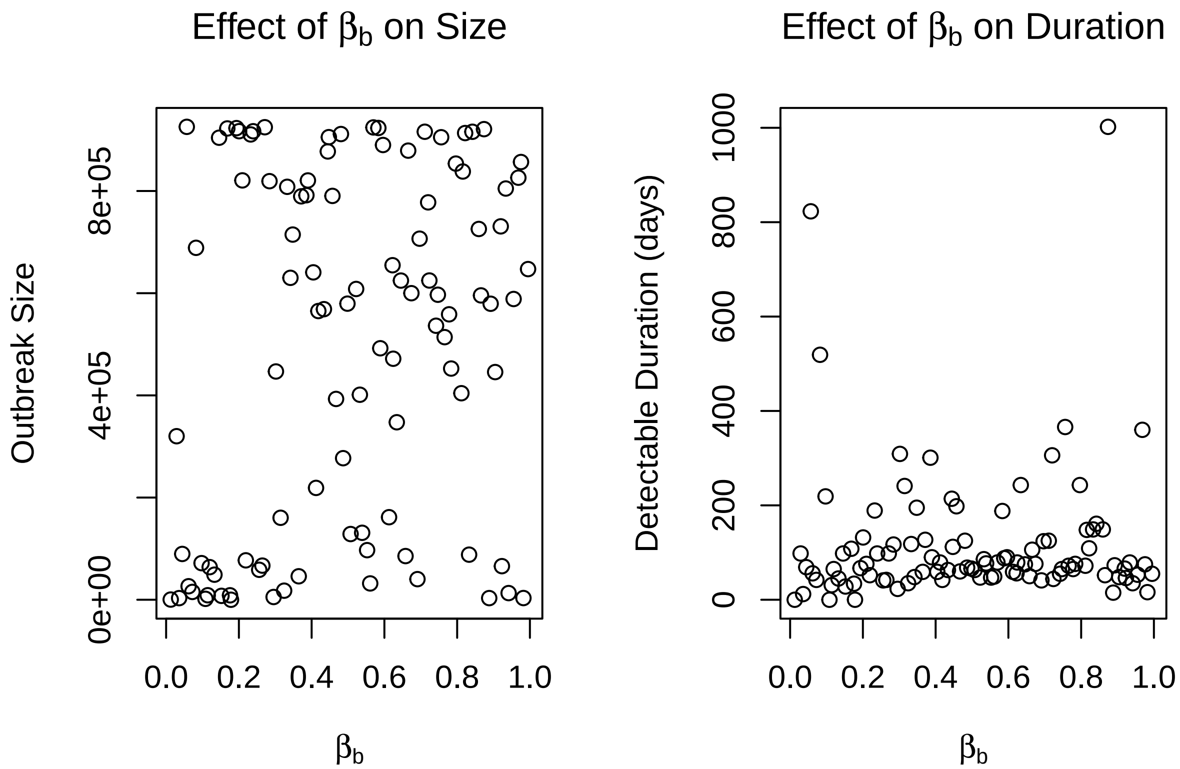


```r
plot(bpSEIR$MaxInf ~ bpSEIR$sigma_b, main = expression(paste("Effect of ", sigma[b],
    " on Size")), xlab = expression(sigma[b]), ylab = "Outbreak Size")
plot(bpSEIR$Thresh100 ~ bpSEIR$sigma_b, main = expression(paste("Effect of ", sigma[b],
    " on Duration")), xlab = expression(sigma[b]), ylab = "Detectable Duration (days)")
```

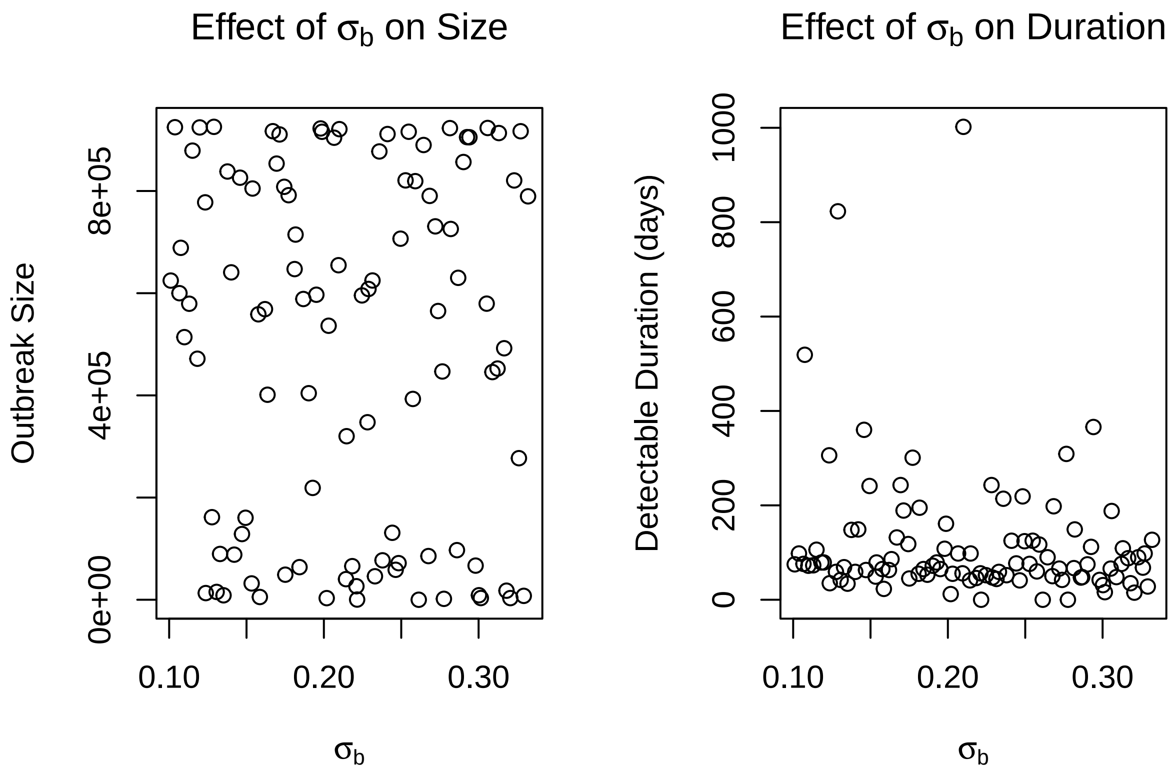


```
plot(bpSEIR$MaxInf ~ bpSEIR$gamma_b, main = expression(paste("Effect of ", gamma[b],
    " on Size")), xlab = expression(gamma[b]), ylab = "Outbreak Size")
plot(bpSEIR$Thresh100 ~ bpSEIR$gamma_b, main = expression(paste("Effect of ", gamma[b],
    " on Duration")), xlab = expression(gamma[b]), ylab = "Detectable Duration (days)")
```

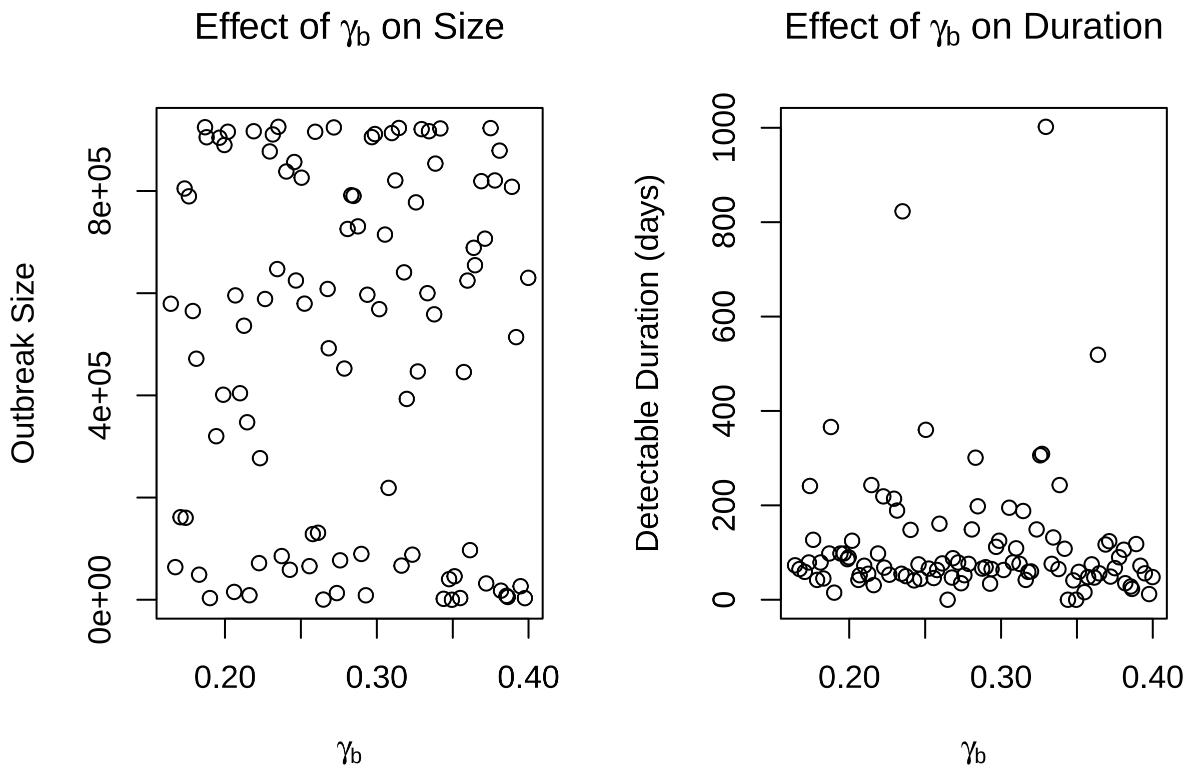


```
plot(bpSEIR$MaxInf ~ bpSEIR$beta_p, main = expression(paste("Effect of ", beta[p],
    " on Size")), xlab = expression(beta[p]), ylab = "Outbreak Size")
plot(bpSEIR$Thresh100 ~ bpSEIR$beta_p, main = expression(paste("Effect of ", beta[p],
    " on Duration")), xlab = expression(beta[p]), ylab = "Detectable Duration (days)")
```

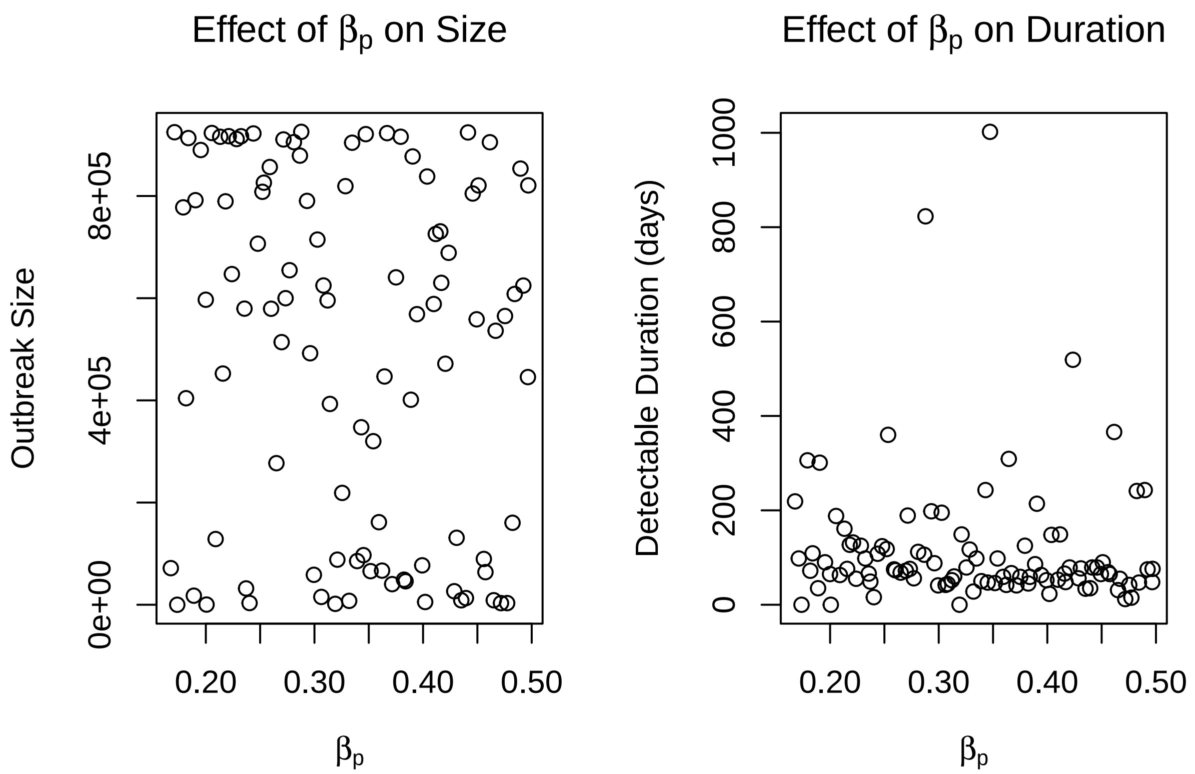


```
plot(bpSEIR$MaxInf ~ bpSEIR$sigma_p, main = expression(paste("Effect of ", sigma[p],
    " on Size")), xlab = expression(sigma[p]), ylab = "Outbreak Size")
plot(bpSEIR$Thresh100 ~ bpSEIR$sigma_p, main = expression(paste("Effect of ", sigma[p],
    " on Duration")), xlab = expression(sigma[p]), ylab = "Detectable Duration (days)")
```

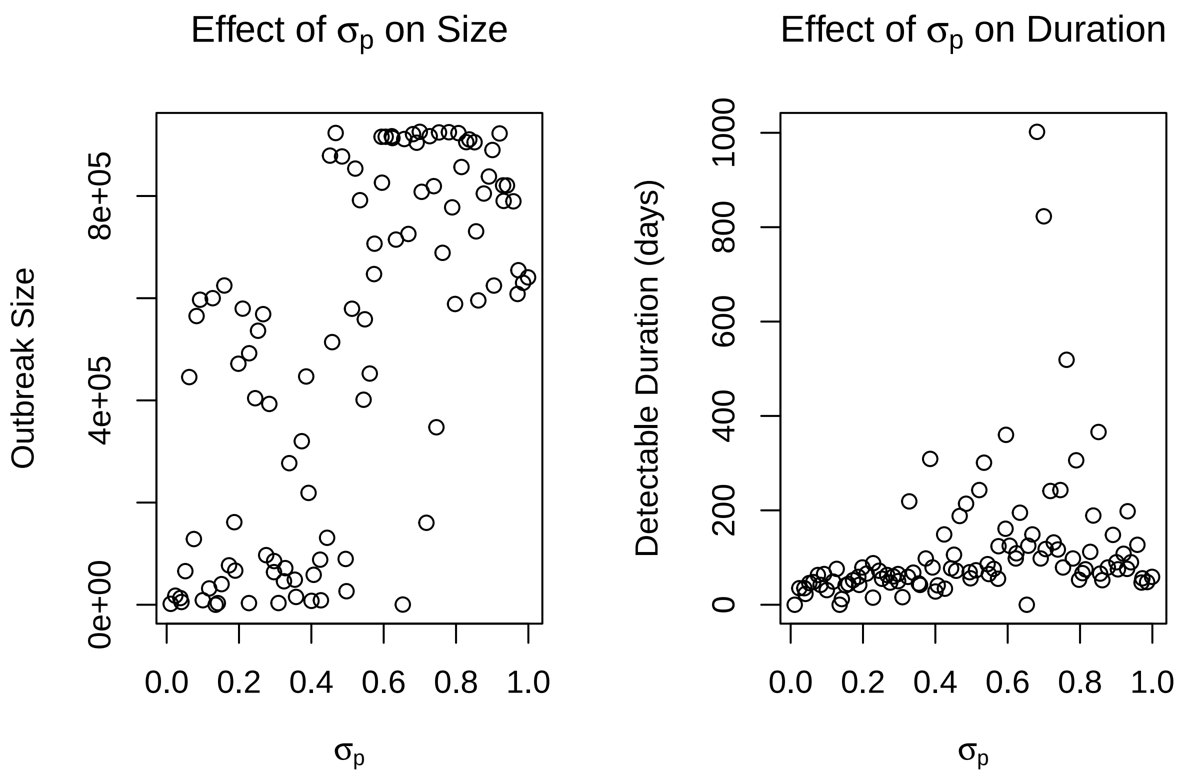


```
plot(bpSEIR$MaxInf ~ bpSEIR$gamma_p, main = expression(paste("Effect of ", gamma[p],
    " on Size")), xlab = expression(gamma[p]), ylab = "Outbreak Size")
plot(bpSEIR$Thresh100 ~ bpSEIR$gamma_p, main = expression(paste("Effect of ", gamma[p],
    " on Duration")), xlab = expression(gamma[p]), ylab = "Detectable Duration (days)")
```

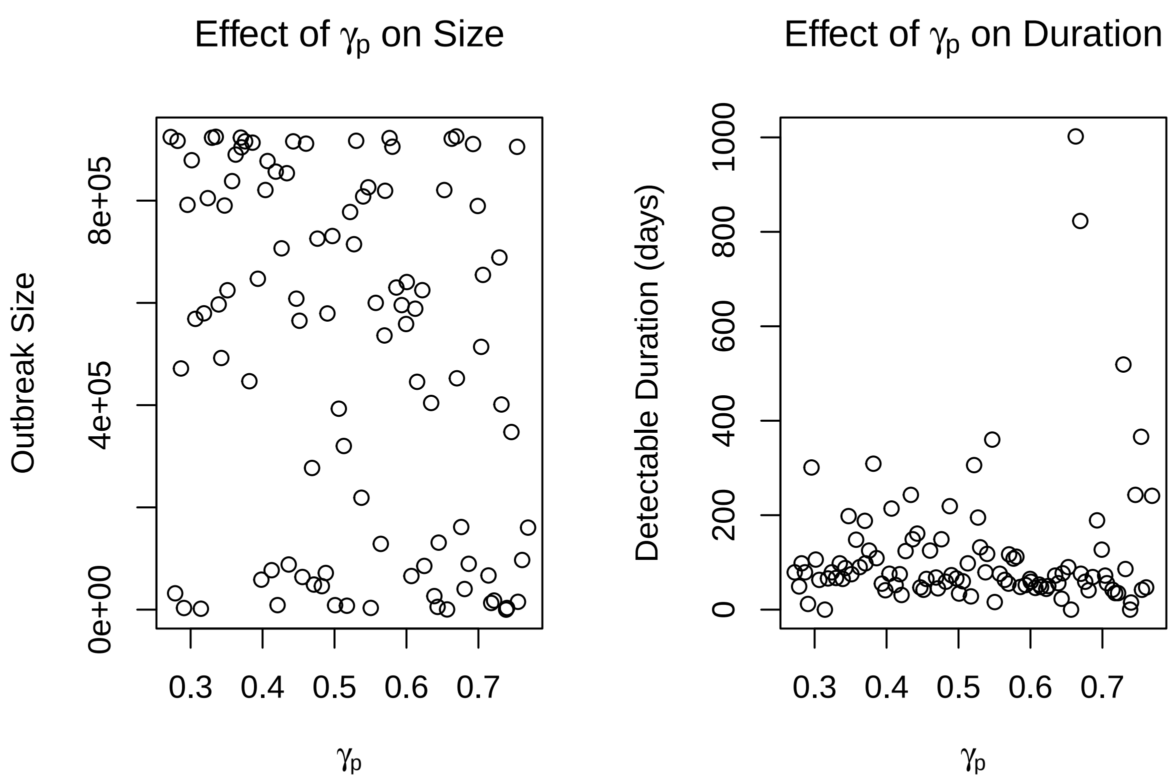


```
plot(bpSEIR$MaxInf ~ bpSEIR$g_h, main = expression(paste("Effect of ", g[h], " on Size")),
    xlab = expression(g[h]), ylab = "Outbreak Size")
plot(bpSEIR$Thresh100 ~ bpSEIR$g_h, main = expression(paste("Effect of ", g[h], " on Duration")),
    xlab = expression(g[h]), ylab = "Detectable Duration (days)")
```

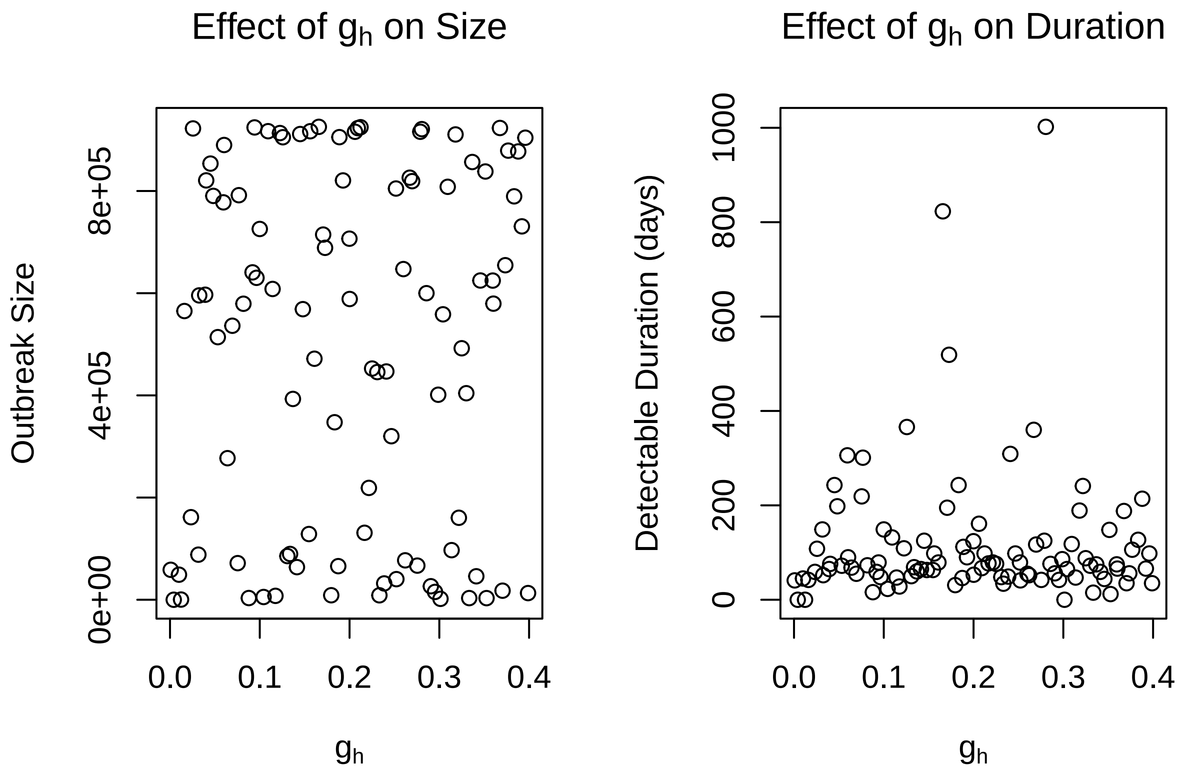


```
plot(bpSEIR$MaxInf ~ bpSEIR$p, main = expression(paste("Effect of ", p, " on Size")),
    xlab = "p", ylab = "Outbreak Size")
plot(bpSEIR$Thresh100 ~ bpSEIR$p, main = expression(paste("Effect of ", p, " on Duration")),
    xlab = "p", ylab = "Detectable Duration (days)")
```

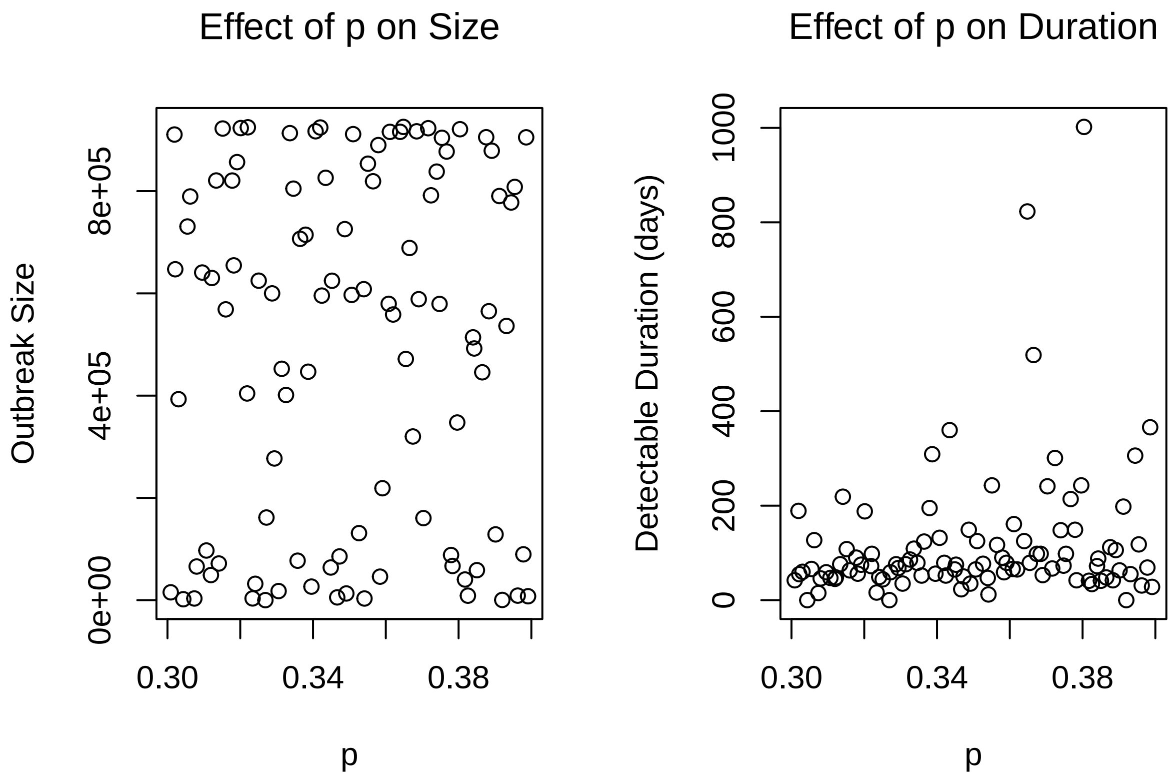


```
plot(bpSEIR$MaxInf ~ bpSEIR$b_h, main = expression(paste("Effect of ", b[h], " on Size")),
    xlab = expression(b[h]), ylab = "Outbreak Size")
plot(bpSEIR$Thresh100 ~ bpSEIR$b_h, main = expression(paste("Effect of ", b[h], " on Duration")),
    xlab = expression(b[h]), ylab = "Detectable Duration (days)")
```

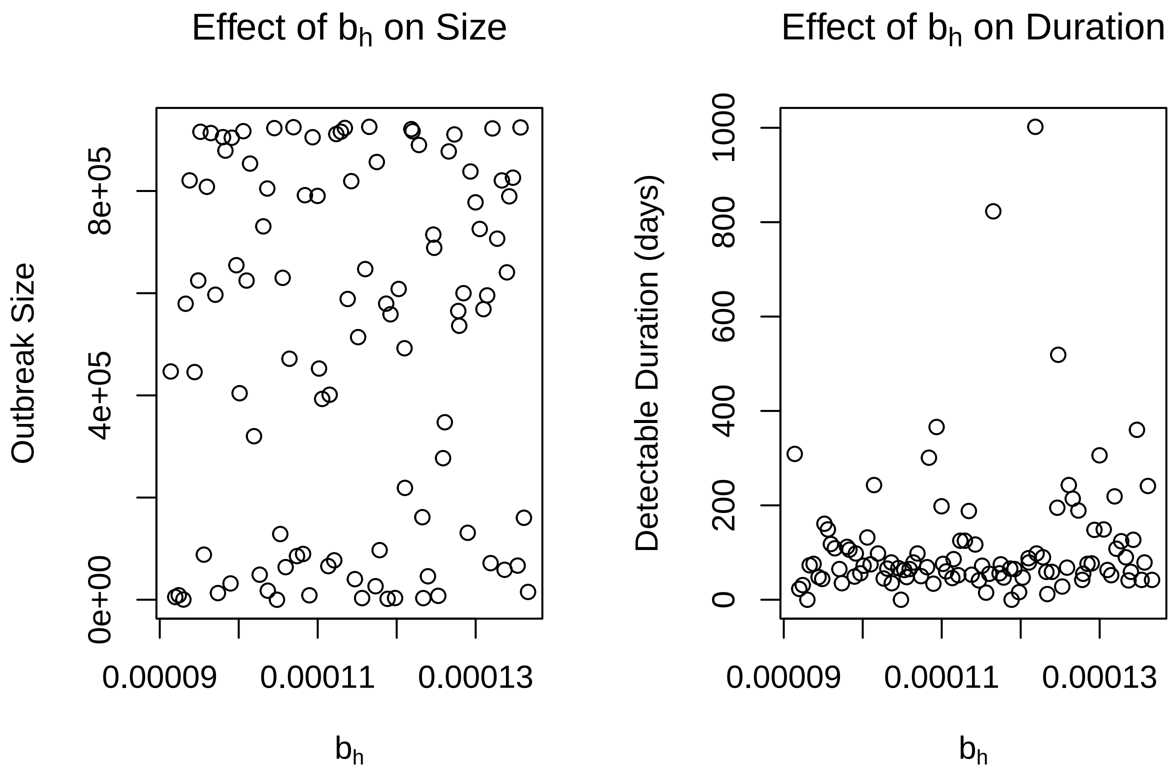


```
plot(bpSEIR$MaxInf ~ bpSEIR$d_h, main = expression(paste("Effect of ", d[h], " on Size")),
    xlab = expression(d[h]), ylab = "Outbreak Size")
plot(bpSEIR$Thresh100 ~ bpSEIR$d_h, main = expression(paste("Effect of ", d[h], " on Duration")),
    xlab = expression(d[h]), ylab = "Detectable Duration (days)")
```

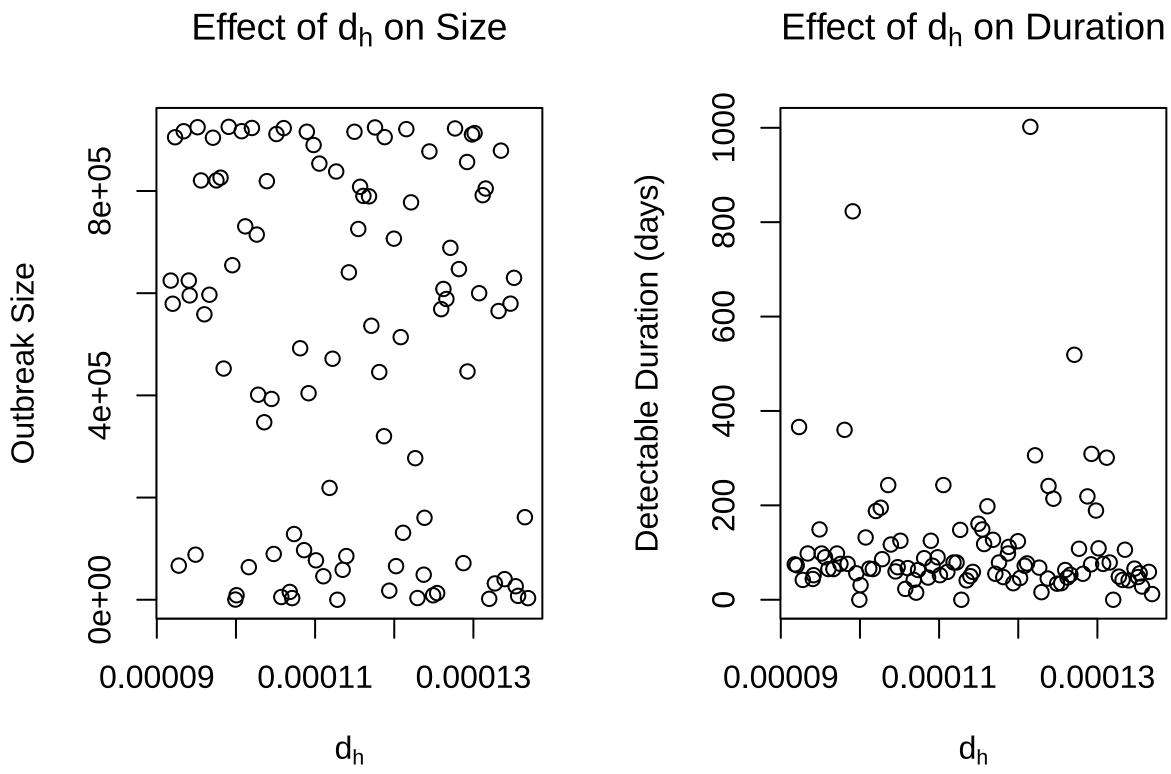


```
par(mfrow = c(1, 2))
boxplot(bpSEIR$MaxInf, main = "Outbreak Size", ylab = "Number of Dead Humans", ylim = c(0,
    923406))
boxplot(bpSEIR$Thresh100, main = "Outbreak Duration", ylab = "Time (Days)")
```

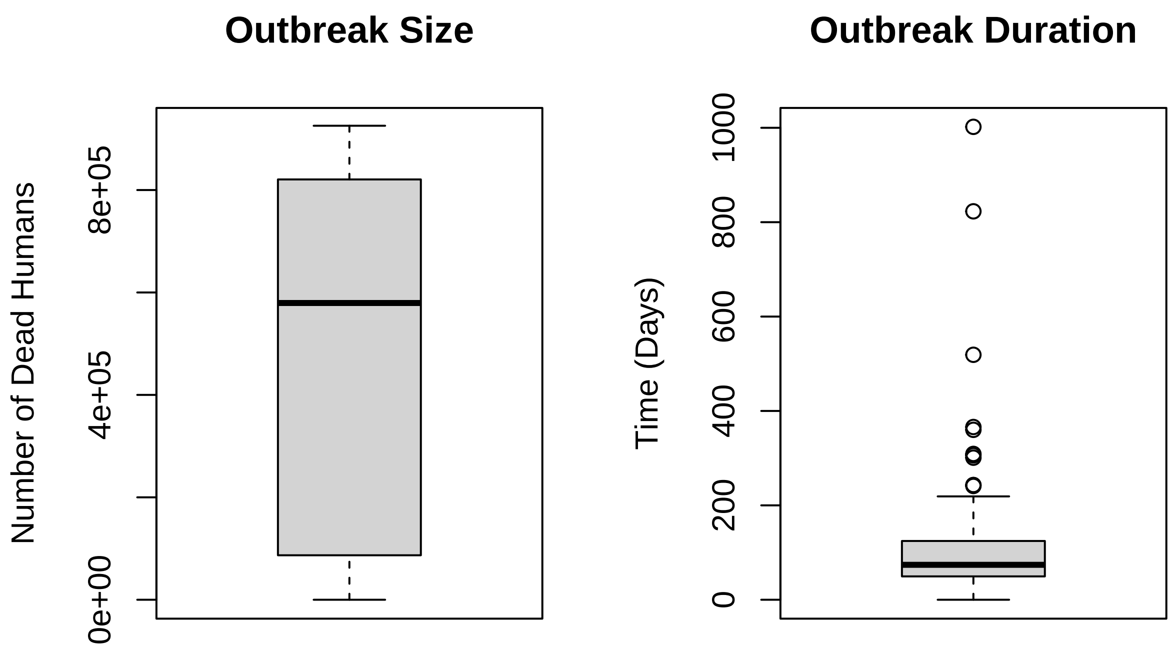


```
bonferroni.alpha <- 0.05/length(parameters)
prcc_size <- pcc(bpSEIR[, 1:length(parameters)], bpSEIR[, length(parameters) + 1],
    nboot = niter, rank = TRUE, conf = 1 - bonferroni.alpha)
prcc_duration <- pcc(bpSEIR[, 1:length(parameters)], bpSEIR[, length(parameters) +
    2], nboot = niter, rank = TRUE, conf = 1 - bonferroni.alpha)

# plot correlation coefficients and confidence intervals for epidemic size and
# duration
size <- prcc_size$PRCC
size$param <- rownames(size)
colnames(size)[4:5] <- c("maxCI", "minCI")
size$maxCI[which(size$maxCI > 1)] <- 1
size$maxCI[which(size$maxCI < -1)] <- -1
size$minCI[which(size$minCI > 1)] <- 1
size$minCI[which(size$minCI < -1)] <- -1

duration <- prcc_duration$PRCC
duration$param <- rownames(duration)
colnames(duration)[4:5] <- c("maxCI", "minCI")
duration$maxCI[which(duration$maxCI > 1)] <- 1
duration$maxCI[which(duration$maxCI < -1)] <- -1
duration$minCI[which(duration$minCI > 1)] <- 1
duration$minCI[which(duration$minCI < -1)] <- -1


A <- ggplot(size, aes(x = param, y = original)) + geom_point(size = 4) + geom_errorbar(aes(ymax = maxCI
```

```
    ymin = minCI)) + ggtitle("A") + xlab("Parameters") + ylab("Partial Rank Correlation Coefficients") +
    scale_x_discrete(labels = c(alpha = expression(alpha), beta_b = expression(beta[b]),
        beta_p = expression(beta[p]), beta_r = expression(beta[r]), b_h = expression(b[h]),
        d_h = expression(d[h]), d_f = expression(d[f]), sigma_b = expression(sigma[b]),
        gamma_b = expression(gamma[b]), sigma_p = expression(sigma[p]), gamma_p = expression(gamma[p]),
        gamma_r = expression(gamma[r]), g_h = expression(g[h]), g_r = expression(g[r]),
        K_f = expression(K[f]), r_f = expression(r[f]))) + ylim(-1, 1)


B <- ggplot(duration, aes(x = param, y = original)) + geom_point(size = 4) + geom_errorbar(aes(ymax = ma
    ymin = minCI)) + ggtitle("B") + xlab("Parameters") + ylab(" ") + scale_x_discrete(labels = c(alpha =
    beta_b = expression(beta[b]), beta_p = expression(beta[p]), beta_r = expression(beta[r]),
    b_h = expression(b[h]), d_h = expression(d[h]), d_f = expression(d[f]), sigma_b = expression(sigma[l
    gamma_b = expression(gamma[b]), sigma_p = expression(sigma[p]), gamma_p = expression(gamma[p]),
    gamma_r = expression(gamma[r]), g_h = expression(g[h]), g_r = expression(g[r]),
    K_f = expression(K[f]), r_f = expression(r[f]))) + ylim(-1, 1)

multiplot(A, B, cols = 2)
```

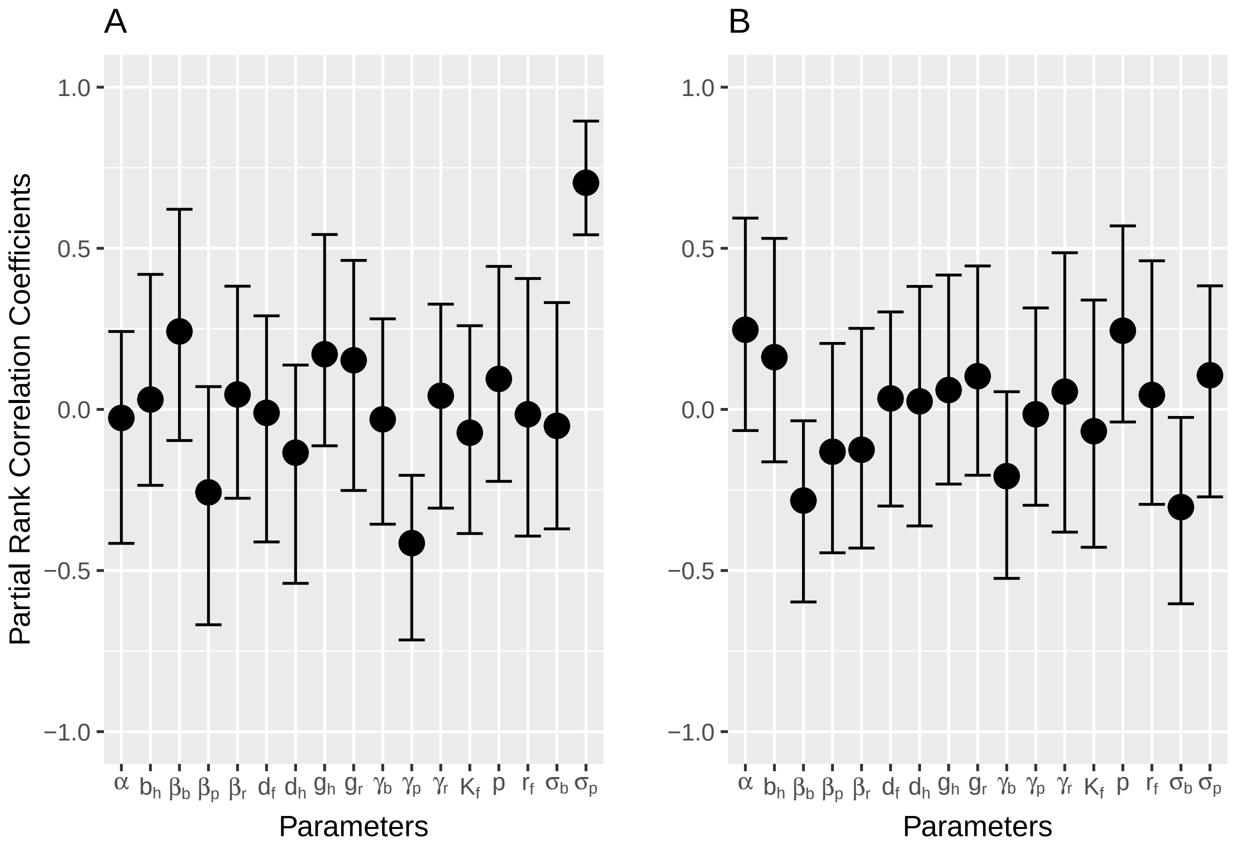


```
# tiff('FigureS17bpSEIR_PRCCuniform.tiff', height =8.7 , width =10, units =
# 'cm', compression = 'lzw', res = 1200) multiplot(A, B, cols=2) dev.off()
```

Smallpox SIR

```
parameters <- c(beta_s = 0.584, gamma_s = 1/9.5, g_s = 0.05, b_h = 1/(25 * 365),
    d_h = 1/(25 * 365))  #you can play with transmission and recovery rates here

# plot scatterplots
par(mfrow = c(1, 2))
plot(sSIR$MaxInf ~ sSIR$beta_s, main = expression(paste("Effect of ", beta[s], " on Size")),
    xlab = expression(beta[s]), ylab = "Outbreak Size")
plot(sSIR$Thresh100 ~ sSIR$beta_s, main = expression(paste("Effect of ", beta[s],
    " on Duration")), xlab = expression(beta[s]), ylab = "Observable Duration (days)")
```

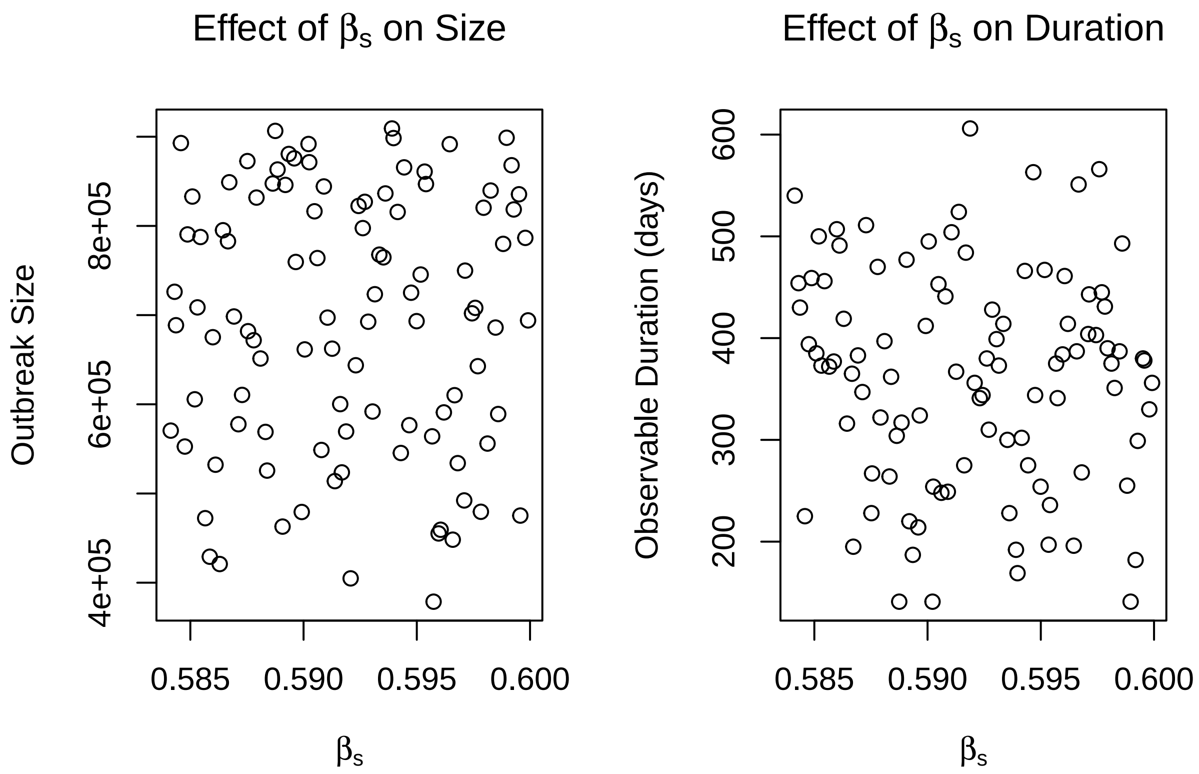


```
plot(sSIR$MaxInf ~ sSIR$gamma_s, main = expression(paste("Effect of ", gamma[s],
    " on Size")), xlab = expression(gamma[s]), ylab = "Outbreak Size")
plot(sSIR$Thresh100 ~ sSIR$gamma_s, main = expression(paste("Effect of ", gamma[s],
    " on Duration")), xlab = expression(gamma[s]), ylab = "Observable Duration (days)")
```

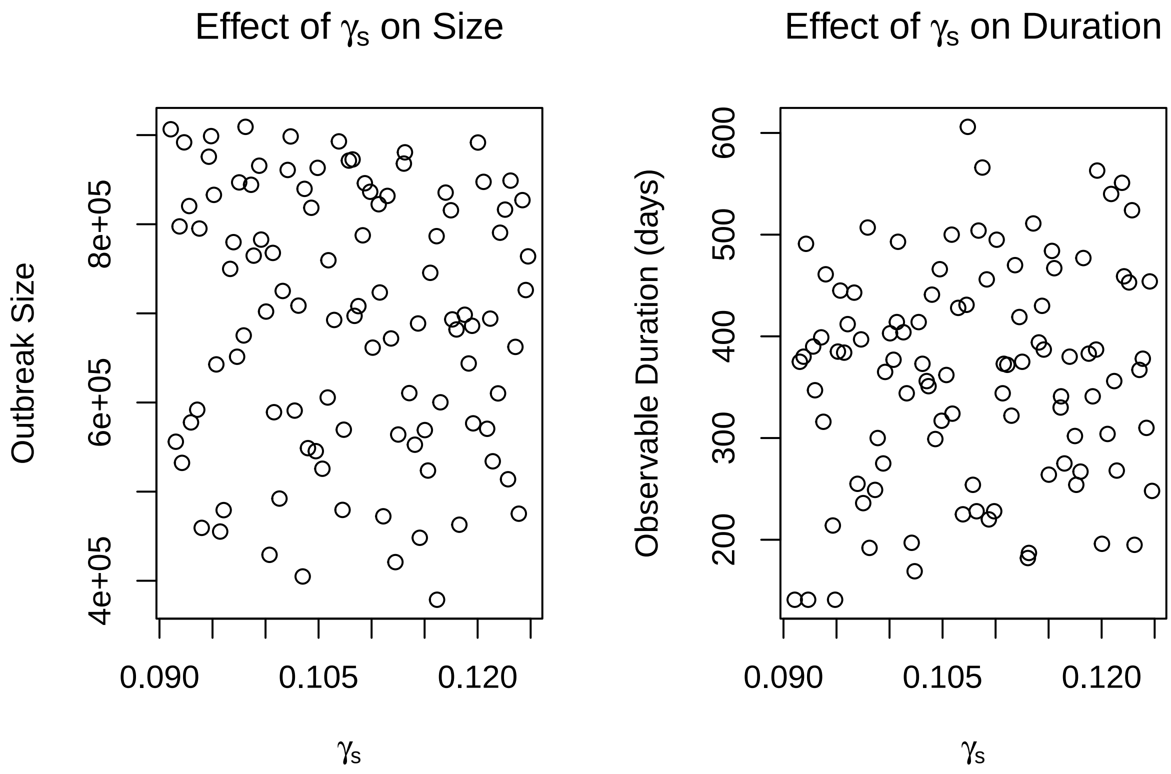


```
plot(sSIR$MaxInf ~ sSIR$g_s, main = expression(paste("Effect of ", g[s], " on Size")),
    xlab = expression(g[s]), ylab = "Outbreak Size")
plot(sSIR$Thresh100 ~ sSIR$g_s, main = expression(paste("Effect of ", g[s], " on Duration")),
    xlab = expression(g[s]), ylab = "Detectable Duration (days)")
```

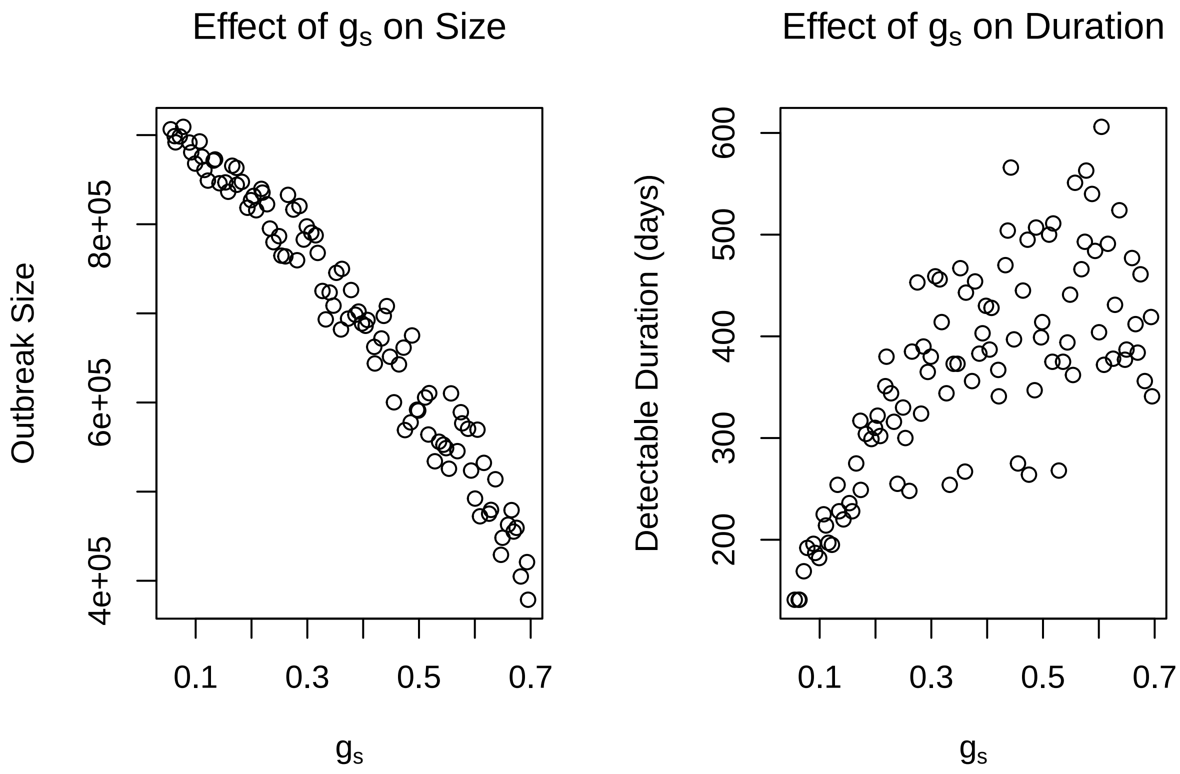


```
plot(sSIR$MaxInf ~ sSIR$b_h, main = expression(paste("Effect of ", b[h], " on Size")),
    xlab = expression(b[h]), ylab = "Outbreak Size")
plot(sSIR$Thresh100 ~ sSIR$b_h, main = expression(paste("Effect of ", b[h], " on Duration")),
    xlab = expression(b[h]), ylab = "Observable Duration (days)")
```

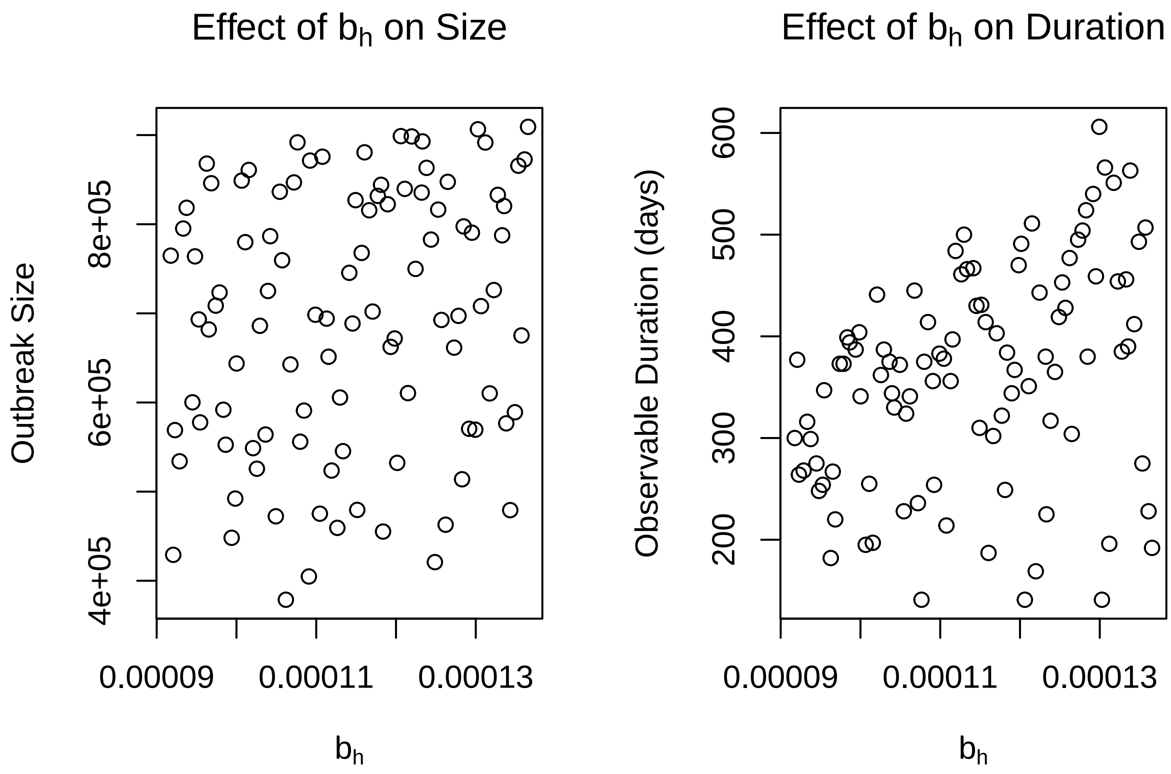


```
plot(sSIR$MaxInf ~ sSIR$d_h, main = expression(paste("Effect of ", d[h], " on Size")),
    xlab = expression(d[h]), ylab = "Outbreak Size")
plot(sSIR$Thresh100 ~ sSIR$d_h, main = expression(paste("Effect of ", d[h], " on Duration")),
    xlab = expression(d[h]), ylab = "Observable Duration (days)")
```

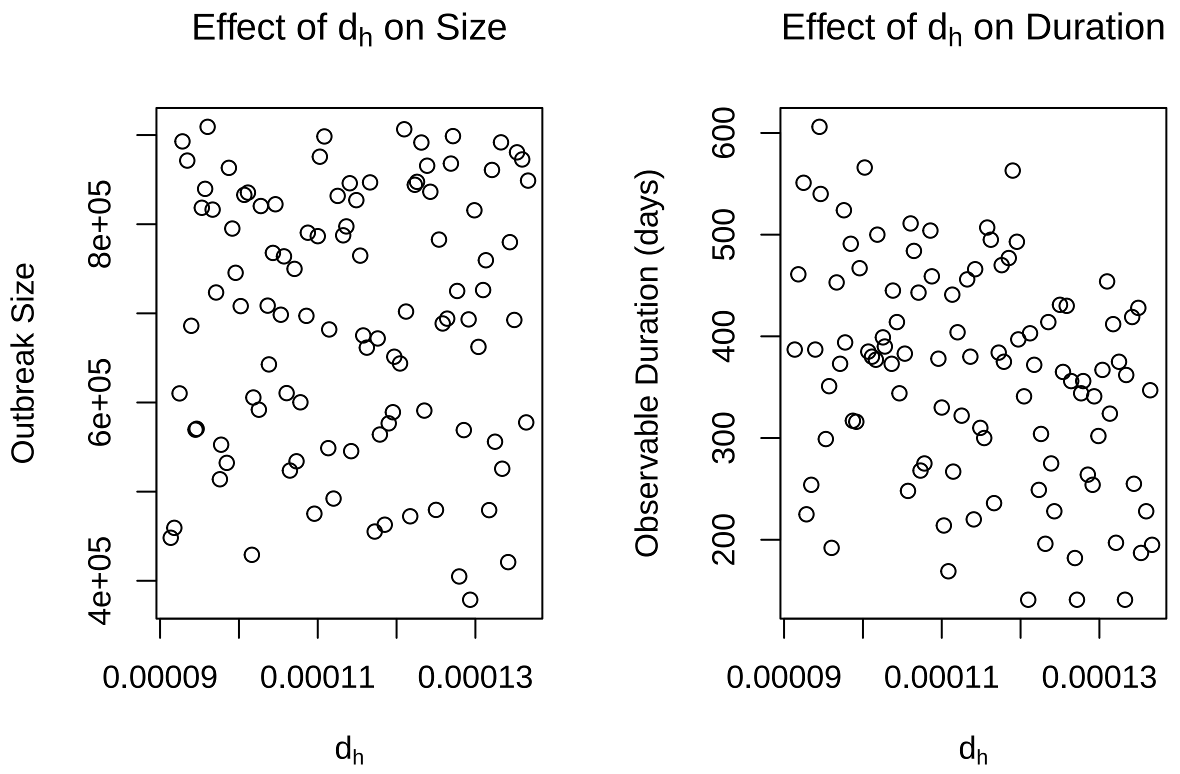


```
par(mfrow = c(1, 2))
boxplot(sSIR$MaxInf, main = "Outbreak Size", ylab = "Number of Dead Humans", ylim = c(0,
    923406))
boxplot(sSIR$Thresh100, main = "Observable Outbreak Duration", ylab = "Time (Days)")
```

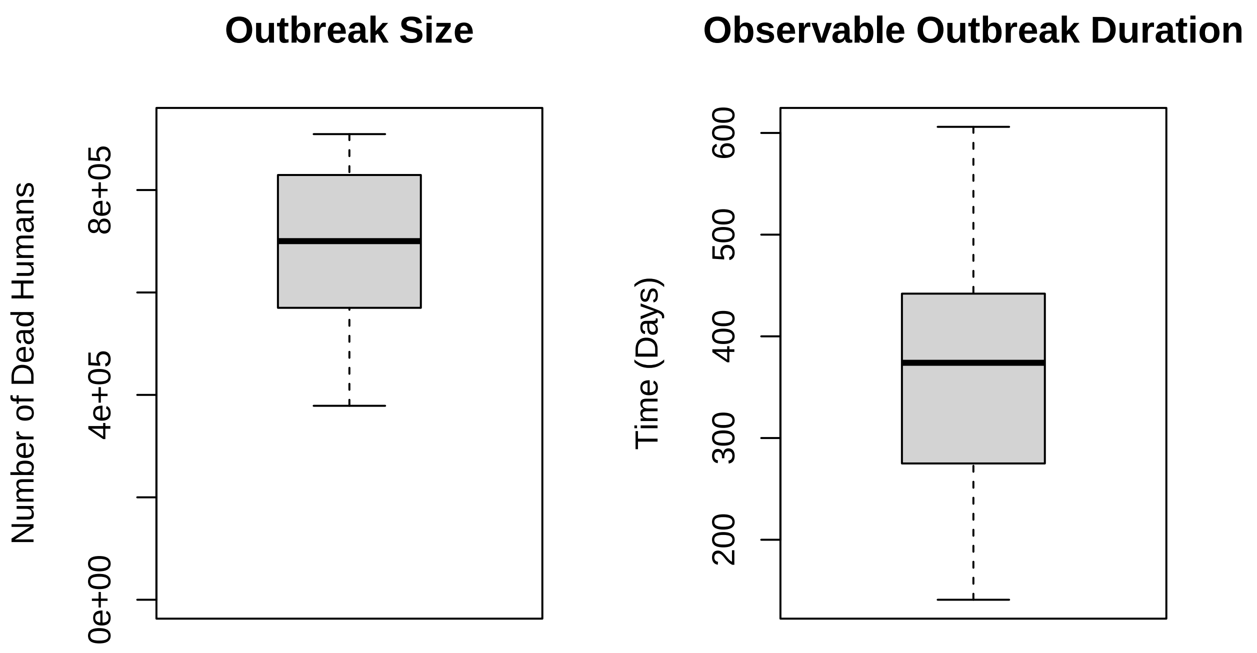


```
bonferroni.alpha <- 0.05/length(parameters)
prcc_size <- pcc(sSIR[, 1:length(parameters)], sSIR$MaxInf, nboot = niter, rank = TRUE,
    conf = 1 - bonferroni.alpha)
prcc_duration <- pcc(sSIR[, 1:length(parameters)], sSIR$Thresh100, nboot = niter,
    rank = TRUE, conf = 1 - bonferroni.alpha)

# plot correlation coefficients and confidence intervals for epidemic size and
# duration
size <- prcc_size$PRCC
size$param <- rownames(size)
colnames(size)[4:5] <- c("maxCI", "minCI")
size$maxCI[which(size$maxCI > 1)] <- 1
size$maxCI[which(size$maxCI < -1)] <- -1
size$minCI[which(size$minCI > 1)] <- 1
size$minCI[which(size$minCI < -1)] <- -1

duration <- prcc_duration$PRCC
duration$param <- rownames(duration)
colnames(duration)[4:5] <- c("maxCI", "minCI")
duration$maxCI[which(duration$maxCI > 1)] <- 1
duration$maxCI[which(duration$maxCI < -1)] <- -1
duration$minCI[which(duration$minCI > 1)] <- 1
duration$minCI[which(duration$minCI < -1)] <- -1


A <- ggplot(size, aes(x = param, y = original)) + geom_point(size = 4) + geom_errorbar(aes(ymax = maxCI
```

```
    ymin = minCI)) + ggtitle("A") + xlab("Parameters") + ylab("Partial Rank Correlation Coefficients") +
    scale_x_discrete(labels = c(beta_s = expression(beta[s]), b_h = expression(b[h]),
        d_h = expression(d[h]), gamma_s = expression(gamma[s]), g_s = expression(g[s]))) +
    ylim(-1, 1)

B <- ggplot(duration, aes(x = param, y = original)) + geom_point(size = 4) + geom_errorbar(aes(ymax = ma
    ymin = minCI)) + ggtitle("B") + xlab("Parameters") + ylab(" ") + scale_x_discrete(labels = c(beta_s
    b_h = expression(b[h]), d_h = expression(d[h]), gamma_s = expression(gamma[s]),
    g_s = expression(g[s]))) + ylim(-1, 1)

multiplot(A, B, cols = 2)
```

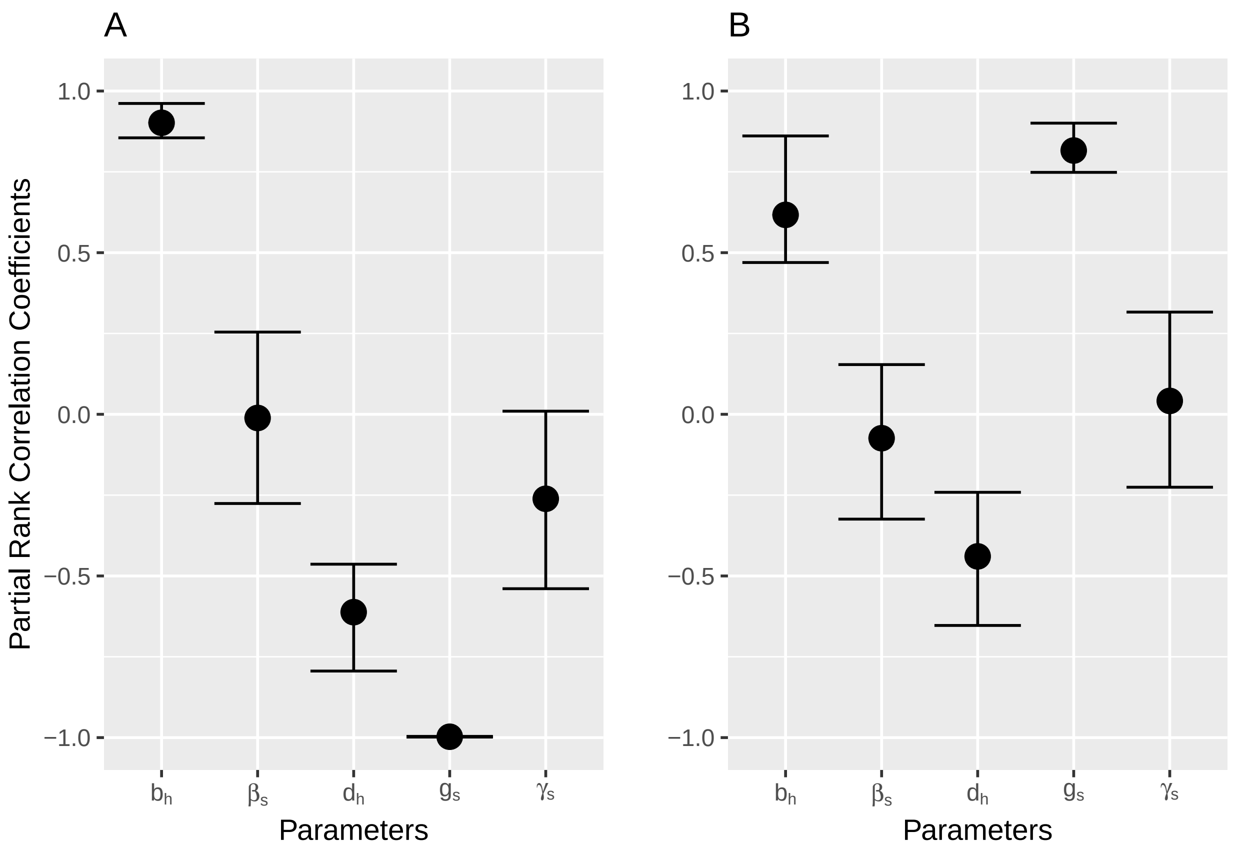


```
# tiff('FigureS18SmallpoxSIR_PRCCuniform.tiff', height =8.7 , width =10, units
# = 'cm', compression = 'lzw', res = 1200) multiplot(A, B, cols=2) dev.off()
```

Smallpox SEIR

```
parameters <- c(beta_s = 0.584, sigma_s = 1/12, gamma_s = 1/9.5, g_s = 0.05, b_h = 1/(25 *
    365), d_h = 1/(25 * 365))  #you can play with transmission and recovery rates here

# plot scatterplots
par(mfrow = c(1, 2))
plot(sSEIR$MaxInf ~ sSEIR$beta_s, main = expression(paste("Effect of ", beta[s],
```

```
    " on Size")), xlab = expression(beta[s]), ylab = "Outbreak Size")
plot(sSEIR$Thresh100 ~ sSEIR$beta_s, main = expression(paste("Effect of ", beta[s],
    " on Duration")), xlab = expression(beta[s]), ylab = "Observable Duration (days)")
```

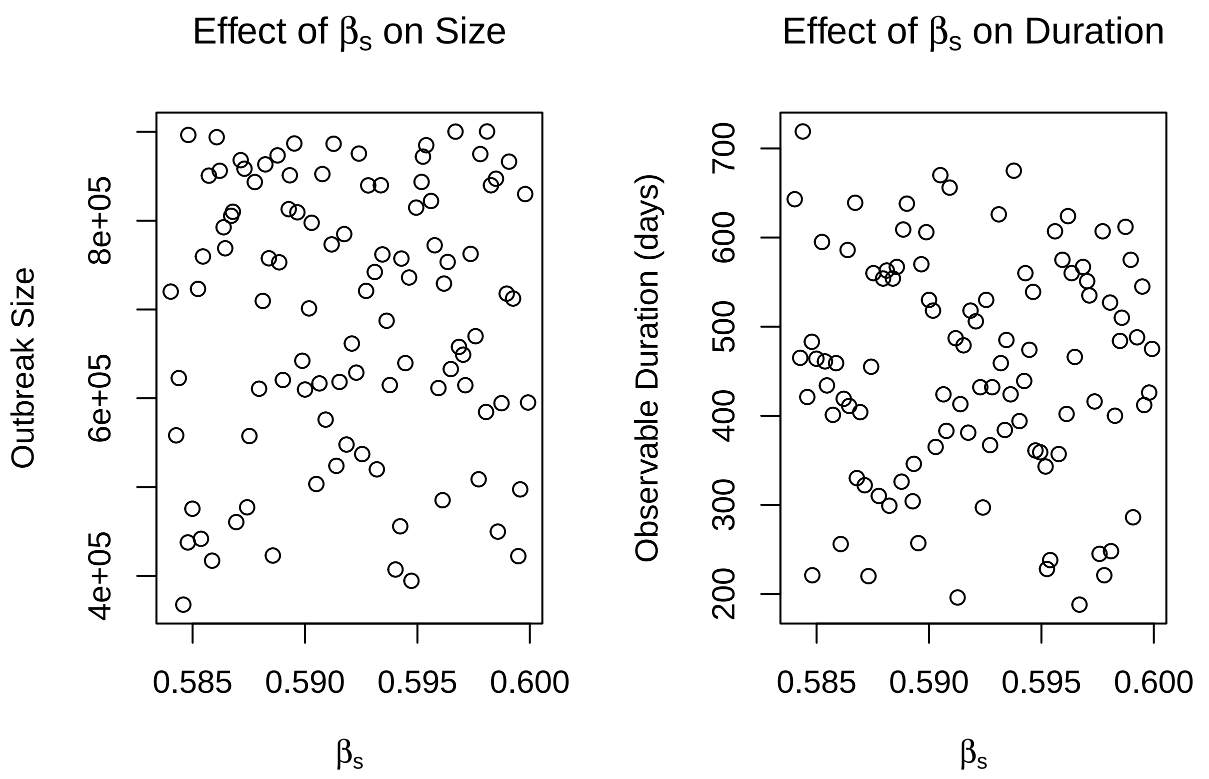


```
plot(sSEIR$MaxInf ~ sSEIR$sigma_s, main = expression(paste("Effect of ", sigma[s],
    " on Size")), xlab = expression(sigma[s]), ylab = "Outbreak Size")
plot(sSEIR$Thresh100 ~ sSEIR$sigma_s, main = expression(paste("Effect of ", sigma[s],
    " on Duration")), xlab = expression(sigma[s]), ylab = "Detectable Outbreak Duration (days)")
```

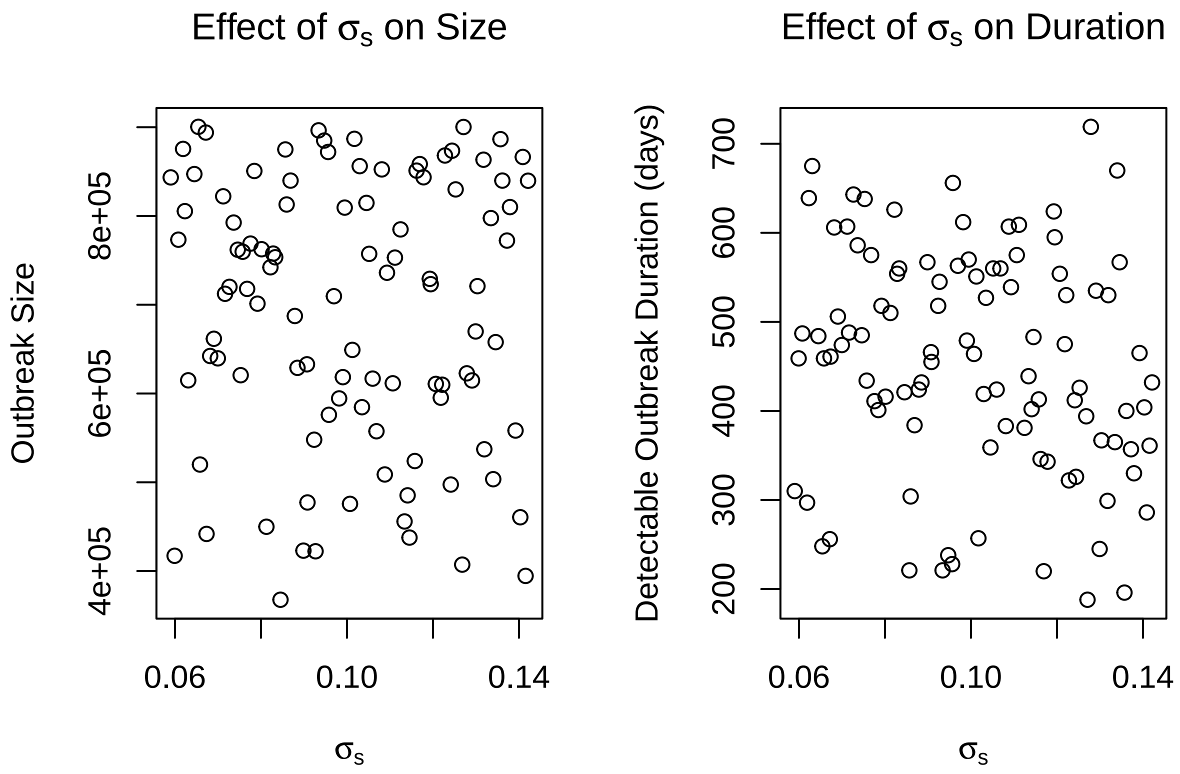


```
plot(sSEIR$MaxInf ~ sSEIR$gamma_s, main = expression(paste("Effect of ", gamma[s],
    " on Size")), xlab = expression(gamma[s]), ylab = "Outbreak Size")
plot(sSEIR$Thresh100 ~ sSEIR$gamma_s, main = expression(paste("Effect of ", gamma[s],
    " on Duration")), xlab = expression(gamma[s]), ylab = "Observable Duration (days)")
```

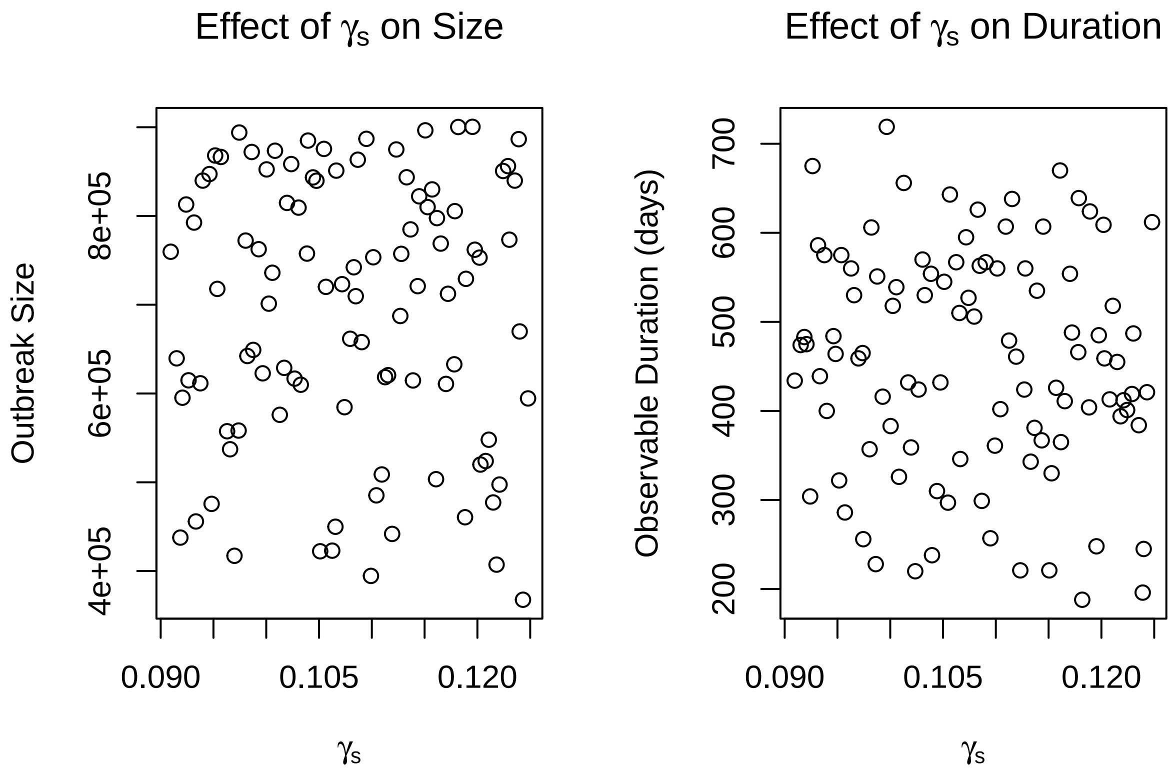


```
plot(sSEIR$MaxInf ~ sSEIR$g_s, main = expression(paste("Effect of ", g[s], " on Size")),
    xlab = expression(g[s]), ylab = "Outbreak Size")
plot(sSEIR$Thresh100 ~ sSEIR$g_s, main = expression(paste("Effect of ", g[s], " on Duration")),
    xlab = expression(g[s]), ylab = "Detectable Duration (days)")
```

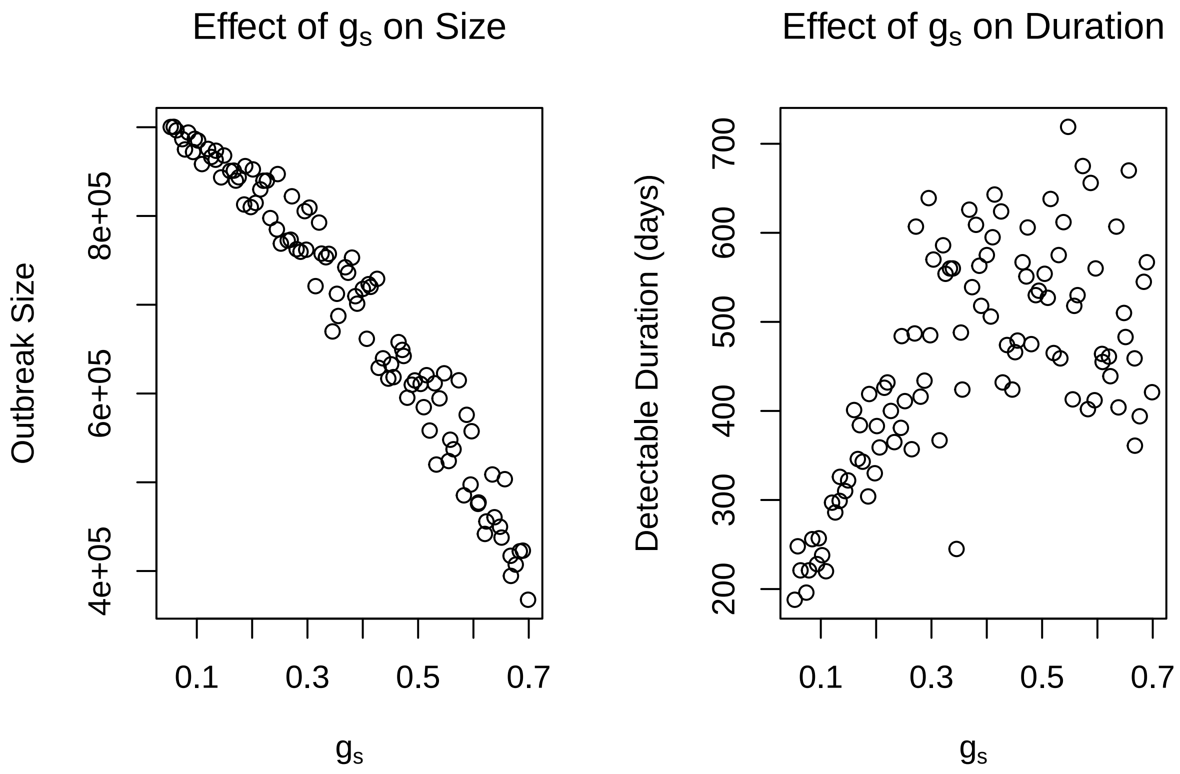


```
plot(sSEIR$MaxInf ~ sSEIR$b_h, main = expression(paste("Effect of ", b[h], " on Size")),
    xlab = expression(b[h]), ylab = "Outbreak Size")
plot(sSEIR$Thresh100 ~ sSEIR$b_h, main = expression(paste("Effect of ", b[h], " on Duration")),
    xlab = expression(b[h]), ylab = "Observable Duration (days)")
```

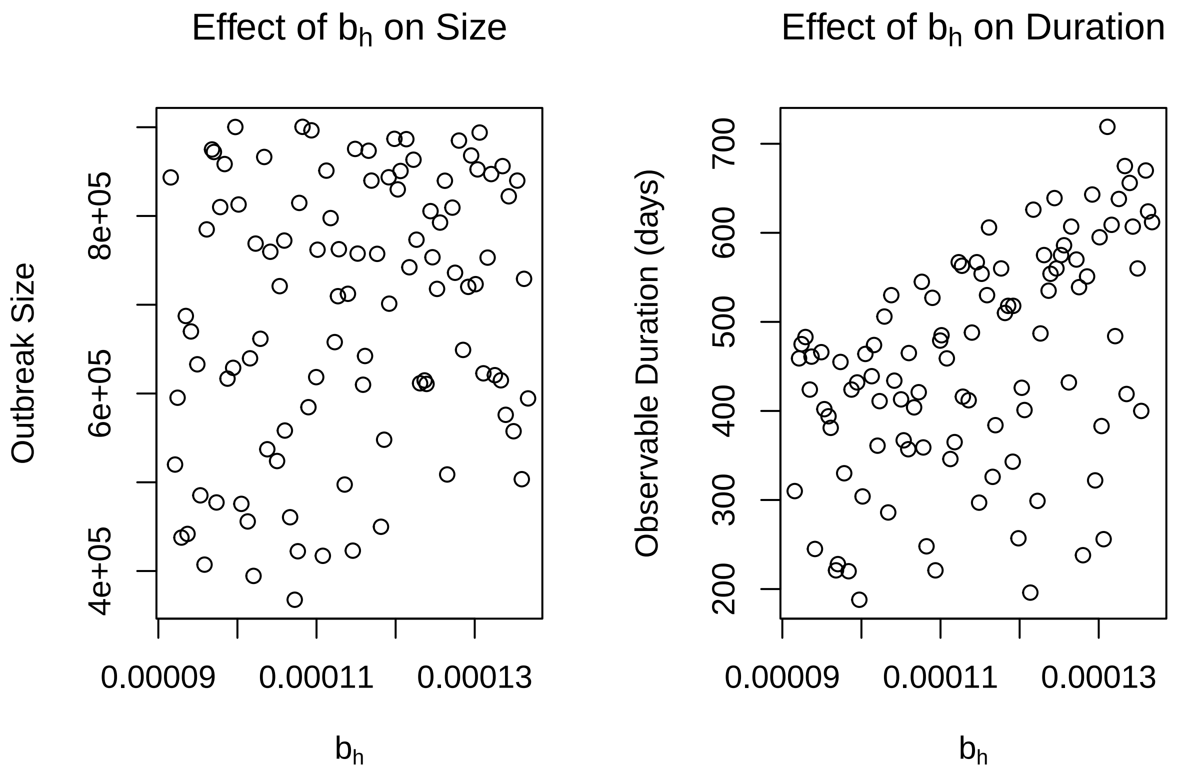


```
plot(sSEIR$MaxInf ~ sSEIR$d_h, main = expression(paste("Effect of ", d[h], " on Size")),
    xlab = expression(d[h]), ylab = "Outbreak Size")
plot(sSEIR$Thresh100 ~ sSEIR$d_h, main = expression(paste("Effect of ", d[h], " on Duration")),
    xlab = expression(d[h]), ylab = "Observable Duration (days)")
```

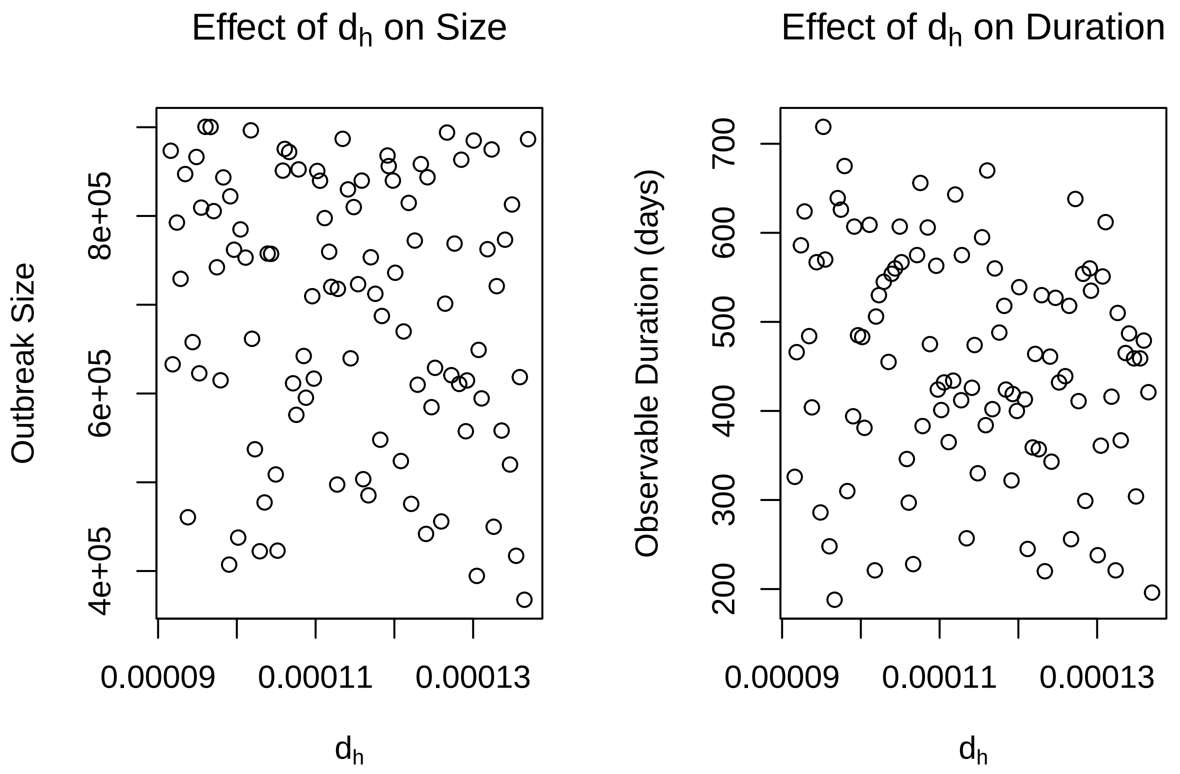


```
par(mfrow = c(1, 2))
boxplot(sSEIR$MaxInf, main = "Outbreak Size", ylab = "Number of Dead Humans", ylim = c(0,
    923406))
boxplot(sSEIR$Thresh100, main = "Outbreak Duration", ylab = "Time (Days)")
```

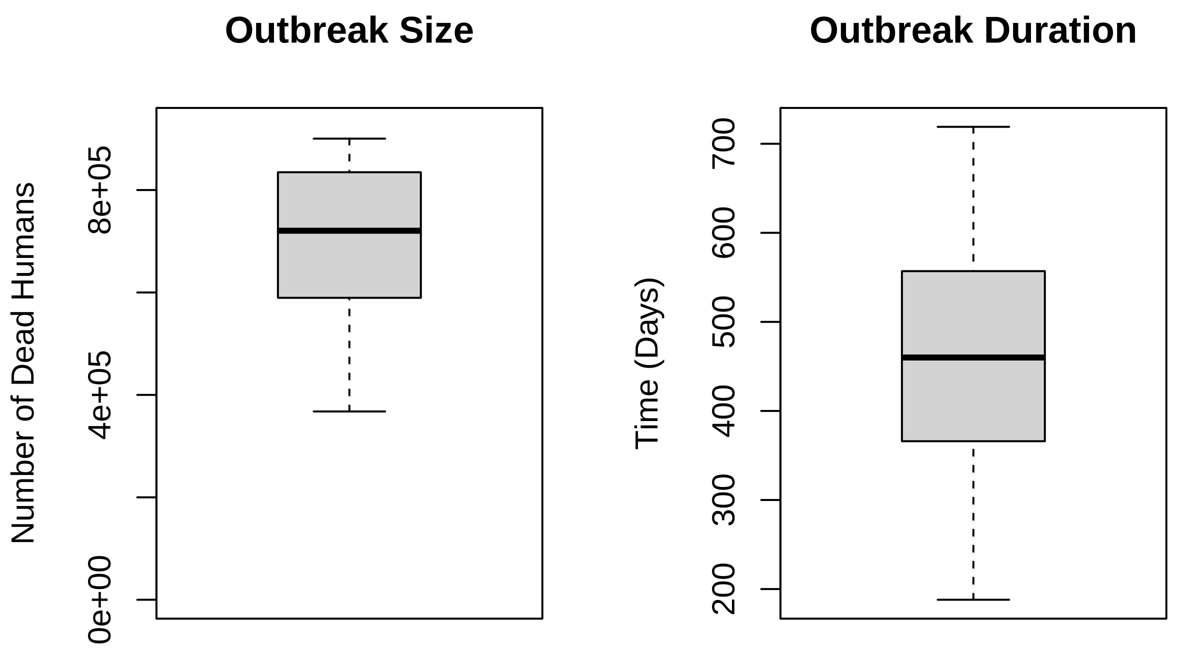


```
bonferroni.alpha <- 0.05/length(parameters)
prcc_size <- pcc(sSEIR[, 1:length(parameters)], sSEIR$MaxInf, nboot = niter, rank = TRUE,
    conf = 1 - bonferroni.alpha)
prcc_duration <- pcc(sSEIR[, 1:length(parameters)], sSEIR$Thresh100, nboot = niter,
    rank = TRUE, conf = 1 - bonferroni.alpha)

# plot correlation coefficients and confidence intervals for epidemic size and
# duration
size <- prcc_size$PRCC
size$param <- rownames(size)
colnames(size)[4:5] <- c("maxCI", "minCI")
size$maxCI[which(size$maxCI > 1)] <- 1
size$maxCI[which(size$maxCI < -1)] <- -1
size$minCI[which(size$minCI > 1)] <- 1
size$minCI[which(size$minCI < -1)] <- -1

duration <- prcc_duration$PRCC
duration$param <- rownames(duration)
colnames(duration)[4:5] <- c("maxCI", "minCI")
duration$maxCI[which(duration$maxCI > 1)] <- 1
duration$maxCI[which(duration$maxCI < -1)] <- -1
duration$minCI[which(duration$minCI > 1)] <- 1
duration$minCI[which(duration$minCI < -1)] <- -1

A <- ggplot(size, aes(x = param, y = original)) + geom_point(size = 4) + geom_errorbar(aes(ymax = maxCI
    ymin = minCI)) + ggtitle("A") + xlab("Parameters") + ylab("Partial Rank Correlation Coefficients") +
```

```
    scale_x_discrete(labels = c(beta_s = expression(beta[s]), b_h = expression(b[h]),
        d_h = expression(d[h]), gamma_s = expression(gamma[s]), g_s = expression(g[s]),
        sigma_s = expression(sigma[s]))) + ylim(-1, 1)

B <- ggplot(duration, aes(x = param, y = original)) + geom_point(size = 4) + geom_errorbar(aes(ymax = ma
    ymin = minCI)) + ggtitle("B") + xlab("Parameters") + ylab(" ") + scale_x_discrete(labels = c(beta_s
    b_h = expression(b[h]), d_h = expression(d[h]), gamma_s = expression(gamma[s]),
    g_s = expression(g[s]), sigma_s = expression(sigma[s]))) + ylim(-1, 1)


multiplot(A, B, cols = 2)
```

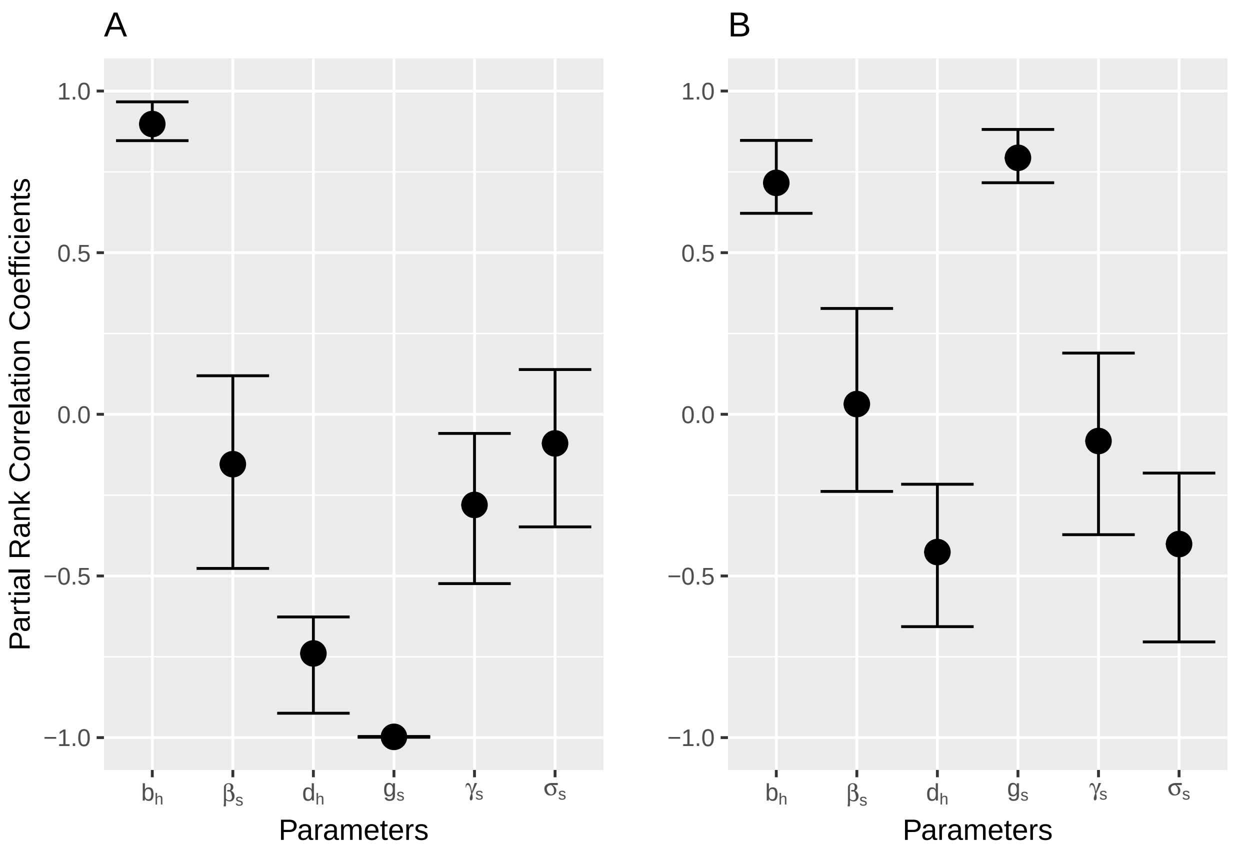


```
# tiff('FigureS19SmallpoxSEIR_PRCCuniform.tiff', height =8.7 , width =10, units
# = 'cm', compression = 'lzw', res = 1200) multiplot(A, B, cols=2) dev.off()
```

Measles SIR

```
parameters <- c(beta_m = 1.175, gamma_m = 1/13, g_m = 0.7, b_h = 1/(25 * 365), d_h = 1/(25 *
    365))  #you can play with transmission and recovery rates here

# plot scatterplots
par(mfrow = c(1, 2))
plot(mSIR$MaxInf ~ mSIR$beta_m, main = expression(paste("Effect of ", beta[m], " on Size")),
```

```
    xlab = expression(beta[m]), ylab = "Outbreak Size")
plot(mSIR$Thresh100 ~ mSIR$beta_m, main = expression(paste("Effect of ", beta[m],
    " on Duration")), xlab = expression(beta[m]), ylab = "Observable Duration (days)")
```

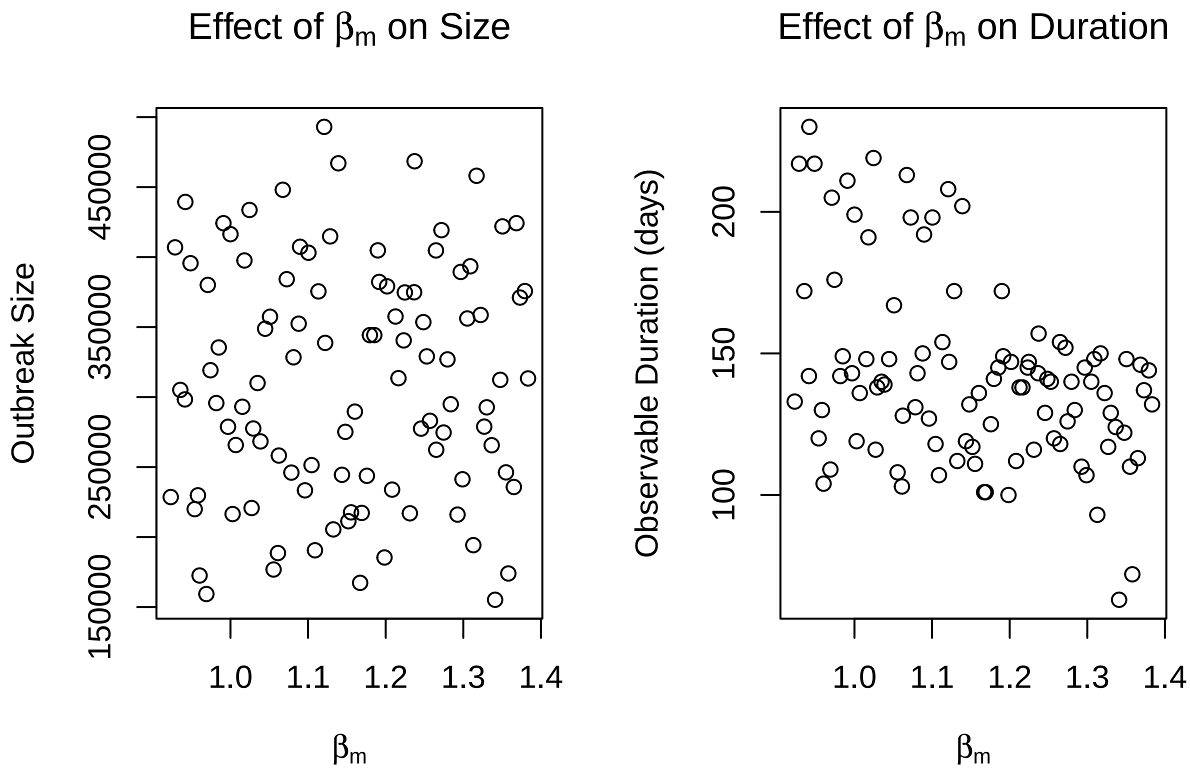


```
plot(mSIR$MaxInf ~ mSIR$gamma_m, main = expression(paste("Effect of ", gamma[m],
    " on Size")), xlab = expression(gamma[m]), ylab = "Outbreak Size")
plot(mSIR$Thresh100 ~ mSIR$gamma_m, main = expression(paste("Effect of ", gamma[m],
    " on Duration")), xlab = expression(gamma[m]), ylab = "Observable Duration (days)")
```

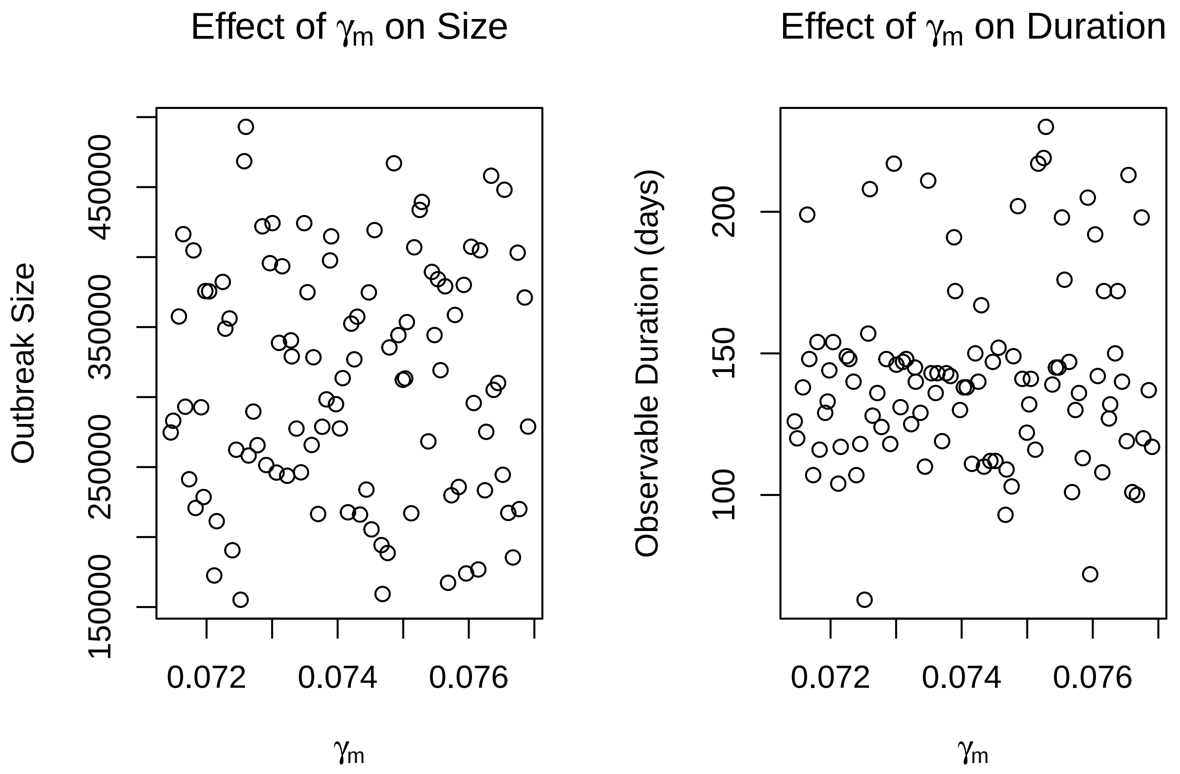


```
plot(mSIR$MaxInf ~ mSIR$g_m, main = expression(paste("Effect of ", g[m], " on Size")),
    xlab = expression(g[m]), ylab = "Outbreak Size")
plot(mSIR$Thresh100 ~ mSIR$g_m, main = expression(paste("Effect of ", g[m], " on Duration")),
    xlab = expression(g[m]), ylab = "Detectable Duration (days)")
```

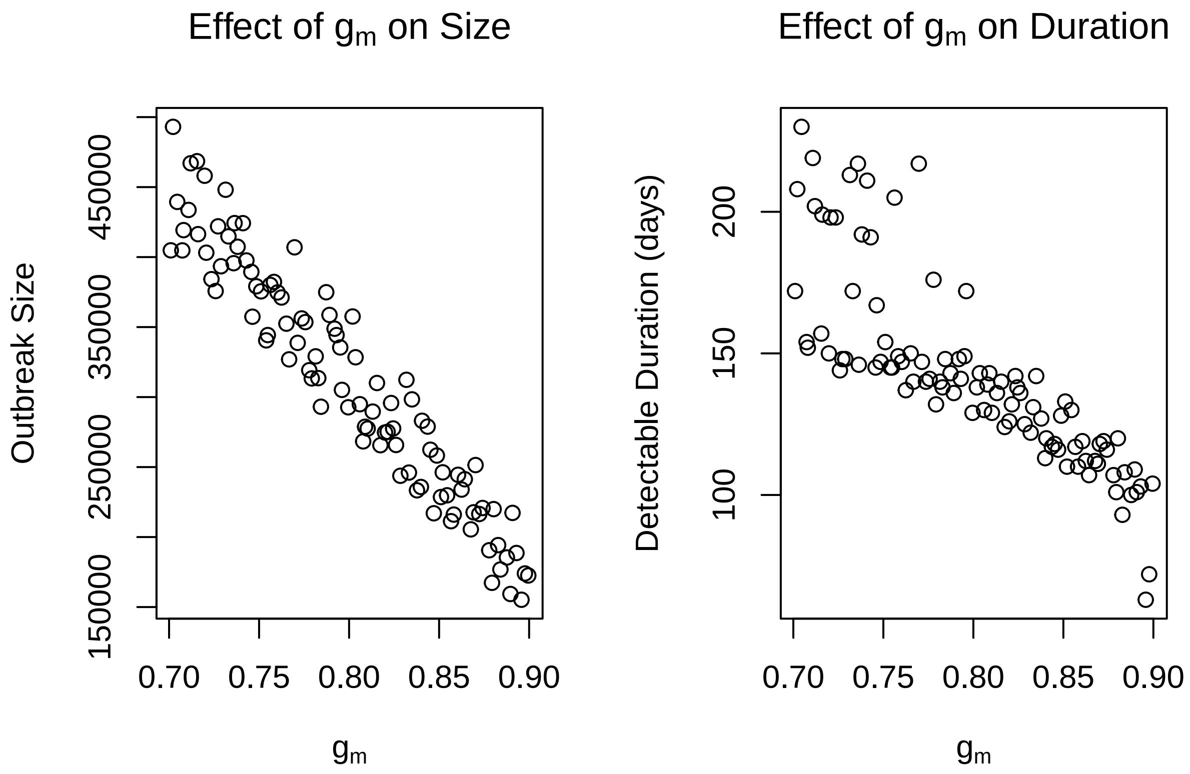


```
plot(mSIR$MaxInf ~ mSIR$b_h, main = expression(paste("Effect of ", b[h], " on Size")),
    xlab = expression(b[h]), ylab = "Outbreak Size")
plot(mSIR$Thresh100 ~ mSIR$b_h, main = expression(paste("Effect of ", b[h], " on Duration")),
    xlab = expression(b[h]), ylab = "Observable Duration (days)")
```

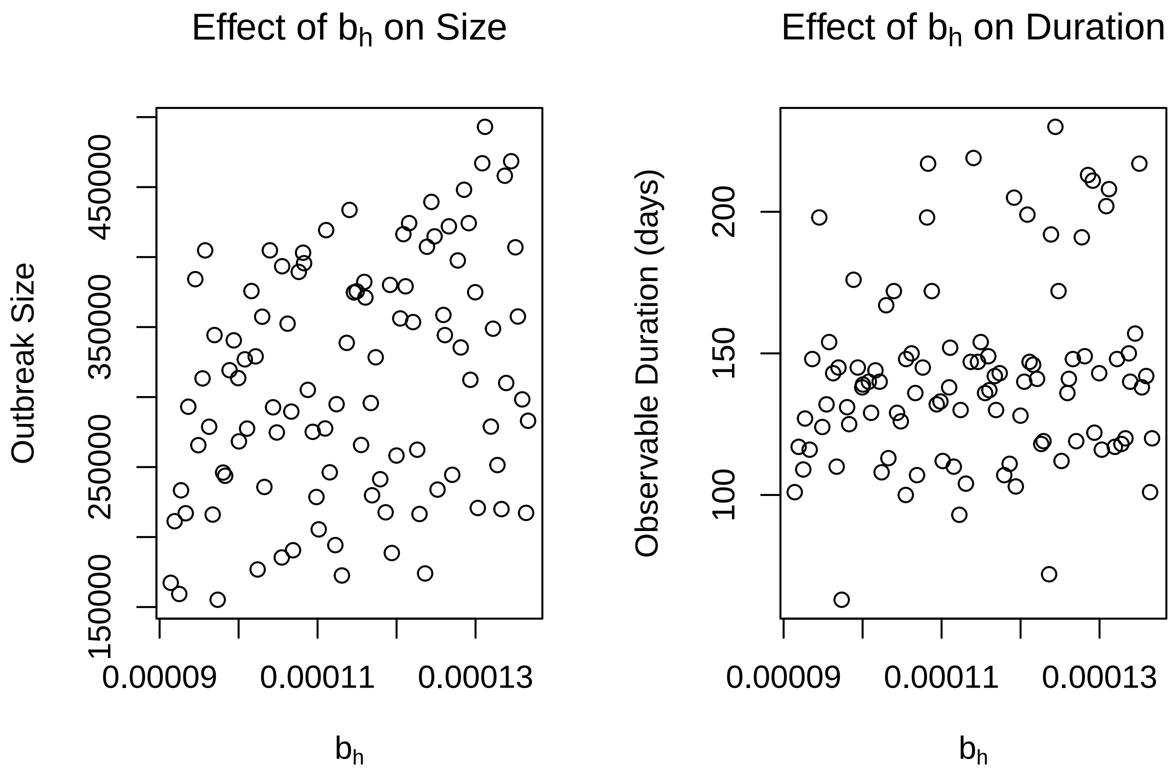


```
plot(mSIR$MaxInf ~ mSIR$d_h, main = expression(paste("Effect of ", d[h], " on Size")),
    xlab = expression(d[h]), ylab = "Outbreak Size")
plot(mSIR$Thresh100 ~ mSIR$d_h, main = expression(paste("Effect of ", d[h], " on Duration")),
    xlab = expression(d[h]), ylab = "Observable Duration (days)")
```

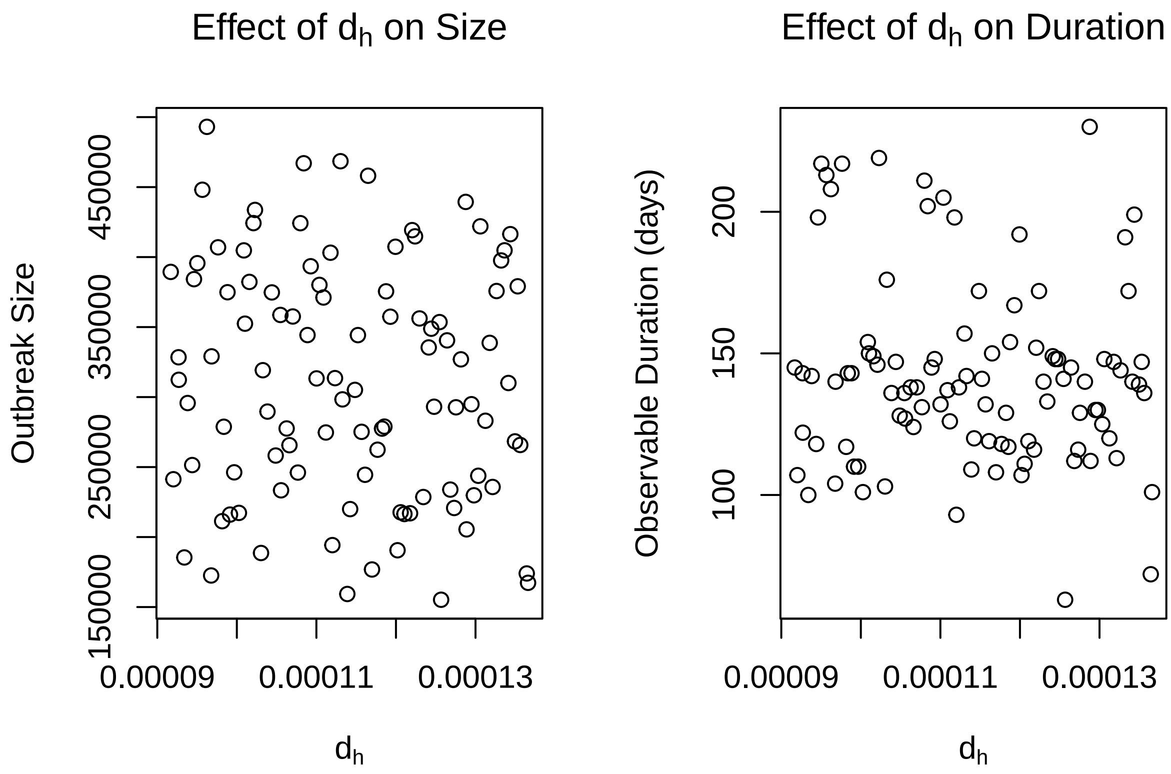


```r
par(mfrow = c(1, 2))
boxplot(mSIR$MaxInf, main = "Outbreak Size", ylab = "Number of Dead Humans", ylim = c(0,
    923406))
boxplot(mSIR$Thresh100, main = "Observable Outbreak Duration", ylab = "Time (Days)")
```

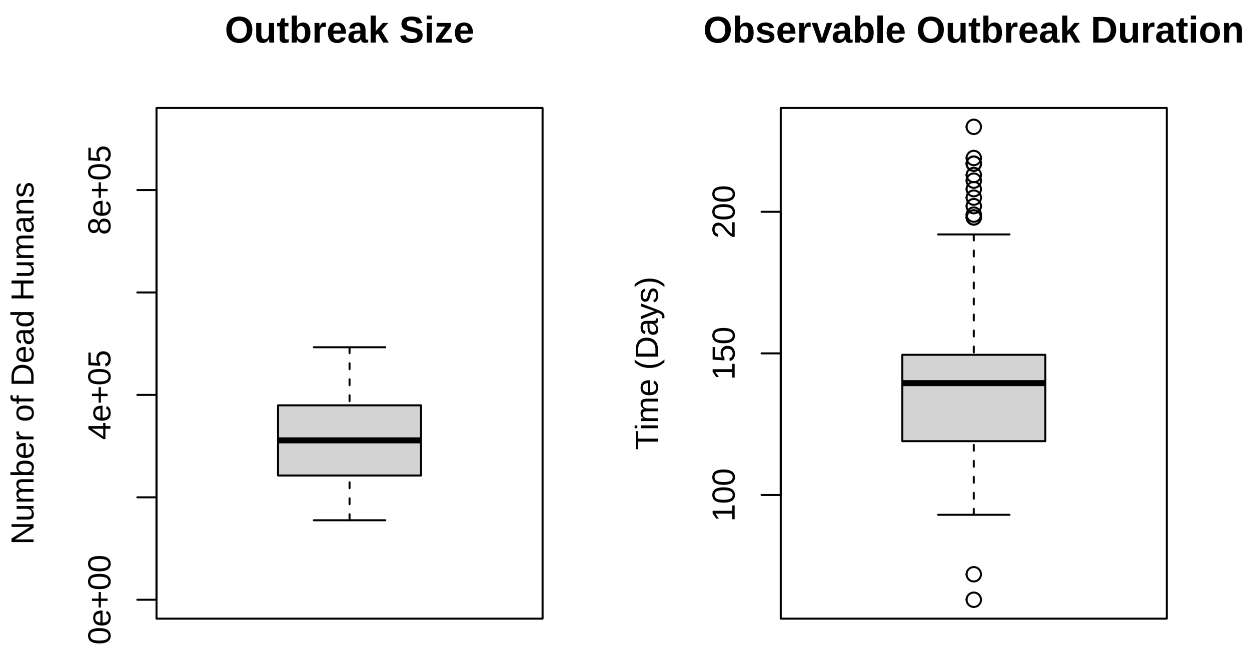


```
bonferroni.alpha <- 0.05/length(parameters)
prcc_size <- pcc(mSIR[, 1:length(parameters)], mSIR$MaxInf, nboot = niter, rank = TRUE,
    conf = 1 - bonferroni.alpha)
prcc_duration <- pcc(mSIR[, 1:length(parameters)], mSIR$Thresh100, nboot = niter,
    rank = TRUE, conf = 1 - bonferroni.alpha)

# plot correlation coefficients and confidence intervals for epidemic size and
# duration
size <- prcc_size$PRCC
size$param <- rownames(size)
colnames(size)[4:5] <- c("maxCI", "minCI")
size$maxCI[which(size$maxCI > 1)] <- 1
size$maxCI[which(size$maxCI < -1)] <- -1
size$minCI[which(size$minCI > 1)] <- 1
size$minCI[which(size$minCI < -1)] <- -1

duration <- prcc_duration$PRCC
duration$param <- rownames(duration)
colnames(duration)[4:5] <- c("maxCI", "minCI")
duration$maxCI[which(duration$maxCI > 1)] <- 1
duration$maxCI[which(duration$maxCI < -1)] <- -1
duration$minCI[which(duration$minCI > 1)] <- 1
duration$minCI[which(duration$minCI < -1)] <- -1


A <- ggplot(size, aes(x = param, y = original)) + geom_point(size = 4) + geom_errorbar(aes(ymax = maxCI
```

```
    ymin = minCI)) + ggtitle("A") + xlab("Parameters") + ylab("Partial Rank Correlation Coefficients") +
    scale_x_discrete(labels = c(beta_m = expression(beta[m]), b_h = expression(b[h]),
        d_h = expression(d[h]), gamma_m = expression(gamma[m]), g_m = expression(g[m]))) +
    ylim(-1, 1)

B <- ggplot(duration, aes(x = param, y = original)) + geom_point(size = 4) + geom_errorbar(aes(ymax = ma
    ymin = minCI)) + ggtitle("B") + xlab("Parameters") + ylab(" ") + scale_x_discrete(labels = c(beta_m
    b_h = expression(b[h]), d_h = expression(d[h]), gamma_m = expression(gamma[m]),
    g_m = expression(g[m]))) + ylim(-1, 1)

multiplot(A, B, cols = 2)
```

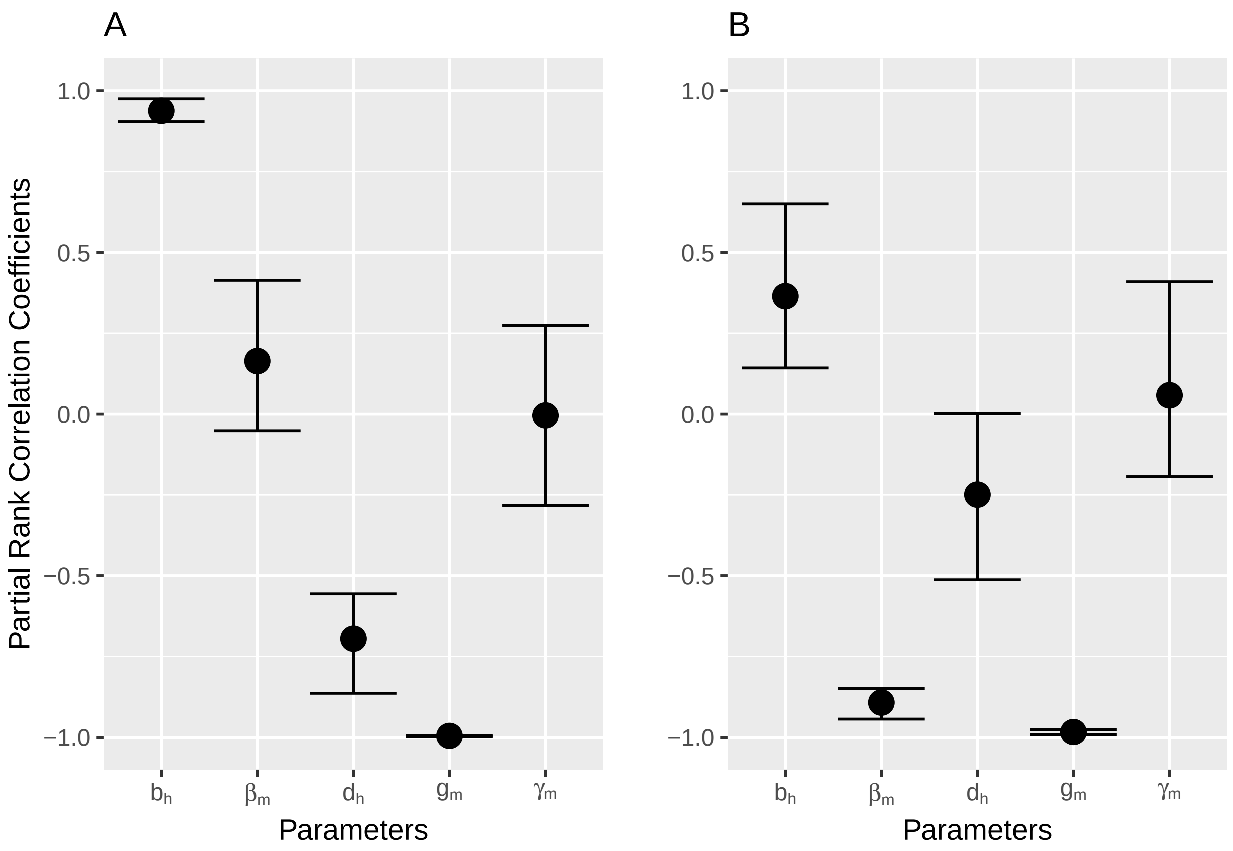


```
# tiff('FigureS20MeaslesSIR_PRCCuniform.tiff', height =8.7 , width =10, units =
# 'cm', compression = 'lzw', res = 1200) multiplot(A, B, cols=2) dev.off()
```

Measles SEIR

```
parameters <- c(beta_m = 1.175, sigma_m = 1/10, gamma_m = 1/13, g_m = 0.7, b_h = 1/(25 *
    365), d_h = 1/(25 * 365))  #you can play with transmission and recovery rates here

# plot scatterplots
par(mfrow = c(1, 2))
plot(mSEIR$MaxInf ~ mSEIR$beta_m, main = expression(paste("Effect of ", beta[m],
```

```
    " on Size")), xlab = expression(beta[m]), ylab = "Outbreak Size")
plot(mSEIR$Thresh100 ~ mSEIR$beta_m, main = expression(paste("Effect of ", beta[m],
    " on Duration")), xlab = expression(beta[m]), ylab = "Observable Duration (days)")
```

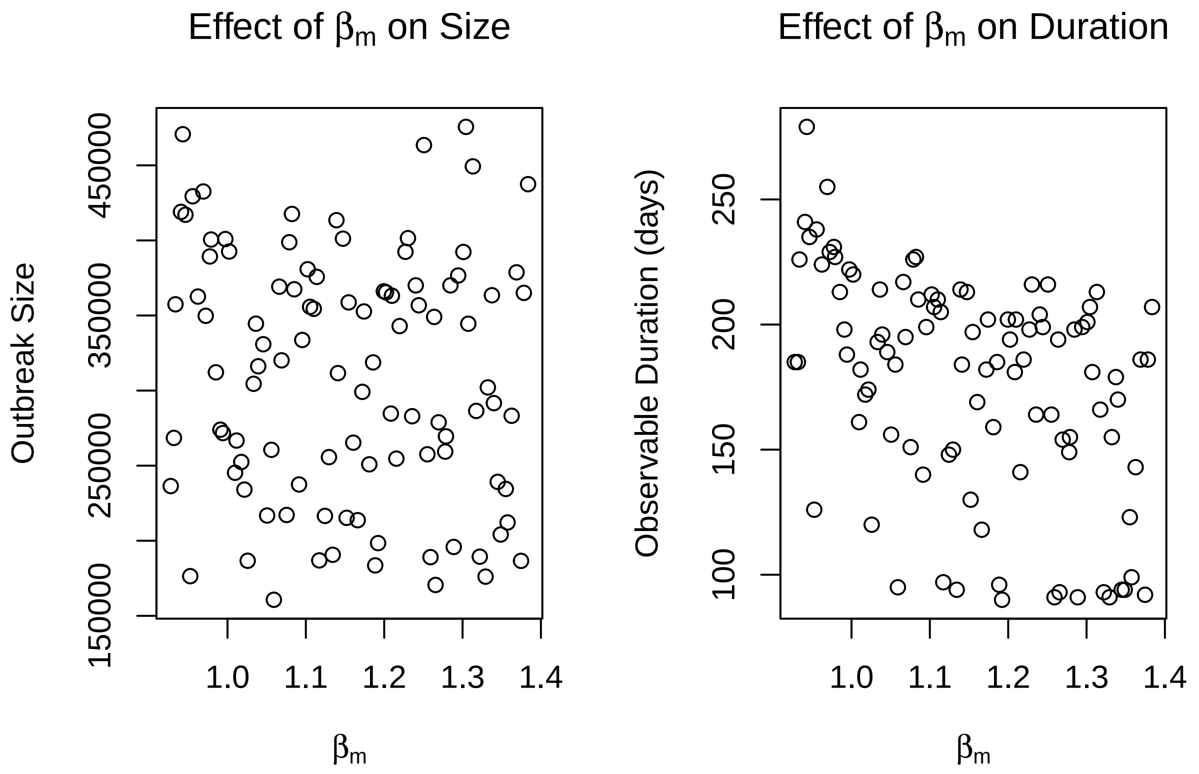


```
plot(mSEIR$MaxInf ~ mSEIR$sigma_m, main = expression(paste("Effect of ", sigma[m],
    " on Size")), xlab = expression(sigma[m]), ylab = "Outbreak Size")
plot(mSEIR$Thresh100 ~ mSEIR$sigma_m, main = expression(paste("Effect of ", sigma[m],
    " on Duration")), xlab = expression(sigma[m]), ylab = "Detectable Outbreak Duration (days)")
```

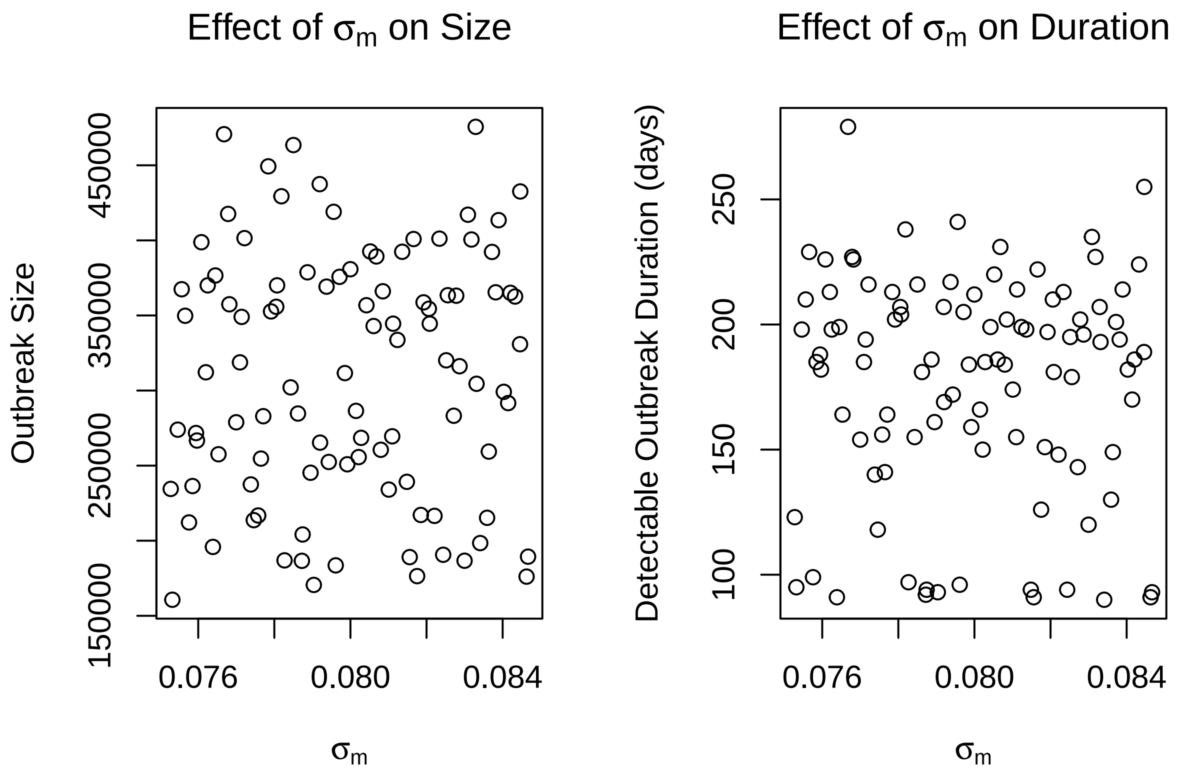


```
plot(mSEIR$MaxInf ~ mSEIR$gamma_m, main = expression(paste("Effect of ", gamma[m],
    " on Size")), xlab = expression(gamma[m]), ylab = "Outbreak Size")
plot(mSEIR$Thresh100 ~ mSEIR$gamma_m, main = expression(paste("Effect of ", gamma[m],
    " on Duration")), xlab = expression(gamma[m]), ylab = "Observable Duration (days)")
```

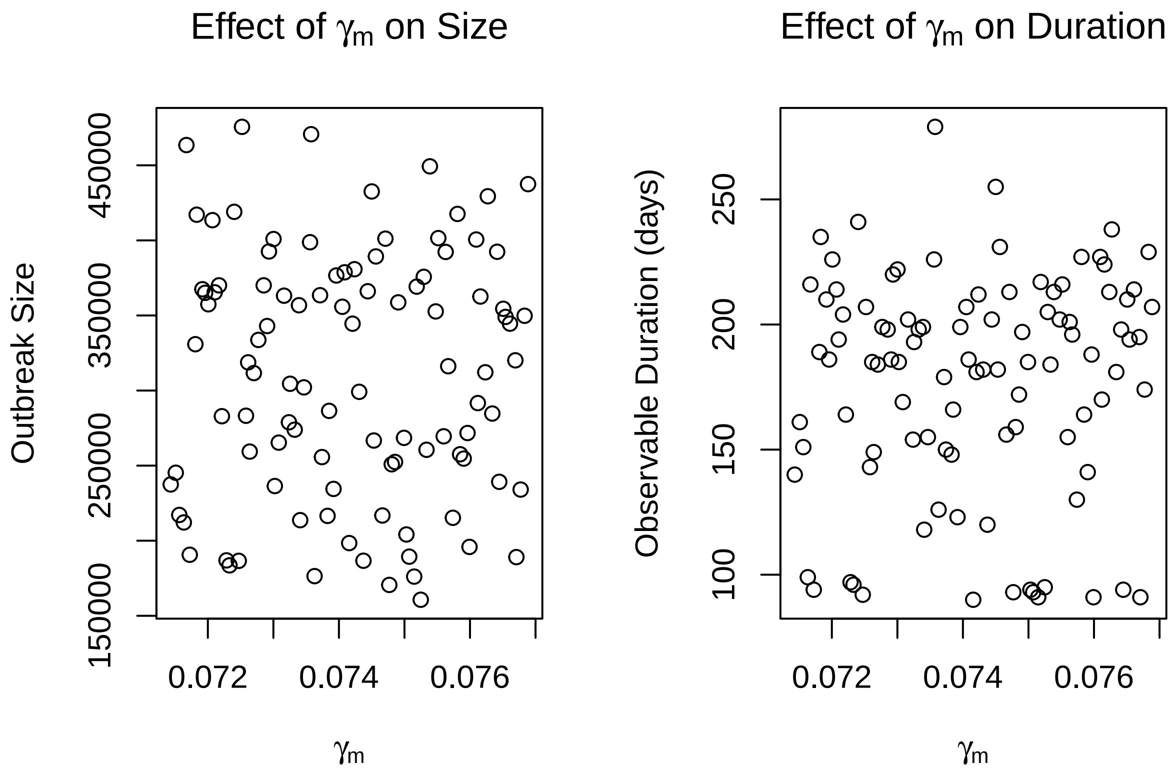


```
plot(mSEIR$MaxInf ~ mSEIR$g_m, main = expression(paste("Effect of ", g[m], " on Size")),
    xlab = expression(g[m]), ylab = "Outbreak Size")
plot(mSEIR$Thresh100 ~ mSEIR$g_m, main = expression(paste("Effect of ", g[m], " on Duration")),
    xlab = expression(g[m]), ylab = "Detectable Duration (days)")
```

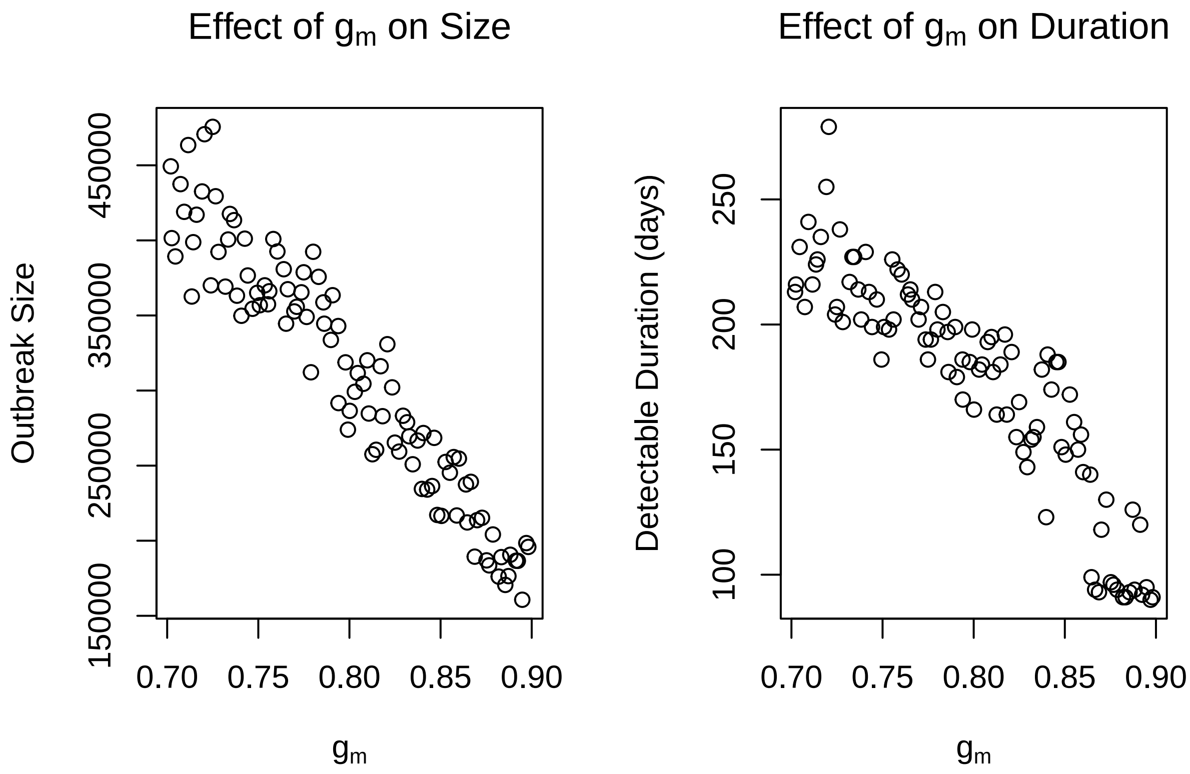


```
plot(mSEIR$MaxInf ~ mSEIR$b_h, main = expression(paste("Effect of ", b[h], " on Size")),
    xlab = expression(b[h]), ylab = "Outbreak Size")
plot(mSEIR$Thresh100 ~ mSEIR$b_h, main = expression(paste("Effect of ", b[h], " on Duration")),
    xlab = expression(b[h]), ylab = "Observable Duration (days)")
```

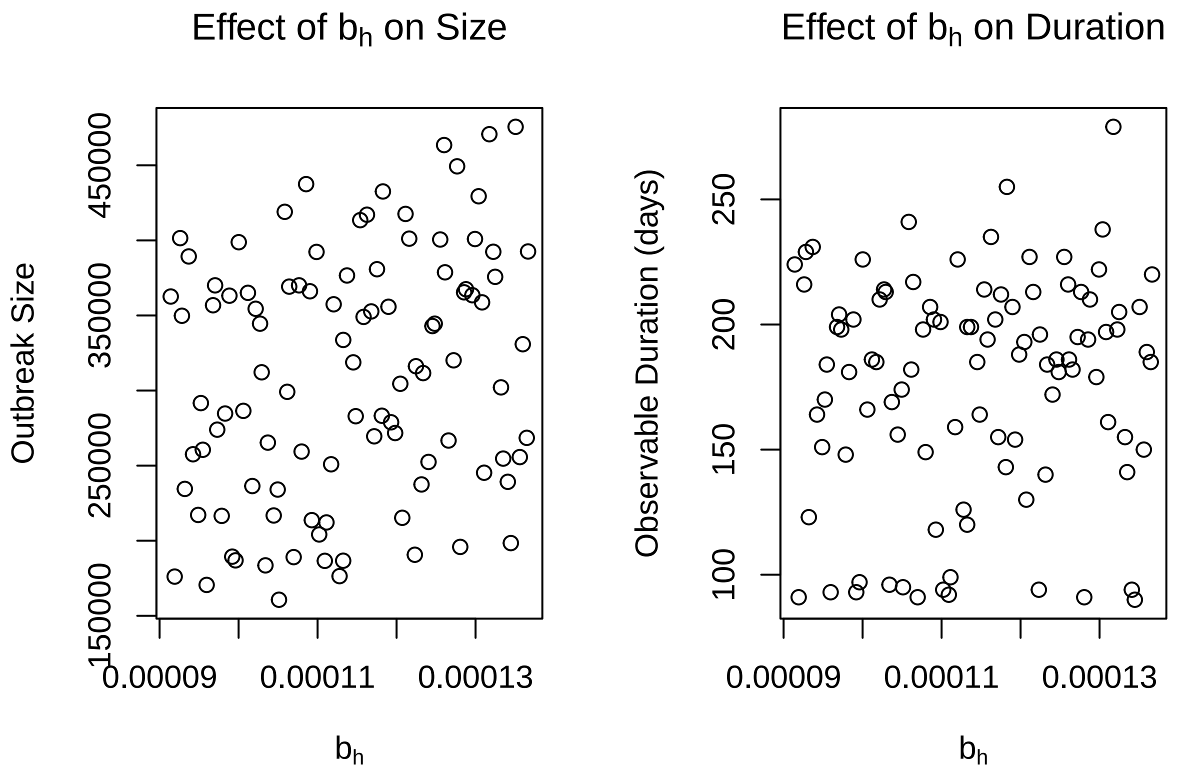


```
plot(mSEIR$MaxInf ~ mSEIR$d_h, main = expression(paste("Effect of ", d[h], " on Size")),
    xlab = expression(d[h]), ylab = "Outbreak Size")
plot(mSEIR$Thresh100 ~ mSEIR$d_h, main = expression(paste("Effect of ", d[h], " on Duration")),
    xlab = expression(d[h]), ylab = "Observable Duration (days)")
```

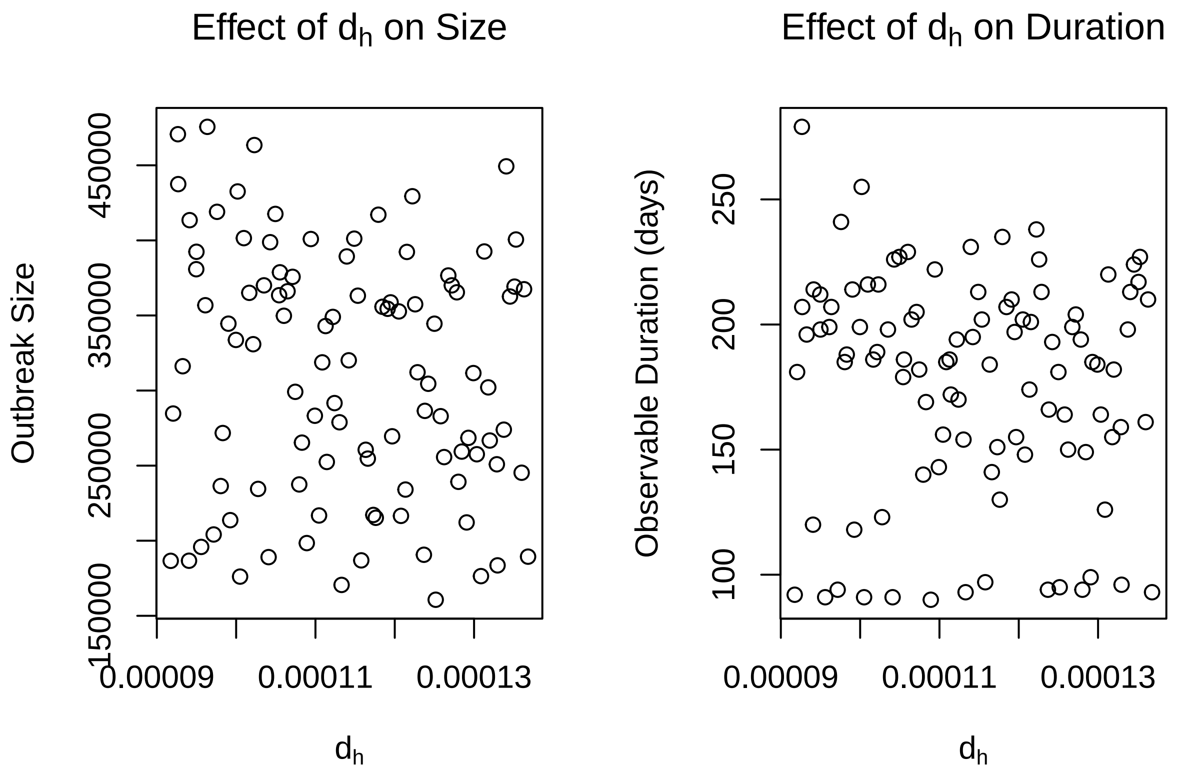


```
par(mfrow = c(1, 2))
boxplot(mSEIR$MaxInf, main = "Outbreak Size", ylab = "Number of Dead Humans", ylim = c(0,
    923406))
boxplot(mSEIR$Thresh100, main = "Outbreak Duration", ylab = "Time (Days)")
```

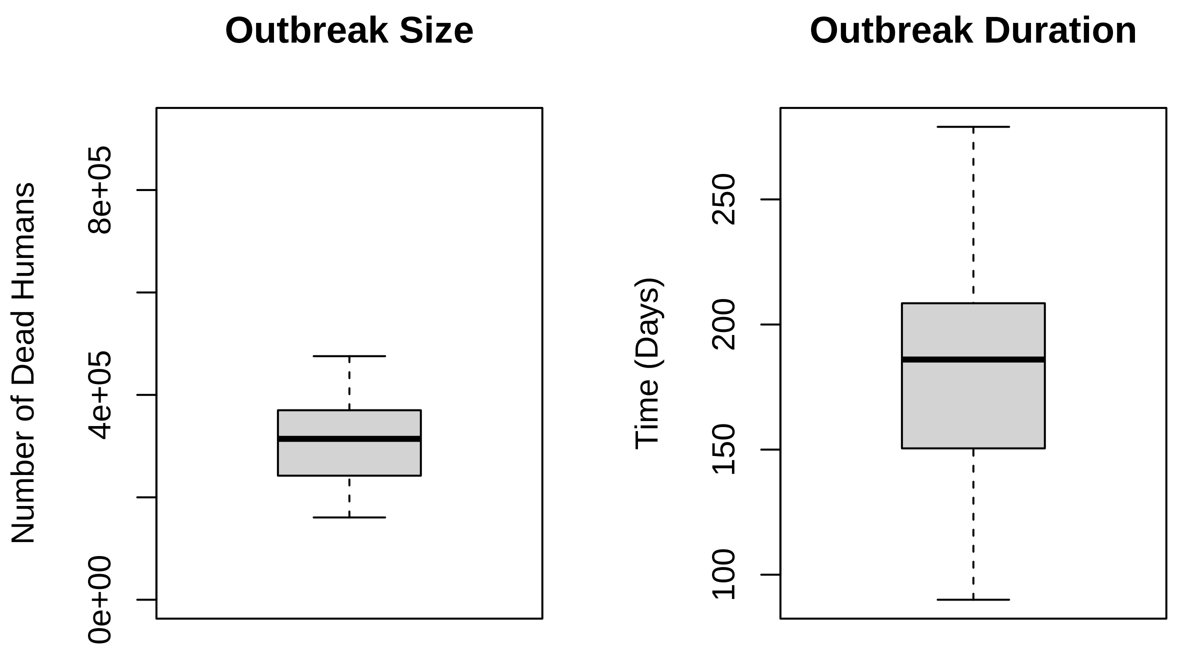


```
bonferroni.alpha <- 0.05/length(parameters)
prcc_size <- pcc(mSEIR[, 1:length(parameters)], mSEIR$MaxInf, nboot = niter, rank = TRUE,
    conf = 1 - bonferroni.alpha)
prcc_duration <- pcc(mSEIR[, 1:length(parameters)], mSEIR$Thresh100, nboot = niter,
    rank = TRUE, conf = 1 - bonferroni.alpha)

# plot correlation coefficients and confidence intervals for epidemic size and
# duration
size <- prcc_size$PRCC
size$param <- rownames(size)
colnames(size)[4:5] <- c("maxCI", "minCI")
size$maxCI[which(size$maxCI > 1)] <- 1
size$maxCI[which(size$maxCI < -1)] <- -1
size$minCI[which(size$minCI > 1)] <- 1
size$minCI[which(size$minCI < -1)] <- -1

duration <- prcc_duration$PRCC
duration$param <- rownames(duration)
colnames(duration)[4:5] <- c("maxCI", "minCI")
duration$maxCI[which(duration$maxCI > 1)] <- 1
duration$maxCI[which(duration$maxCI < -1)] <- -1
duration$minCI[which(duration$minCI > 1)] <- 1
duration$minCI[which(duration$minCI < -1)] <- -1

A <- ggplot(size, aes(x = param, y = original)) + geom_point(size = 4) + geom_errorbar(aes(ymax = maxCI
    ymin = minCI)) + ggtitle("A") + xlab("Parameters") + ylab("Partial Rank Correlation Coefficients")
```

```
    scale_x_discrete(labels = c(beta_m = expression(beta[m]), b_h = expression(b[h]),
        d_h = expression(d[h]), gamma_m = expression(gamma[m]), g_m = expression(g[m]),
        sigma_m = expression(sigma[m]))) + ylim(-1, 1)

B <- ggplot(duration, aes(x = param, y = original)) + geom_point(size = 4) + geom_errorbar(aes(ymax = ma
    ymin = minCI)) + ggtitle("B") + xlab("Parameters") + ylab(" ") + scale_x_discrete(labels = c(beta_m
    b_h = expression(b[h]), d_h = expression(d[h]), gamma_m = expression(gamma[m]),
    g_m = expression(g[m]), sigma_m = expression(sigma[m]))) + ylim(-1, 1)

multiplot(A, B, cols = 2)
```

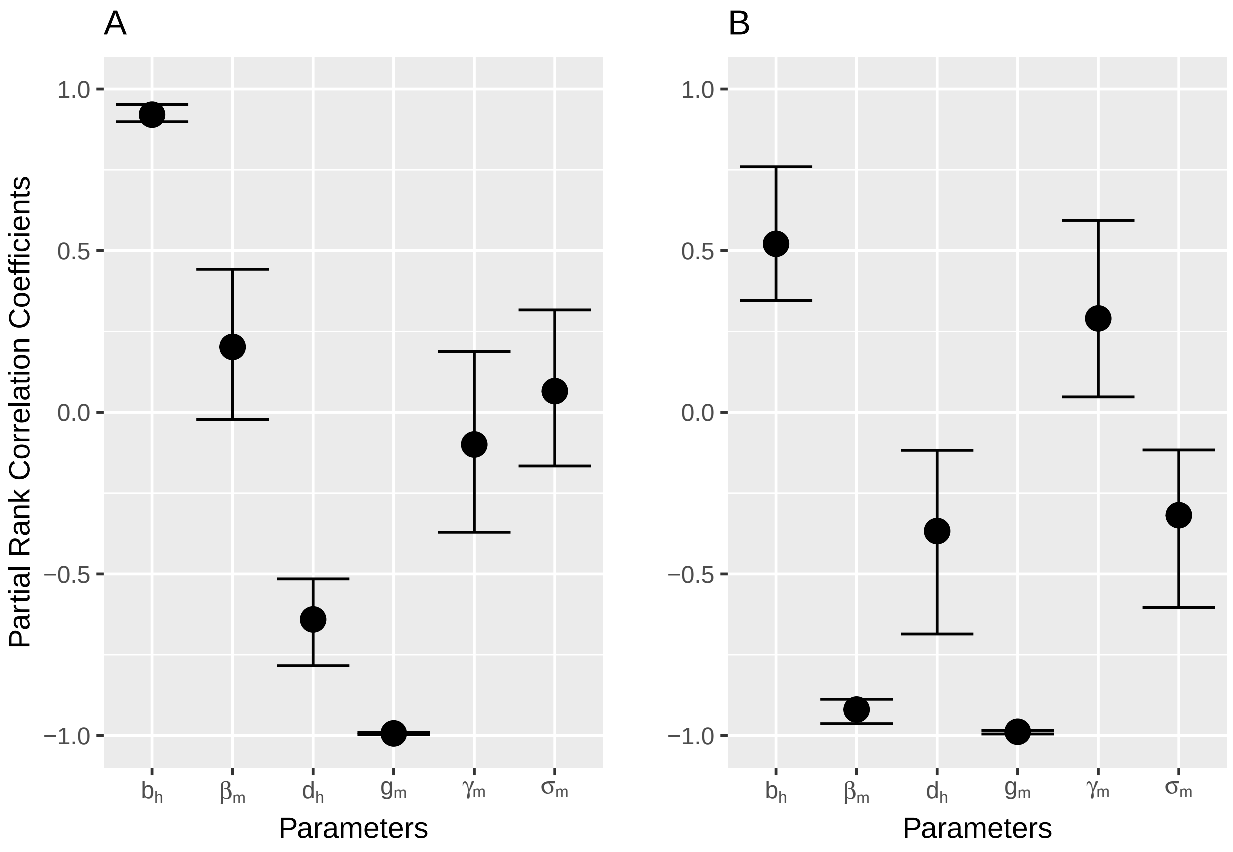


```
# tiff('FigureS21MeaslesSEIR_PRCCuniform.tiff', height =8.7 , width =10, units
# = 'cm', compression = 'lzw', res = 1200) multiplot(A, B, cols=2) dev.off()
```
