# Supplementary material for: Comparative SIR/SEIR modeling of the Antonine Plague in Rome
Source: PLoS One. 2025 Feb 13;20(2):e0313684. doi: 10.1371/journal.pone.0313684 (PMC11824979; doi:10.1371/journal.pone.0313684)
Supplement: S2 Appendix — Contains scatter plots of parameters vs. model outcomes and PRCC plots. (PDF) [file pone.0313684.s010.pdf]

S2. File: Non Uniform Sensitivity Analysis

Anestis Karasaridis (file adapted from an original by Lauren White)

January 16th, 2022

Sensitivity Analysis with Non-Uniform Parameter Distributions

- Begin by creating LHS sampling space with `LHSnonuniform.R`

```
#' Adapted from: https://daphnia.ecology.uga.edu/drakelab/wp-content/uploads/2015/07/sensitivity-ebola.[illegible]
#' load ode functions for each of the models
source("./Plague_model_functions.R")

require(lhs)  #add the lhs library
library(sensitivity)
require(ggplot2)
library(tidyverse)
library(ggforce)
require(deSolve)

set.seed(2718)  #set random seed
times <- seq(0, 9500, by = 1)
h <- 100  #choose number of parameter sets/subdivisions to sample to sample
N_r0 <- 923406  #initial conditions for ODE model- number of rats
niter <- 500  #number of times to bootstrap CI for PRCC

#' load uniform and non uniform LHS distributions
source("./LHSnonuniform.R")
uniform <- FALSE  #choose uniform (TRUE) or non-uniform (FALSE) distributions
```

Latin Hypercube Sampling and Partial Ranked Correlation Coefficients (LHS-PRCC)

- Use `GlobalSensitivityAnalysis.R` to run ODE models on LHS parameter sets produced in `LHSnonuniform.R`
- Plot scatter plots of each parameter vs. outbreak size and dectable outbreak duration (days)
- Calculate and plot PRCC values for each parameter for outbreak size and detectable outbreak duration (days)

```
source("./GlobalSensitivityAnalysis.R")
```

Define multiplot function

Use to produce multipaneled ggplot2 figures

```
#' Multiplot function
# ggplot objects can be passed in ..., or to plotlist (as a list of ggplot
# objects) - cols: Number of columns in layout - layout: A matrix specifying
# the layout. If present, 'cols' is ignored.  If the layout is something like
# matrix(c(1,2,3,3), nrow=2, byrow=TRUE), then plot 1 will go in the upper
# left, 2 will go in the upper right, and 3 will go all the way across the
# bottom.
multiplot <- function(..., plotlist = NULL, file, cols = 1, layout = NULL) {
    library(grid)

    # Make a list from the ... arguments and plotlist
    plots <- c(list(...), plotlist)

    numPlots = length(plots)

    # If layout is NULL, then use 'cols' to determine layout
    if (is.null(layout)) {
        # Make the panel ncol: Number of columns of plots nrow: Number of rows
        # needed, calculated from # of cols
        layout <- matrix(seq(1, cols * ceiling(numPlots/cols)), ncol = cols, nrow = ceiling(numPlots/col
            byrow = TRUE)
    }

    if (numPlots == 1) {
        print(plots[[1]])

    } else {
        # Set up the page
        grid.newpage()
        pushViewport(viewport(layout = grid.layout(nrow(layout), ncol(layout))))

        # Make each plot, in the correct location
        for (i in 1:numPlots) {
            # Get the i,j matrix positions of the regions that contain this
            # subplot
            matchidx <- as.data.frame(which(layout == i, arr.ind = TRUE))

            print(plots[[i]], vp = viewport(layout.pos.row = matchidx$row, layout.pos.col = matchidx$col
        }
    }
}
```

Comparative Figure Across Models

```
# Comparative Figure for All Models ---------------------------------------
comp_size <- data.frame(bSIR = bSIR$MaxInf, bSEIR = bSEIR$MaxInf, bSIRrK = bSIRrK$MaxInf,
    bSEIRrK = bSEIRrK$MaxInf, bpSEIR = bpSEIR$MaxInf, sSIR = sSIR$MaxInf, sSEIR = sSEIR$MaxInf,
    mSIR = mSIR$MaxInf, mSEIR = mSEIR$MaxInf)
```

```
comp_dur <- data.frame(bSIR = bSIR$Thresh100, bSEIR = bSEIR$Thresh100, bSIRrK = bSIRrK$Thresh100,
    bSEIRrK = bSEIRrK$Thresh100, bpSEIR = bpSEIR$Thresh100, sSIR = sSIR$Thresh100,
    sSEIR = sSEIR$Thresh100, mSIR = mSIR$Thresh100, mSEIR = mSEIR$Thresh100)
comp_dur250 <- data.frame(bSIR = bSIR$Thresh250, bSEIR = bSEIR$Thresh250, bSIRrK = bSIRrK$Thresh250,
    bSEIRrK = bSEIRrK$Thresh250, bpSEIR = bpSEIR$Thresh250, sSIR = sSIR$Thresh250,
    sSEIR = sSEIR$Thresh250, mSIR = mSIR$Thresh250, mSEIR = mSEIR$Thresh250)
comp_dur2000 <- data.frame(bSIR = bSIR$Thresh2000, bSEIR = bSEIR$Thresh2000, bSIRrK = bSIRrK$Thresh2000
    bSEIRrK = bSEIRrK$Thresh2000, bpSEIR = bpSEIR$Thresh2000, sSIR = sSIR$Thresh2000,
    sSEIR = sSEIR$Thresh2000, mSIR = mSIR$Thresh2000, mSEIR = mSEIR$Thresh2000)

long_DFsize <- comp_size %>%
    gather(Model, NumberDead, c(bSIR, bSEIR, bSIRrK, bSEIRrK, bpSEIR, sSIR, sSEIR,
        mSIR, mSEIR))
long_DFdur <- comp_size %>%
    gather(Model, Duration, c(bSIR, bSEIR, bSIRrK, bSEIRrK, bpSEIR, sSIR, sSEIR,
        mSIR, mSEIR))
long_DFdur250 <- comp_dur250 %>%
    gather(Model, Duration, c(bSIR, bSEIR, bSIRrK, bSEIRrK, bpSEIR, sSIR, sSEIR,
        mSIR, mSEIR))
long_DFdur2000 <- comp_dur2000 %>%
    gather(Model, Duration, c(bSIR, bSEIR, bSIRrK, bSEIRrK, bpSEIR, sSIR, sSEIR,
        mSIR, mSEIR))

long_DFsize$Model <- as.character(long_DFsize$Model)
long_DFsize$Model <- factor(long_DFsize$Model, levels = c("bSIR", "bSEIR", "bSIRrK",
    "bSEIRrK", "bpSEIR", "sSIR", "sSEIR", "mSIR", "mSEIR"))

long_DFdur$Model <- as.character(long_DFdur$Model)
long_DFdur$Model <- factor(long_DFdur$Model, levels = c("bSIR", "bSEIR", "bSIRrK",
    "bSEIRrK", "bpSEIR", "sSIR", "sSEIR", "mSIR", "mSEIR"))

long_DFdur250$Model <- as.character(long_DFdur250$Model)
long_DFdur250$Model <- factor(long_DFdur250$Model, levels = c("bSIR", "bSEIR", "bSIRrK",
    "bSEIRrK", "bpSEIR", "sSIR", "sSEIR", "mSIR", "mSEIR"))

long_DFdur2000$Model <- as.character(long_DFdur2000$Model)
long_DFdur2000$Model <- factor(long_DFdur2000$Model, levels = c("bSIR", "bSEIR",
    "bSIRrK", "bSEIRrK", "bpSEIR", "sSIR", "sSEIR", "mSIR", "mSEIR"))

A <- ggplot(long_DFsize, aes(Model, NumberDead)) + geom_boxplot() + geom_jitter(alpha = 0.5) +
    ylab("Number of Human\nMortalities") + xlab("") + scale_x_discrete(labels = c(bSIR = "Bubonic\nSIR"
    bSEIR = "Bubonic\nSEIR", bSIRrK = "Bubonic SIR\n(Rat Dyn.)", bpSEIR = "Bubonic &\nPneumonic\nSEIR",
    bSEIRrK = "Bubonic SEIR\n(Rat Dyn.)", sSIR = "Smallpox\nSIR", sSEIR = "Smallpox\nSEIR",
    mSIR = "Measles\nSIR", mSEIR = "Measles\nSEIR")) + geom_hline(yintercept = 923406/2,
    color = "red") + ggtitle("A") + theme_bw() + theme(panel.border = element_blank(),
    panel.grid.major = element_blank(), panel.grid.minor = element_blank(), axis.line = element_line(co
    axis.text.x = element_text(angle = 90, hjust = 1, vjust = 0.5))

B <- ggplot(long_DFdur, aes(Model, Duration)) + geom_boxplot() + geom_jitter(alpha = 0.5) +
    ylab("Detectable Duration\n (>100 Deaths/Day) (Days)") + xlab("") + scale_x_discrete(labels = c(bSI
    bSEIR = "Bubonic\nSEIR", bSIRrK = "Bubonic SIR\n(Rat Dyn.)", bpSEIR = "Bubonic &\nPneumonic\nSEIR",
    bSEIRrK = "Bubonic SEIR\n(Rat Dyn.)", sSIR = "Smallpox\nSIR", sSEIR = "Smallpox\nSEIR",
    mSIR = "Measles\nSIR", mSEIR = "Measles\nSEIR")) + theme(axis.text.x = element_text(angle = 90,
```

```
    hjust = 1)) + geom_hline(yintercept = 120, color = "red") + ggtitle("B") + theme_bw() +
    theme(panel.border = element_blank(), panel.grid.major = element_blank(), panel.grid.minor = element
        axis.line = element_line(colour = "black"), axis.text.x = element_text(angle = 90,
            hjust = 1, vjust = 0.5)) + facet_zoom(ylim = c(0, 300))

C <- ggplot(long_DFdur250, aes(Model, Duration)) + geom_boxplot() + geom_jitter(alpha = 0.5) +
    ylab("Detectable Duration\n (>250 Deaths/Day) (Days)") + xlab("") + scale_x_discrete(labels = c(bSI
    bSEIR = "Bubonic\nSEIR", bSIRrK = "Bubonic SIR\n(Rat Dyn.)", bpSEIR = "Bubonic &\nPneumonic\nSEIR",
    bSEIRrK = "Bubonic SEIR\n(Rat Dyn.)", sSIR = "Smallpox\nSIR", sSEIR = "Smallpox\nSEIR",
    mSIR = "Measles\nSIR", mSEIR = "Measles\nSEIR")) + theme(axis.text.x = element_text(angle = 90,
    hjust = 1)) + geom_hline(yintercept = 90, color = "red") + ggtitle("C") + theme_bw() +
    theme(panel.border = element_blank(), panel.grid.major = element_blank(), panel.grid.minor = elemen
        axis.line = element_line(colour = "black"), axis.text.x = element_text(angle = 90,
            hjust = 1, vjust = 0.5)) + facet_zoom(ylim = c(0, 250))

D <- ggplot(long_DFdur2000, aes(Model, Duration)) + geom_boxplot() + geom_jitter(alpha = 0.5) +
    ylab("Detectable Duration\n (>2000 Deaths/Day) (Days)") + xlab("") + scale_x_discrete(labels = c(bS
    bSEIR = "Bubonic\nSEIR", bSIRrK = "Bubonic SIR\n(Rat Dyn.)", bpSEIR = "Bubonic &\nPneumonic\nSEIR",
    bSEIRrK = "Bubonic SEIR\n(Rat Dyn.)", sSIR = "Smallpox\nSIR", sSEIR = "Smallpox\nSEIR",
    mSIR = "Measles\nSIR", mSEIR = "Measles\nSEIR")) + theme(axis.text.x = element_text(angle = 90,
    hjust = 1)) + geom_hline(yintercept = 30, color = "red") + ggtitle("C") + theme_bw() +
    theme(panel.border = element_blank(), panel.grid.major = element_blank(), panel.grid.minor = elemen
        axis.line = element_line(colour = "black"), axis.text.x = element_text(angle = 90,
            hjust = 1, vjust = 0.5)) + facet_zoom(ylim = c(0, 250))

tiff("Fig2.tiff", height = 22.23, width = 19.05, units = "cm", compression = "lzw",
    res = 600)
multiplot(A, B, C, D, cols = 1)
dev.off()
```

```
## pdf
##   2
```

LHS-PRCC Results

In the basic bubonic plague SIR and SEIR models without rat population dynamics, flea searching efficiency (α), flea death rate (d_f), and rat recovery probability (g_r) were negatively correlated with the size of outbreak (number of deaths) (Fig S4 , panels A & C) and its duration (Fig S4, panel B & D). In the bubonic plague SIR and SEIR models with rat population dynamics, flea searching efficiency (α) was negatively correlated with the size of the outbreak, and transmission rate from fleas to humans (β_b) was moderately positively correlated with the size outbreak (Fig S5, panels A & C). In the bubonic-pneumonic plague SEIR model, duration of pneumonic infection period in humans (γ_p^{-1}) was negatively correlated with outbreak size, duration of pneumonic incubation period in humans (σ_p^{-1}) was positively correlated with outbreak size (Fig S6, panel A), flea searching efficiency (α) was moderately positively correlated with outbreak duration, and transmission rate of bubonic plague from fleas to humans (β_b) was negatively correlated with outbreak duration (Fig S6, panel B). The sensitivity analysis of plague models corresponds with the results of White and Mordechai (12). In the smallpox SIR and SEIR models, birth rate (b_h) was positively correlated with outbreak size, natural death rate (d_h) was moderately negatively correlated with outbreak size (the correlation was stronger in the SIR model), and probability of recovering from smallpox (g_s) was strongly negatively correlated with outbreak size (Fig S7, panels A & C). Furthermore, birth rate (b_h) and probability of recovering from smallpox (g_s) were positively correlated with outbreak duration (Fig S7, panels B & D). In addition, duration of smallpox incubation period (σ_s^{-1}) was moderately negatively correlated with outbreak

duration in the SEIR model (Fig S7, panel D). In the measles SIR and SEIR model, birth rate (b_h) was strongly positively correlated with outbreak size, and natural death rate (d_h) and probability of recovering from measles (g_m) were strongly negatively correlated with outbreak size (Fig S8, panels A & C). Moreover, transmission rate of measles (β_m) and probability of recovering from measles (g_m) were strongly negatively correlated with outbreak duration (Fig S8, panels B & D).

Bubonic SIR model - Figure S4 (Panels A & B)

```
parameters <- c(beta_r = 0.09, alpha = 3/923406, gamma_r = 1/5.15, g_r = 0.1, r_f = 0.0084,
    K_f = 6, d_f = 1/5, beta_h = 0.19, gamma_h = 1/10, g_h = 0.34, b_h = 1/(25 *
        365), d_h = 1/(25 * 365))  #you can play with transmission and recovery rates here

par(mfrow = c(1, 2))
plot(bSIR$MaxInf ~ bSIR$beta_r, main = expression(paste("Effect of ", beta[r], " on Size")),
    xlab = expression(beta[r]), ylab = "Outbreak Size")
plot(bSIR$Thresh100 ~ bSIR$beta_r, main = expression(paste("Effect of ", beta[r],
    " on Duration")), xlab = expression(beta[r]), ylab = "Detectable Duration (days)")
```

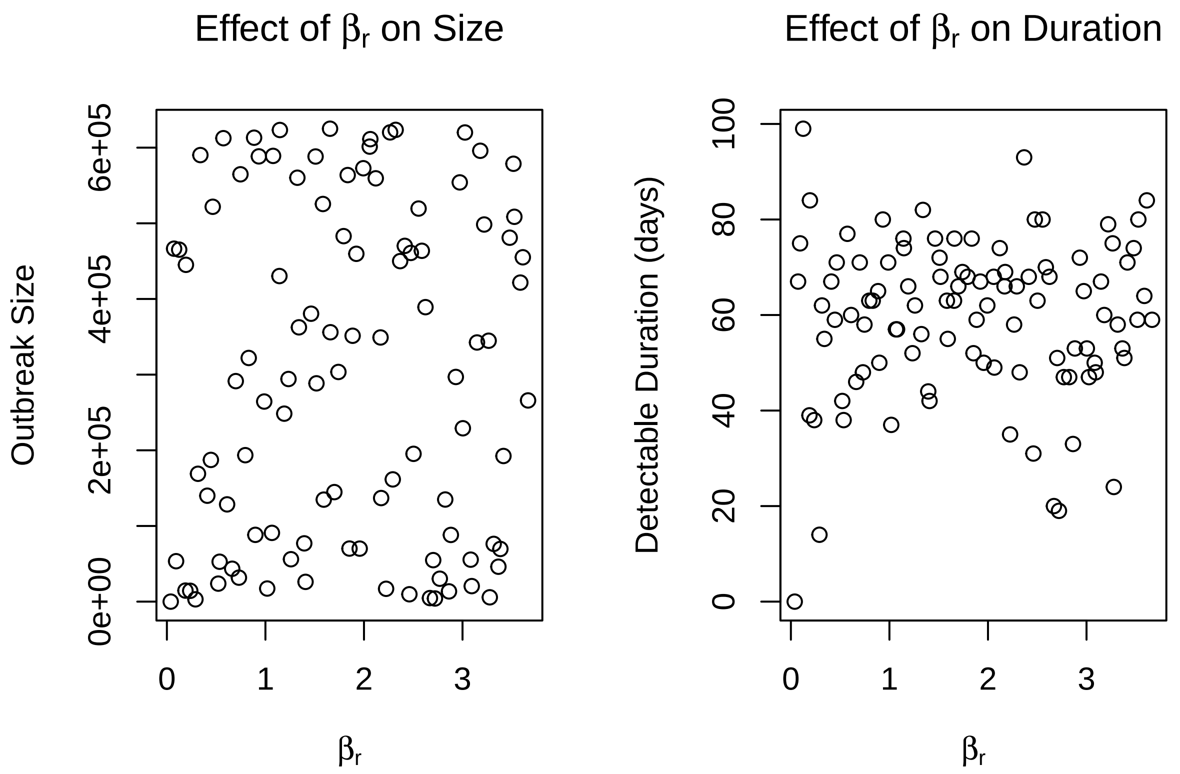


```
plot(bSIR$MaxInf ~ bSIR$alpha, main = expression(paste("Effect of ", alpha, " on Size")),
    xlab = expression(alpha), ylab = "Outbreak Size")
plot(bSIR$Thresh100 ~ bSIR$alpha, main = expression(paste("Effect of ", alpha, " on Duration")),
    xlab = expression(alpha), ylab = "Detectable Duration (days)")
```

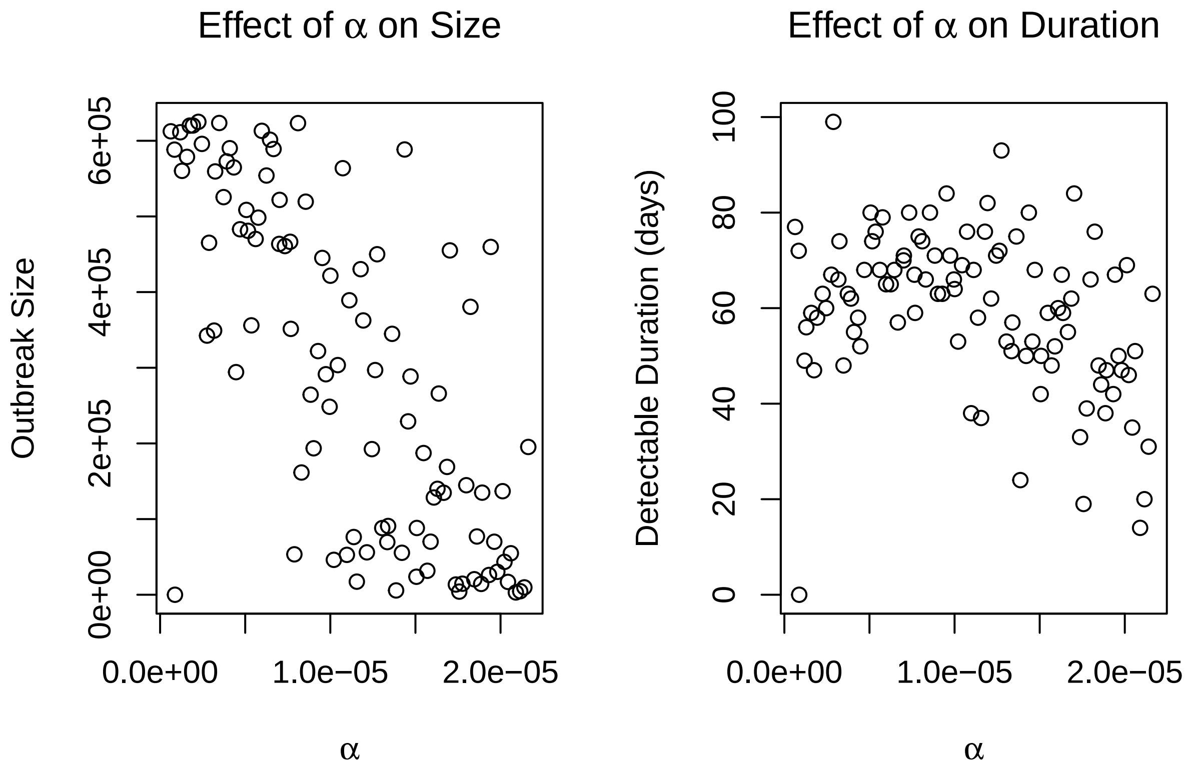


```r
plot(bSIR$MaxInf ~ bSIR$gamma_r, main = expression(paste("Effect of ", gamma[r],
    " on Size")), xlab = expression(gamma[r]), ylab = "Outbreak Size")
plot(bSIR$Thresh100 ~ bSIR$gamma_r, main = expression(paste("Effect of ", gamma[r],
    " on Duration")), xlab = expression(gamma[r]), ylab = "Detectable Duration (days)")
```

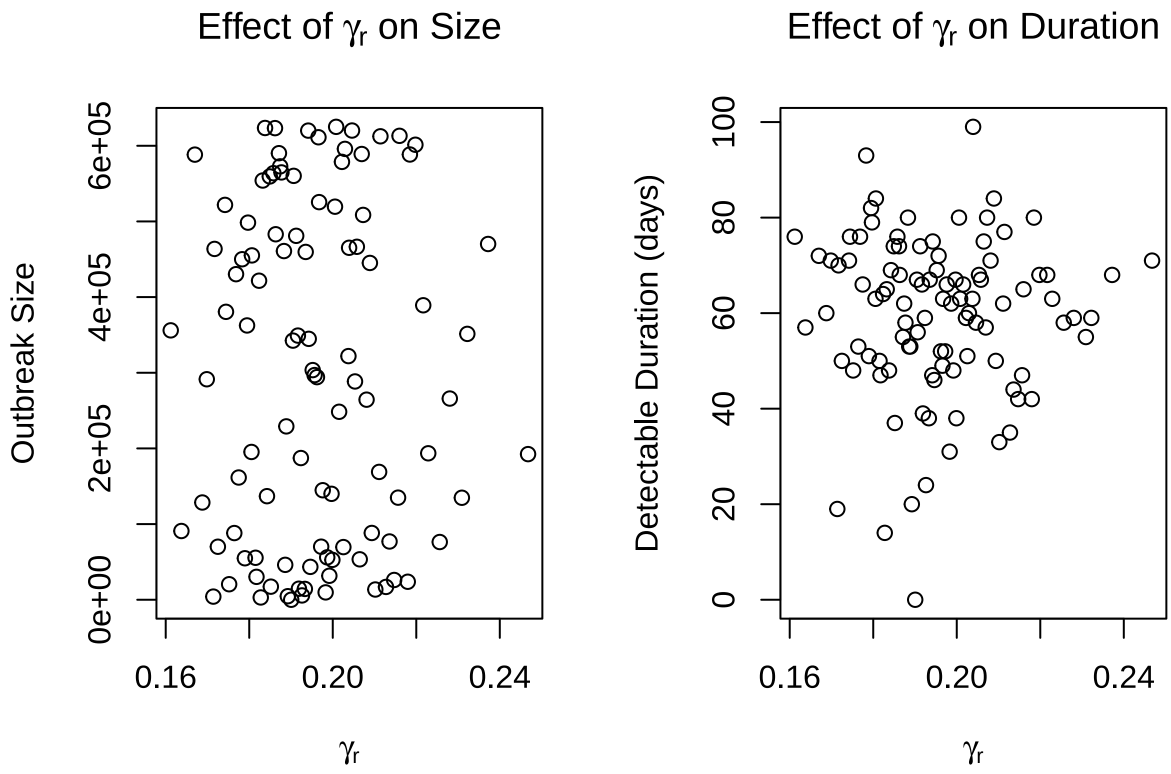


```r
plot(bSIR$MaxInf ~ bSIR$g_r, main = expression(paste("Effect of ", g[r], " on Size")),
    xlab = expression(g[r]), ylab = "Outbreak Size")
plot(bSIR$Thresh100 ~ bSIR$g_r, main = expression(paste("Effect of ", g[r], " on Duration")),
    xlab = expression(g[r]), ylab = "Detectable Duration (days)")
```

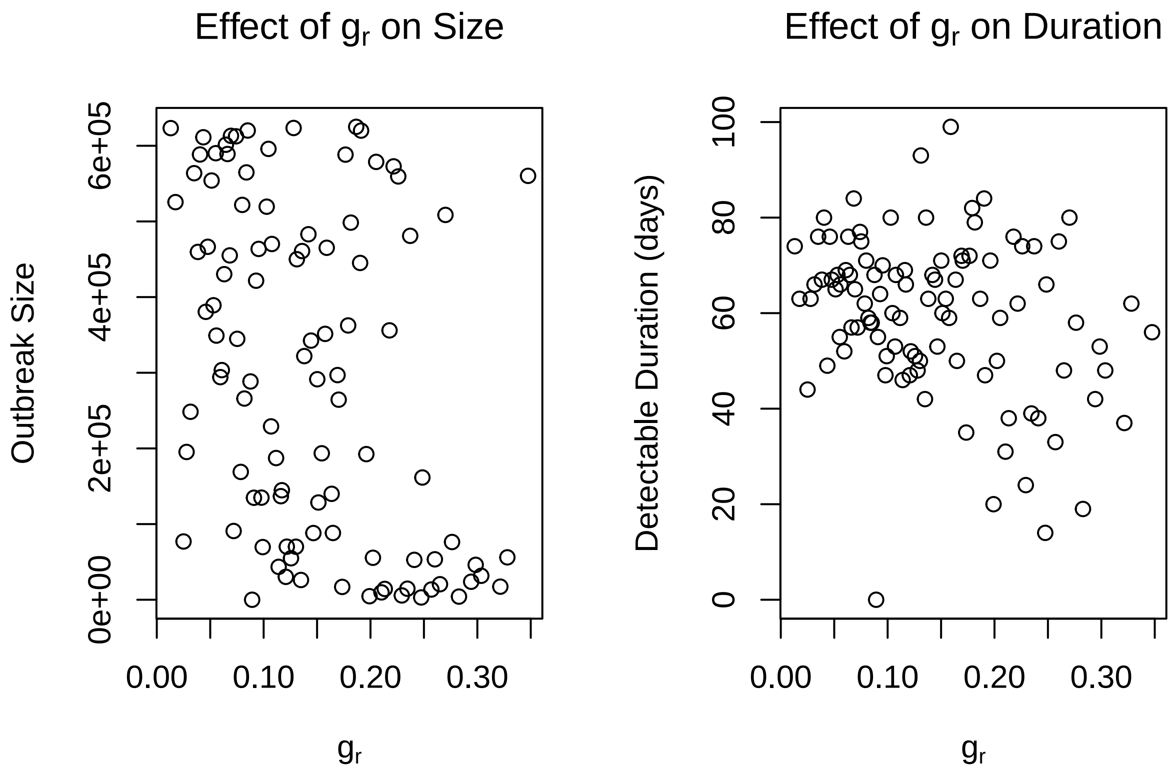


```
plot(bSIR$MaxInf ~ bSIR$r_f, main = expression(paste("Effect of ", r[f], " on Size")),
    xlab = expression(r[f]), ylab = "Outbreak Size")
plot(bSIR$Thresh100 ~ bSIR$r_f, main = expression(paste("Effect of ", r[f], " on Duration")),
    xlab = expression(r[f]), ylab = "Detectable Duration (days)")
```

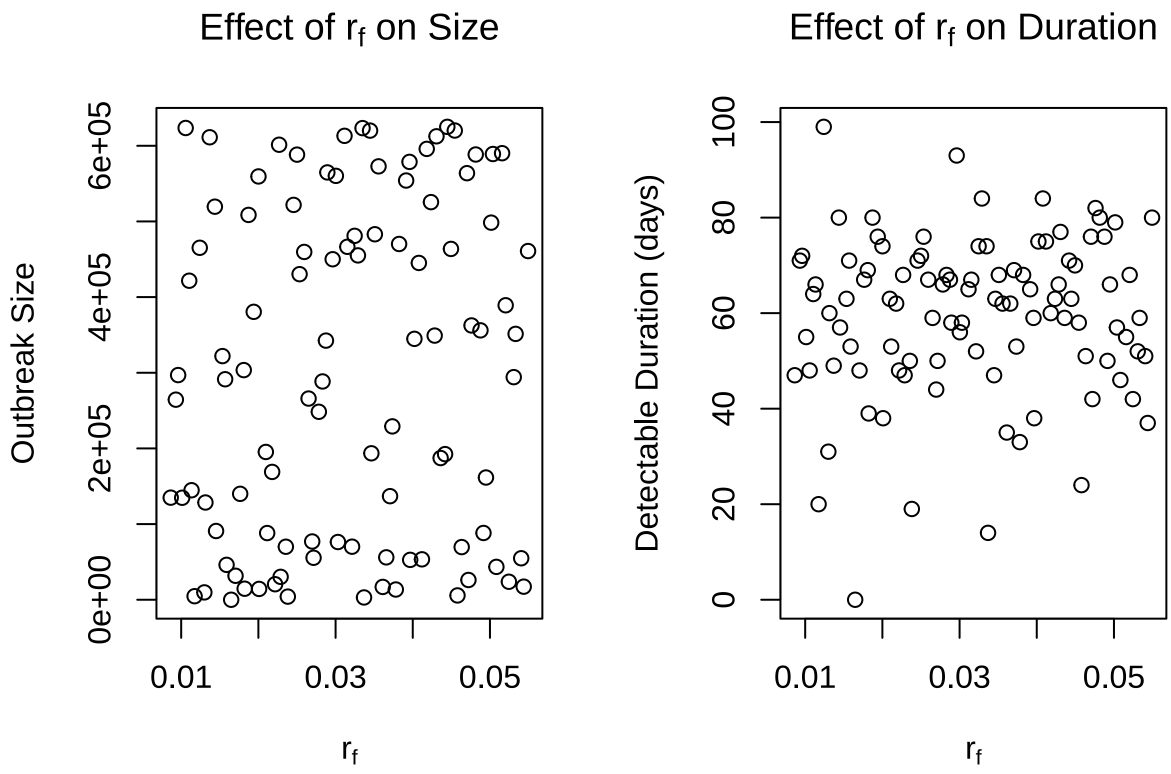


```
plot(bSIR$MaxInf ~ bSIR$K_f, main = expression(paste("Effect of ", K[f], " on Size")),
    xlab = expression(K[f]), ylab = "Outbreak Size")
plot(bSIR$Thresh100 ~ bSIR$K_f, main = expression(paste("Effect of ", K[f], " on Duration")),
    xlab = expression(K[f]), ylab = "Detectable Duration (days)")
```

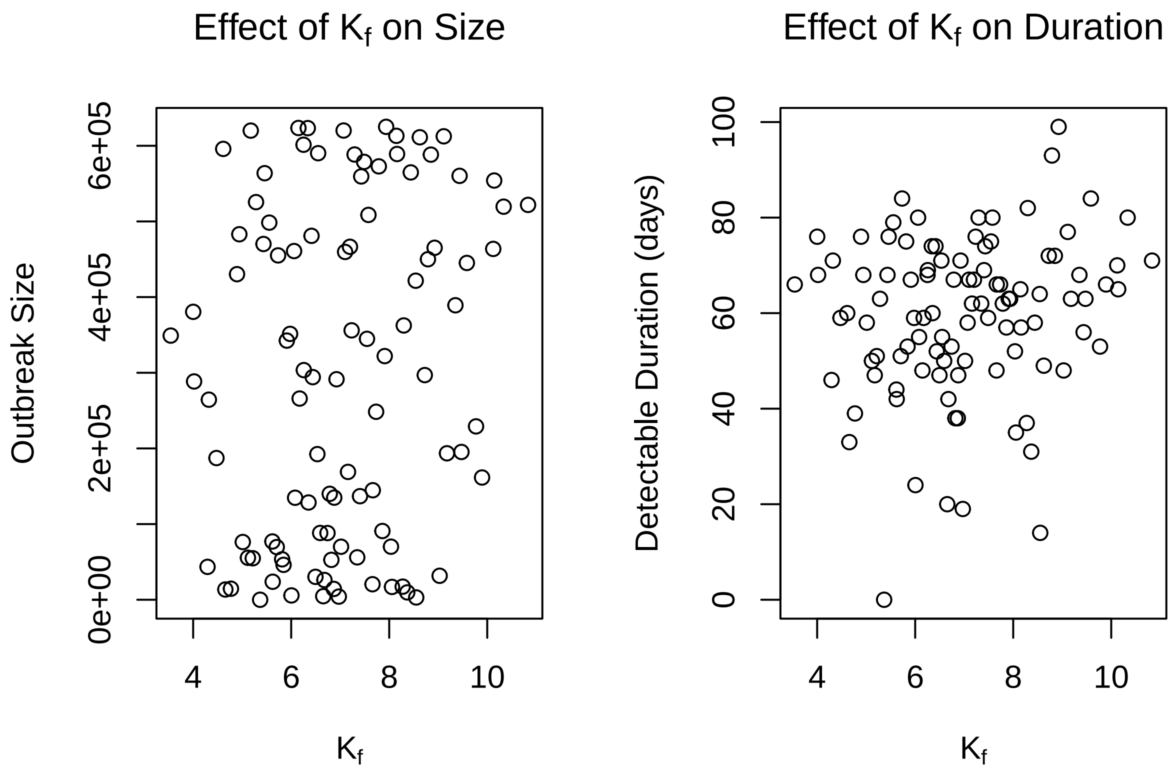


```
plot(bSIR$MaxInf ~ bSIR$d_f, main = expression(paste("Effect of ", d[f], " on Size")),
    xlab = expression(d[f]), ylab = "Outbreak Size")
plot(bSIR$Thresh100 ~ bSIR$d_f, main = expression(paste("Effect of ", d[f], " on Duration")),
    xlab = expression(d[f]), ylab = "Detectable Duration (days)")
```

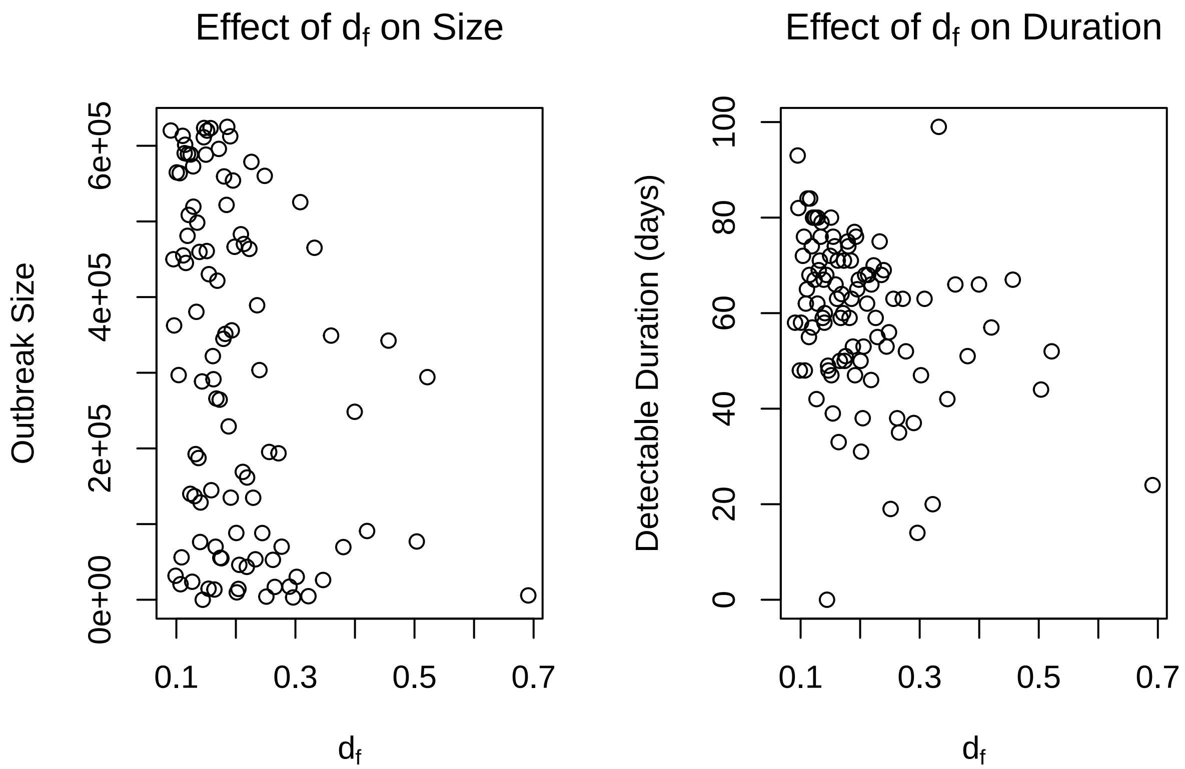


```
plot(bSIR$MaxInf ~ bSIR$beta_h, main = expression(paste("Effect of ", beta[b], " on Size")),
    xlab = expression(beta[b]), ylab = "Outbreak Size")
plot(bSIR$Thresh100 ~ bSIR$beta_h, main = expression(paste("Effect of ", beta[b],
    " on Duration")), xlab = expression(beta[b]), ylab = "Detectable Duration (days)")
```

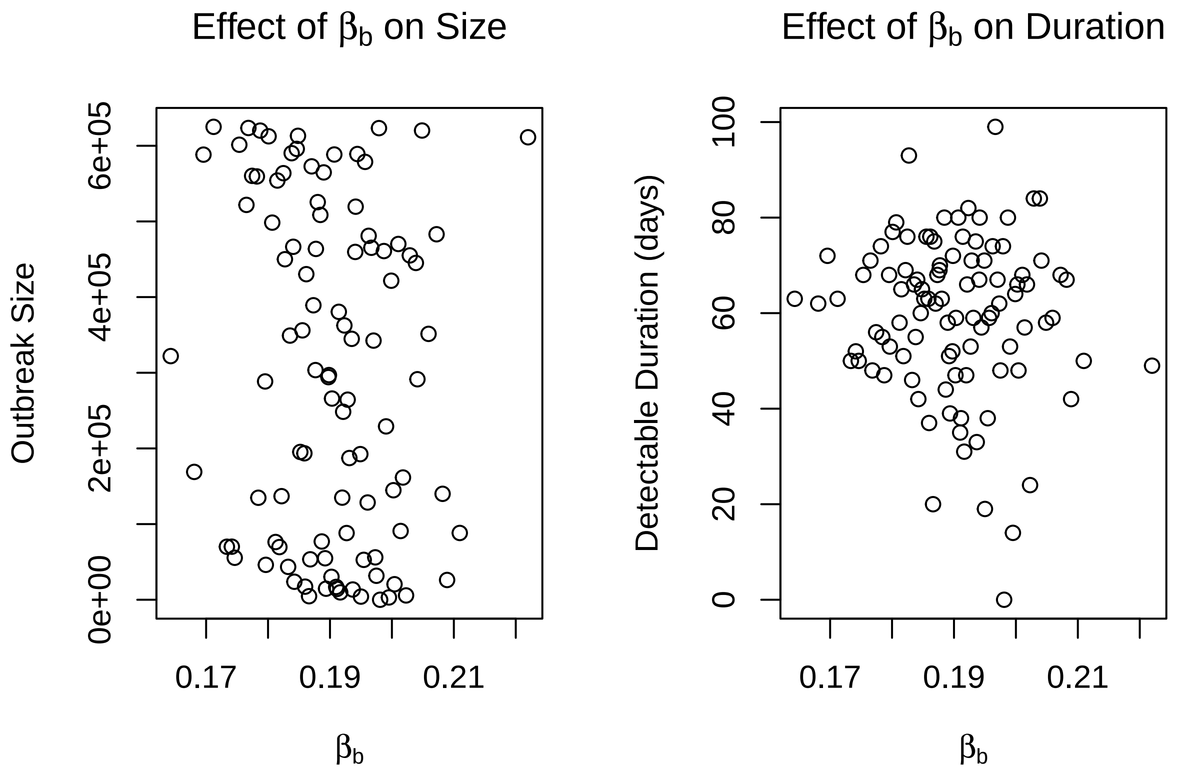


```
plot(bSIR$MaxInf ~ bSIR$gamma_h, main = expression(paste("Effect of ", gamma[b],
    " on Size")), xlab = expression(gamma[b]), ylab = "Outbreak Size")
plot(bSIR$Thresh100 ~ bSIR$gamma_h, main = expression(paste("Effect of ", gamma[b],
    " on Duration")), xlab = expression(gamma[b]), ylab = "Detectable Duration (days)")
```

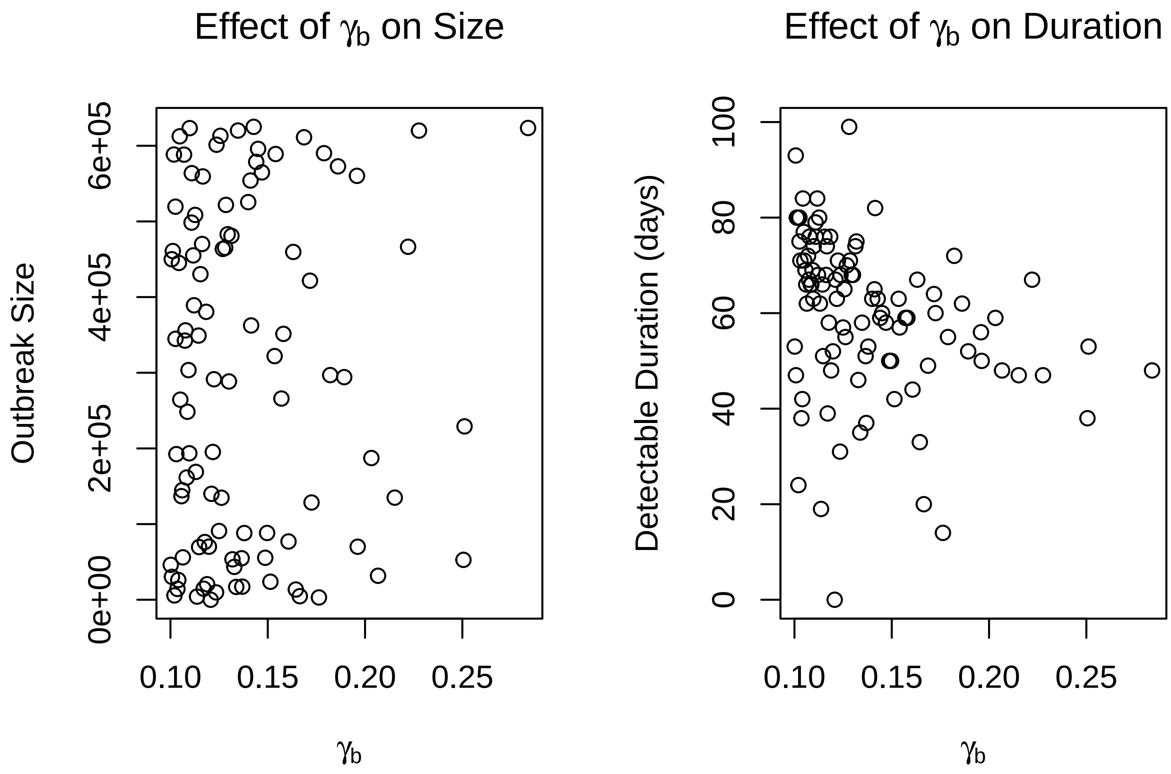


```r
plot(bSIR$MaxInf ~ bSIR$g_h, main = expression(paste("Effect of ", g[h], " on Size")),
    xlab = expression(g[h]), ylab = "Outbreak Size")
plot(bSIR$Thresh100 ~ bSIR$g_h, main = expression(paste("Effect of ", g[h], " on Duration")),
    xlab = expression(g[h]), ylab = "Detectable Duration (days)")
```

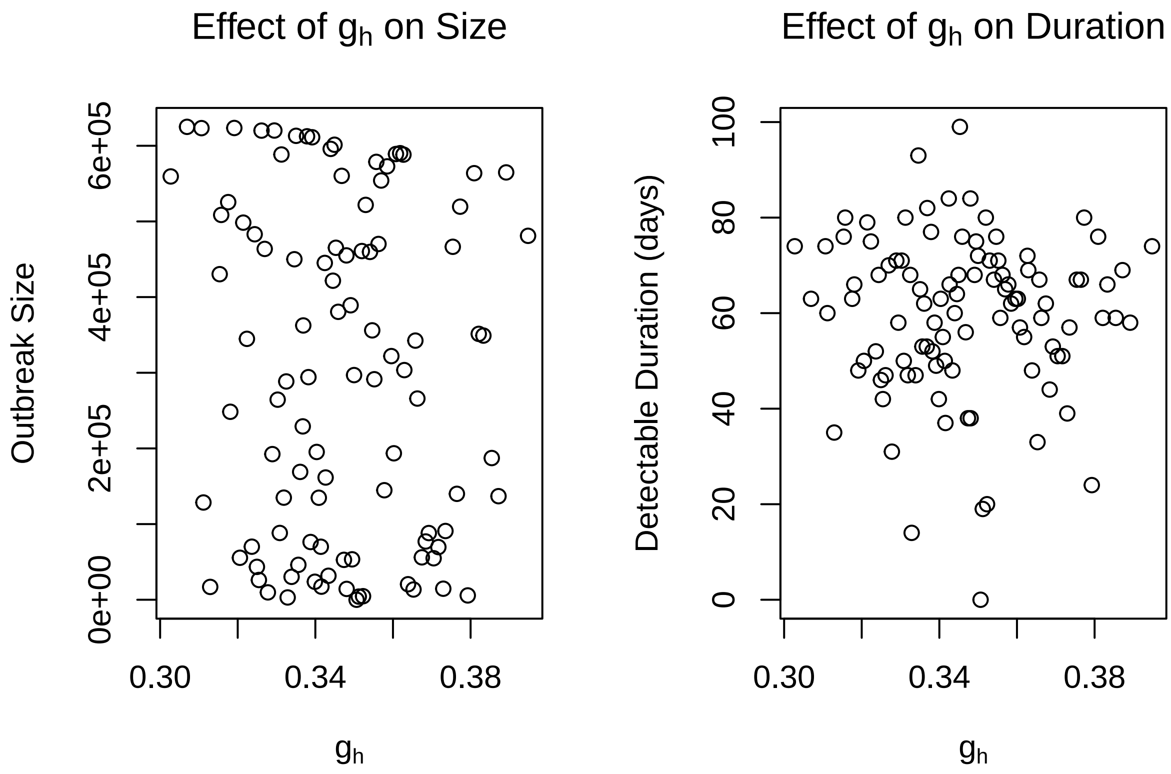


```
plot(bSIR$MaxInf ~ bSIR$b_h, main = expression(paste("Effect of ", b[h], " on Size")),
    xlab = expression(b[h]), ylab = "Outbreak Size")
plot(bSIR$Thresh100 ~ bSIR$b_h, main = expression(paste("Effect of ", b[h], " on Duration")),
    xlab = expression(b[h]), ylab = "Detectable Duration (days)")
```

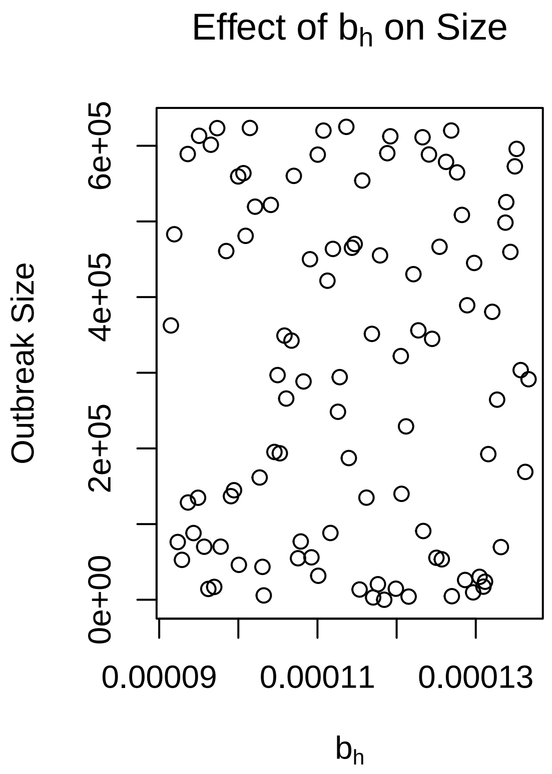

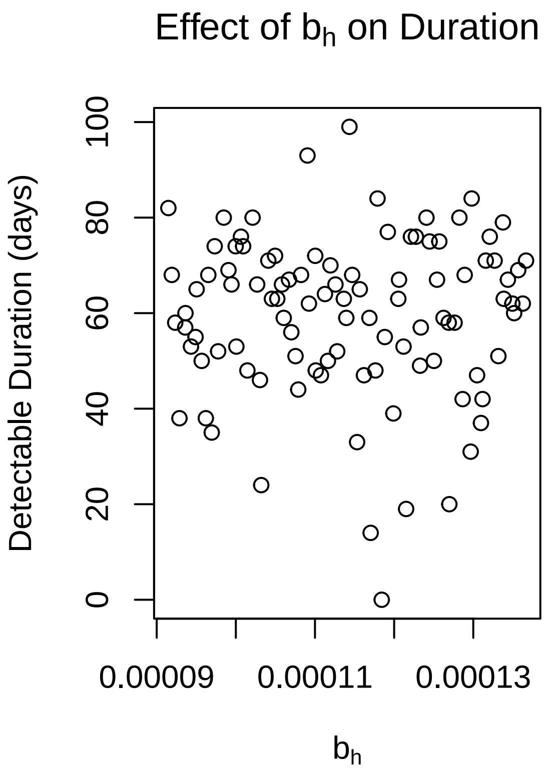


```
plot(bSIR$MaxInf ~ bSIR$d_h, main = expression(paste("Effect of ", d[h], " on Size")),
    xlab = expression(d[h]), ylab = "Outbreak Size")
plot(bSIR$Thresh100 ~ bSIR$d_h, main = expression(paste("Effect of ", d[h], " on Duration")),
    xlab = expression(d[h]), ylab = "Detectable Duration (days)")
```

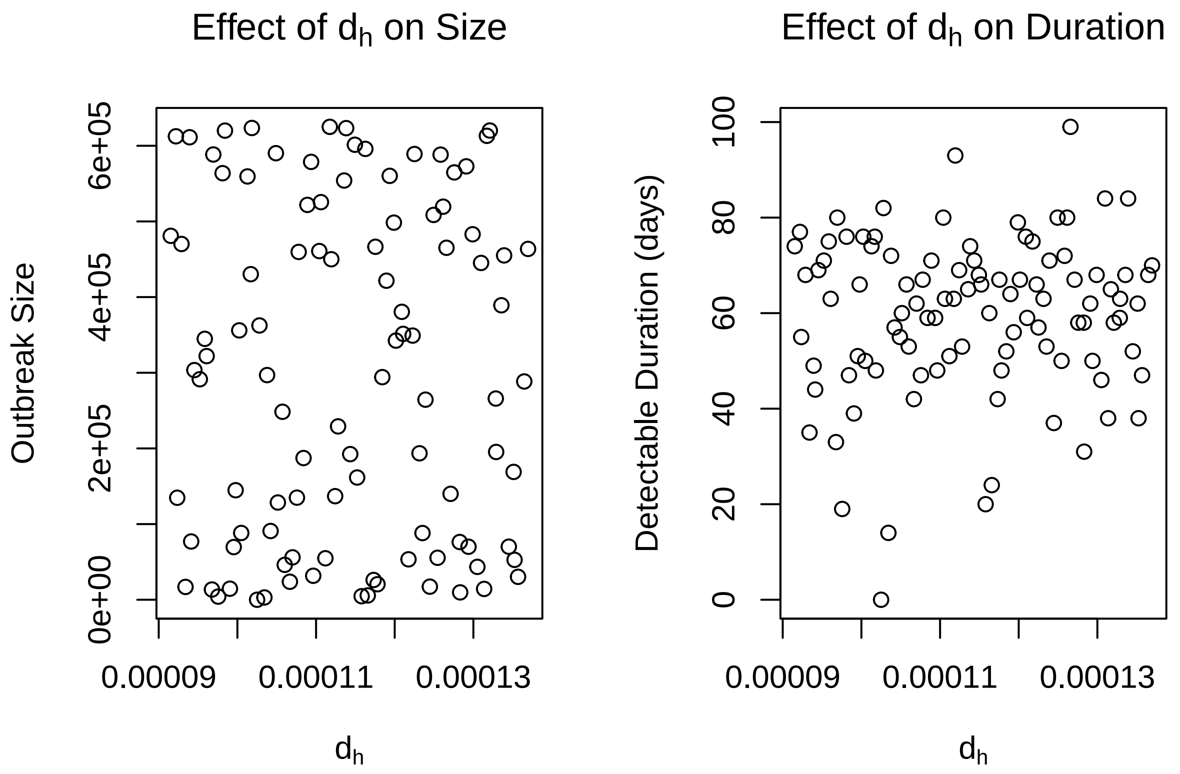


```r
par(mfrow = c(1, 2))
boxplot(bSIR$MaxInf, main = "Outbreak Size", ylab = "Number of Dead Humans", ylim = c(0,
    923406))
boxplot(bSIR$Thresh100, main = "Outbreak Duration", ylab = "Time (Days)")
```

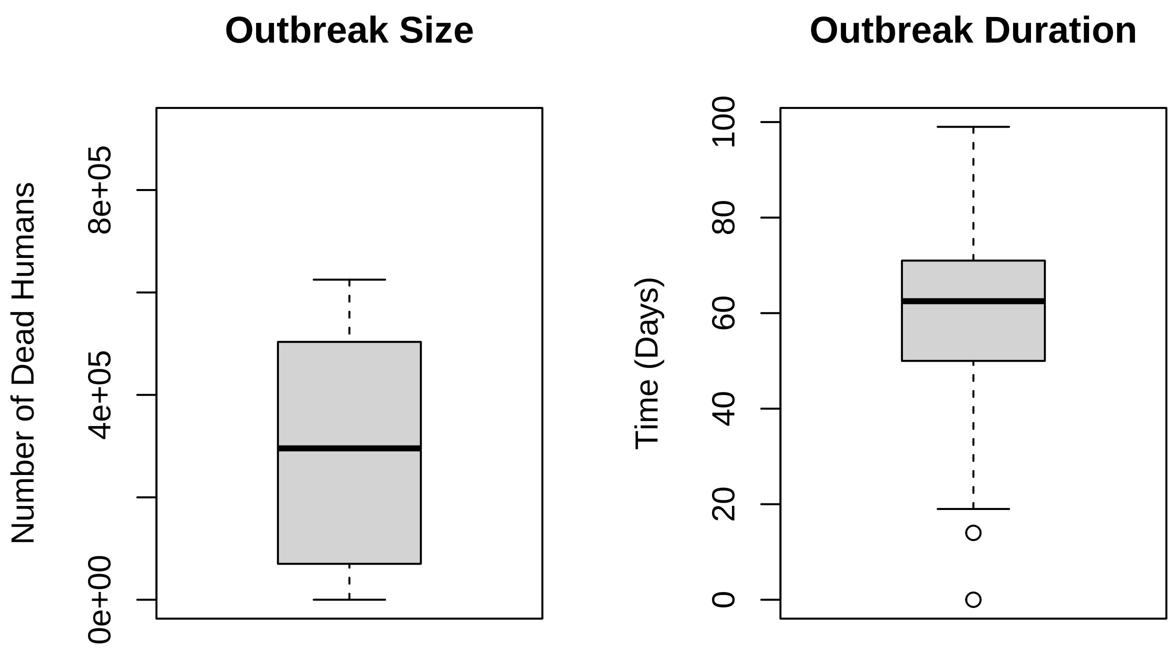


```
bonferroni.alpha <- 0.05/length(parameters)
prcc_size <- pcc(bSIR[, 1:length(parameters)], bSIR$MaxInf, nboot = niter, rank = TRUE,
    conf = 1 - bonferroni.alpha)
prcc_duration <- pcc(bSIR[, 1:length(parameters)], bSIR$Thresh100, nboot = niter,
    rank = TRUE, conf = 1 - bonferroni.alpha)

# plot correlation coefficients and confidence intervals for epidemic size and
# duration
size <- prcc_size$PRCC
size$param <- rownames(size)
colnames(size)[4:5] <- c("maxCI", "minCI")
size$maxCI[which(size$maxCI > 1)] <- 1
size$maxCI[which(size$maxCI < -1)] <- -1
size$minCI[which(size$minCI > 1)] <- 1
size$minCI[which(size$minCI < -1)] <- -1

duration <- prcc_duration$PRCC
duration$param <- rownames(duration)
colnames(duration)[4:5] <- c("maxCI", "minCI")
duration$maxCI[which(duration$maxCI > 1)] <- 1
duration$maxCI[which(duration$maxCI < -1)] <- -1
duration$minCI[which(duration$minCI > 1)] <- 1
duration$minCI[which(duration$minCI < -1)] <- -1


A <- ggplot(size, aes(x = param, y = original)) + geom_point(size = 4) + geom_errorbar(aes(ymax = maxCI
```

```
    ymin = minCI)) + ggtitle("A") + xlab("Parameters") + ylab("Partial Rank Correlation Coefficients") +
    scale_x_discrete(labels = c(alpha = expression(alpha), beta_h = expression(beta[b]),
        beta_r = expression(beta[r]), b_h = expression(b[h]), d_h = expression(d[h]),
        d_f = expression(d[f]), gamma_h = expression(gamma[b]), gamma_r = expression(gamma[r]),
        g_h = expression(g[h]), g_r = expression(g[r]), K_f = expression(K[f]), r_f = expression(r[f]))
    ylim(-1, 1)

B <- ggplot(duration, aes(x = param, y = original)) + geom_point(size = 4) + geom_errorbar(aes(ymax = ma
    ymin = minCI)) + ggtitle("B") + xlab("Parameters") + ylab(" ") + scale_x_discrete(labels = c(alpha =
    beta_h = expression(beta[b]), beta_r = expression(beta[r]), b_h = expression(b[h]),
    d_h = expression(d[h]), d_f = expression(d[f]), gamma_h = expression(gamma[b]),
    gamma_r = expression(gamma[r]), g_h = expression(g[h]), g_r = expression(g[r]),
    K_f = expression(K[f]), r_f = expression(r[f]))) + ylim(-1, 1)
```

Bubonic SEIR - Figure S4 (Panels C & D)

```
parameters <- c(beta_r = 0.09, alpha = 3/923406, gamma_r = 1/5.15, g_r = 0.1, r_f = 0.0084,
    K_f = 6, d_f = 1/5, beta_h = 0.19, sigma_h = 1/4, gamma_h = 1/10, g_h = 0.34,
    b_h = 1/(25 * 365), d_h = 1/(25 * 365))  #you can play with transmission and recovery rates here

par(mfrow = c(1, 2))
plot(bSEIR$MaxInf ~ bSEIR$beta_r, main = expression(paste("Effect of ", beta[r],
    " on Size")), xlab = expression(beta[r]), ylab = "Outbreak Size")
plot(bSEIR$Thresh100 ~ bSEIR$beta_r, main = expression(paste("Effect of ", beta[r],
    " on Duration")), xlab = expression(beta[r]), ylab = "Detectable Duration (days)")
```

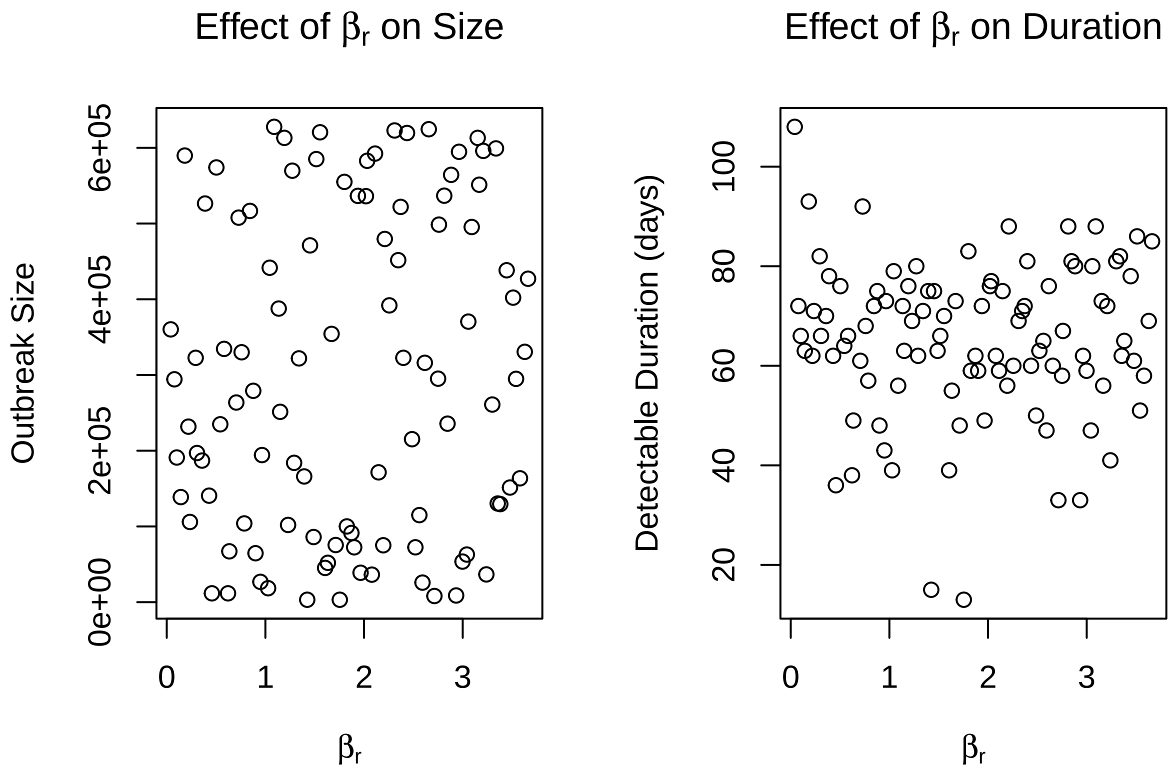


```
plot(bSEIR$MaxInf ~ bSEIR$alpha, main = expression(paste("Effect of ", alpha, " on Size")),
    xlab = expression(alpha), ylab = "Outbreak Size")
plot(bSEIR$Thresh100 ~ bSEIR$alpha, main = expression(paste("Effect of ", alpha,
    " on Duration")), xlab = expression(alpha), ylab = "Detectable Duration (days)")
```

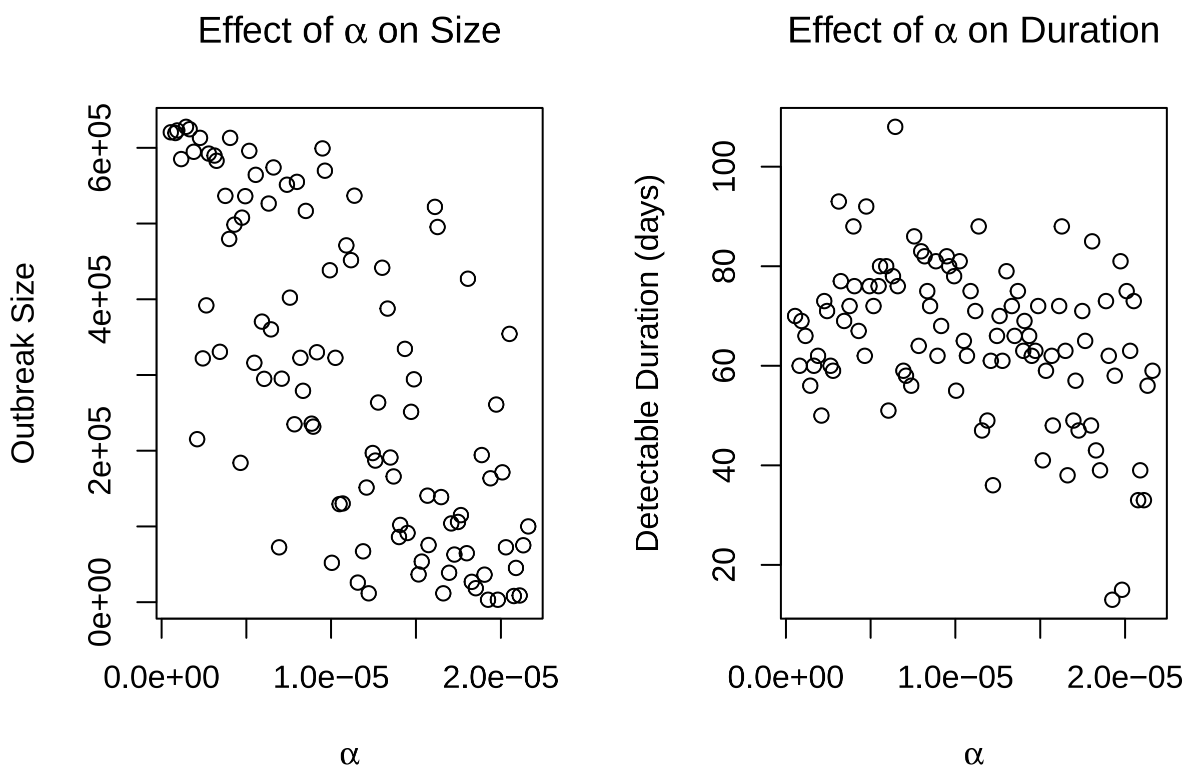


```
plot(bSEIR$MaxInf ~ bSEIR$gamma_r, main = expression(paste("Effect of ", gamma[r],
    " on Size")), xlab = expression(gamma[r]), ylab = "Outbreak Size")
plot(bSEIR$Thresh100 ~ bSEIR$gamma_r, main = expression(paste("Effect of ", gamma[r],
    " on Duration")), xlab = expression(gamma[r]), ylab = "Detectable Duration (days)")
```

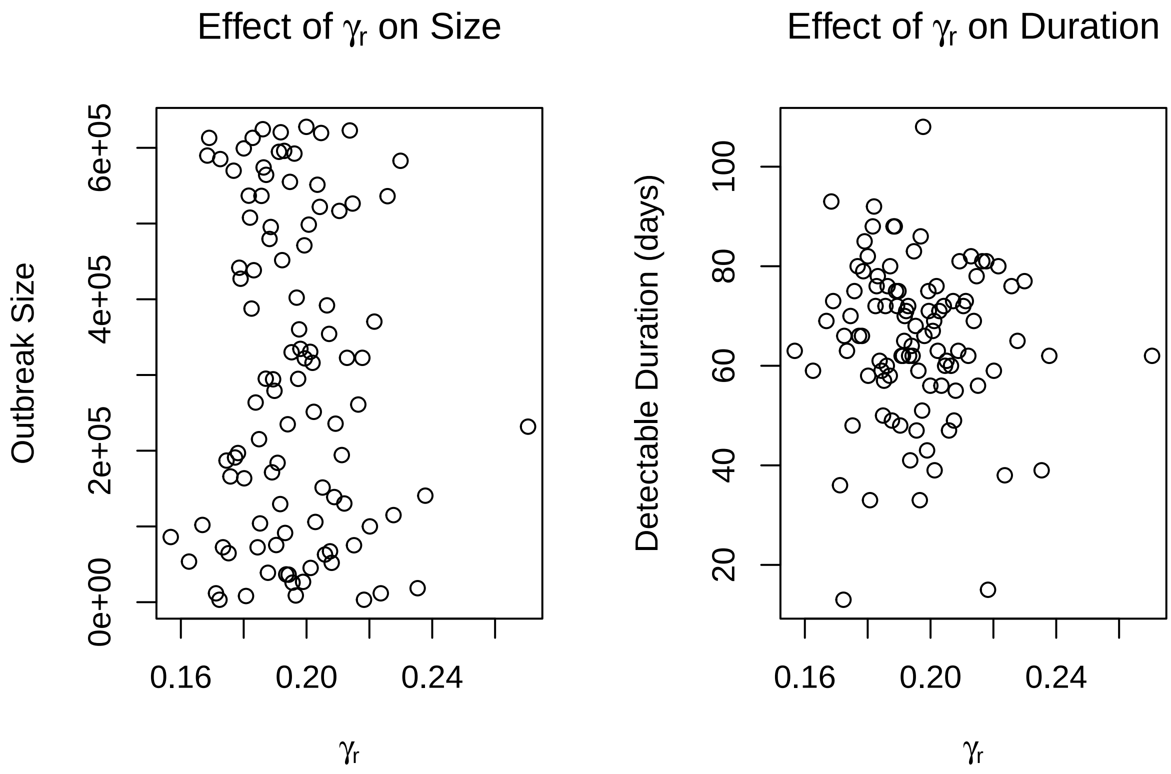


```
plot(bSEIR$MaxInf ~ bSEIR$g_r, main = expression(paste("Effect of ", g[r], " on Size")),
    xlab = expression(g[r]), ylab = "Outbreak Size")
plot(bSEIR$Thresh100 ~ bSEIR$g_r, main = expression(paste("Effect of ", g[r], " on Duration")),
    xlab = expression(g[r]), ylab = "Detectable Duration (days)")
```

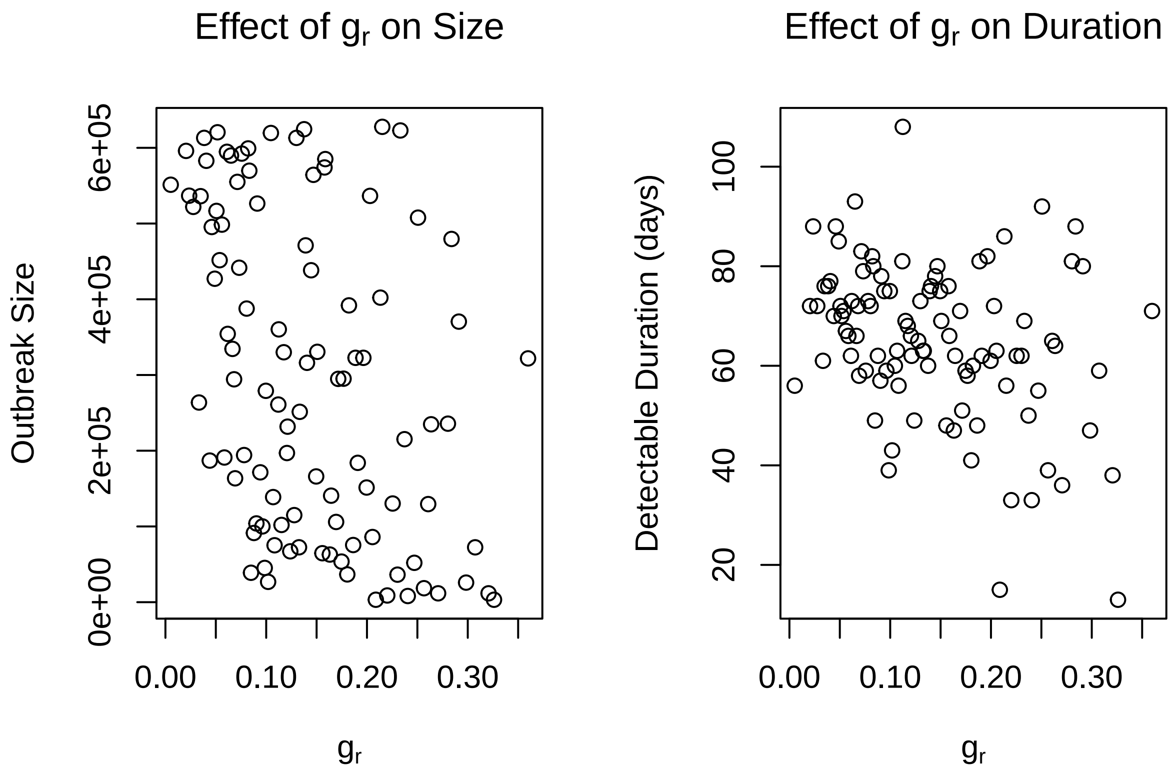


```
plot(bSEIR$MaxInf ~ bSEIR$r_f, main = expression(paste("Effect of ", r[f], " on Size")),
    xlab = expression(r[f]), ylab = "Outbreak Size")
plot(bSEIR$Thresh100 ~ bSEIR$r_f, main = expression(paste("Effect of ", r[f], " on Duration")),
    xlab = expression(r[f]), ylab = "Detectable Duration (days)")
```

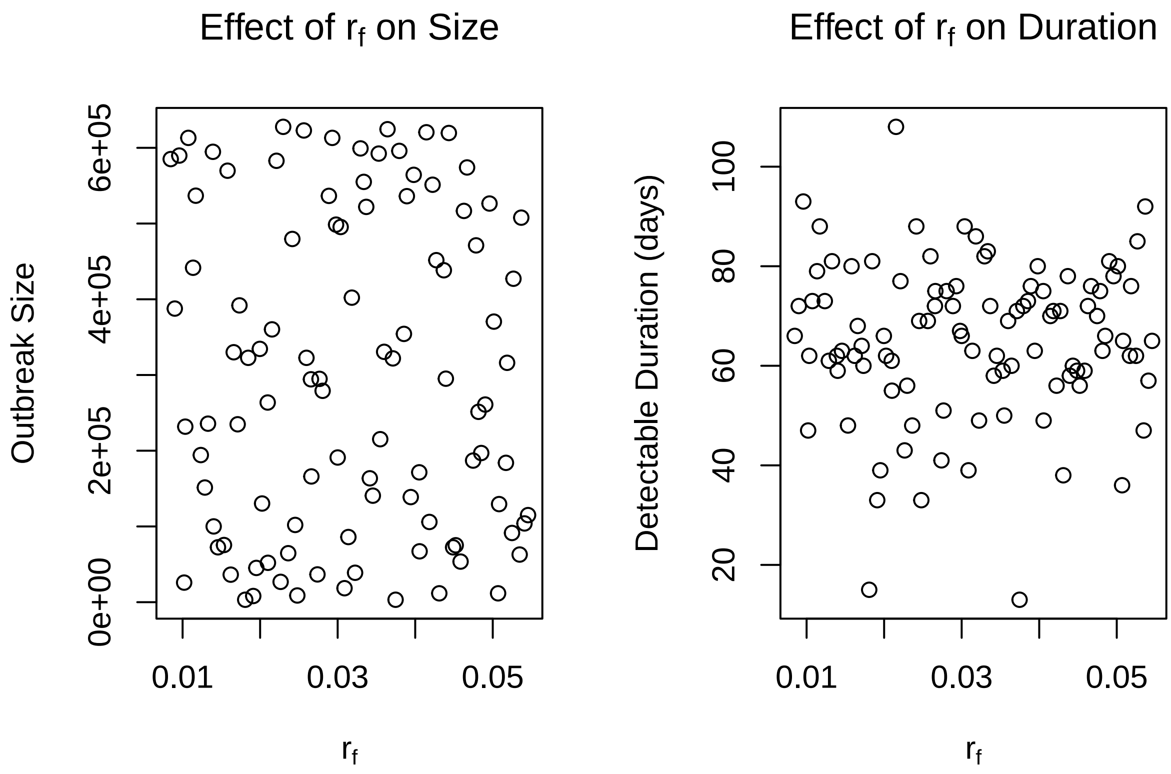


```
plot(bSEIR$MaxInf ~ bSEIR$K_f, main = expression(paste("Effect of ", K[f], " on Size")),
    xlab = expression(K[f]), ylab = "Outbreak Size")
plot(bSEIR$Thresh100 ~ bSEIR$K_f, main = expression(paste("Effect of ", K[f], " on Duration")),
    xlab = expression(K[f]), ylab = "Detectable Duration (days)")
```

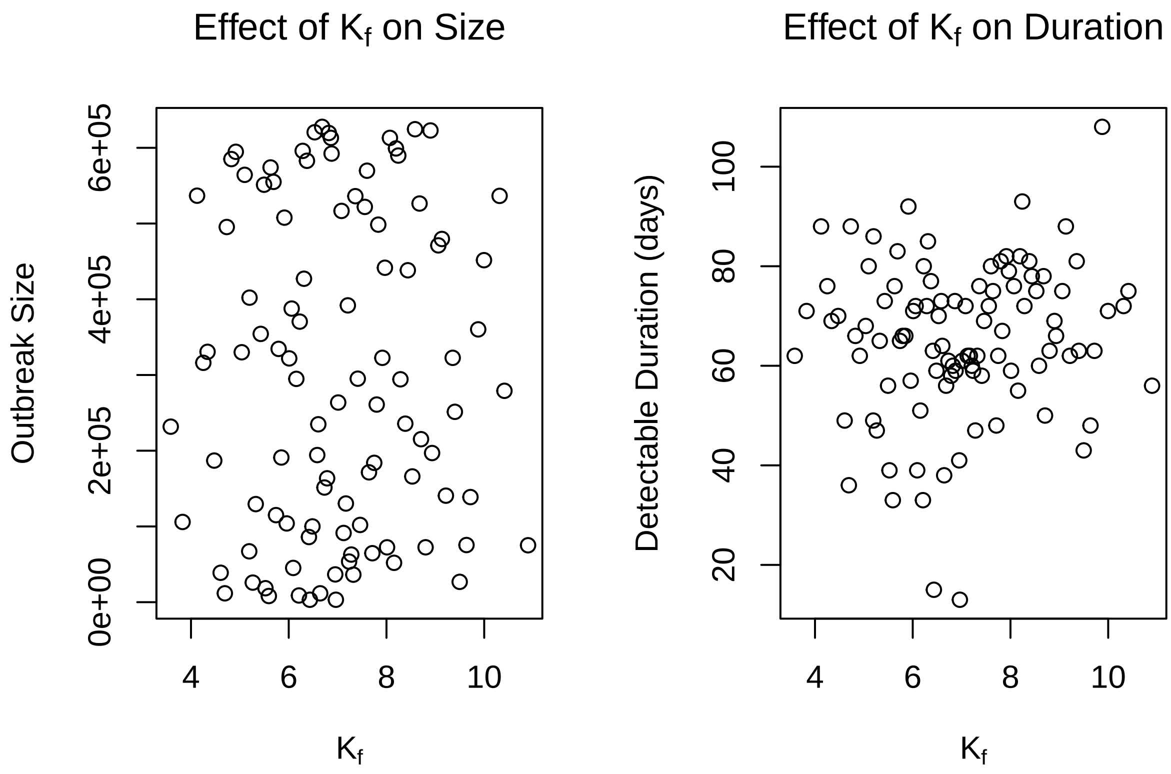


```
plot(bSEIR$MaxInf ~ bSEIR$d_f, main = expression(paste("Effect of ", d[f], " on Size")),
    xlab = expression(d[f]), ylab = "Outbreak Size")
plot(bSEIR$Thresh100 ~ bSEIR$d_f, main = expression(paste("Effect of ", d[f], " on Duration")),
    xlab = expression(d[f]), ylab = "Detectable Duration (days)")
```

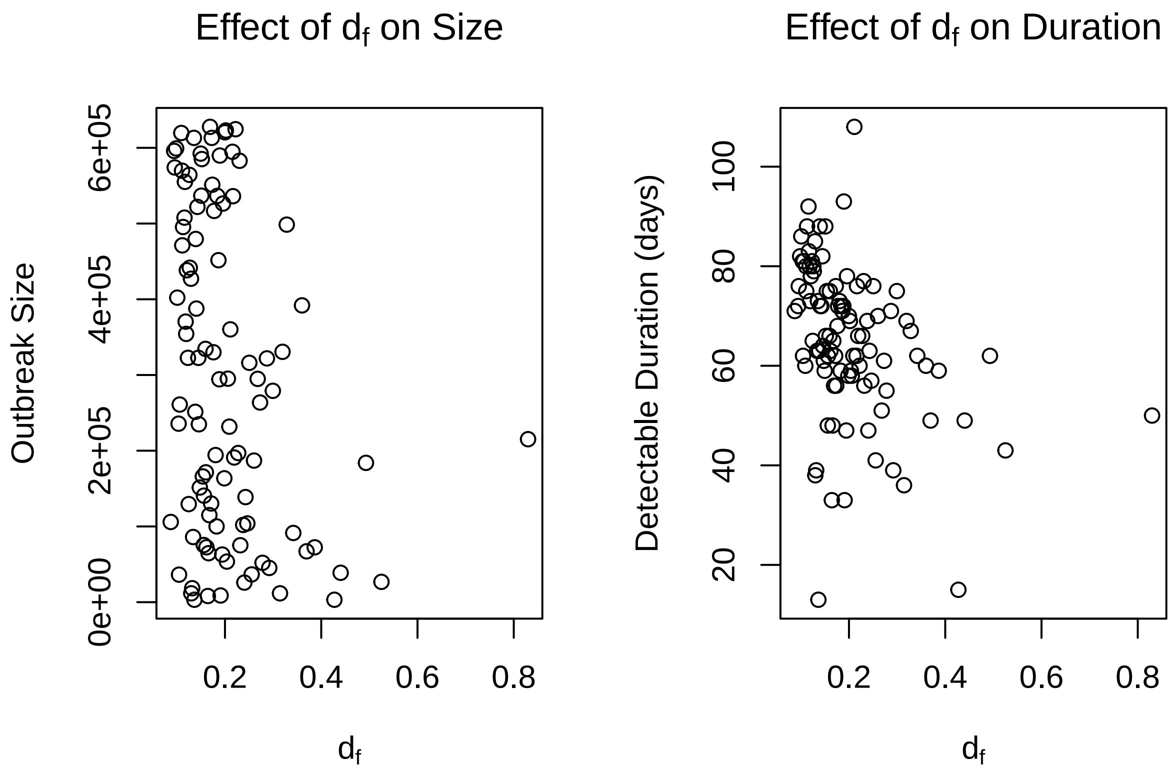


```
plot(bSEIR$MaxInf ~ bSEIR$beta_h, main = expression(paste("Effect of ", beta[b],
    " on Size")), xlab = expression(beta[b]), ylab = "Outbreak Size")
plot(bSEIR$Thresh100 ~ bSEIR$beta_h, main = expression(paste("Effect of ", beta[b],
    " on Duration")), xlab = expression(beta[b]), ylab = "Detectable Duration (days)")
```

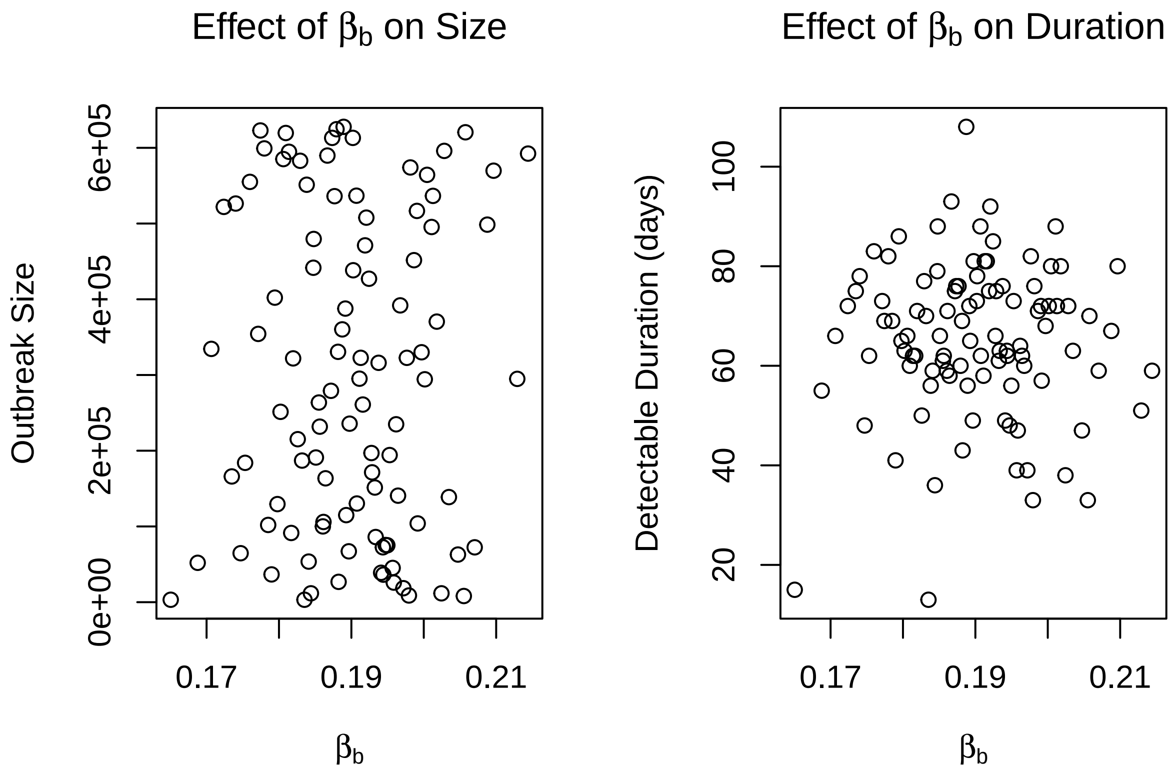


```
plot(bSEIR$MaxInf ~ bSEIR$gamma_h, main = expression(paste("Effect of ", gamma[b],
    " on Size")), xlab = expression(gamma[b]), ylab = "Outbreak Size")
plot(bSEIR$Thresh100 ~ bSEIR$gamma_h, main = expression(paste("Effect of ", gamma[b],
    " on Duration")), xlab = expression(gamma[b]), ylab = "Detectable Duration (days)")
```

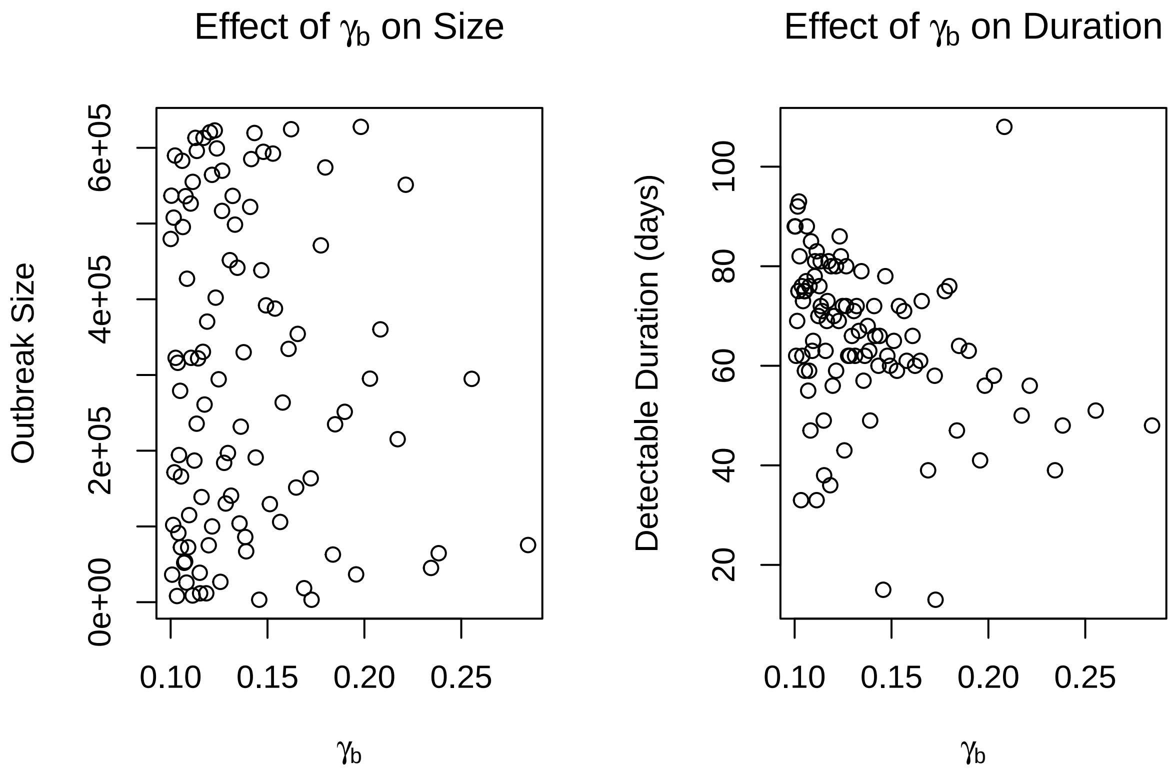


```
plot(bSEIR$MaxInf ~ bSEIR$g_h, main = expression(paste("Effect of ", g[h], " on Size")),
    xlab = expression(g[h]), ylab = "Outbreak Size")
plot(bSEIR$Thresh100 ~ bSEIR$g_h, main = expression(paste("Effect of ", g[h], " on Duration")),
    xlab = expression(g[h]), ylab = "Detectable Duration (days)")
```

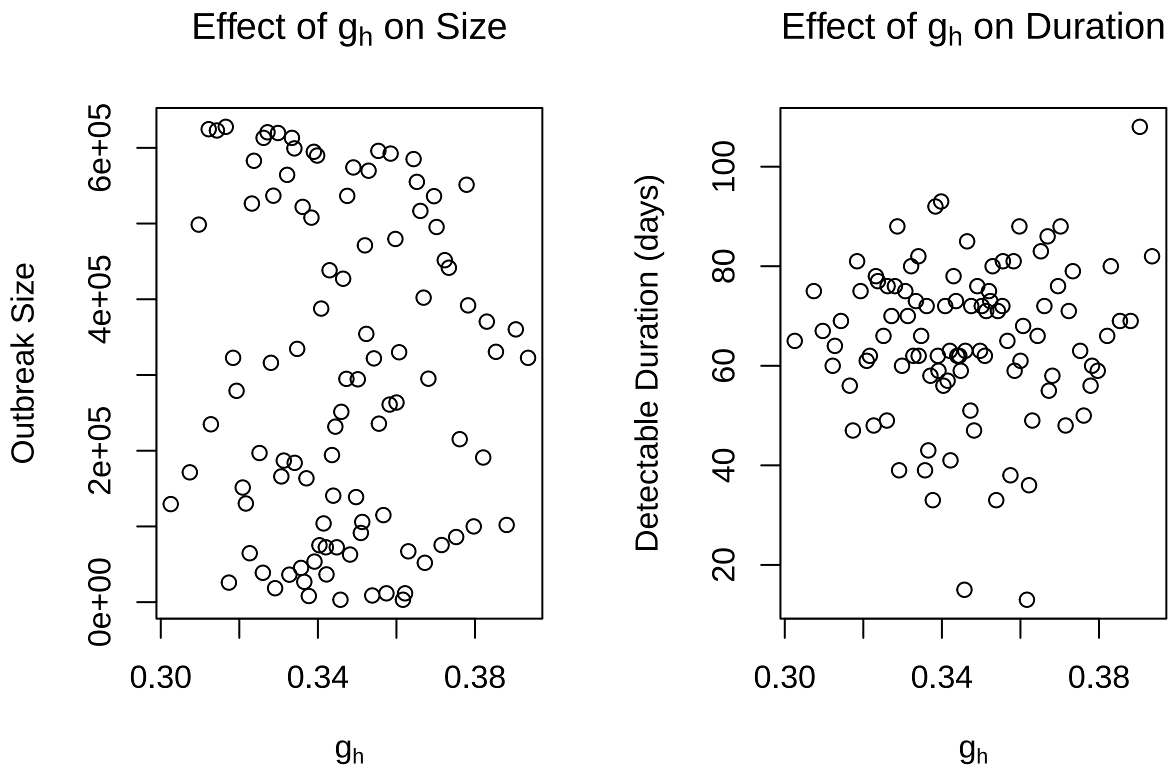


```
plot(bSEIR$MaxInf ~ bSEIR$b_h, main = expression(paste("Effect of ", b[h], " on Size")),
    xlab = expression(b[h]), ylab = "Outbreak Size")
plot(bSEIR$Thresh100 ~ bSEIR$b_h, main = expression(paste("Effect of ", b[h], " on Duration")),
    xlab = expression(b[h]), ylab = "Detectable Duration (days)")
```

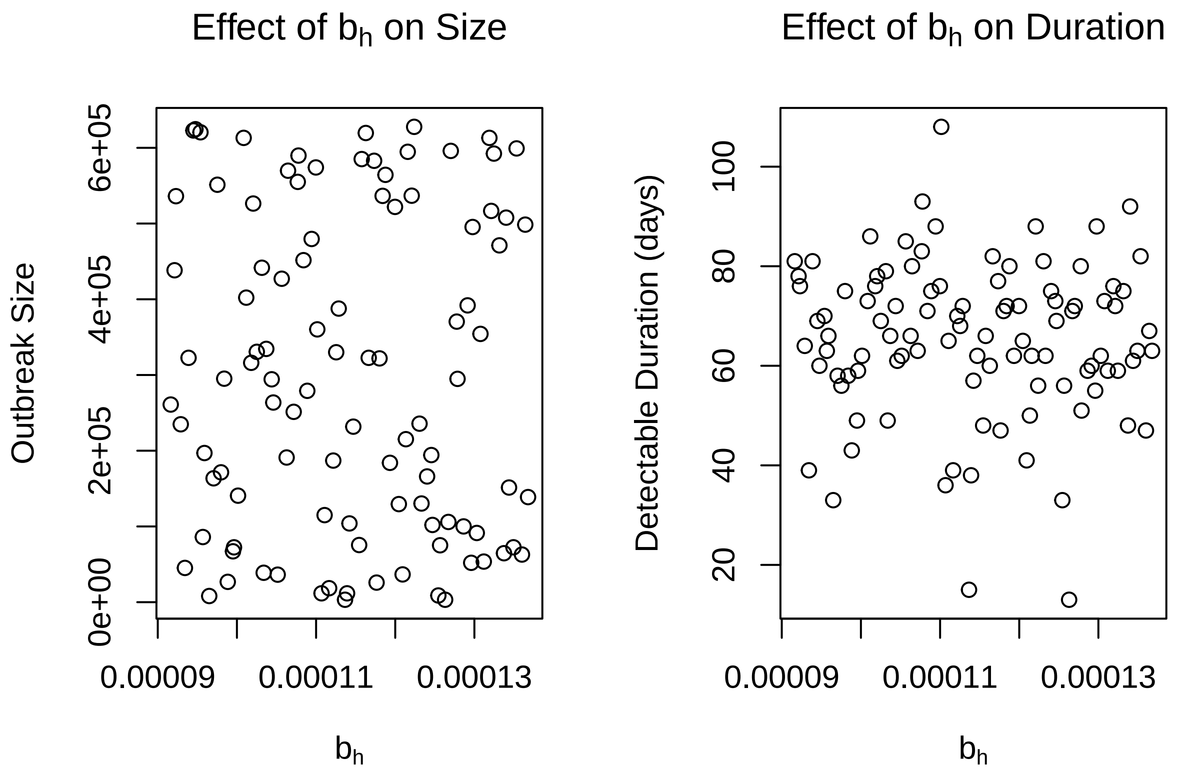


```
plot(bSEIR$MaxInf ~ bSEIR$d_h, main = expression(paste("Effect of ", d[h], " on Size")),
     xlab = expression(d[h]), ylab = "Outbreak Size")
plot(bSEIR$Thresh100 ~ bSEIR$d_h, main = expression(paste("Effect of ", d[h], " on Duration")),
     xlab = expression(d[h]), ylab = "Detectable Duration (days)")
```

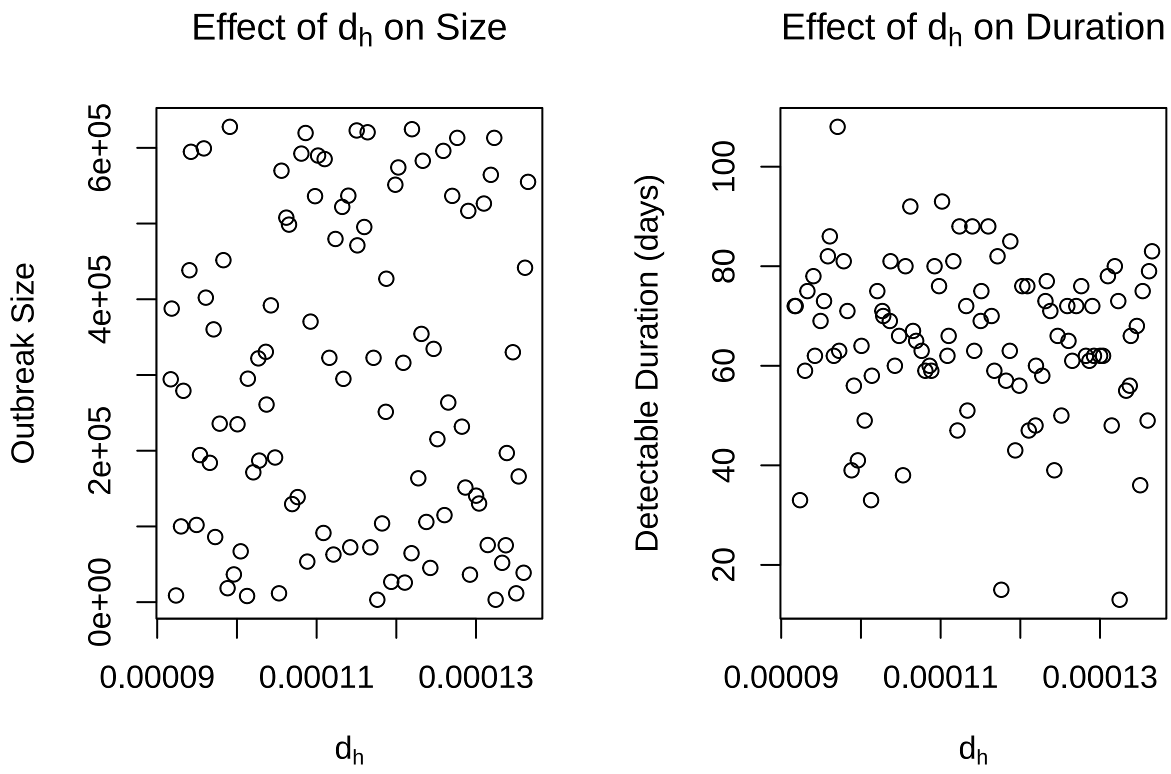


```
plot(bSEIR$MaxInf ~ bSEIR$sigma_h, main = expression(paste("Effect of ", sigma[b],
    " on Size")), xlab = expression(sigma[b]), ylab = "Outbreak Size")
plot(bSEIR$Thresh100 ~ bSEIR$sigma_h, main = expression(paste("Effect of ", sigma[b],
    " on Duration")), xlab = expression(sigma[b]), ylab = "Detectable Duration (days)")
```

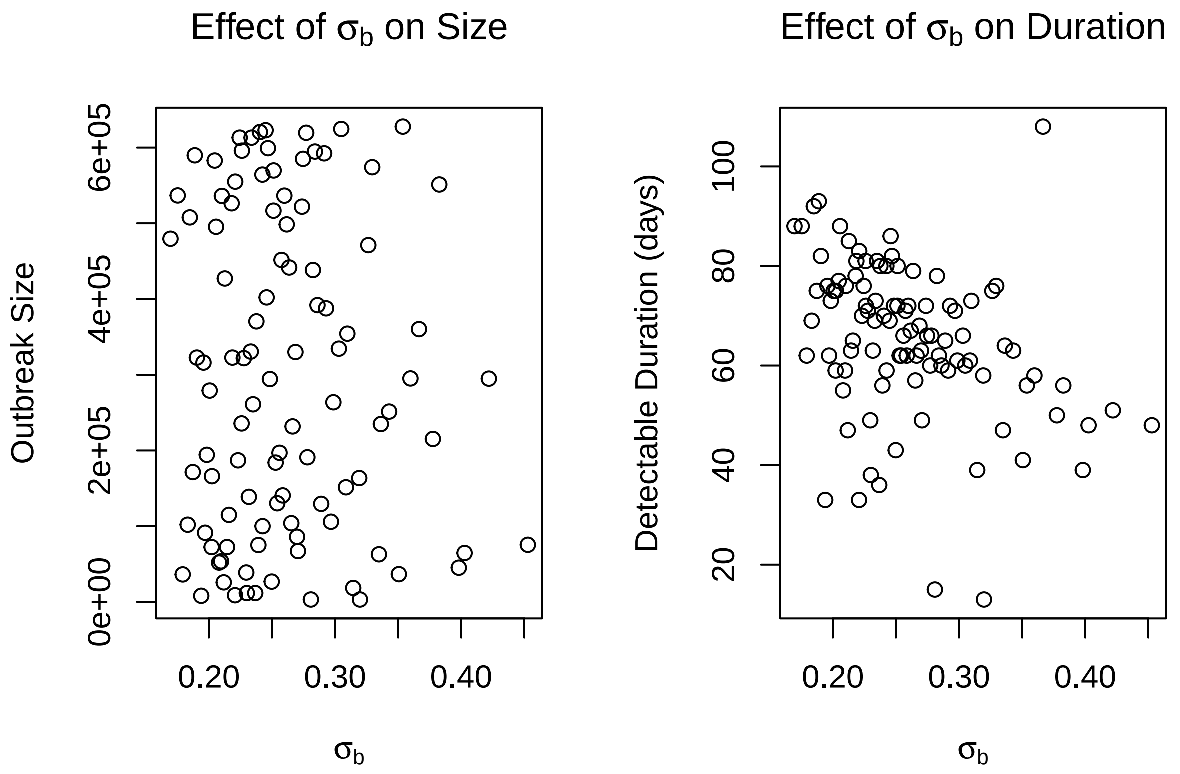


```
par(mfrow = c(1, 2))
boxplot(bSEIR$MaxInf, main = "Outbreak Size", ylab = "Number of Dead Humans", ylim = c(0,
    923406))
boxplot(bSEIR$Thresh100, main = "Outbreak Duration", ylab = "Time (Days)")
```

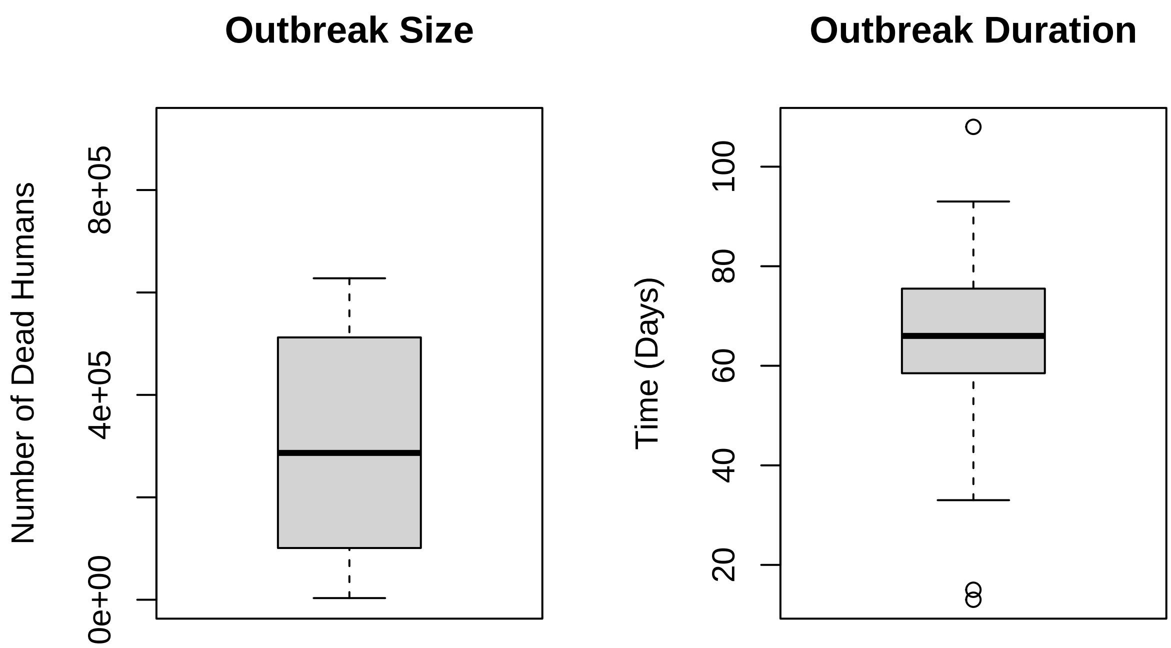


```
bonferroni.alpha <- 0.05/length(parameters)
prcc_size <- pcc(bSEIR[, 1:length(parameters)], bSEIR$MaxInf, nboot = niter, rank = TRUE,
    conf = 1 - bonferroni.alpha)
prcc_duration <- pcc(bSEIR[, 1:length(parameters)], bSEIR$Thresh100, nboot = niter,
    rank = TRUE, conf = 1 - bonferroni.alpha)

# plot correlation coefficients and confidence intervals for epidemic size and
# duration
size <- prcc_size$PRCC
size$param <- rownames(size)
colnames(size)[4:5] <- c("maxCI", "minCI")
size$maxCI[which(size$maxCI > 1)] <- 1
size$maxCI[which(size$maxCI < -1)] <- -1
size$minCI[which(size$minCI > 1)] <- 1
size$minCI[which(size$minCI < -1)] <- -1

duration <- prcc_duration$PRCC
duration$param <- rownames(duration)
colnames(duration)[4:5] <- c("maxCI", "minCI")
duration$maxCI[which(duration$maxCI > 1)] <- 1
duration$maxCI[which(duration$maxCI < -1)] <- -1
duration$minCI[which(duration$minCI > 1)] <- 1
duration$minCI[which(duration$minCI < -1)] <- -1


C <- ggplot(size, aes(x = param, y = original)) + geom_point(size = 4) + geom_errorbar(aes(ymax = maxCI
```

```
    ymin = minCI)) + ggtitle("C") + xlab("Parameters") + ylab("Partial Rank Correlation Coefficients") +
    scale_x_discrete(labels = c(alpha = expression(alpha), beta_h = expression(beta[b]),
        beta_r = expression(beta[r]), b_h = expression(b[h]), d_h = expression(d[h]),
        d_f = expression(d[f]), sigma_h = expression(sigma[b]), gamma_h = expression(gamma[b]),
        gamma_r = expression(gamma[r]), g_h = expression(g[h]), g_r = expression(g[r]),
        K_f = expression(K[f]), r_f = expression(r[f]))) + ylim(-1, 1)

D <- ggplot(duration, aes(x = param, y = original)) + geom_point(size = 4) + geom_errorbar(aes(ymax = ma
    ymin = minCI)) + ggtitle("D") + xlab("Parameters") + ylab(" ") + scale_x_discrete(labels = c(alpha =
    beta_h = expression(beta[b]), beta_r = expression(beta[r]), b_h = expression(b[h]),
    d_h = expression(d[h]), d_f = expression(d[f]), sigma_h = expression(sigma[b]),
    gamma_h = expression(gamma[b]), gamma_r = expression(gamma[r]), g_h = expression(g[h]),
    g_r = expression(g[r]), K_f = expression(K[f]), r_f = expression(r[f]))) + ylim(-1,
    1)

tiff("FigS4.tiff", height = 22.23, width = 19.05, units = "cm", compression = "lzw",
    res = 1200)
multiplot(A, B, C, D, cols = 2)
dev.off()
```

```
## pdf
##   2
```

Bubonic SIR with rat carrying capacity and resistance - Figure S5 (Panels A & B)

```
parameters <- c(r_r = 0.014, K_r = 923405, p_r = 0.975, d_r = 0.00055, beta_r = 0.09,
    alpha = 3/923406, gamma_r = 1/5.15, g_r = 0.1, r_f = 0.0084, K_f = 6, d_f = 1/5,
    beta_h = 0.19, gamma_h = 1/10, g_h = 0.34, b_h = 1/(25 * 365), d_h = 1/(25 *
        365))  #you can play with transmission and recovery rates here

par(mfrow = c(1, 2))
plot(bSIRrK$MaxInf ~ bSIRrK$r_r, main = expression(paste("Effect of ", r[r], " on Size")),
    xlab = expression(r[r]), ylab = "Outbreak Size")
plot(bSIRrK$Thresh100 ~ bSIRrK$r_r, main = expression(paste("Effect of ", r[r], " on Duration")),
    xlab = expression(r[r]), ylab = "Detectable Duration (days)")
```

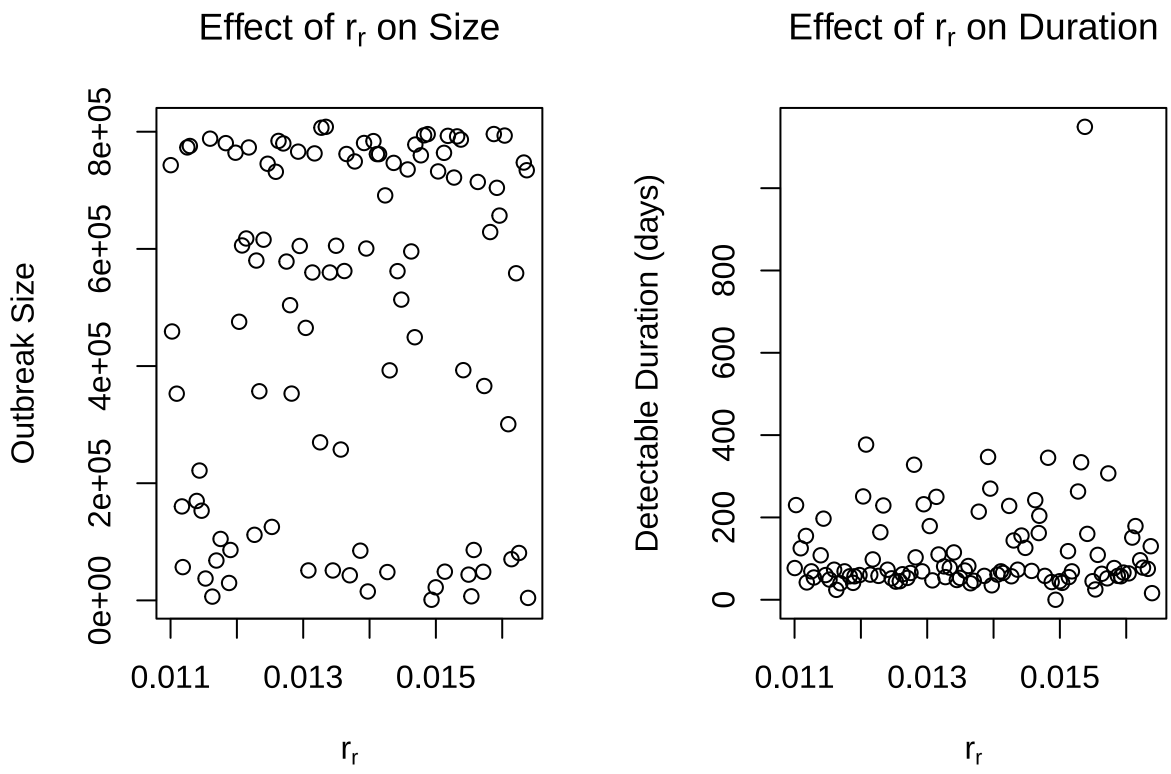


```
plot(bSIRrK$MaxInf ~ bSIRrK$K_r, main = expression(paste("Effect of ", K[r], " on Size")),
    xlab = expression(K[r]), ylab = "Outbreak Size")
plot(bSIRrK$Thresh100 ~ bSIRrK$K_r, main = expression(paste("Effect of ", K[r], " on Duration")),
    xlab = expression(K[r]), ylab = "Detectable Duration (days)")
```

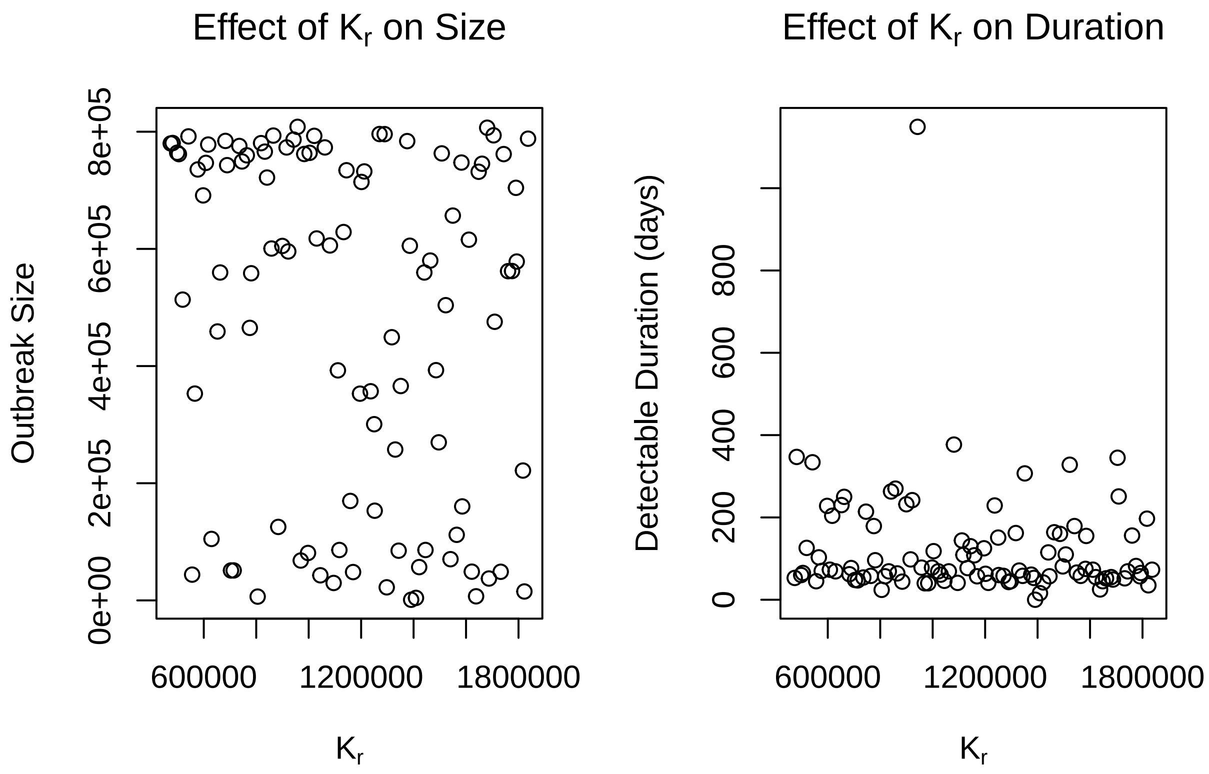


```
plot(bSIRrK$MaxInf ~ bSIRrK$p_r, main = expression(paste("Effect of ", p[r], " on Size")),
    xlab = expression(p[r]), ylab = "Outbreak Size")
plot(bSIRrK$Thresh100 ~ bSIRrK$p_r, main = expression(paste("Effect of ", p[r], " on Duration")),
    xlab = expression(p[r]), ylab = "Detectable Duration (days)")
```

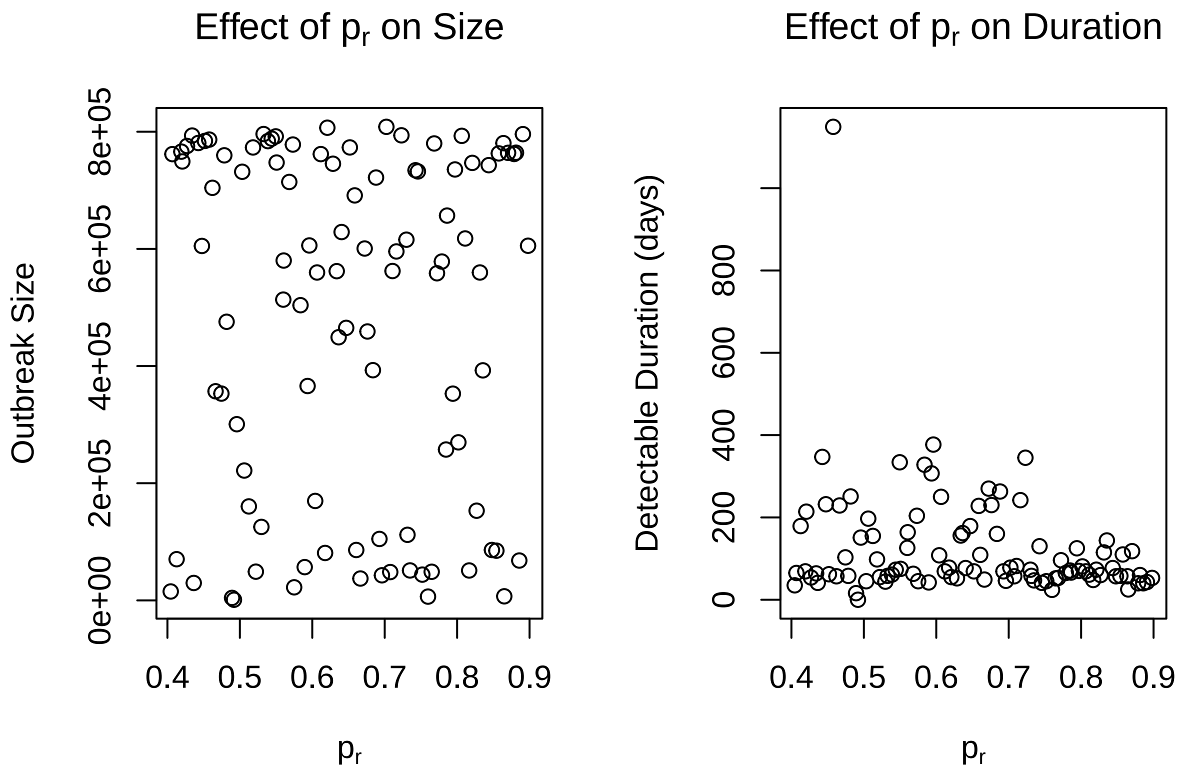


```r
plot(bSIRrK$MaxInf ~ bSIRrK$d_r, main = expression(paste("Effect of ", d[r], " on Size")),
    xlab = expression(d[r]), ylab = "Outbreak Size")
plot(bSIRrK$Thresh100 ~ bSIRrK$d_r, main = expression(paste("Effect of ", d[r], " on Duration")),
    xlab = expression(d[r]), ylab = "Detectable Duration (days)")
```

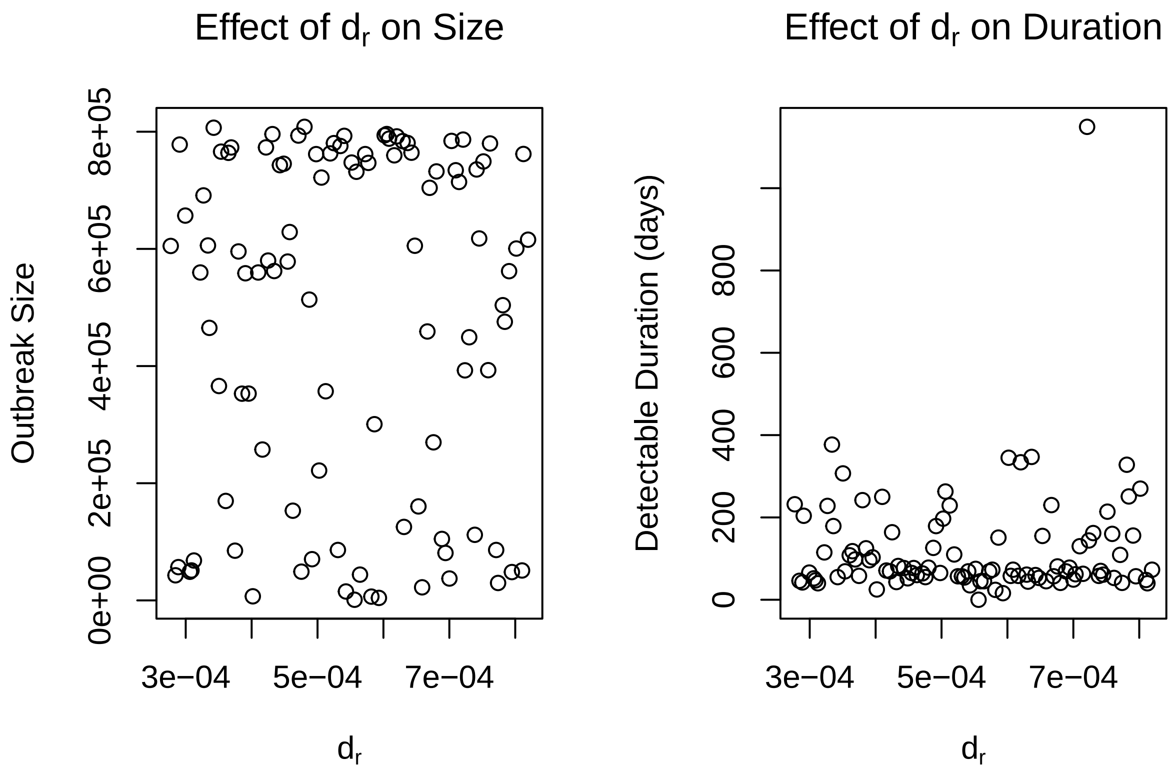


```
plot(bSIRrK$MaxInf ~ bSIRrK$beta_r, main = expression(paste("Effect of ", beta[r],
    " on Size")), xlab = expression(beta[r]), ylab = "Outbreak Size")
plot(bSIRrK$Thresh100 ~ bSIRrK$beta_r, main = expression(paste("Effect of ", beta[r],
    " on Duration")), xlab = expression(beta[r]), ylab = "Detectable Duration (days)")
```

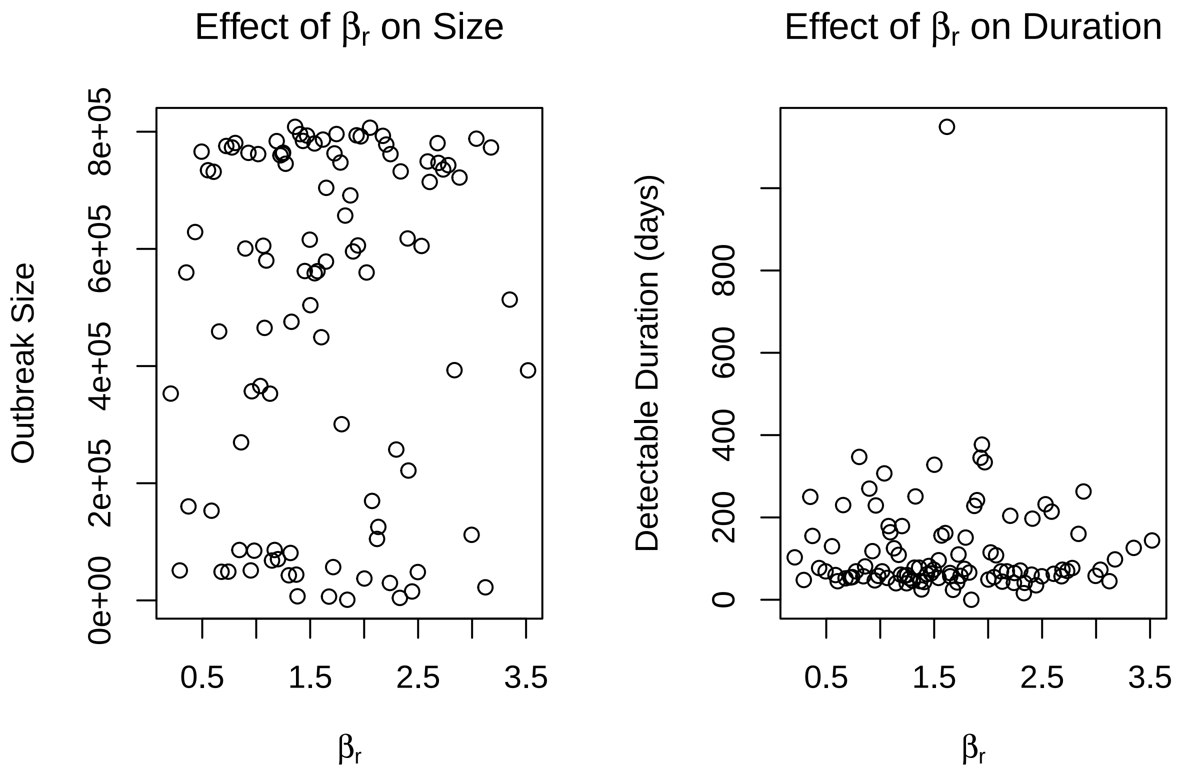


```r
plot(bSIRrK$MaxInf ~ bSIRrK$alpha, main = expression(paste("Effect of ", alpha, " on Size")),
    xlab = expression(alpha), ylab = "Outbreak Size")
plot(bSIRrK$Thresh100 ~ bSIRrK$alpha, main = expression(paste("Effect of ", alpha,
    " on Duration")), xlab = expression(alpha), ylab = "Detectable Duration (days)")
```

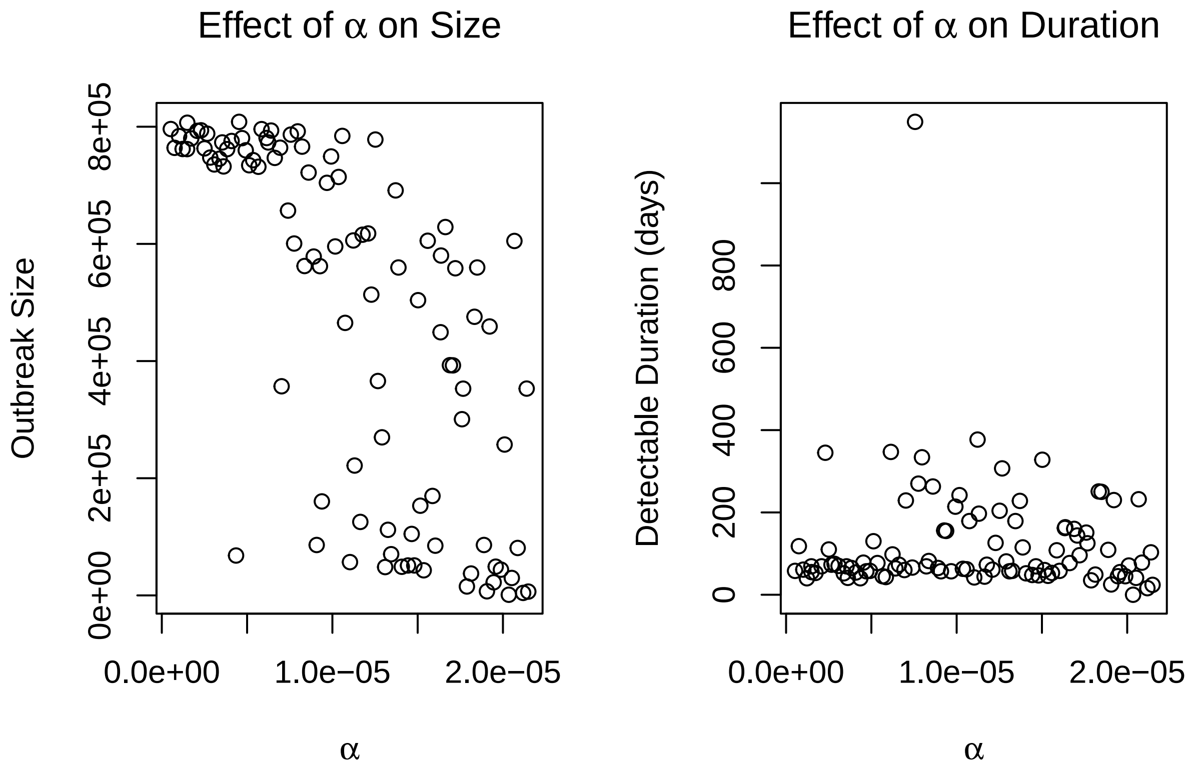


```
plot(bSIRrK$MaxInf ~ bSIRrK$gamma_r, main = expression(paste("Effect of ", gamma[r],
    " on Size")), xlab = expression(gamma[r]), ylab = "Outbreak Size")
plot(bSIRrK$Thresh100 ~ bSIRrK$gamma_r, main = expression(paste("Effect of ", gamma[r],
    " on Duration")), xlab = expression(gamma[r]), ylab = "Detectable Duration (days)")
```

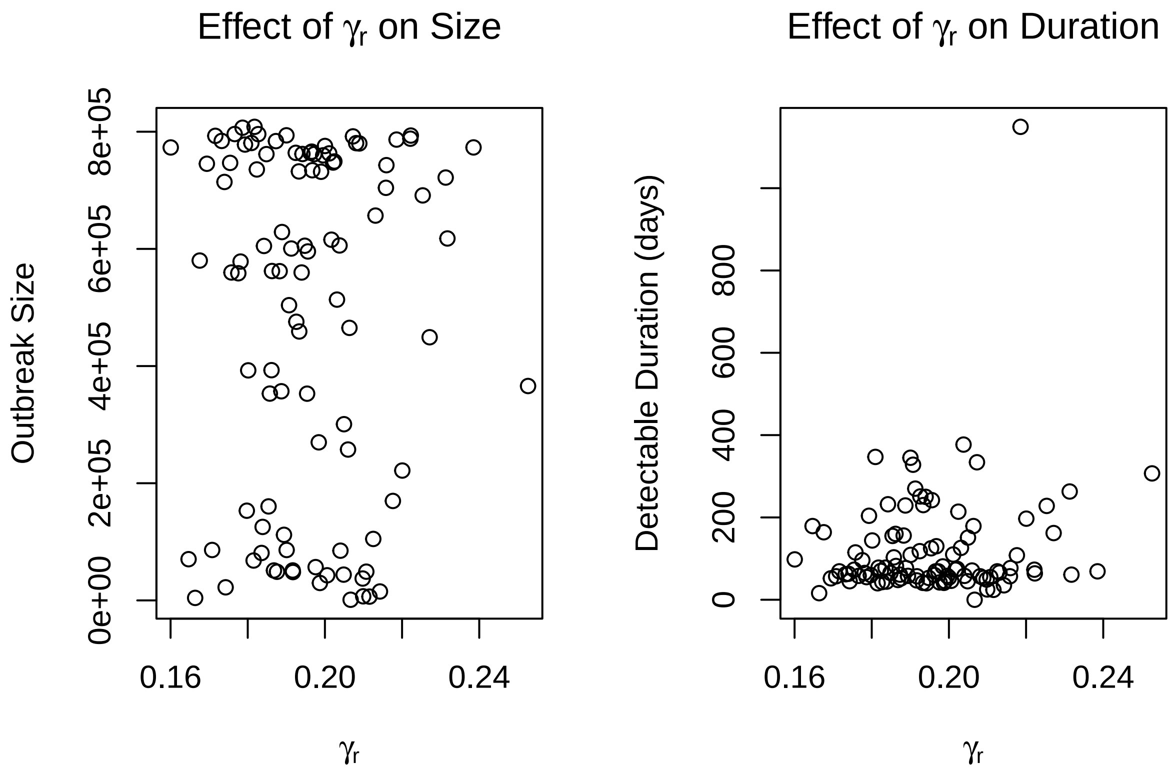


```
plot(bSIRrK$MaxInf ~ bSIRrK$g_r, main = expression(paste("Effect of ", g[r], " on Size")),
    xlab = expression(g[r]), ylab = "Outbreak Size")
plot(bSIRrK$Thresh100 ~ bSIRrK$g_r, main = expression(paste("Effect of ", g[r], " on Duration")),
    xlab = expression(g[r]), ylab = "Detectable Duration (days)")
```

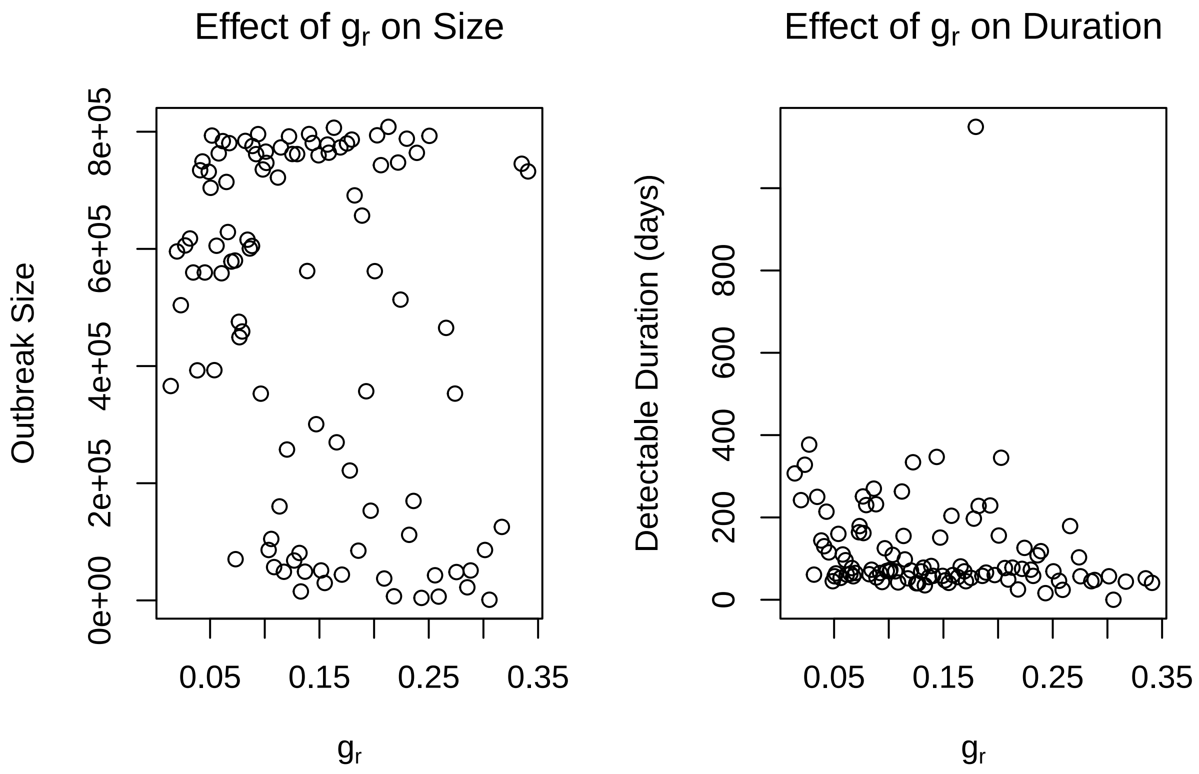


```
plot(bSIRrK$MaxInf ~ bSIRrK$r_f, main = expression(paste("Effect of ", r[f], " on Size")),
    xlab = expression(r[f]), ylab = "Outbreak Size")
plot(bSIRrK$Thresh100 ~ bSIRrK$r_f, main = expression(paste("Effect of ", r[f], " on Duration")),
    xlab = expression(r[f]), ylab = "Detectable Duration (days)")
```

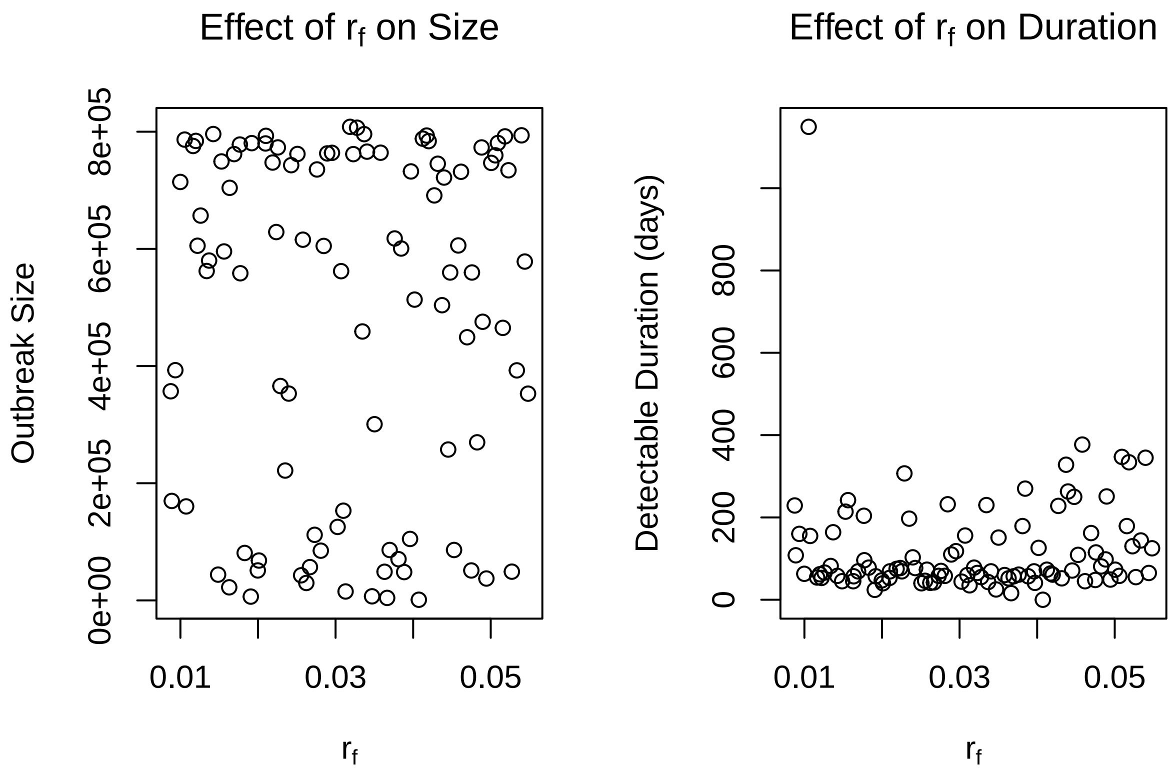


```
plot(bSIRrK$MaxInf ~ bSIRrK$K_f, main = expression(paste("Effect of ", K[f], " on Size")),
    xlab = expression(K[f]), ylab = "Outbreak Size")
plot(bSIRrK$Thresh100 ~ bSIRrK$K_f, main = expression(paste("Effect of ", K[f], " on Duration")),
    xlab = expression(K[f]), ylab = "Detectable Duration (days)")
```

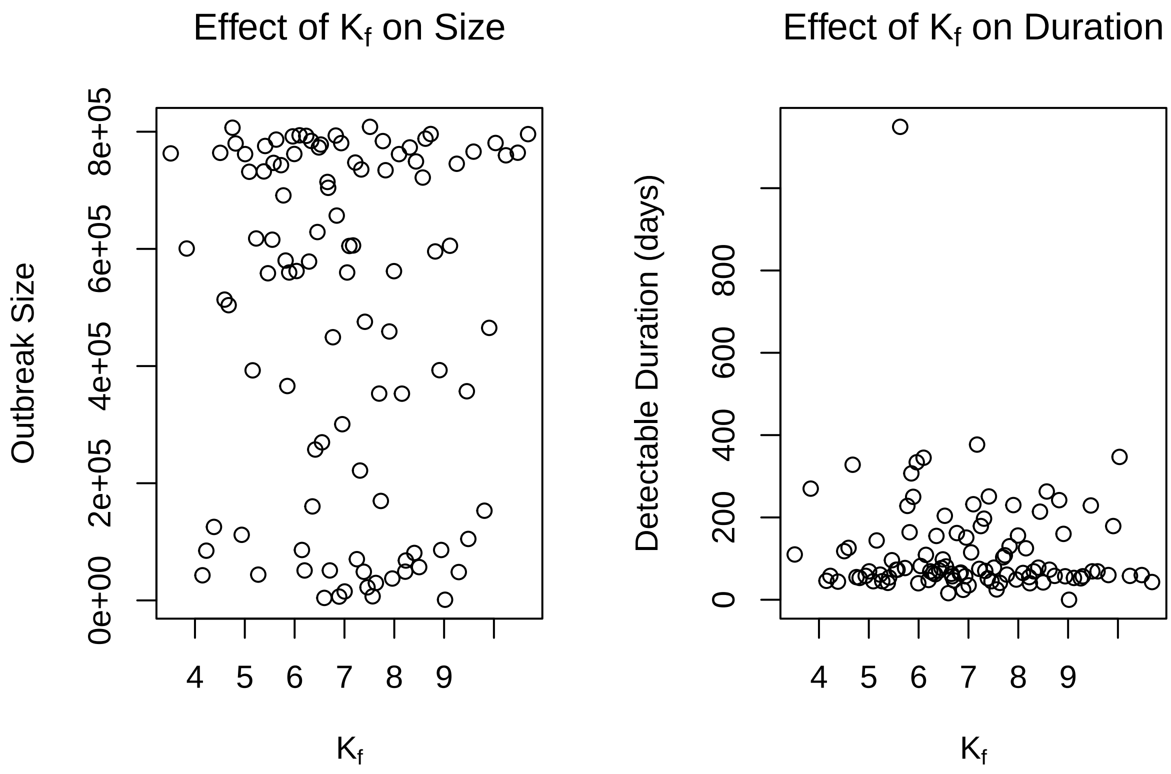


```
plot(bSIRrK$MaxInf ~ bSIRrK$d_f, main = expression(paste("Effect of ", d[f], " on Size")),
    xlab = expression(d[f]), ylab = "Outbreak Size")
plot(bSIRrK$Thresh100 ~ bSIRrK$d_f, main = expression(paste("Effect of ", d[f], " on Duration")),
    xlab = expression(d[f]), ylab = "Detectable Duration (days)")
```

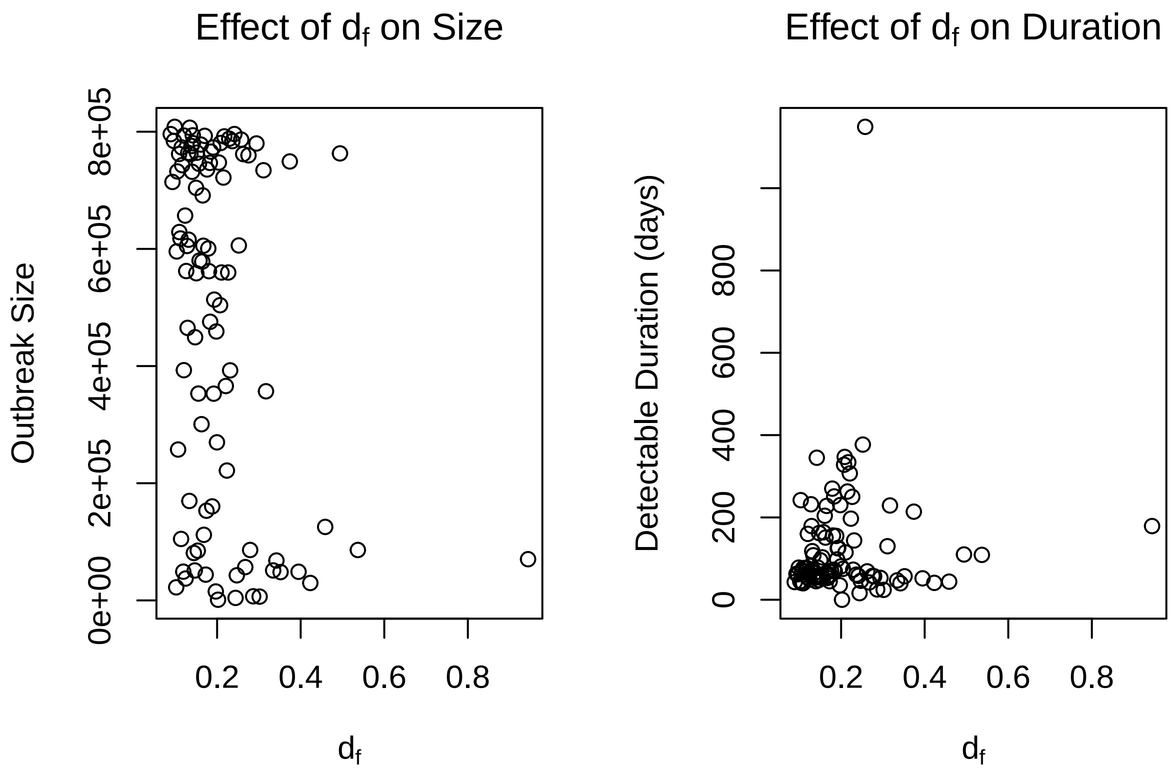


```r
plot(bSIRrK$MaxInf ~ bSIRrK$beta_h, main = expression(paste("Effect of ", beta[b],
    " on Size")), xlab = expression(beta[b]), ylab = "Outbreak Size")
plot(bSIRrK$Thresh100 ~ bSIRrK$beta_h, main = expression(paste("Effect of ", beta[b],
    " on Duration")), xlab = expression(beta[b]), ylab = "Detectable Duration (days)")
```

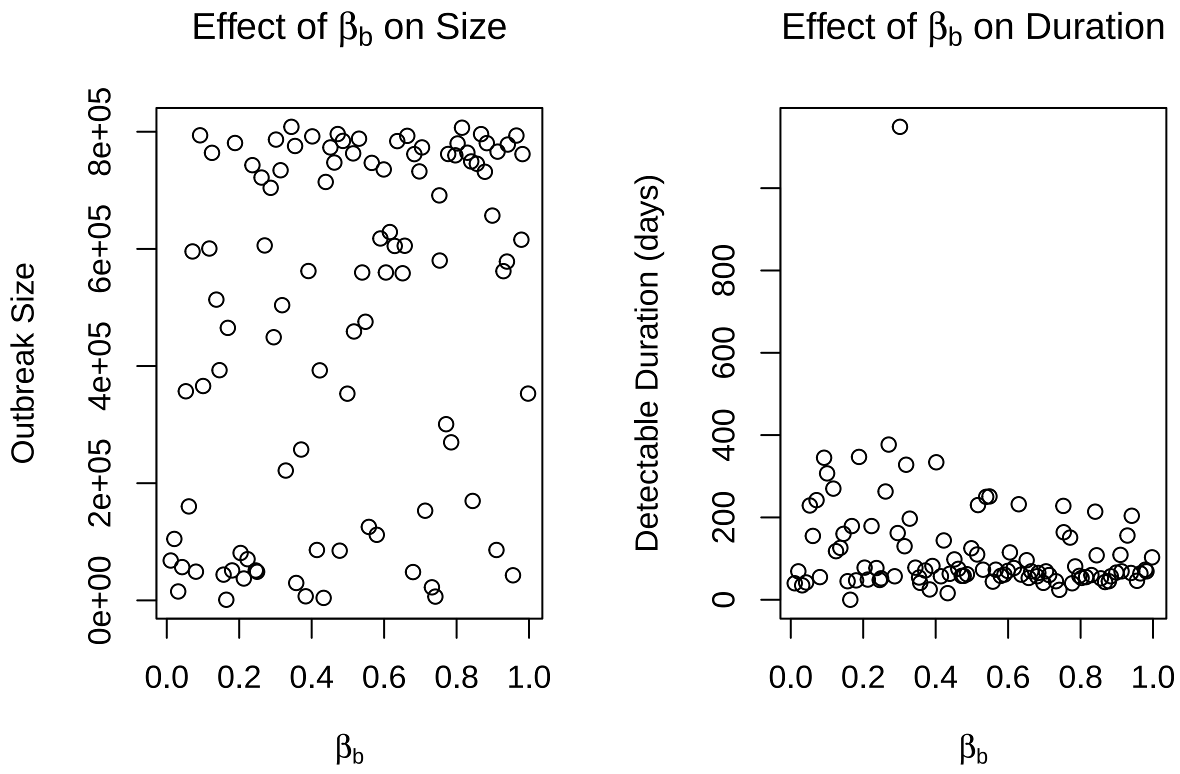


```
plot(bSIRrK$MaxInf ~ bSIRrK$gamma_h, main = expression(paste("Effect of ", gamma[b],
    " on Size")), xlab = expression(gamma[b]), ylab = "Outbreak Size")
plot(bSIRrK$Thresh100 ~ bSIRrK$gamma_h, main = expression(paste("Effect of ", gamma[b],
    " on Duration")), xlab = expression(gamma[b]), ylab = "Detectable Duration (days)")
```

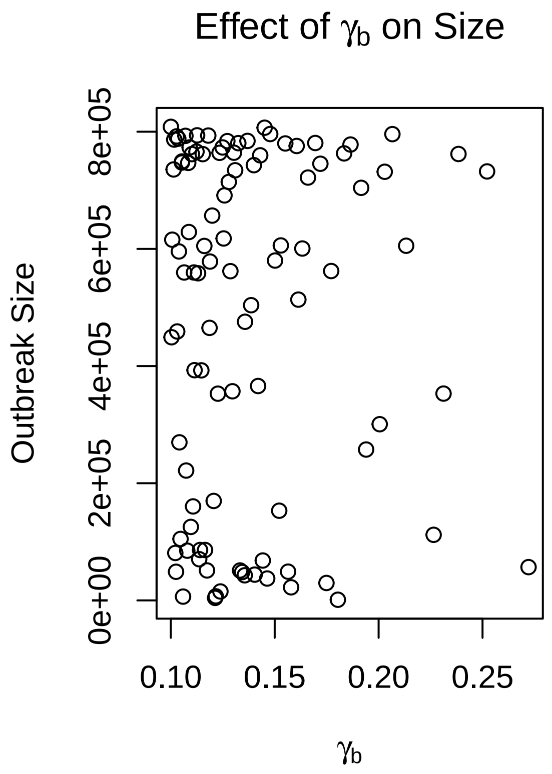

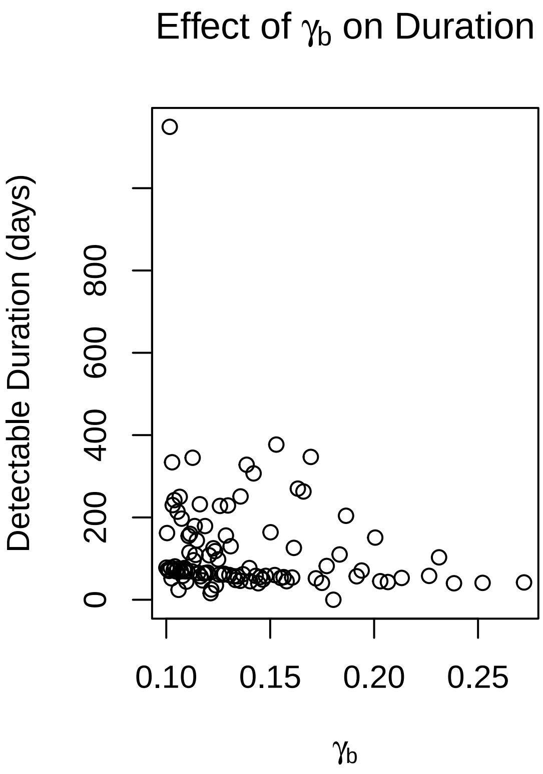


```
plot(bSIRrK$MaxInf ~ bSIRrK$g_h, main = expression(paste("Effect of ", g[h], " on Size")),
    xlab = expression(g[h]), ylab = "Outbreak Size")
plot(bSIRrK$Thresh100 ~ bSIRrK$g_h, main = expression(paste("Effect of ", g[h], " on Duration")),
    xlab = expression(g[h]), ylab = "Detectable Duration (days)")
```

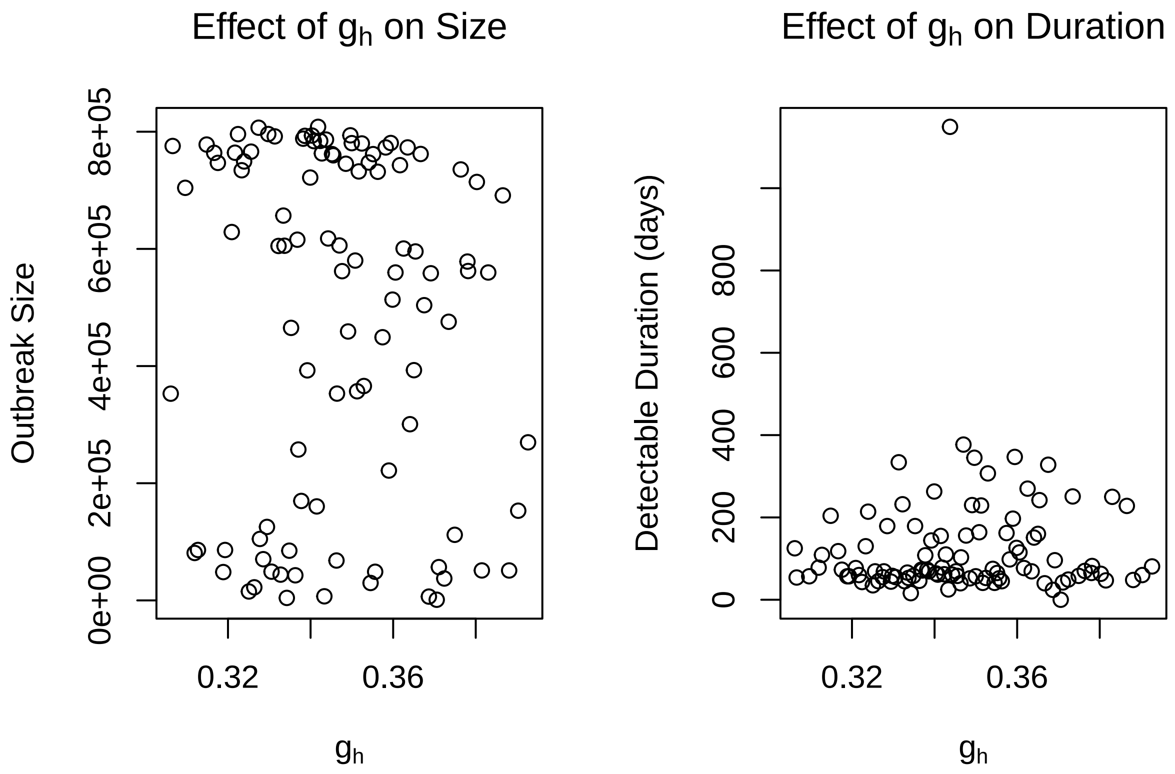


```
plot(bSIRrK$MaxInf ~ bSIRrK$b_h, main = expression(paste("Effect of ", b[h], " on Size")),
    xlab = expression(b[h]), ylab = "Outbreak Size")
plot(bSIRrK$Thresh100 ~ bSIRrK$b_h, main = expression(paste("Effect of ", b[h], " on Duration")),
    xlab = expression(b[h]), ylab = "Detectable Duration (days)")
```

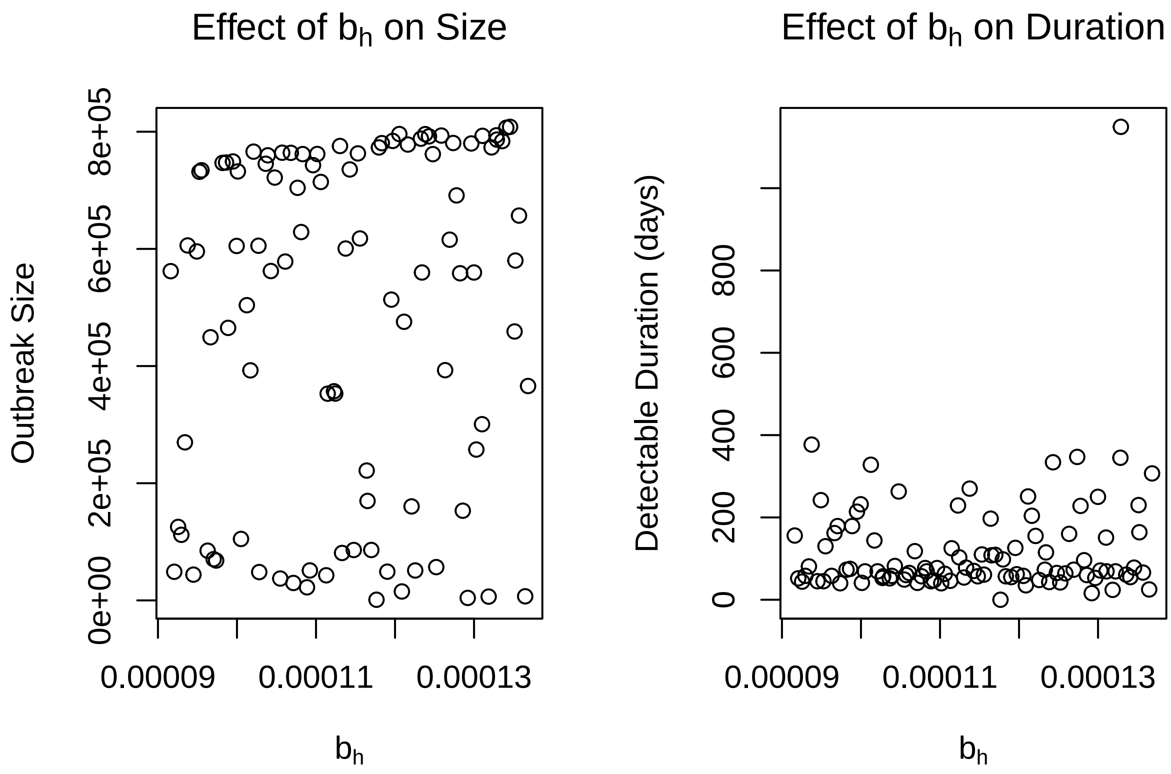


```
plot(bSIRrK$MaxInf ~ bSIRrK$d_h, main = expression(paste("Effect of ", d[h], " on Size")),
    xlab = expression(d[h]), ylab = "Outbreak Size")
plot(bSIRrK$Thresh100 ~ bSIRrK$d_h, main = expression(paste("Effect of ", d[h], " on Duration")),
    xlab = expression(d[h]), ylab = "Detectable Duration (days)")
```

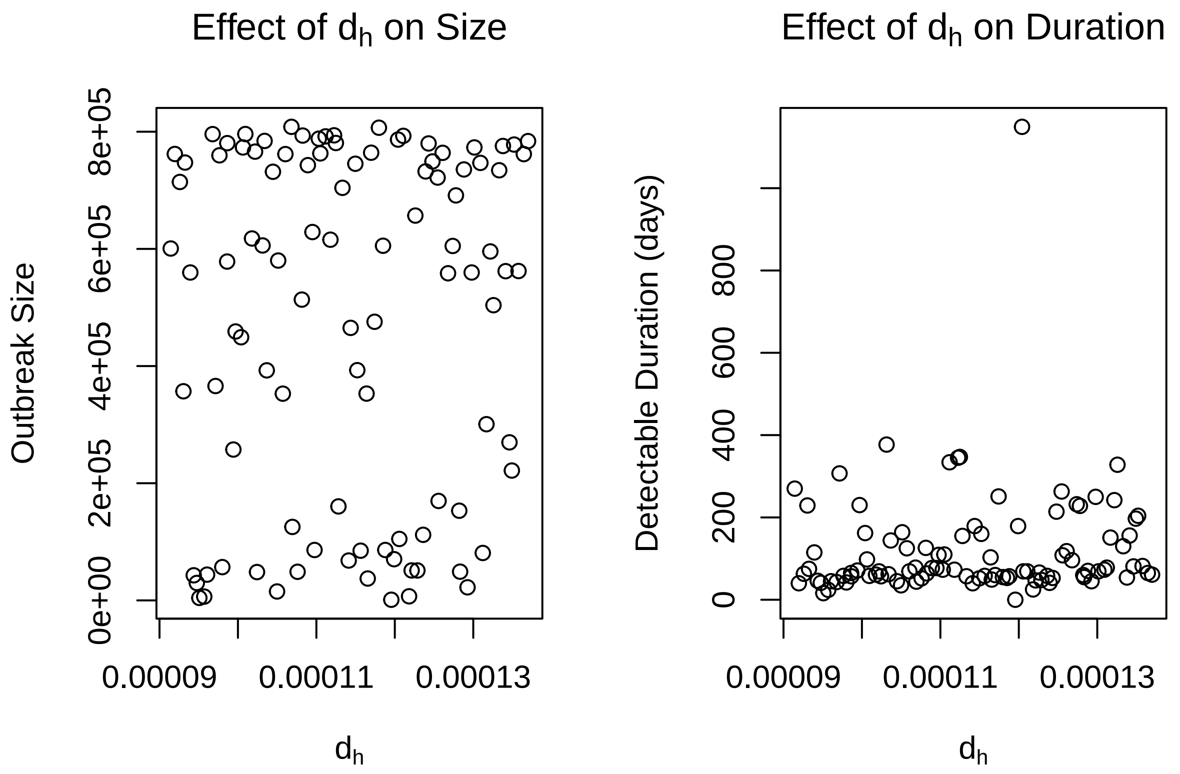


```
par(mfrow = c(1, 2))
boxplot(bSIRrK$MaxInf, main = "Outbreak Size", ylab = "Number of Dead Humans", ylim = c(0,
    923406))
boxplot(bSIRrK$Thresh100, main = "Outbreak Duration", ylab = "Time (Days)")
```

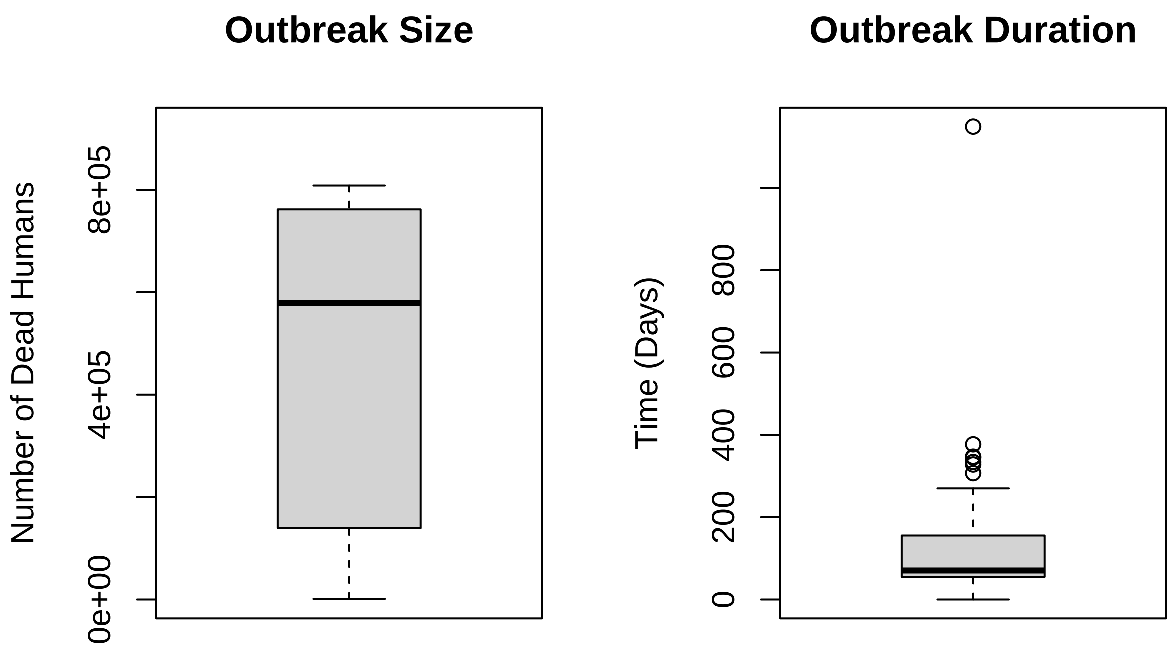


```
bonferroni.alpha <- 0.05/length(parameters)
prcc_size <- pcc(bSIRrK[, 1:length(parameters)], bSIRrK$MaxInf, nboot = niter, rank = TRUE,
    conf = 1 - bonferroni.alpha)
prcc_duration <- pcc(bSIRrK[, 1:length(parameters)], bSIRrK$Thresh100, nboot = niter,
    rank = TRUE, conf = 1 - bonferroni.alpha)

# plot correlation coefficients and confidence intervals for epidemic size and
# duration
size <- prcc_size$PRCC
size$param <- rownames(size)
colnames(size)[4:5] <- c("maxCI", "minCI")
size$maxCI[which(size$maxCI > 1)] <- 1
size$maxCI[which(size$maxCI < -1)] <- -1
size$minCI[which(size$minCI > 1)] <- 1
size$minCI[which(size$minCI < -1)] <- -1

duration <- prcc_duration$PRCC
duration$param <- rownames(duration)
colnames(duration)[4:5] <- c("maxCI", "minCI")
duration$maxCI[which(duration$maxCI > 1)] <- 1
duration$maxCI[which(duration$maxCI < -1)] <- -1
duration$minCI[which(duration$minCI > 1)] <- 1
duration$minCI[which(duration$minCI < -1)] <- -1


A <- ggplot(size, aes(x = param, y = original)) + geom_point(size = 4) + geom_errorbar(aes(ymax = maxCI
```

```
    ymin = minCI)) + ggtitle("A") + xlab("Parameters") + ylab("Partial Rank Correlation Coefficients") +
    scale_x_discrete(labels = c(r_r = expression(r[r]), d_r = expression(d[r]), K_r = expression(K[r]),
        p_r = expression(p[r]), alpha = expression(alpha), beta_h = expression(beta[b]),
        beta_r = expression(beta[r]), b_h = expression(b[h]), d_h = expression(d[h]),
        d_f = expression(d[f]), gamma_h = expression(gamma[b]), gamma_r = expression(gamma[r]),
        g_h = expression(g[h]), g_r = expression(g[r]), K_f = expression(K[f]), r_f = expression(r[f])))
    ylim(-1, 1)

B <- ggplot(duration, aes(x = param, y = original)) + geom_point(size = 4) + geom_errorbar(aes(ymax = ma
    ymin = minCI)) + ggtitle("B") + xlab("Parameters") + ylab(" ") + scale_x_discrete(labels = c(r_r = e
    d_r = expression(d[r]), K_r = expression(K[r]), p_r = expression(p[r]), alpha = expression(alpha),
    beta_h = expression(beta[b]), beta_r = expression(beta[r]), b_h = expression(b[h]),
    d_h = expression(d[h]), d_f = expression(d[f]), gamma_h = expression(gamma[b]),
    gamma_r = expression(gamma[r]), g_h = expression(g[h]), g_r = expression(g[r]),
    K_f = expression(K[f]), r_f = expression(r[f]))) + ylim(-1, 1)
```

Bubonic SEIR with rat carrying capacity and resistance - Figure S5 (Panels C & D)

```
parameters <- c(r_r = 0.014, K_r = 923405, p_r = 0.975, d_r = 0.00055, beta_r = 0.09,
    alpha = 3/923406, gamma_r = 1/5.15, g_r = 0.1, r_f = 0.0084, K_f = 6, d_f = 1/5,
    beta_h = 0.19, sigma_h = 1/4, gamma_h = 1/10, g_h = 0.34, b_h = 1/(25 * 365),
    d_h = 1/(25 * 365))  #you can play with transmission and recovery rates here

par(mfrow = c(1, 2))
plot(bSEIRrK$MaxInf ~ bSEIRrK$r_r, main = expression(paste("Effect of ", r[r], " on Size")),
    xlab = expression(r[r]), ylab = "Outbreak Size")
plot(bSEIRrK$Thresh100 ~ bSEIRrK$r_r, main = expression(paste("Effect of ", r[r],
    " on Duration")), xlab = expression(r[r]), ylab = "Detectable Duration (days)")
```

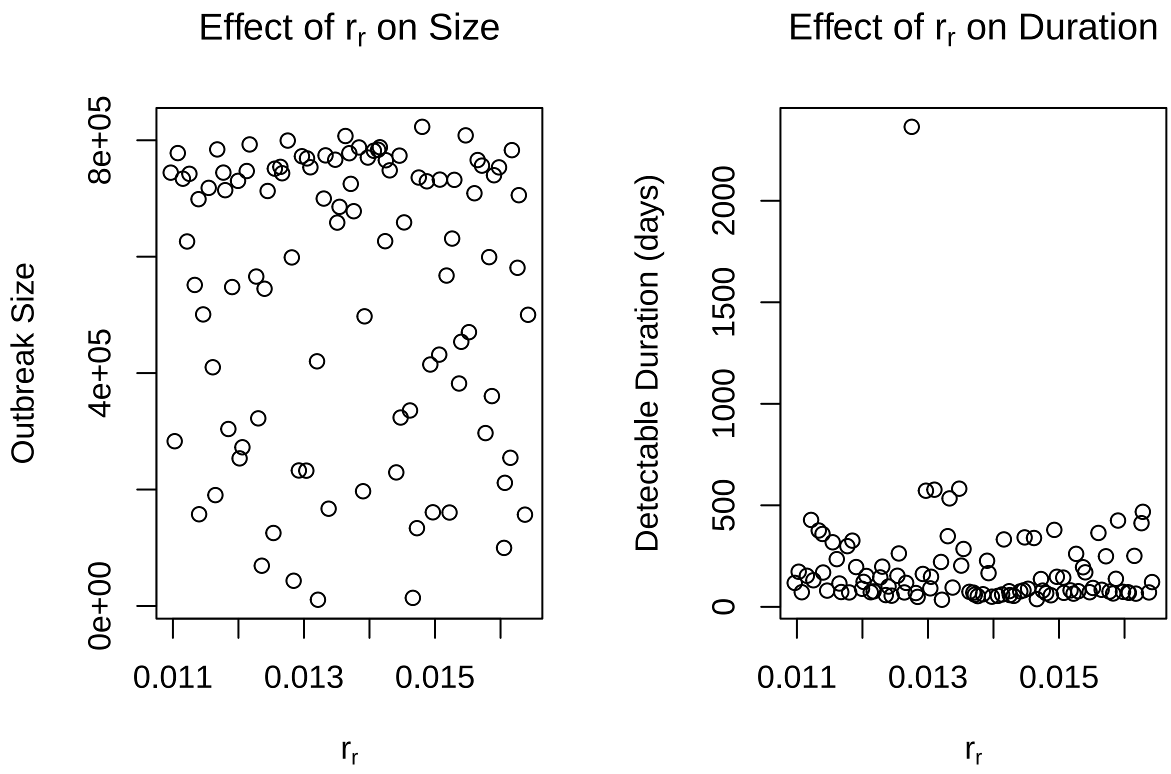


```
plot(bSEIRrK$MaxInf ~ bSEIRrK$K_r, main = expression(paste("Effect of ", K[r], " on Size")),
    xlab = expression(K[r]), ylab = "Outbreak Size")
plot(bSEIRrK$Thresh100 ~ bSEIRrK$K_r, main = expression(paste("Effect of ", K[r],
    " on Duration")), xlab = expression(K[r]), ylab = "Detectable Duration (days)")
```

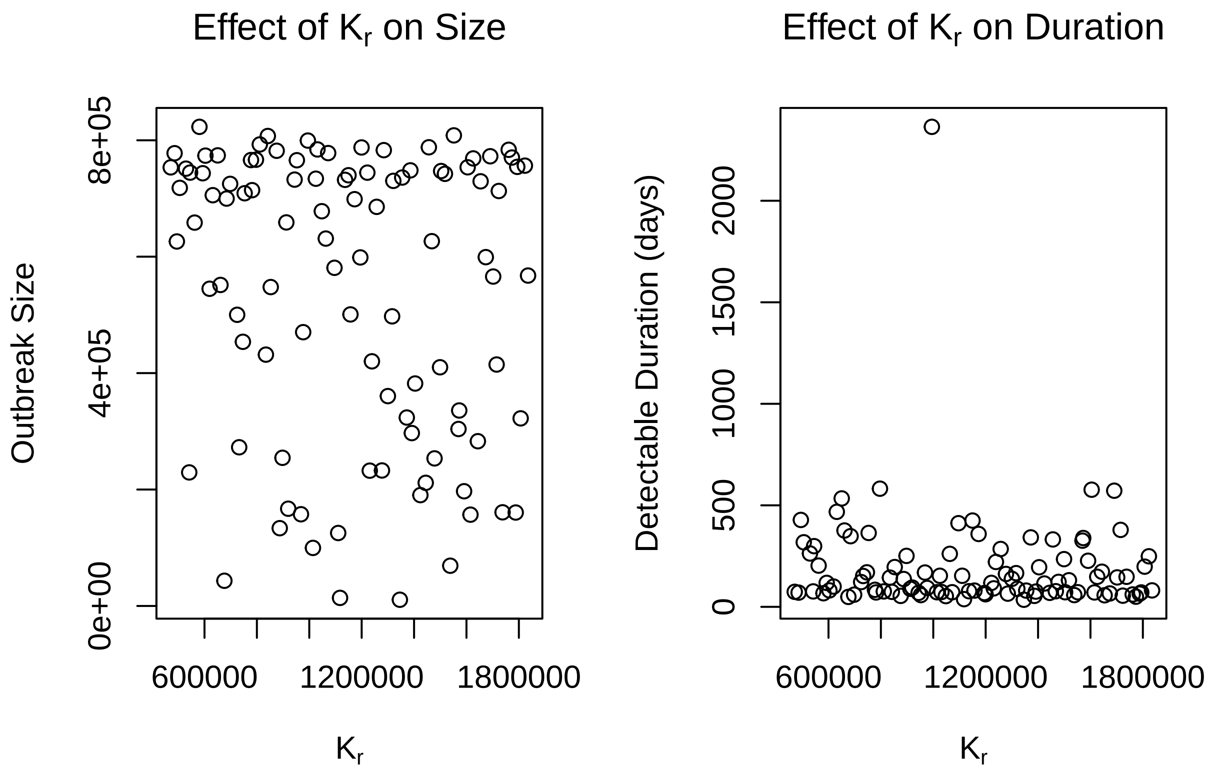


```r
plot(bSEIRrK$MaxInf ~ bSEIRrK$p_r, main = expression(paste("Effect of ", p[r], " on Size")),
    xlab = expression(p[r]), ylab = "Outbreak Size")
plot(bSEIRrK$Thresh100 ~ bSEIRrK$p_r, main = expression(paste("Effect of ", p[r],
    " on Duration")), xlab = expression(p[r]), ylab = "Detectable Duration (days)")
```

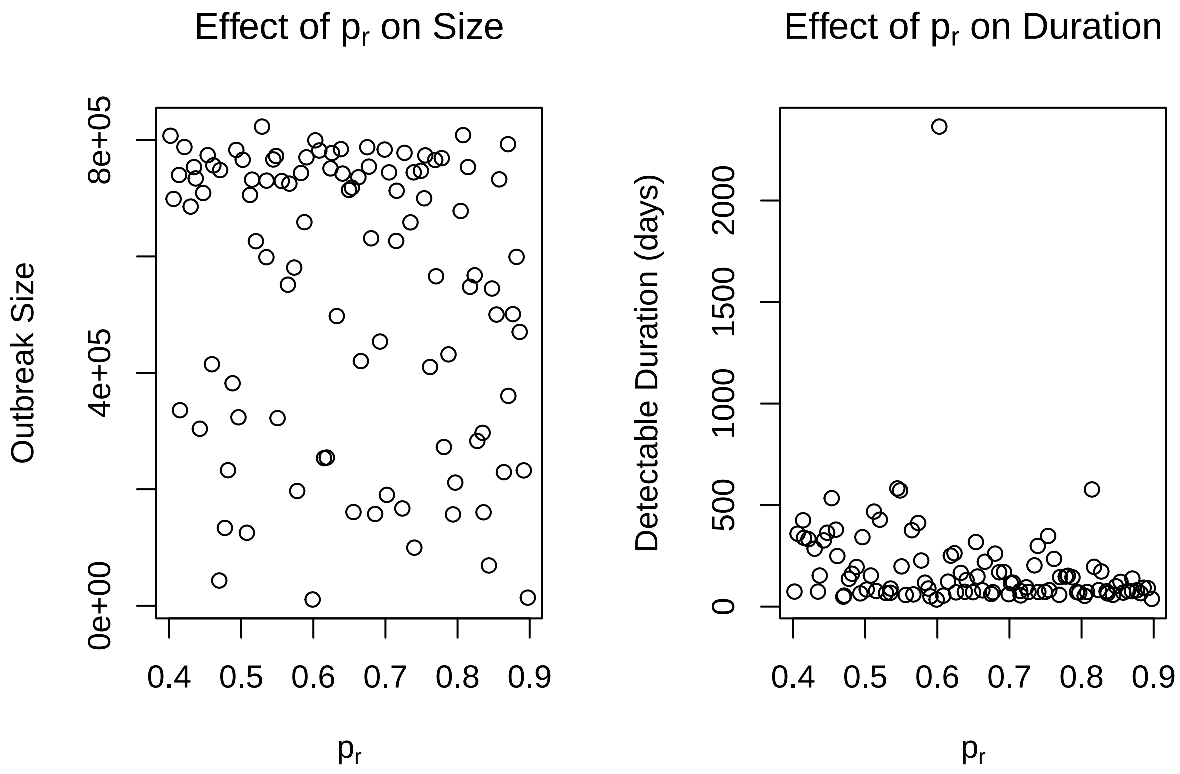


```
plot(bSEIRrK$MaxInf ~ bSEIRrK$d_r, main = expression(paste("Effect of ", d[r], " on Size")),
    xlab = expression(d[r]), ylab = "Outbreak Size")
plot(bSEIRrK$Thresh100 ~ bSEIRrK$d_r, main = expression(paste("Effect of ", d[r],
    " on Duration")), xlab = expression(d[r]), ylab = "Detectable Duration (days)")
```

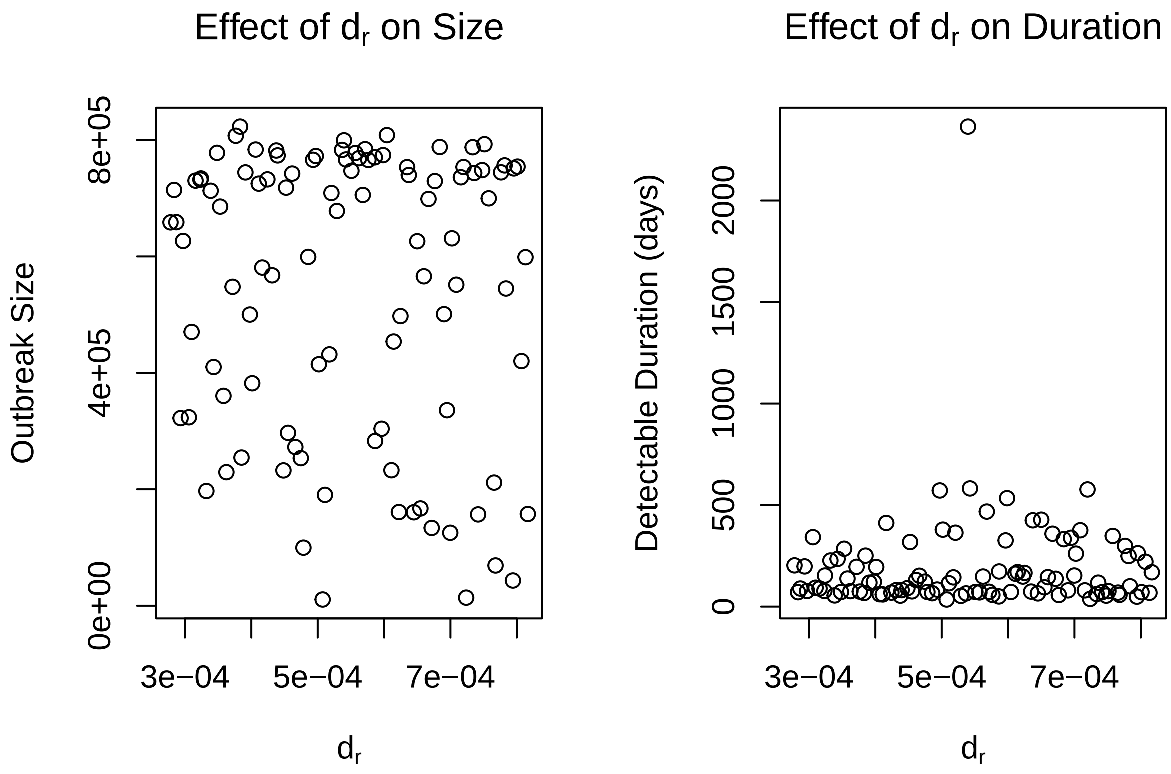


```
plot(bSEIRrK$MaxInf ~ bSEIRrK$beta_r, main = expression(paste("Effect of ", beta[r],
    " on Size")), xlab = expression(beta[r]), ylab = "Outbreak Size")
plot(bSEIRrK$Thresh100 ~ bSEIRrK$beta_r, main = expression(paste("Effect of ", beta[r],
    " on Duration")), xlab = expression(beta[r]), ylab = "Detectable Duration (days)")
```

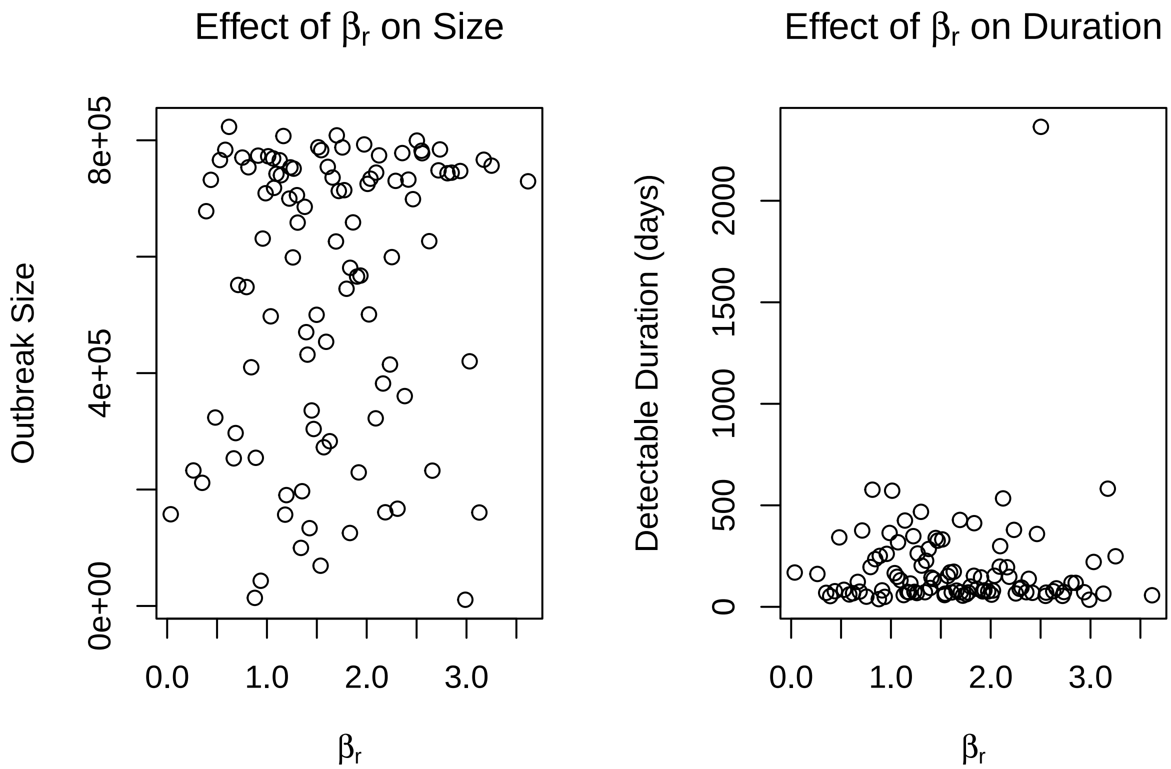


```
plot(bSEIRrK$MaxInf ~ bSEIRrK$alpha, main = expression(paste("Effect of ", alpha,
    " on Size")), xlab = expression(alpha), ylab = "Outbreak Size")
plot(bSEIRrK$Thresh100 ~ bSEIRrK$alpha, main = expression(paste("Effect of ", alpha,
    " on Duration")), xlab = expression(alpha), ylab = "Detectable Duration (days)")
```

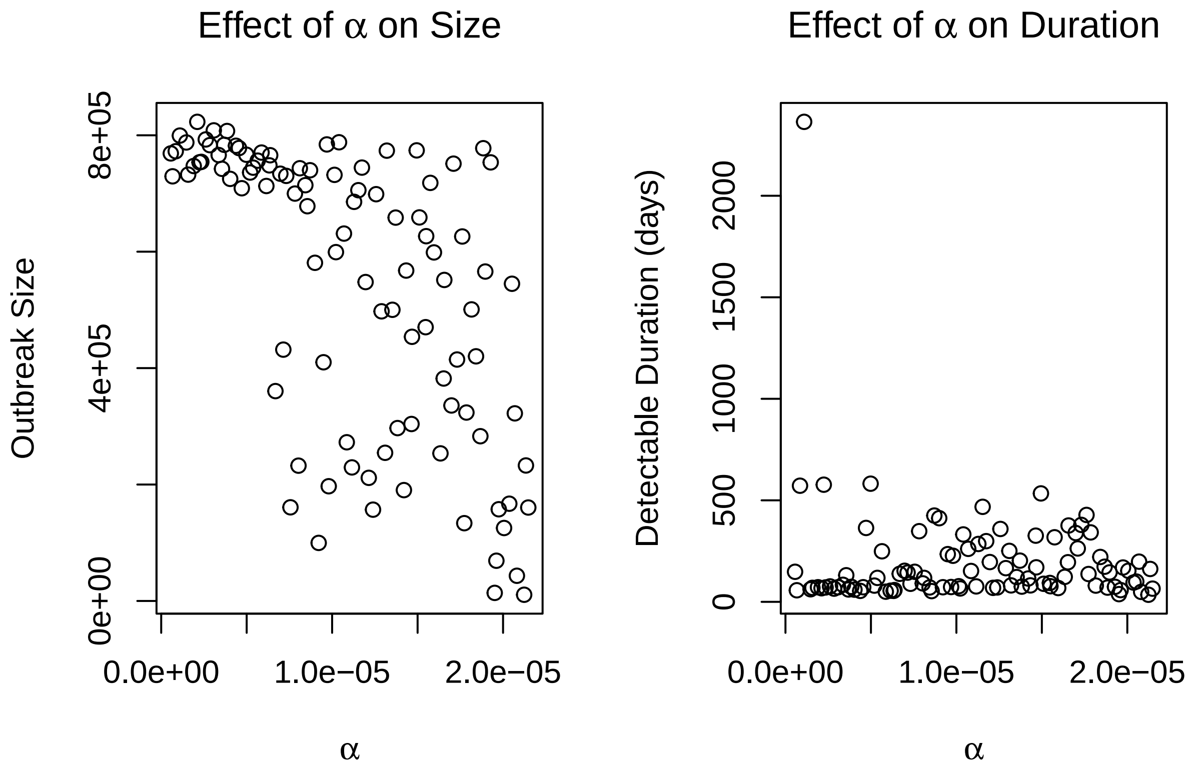


```r
plot(bSEIRrK$MaxInf ~ bSEIRrK$gamma_r, main = expression(paste("Effect of ", gamma[r],
    " on Size")), xlab = expression(gamma[r]), ylab = "Outbreak Size")
plot(bSEIRrK$Thresh100 ~ bSEIRrK$gamma_r, main = expression(paste("Effect of ", gamma[r],
    " on Duration")), xlab = expression(gamma[r]), ylab = "Detectable Duration (days)")
```

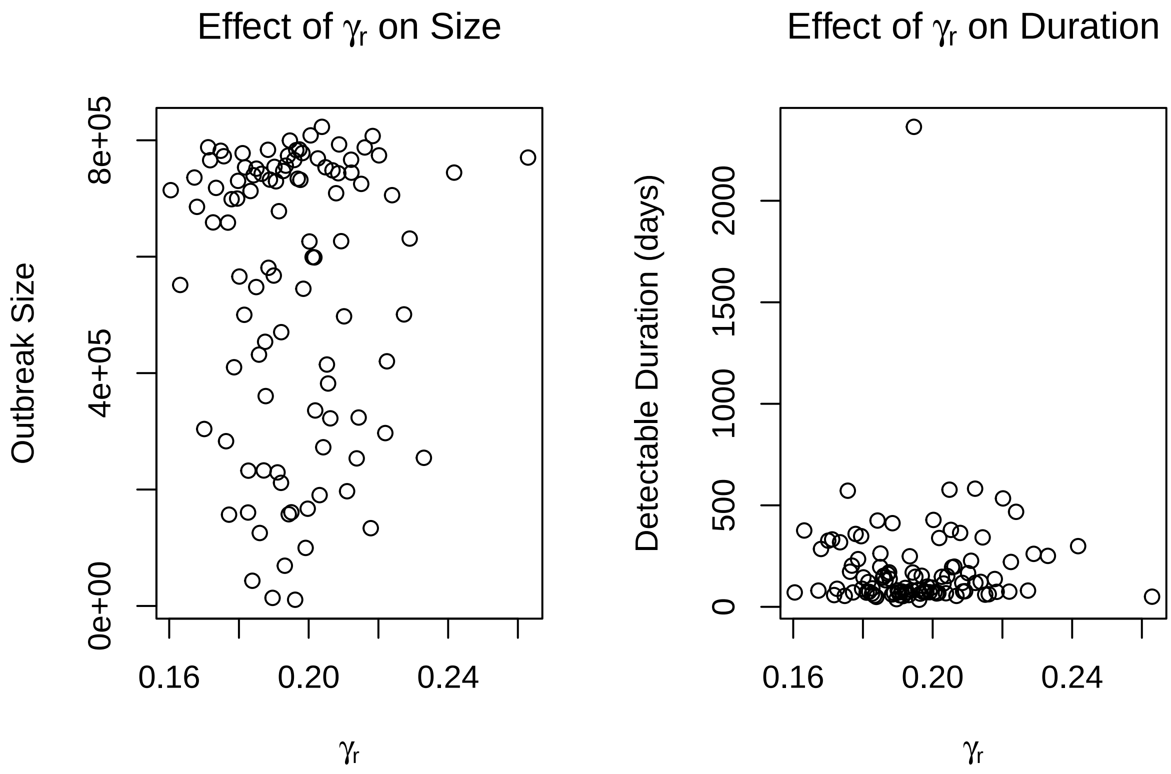


```
plot(bSEIRrK$MaxInf ~ bSEIRrK$g_r, main = expression(paste("Effect of ", g[r], " on Size")),
    xlab = expression(g[r]), ylab = "Outbreak Size")
plot(bSEIRrK$Thresh100 ~ bSEIRrK$g_r, main = expression(paste("Effect of ", g[r],
    " on Duration")), xlab = expression(g[r]), ylab = "Detectable Duration (days)")
```

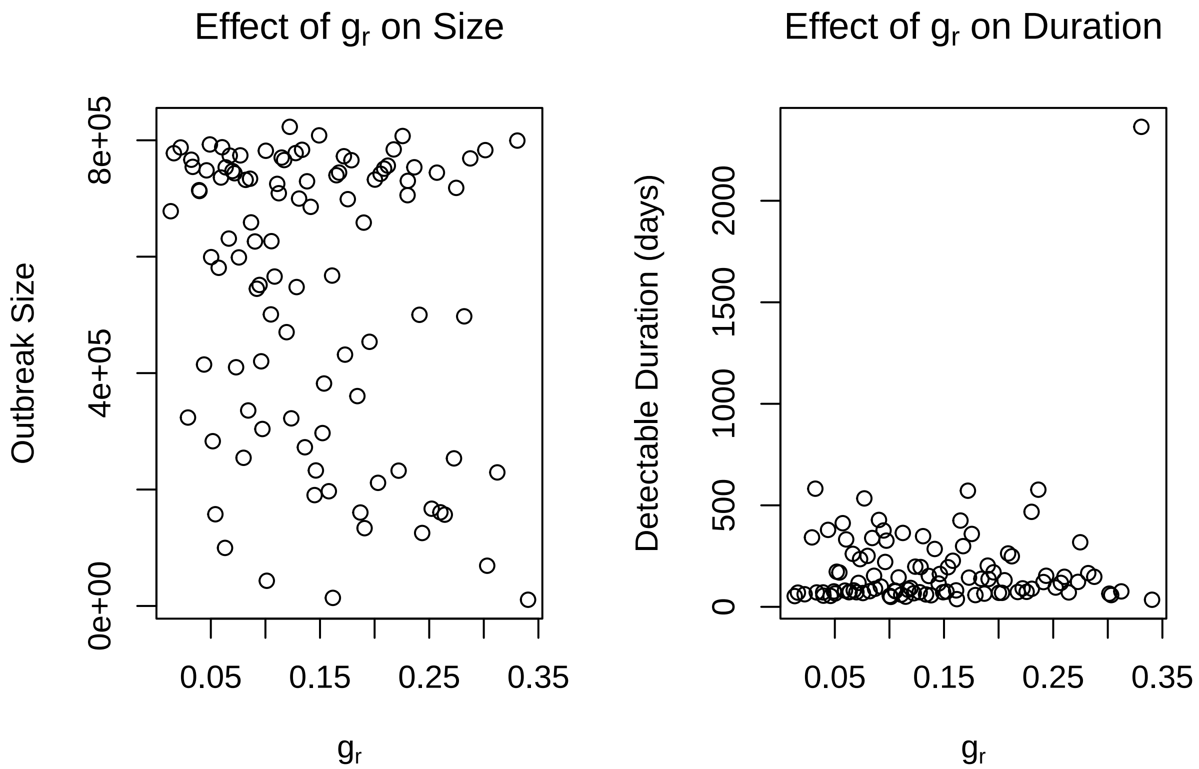


```r
plot(bSEIRrK$MaxInf ~ bSEIRrK$r_f, main = expression(paste("Effect of ", r[f], " on Size")),
    xlab = expression(r[f]), ylab = "Outbreak Size")
plot(bSEIRrK$Thresh100 ~ bSEIRrK$r_f, main = expression(paste("Effect of ", r[f],
    " on Duration")), xlab = expression(r[f]), ylab = "Detectable Duration (days)")
```

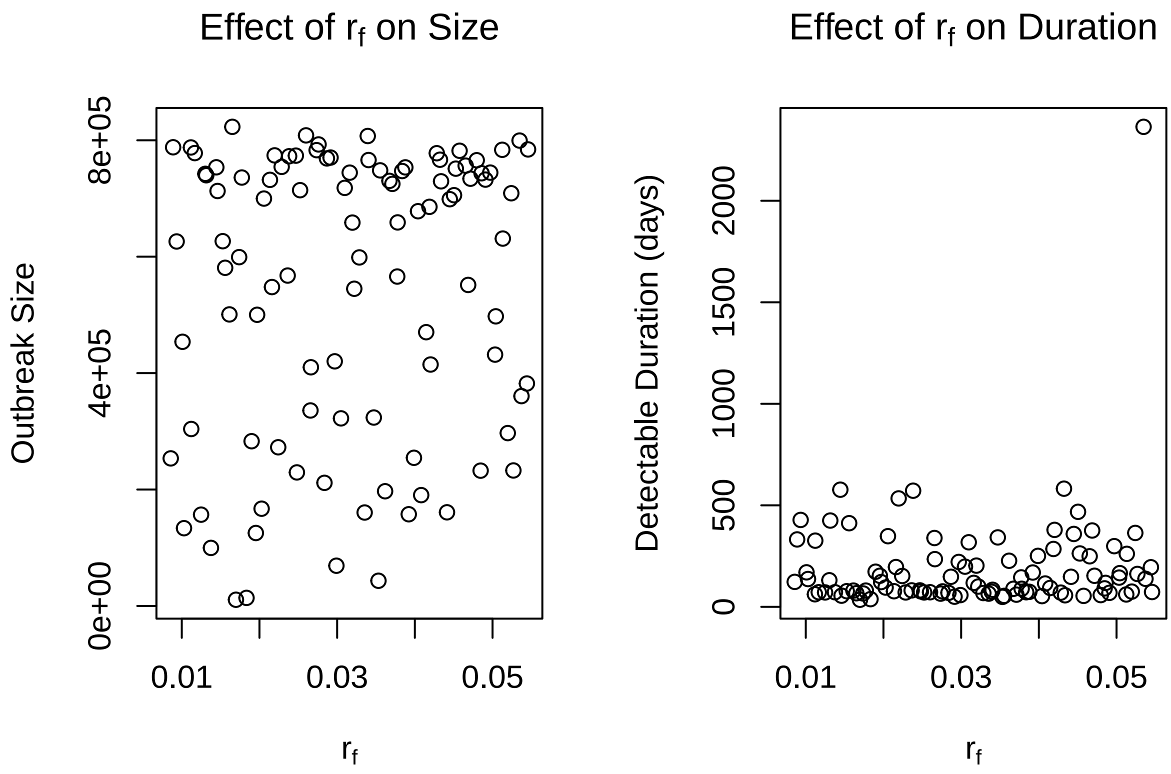


```r
plot(bSEIRrK$MaxInf ~ bSEIRrK$K_f, main = expression(paste("Effect of ", K[f], " on Size")),
    xlab = expression(K[f]), ylab = "Outbreak Size")
plot(bSEIRrK$Thresh100 ~ bSEIRrK$K_f, main = expression(paste("Effect of ", K[f],
    " on Duration")), xlab = expression(K[f]), ylab = "Detectable Duration (days)")
```

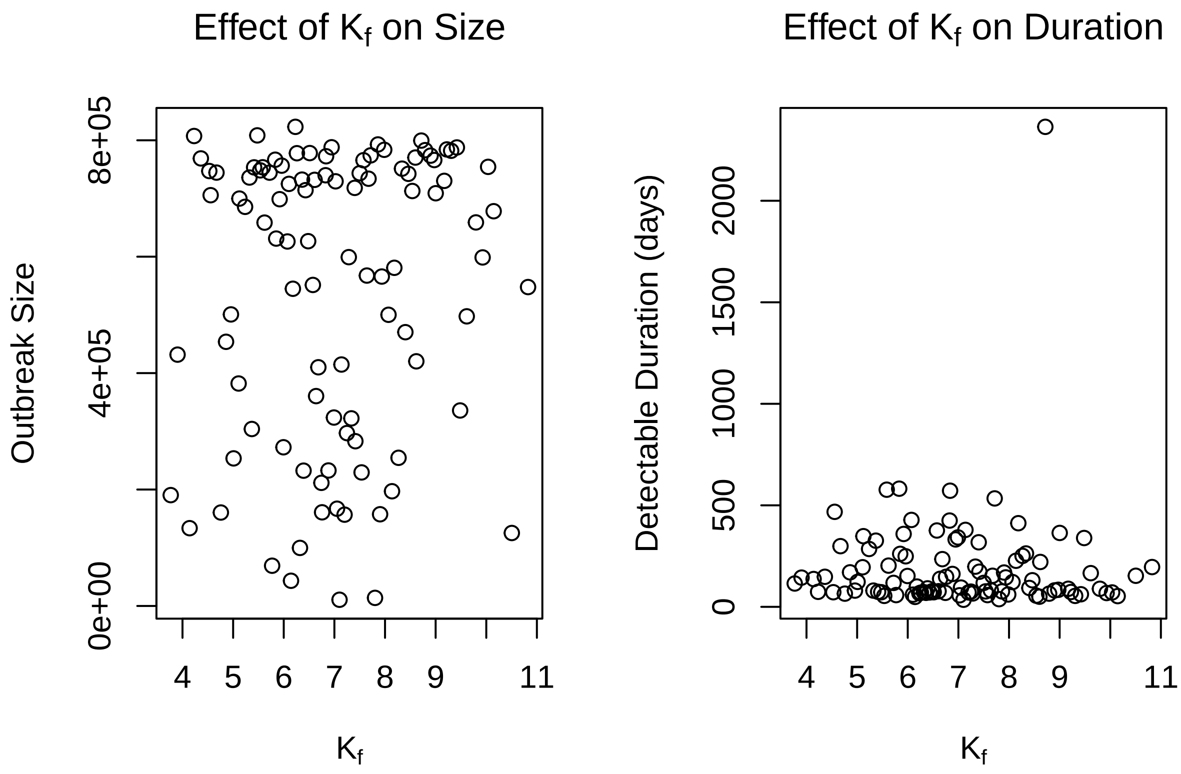


```
plot(bSEIRrK$MaxInf ~ bSEIRrK$d_f, main = expression(paste("Effect of ", d[f], " on Size")),
    xlab = expression(d[f]), ylab = "Outbreak Size")
plot(bSEIRrK$Thresh100 ~ bSEIRrK$d_f, main = expression(paste("Effect of ", d[f],
    " on Duration")), xlab = expression(d[f]), ylab = "Detectable Duration (days)")
```

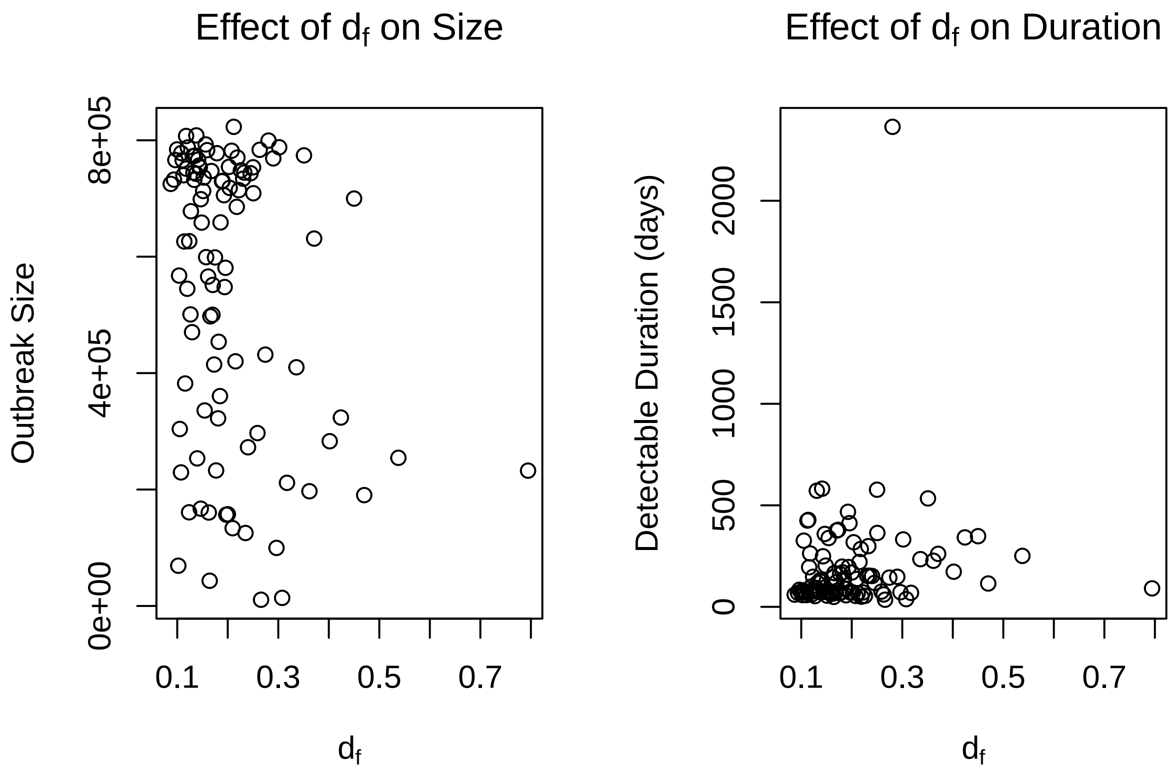


```
plot(bSEIRrK$MaxInf ~ bSEIRrK$beta_h, main = expression(paste("Effect of ", beta[b],
    " on Size")), xlab = expression(beta[b]), ylab = "Outbreak Size")
plot(bSEIRrK$Thresh100 ~ bSEIRrK$beta_h, main = expression(paste("Effect of ", beta[b],
    " on Duration")), xlab = expression(beta[b]), ylab = "Detectable Duration (days)")
```

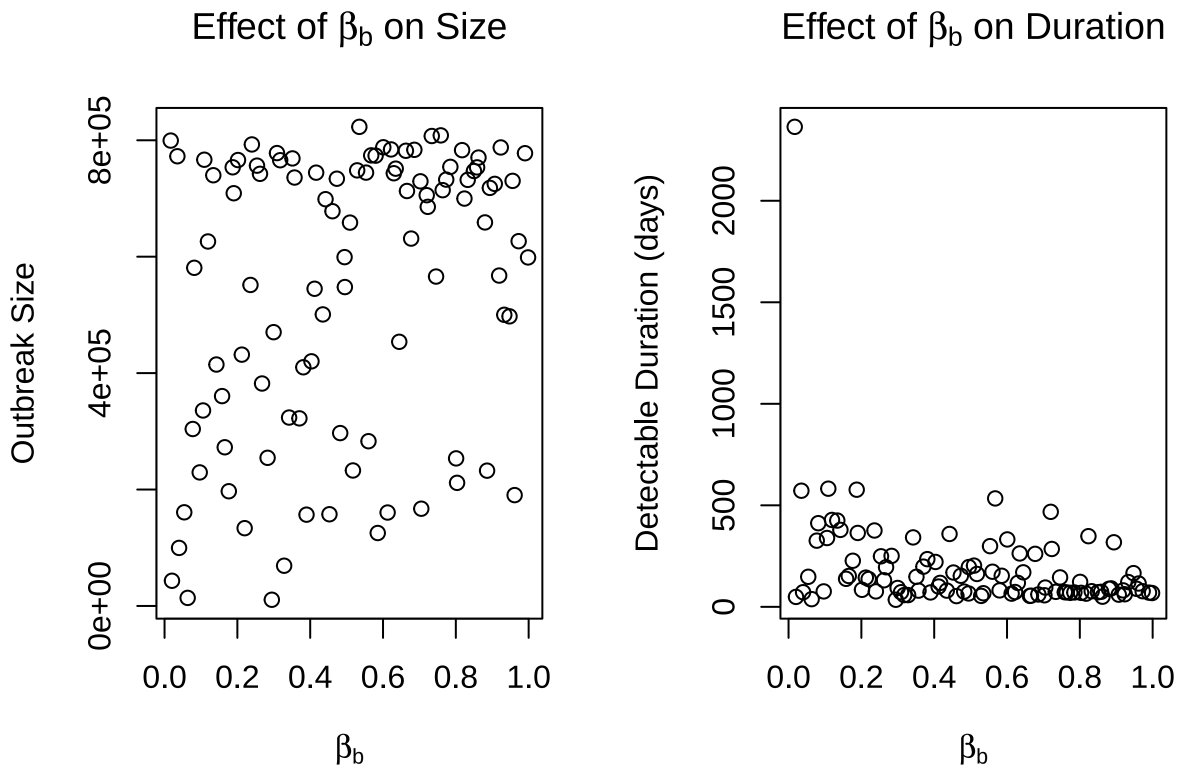


```
plot(bSEIRrK$MaxInf ~ bSEIRrK$sigma_h, main = expression(paste("Effect of ", sigma[b],
    " on Size")), xlab = expression(sigma[b]), ylab = "Outbreak Size")
plot(bSEIRrK$Thresh100 ~ bSEIRrK$sigma_h, main = expression(paste("Effect of ", sigma[b],
    " on Duration")), xlab = expression(sigma[b]), ylab = "Detectable Duration (days)")
```

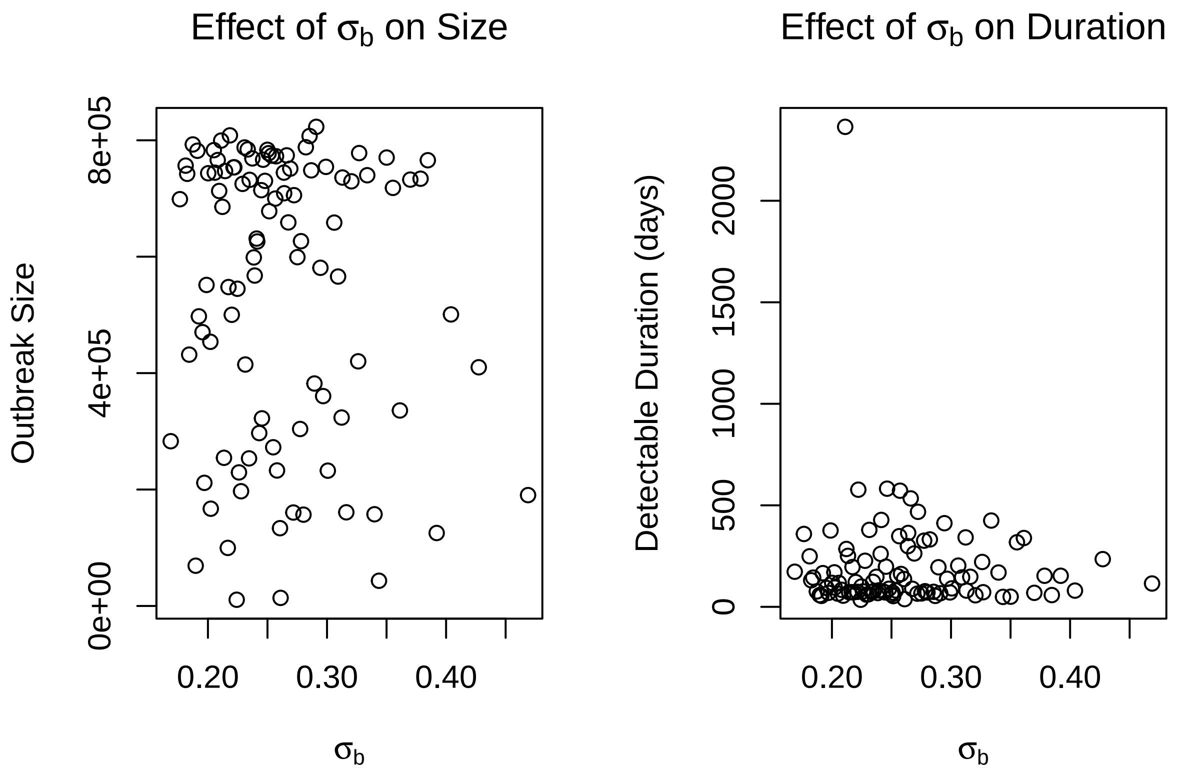


```
plot(bSEIRrK$MaxInf ~ bSEIRrK$gamma_h, main = expression(paste("Effect of ", gamma[b],
    " on Size")), xlab = expression(gamma[b]), ylab = "Outbreak Size")
plot(bSEIRrK$Thresh100 ~ bSEIRrK$gamma_h, main = expression(paste("Effect of ", gamma[b],
    " on Duration")), xlab = expression(gamma[b]), ylab = "Detectable Duration (days)")
```

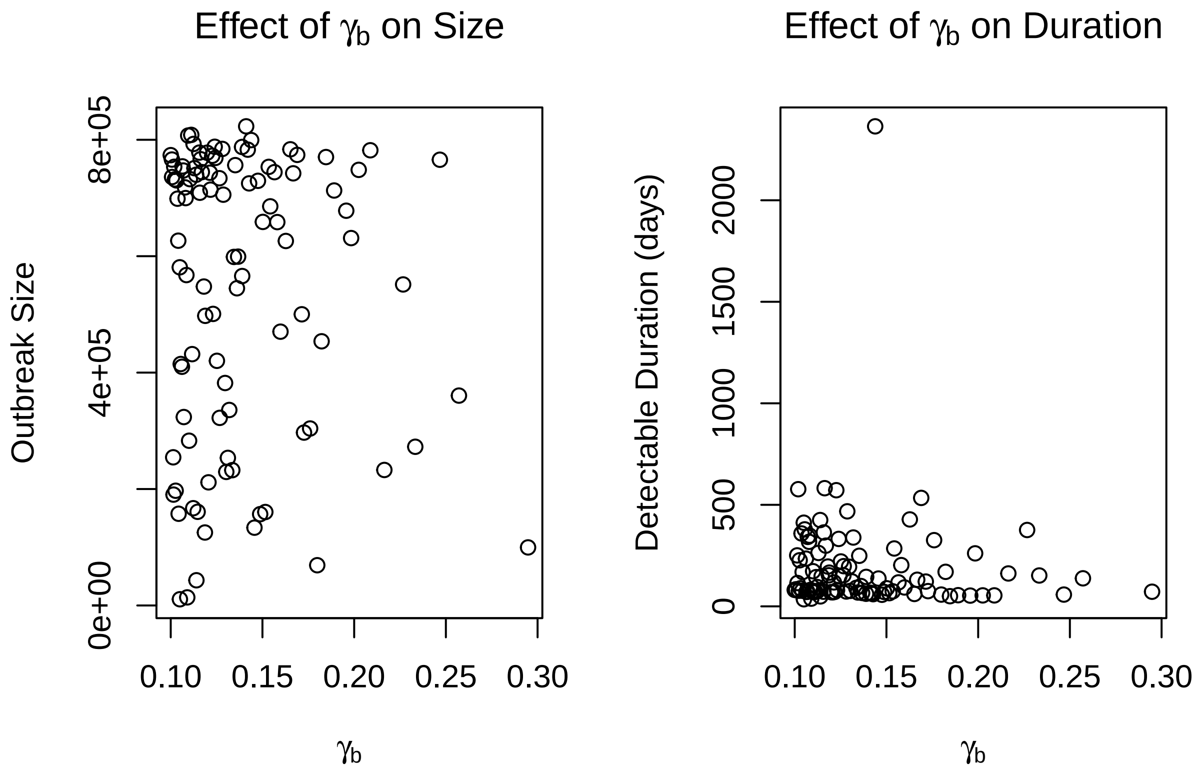


```r
plot(bSEIRrK$MaxInf ~ bSEIRrK$g_h, main = expression(paste("Effect of ", g[h], " on Size")),
    xlab = expression(g[h]), ylab = "Outbreak Size")
plot(bSEIRrK$Thresh100 ~ bSEIRrK$g_h, main = expression(paste("Effect of ", g[h],
    " on Duration")), xlab = expression(g[h]), ylab = "Detectable Duration (days)")
```

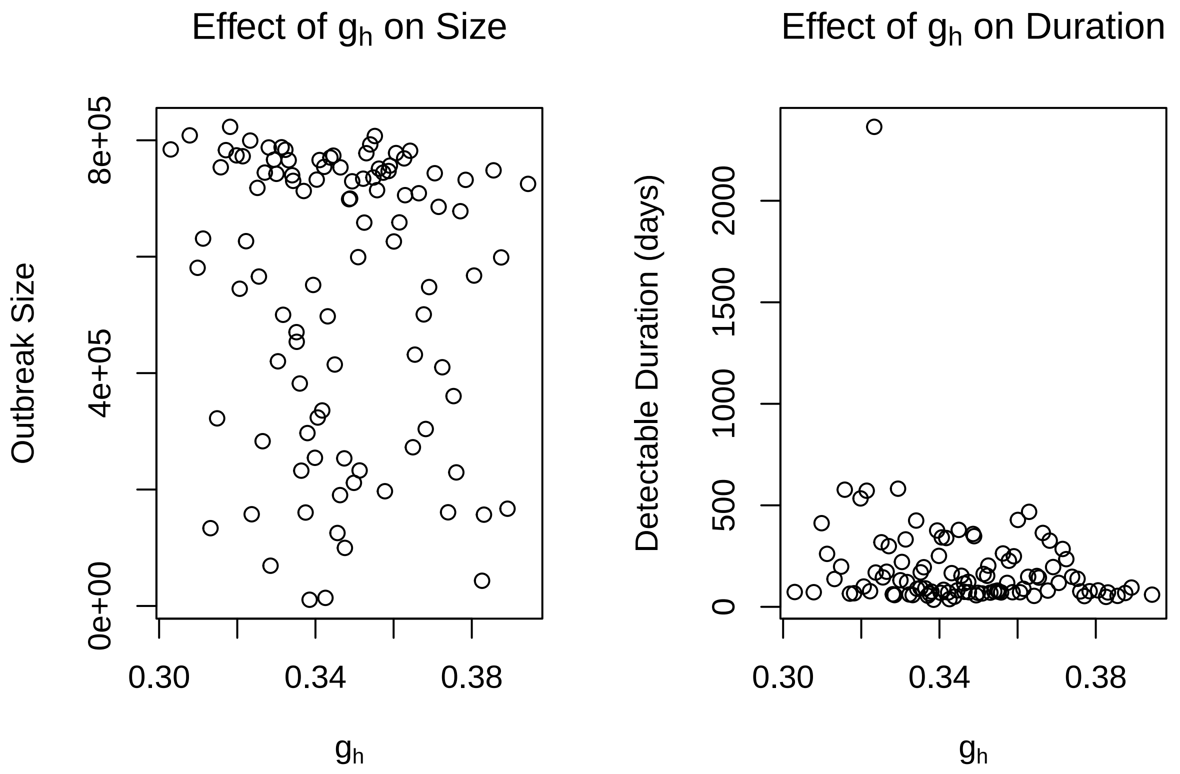


```
plot(bSEIRrK$MaxInf ~ bSEIRrK$b_h, main = expression(paste("Effect of ", b[h], " on Size")),
    xlab = expression(b[h]), ylab = "Outbreak Size")
plot(bSEIRrK$Thresh100 ~ bSEIRrK$b_h, main = expression(paste("Effect of ", b[h],
    " on Duration")), xlab = expression(b[h]), ylab = "Detectable Duration (days)")
```

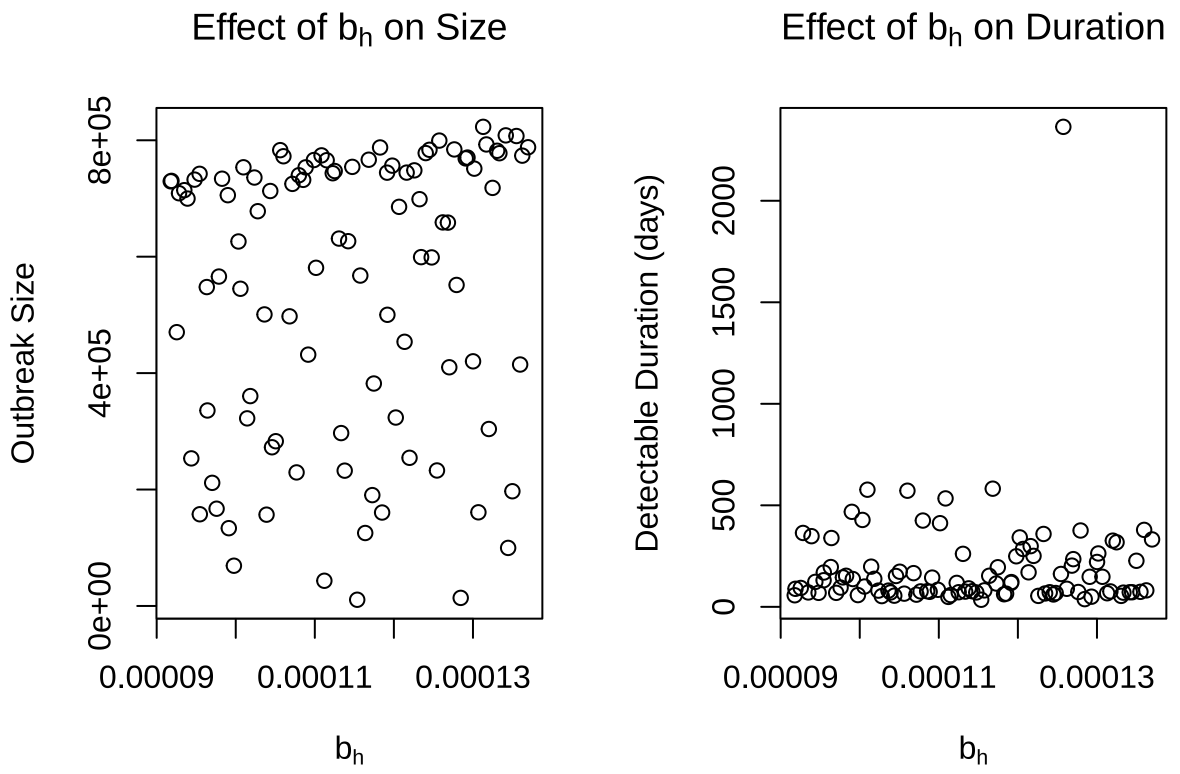


```r
plot(bSEIRrK$MaxInf ~ bSEIRrK$d_h, main = expression(paste("Effect of ", d[h], " on Size")),
    xlab = expression(d[h]), ylab = "Outbreak Size")
plot(bSEIRrK$Thresh100 ~ bSEIRrK$d_h, main = expression(paste("Effect of ", d[h],
    " on Duration")), xlab = expression(d[h]), ylab = "Detectable Duration (days)")
```

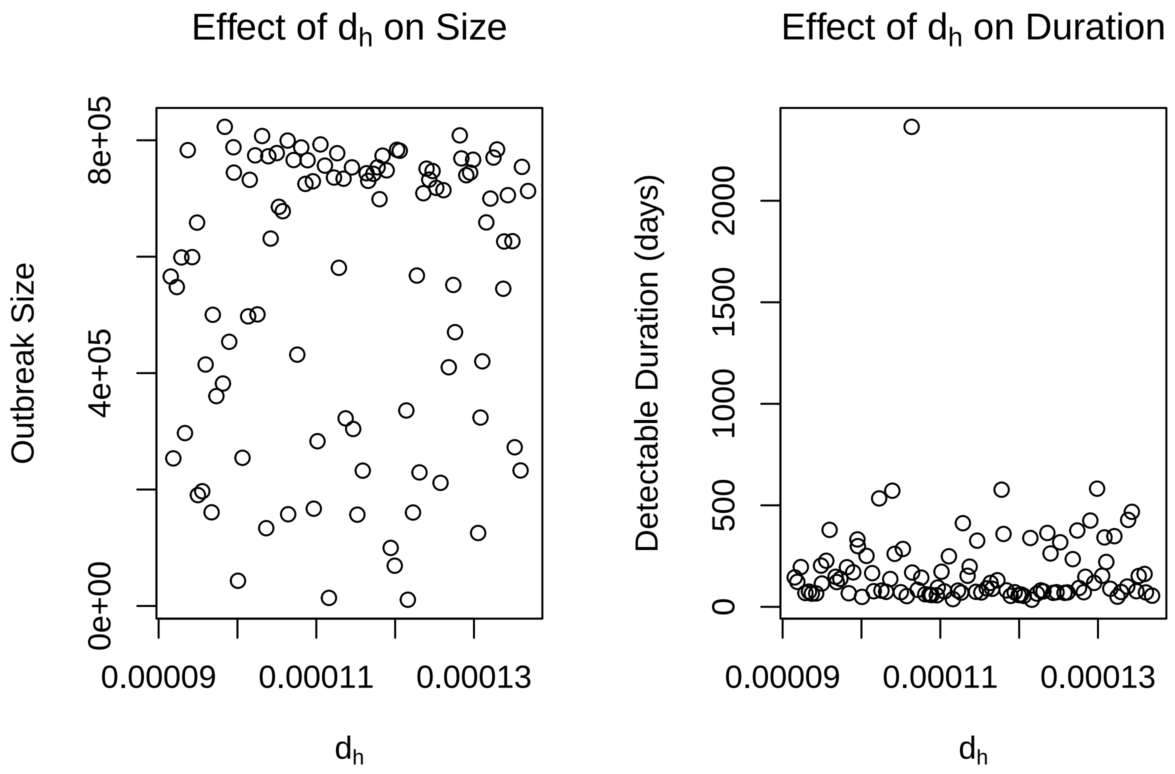


```
par(mfrow = c(1, 2))
boxplot(bSEIRrK$MaxInf, main = "Outbreak Size", ylab = "Number of Dead Humans", ylim = c(0,
    923406))
boxplot(bSEIRrK$Thresh100, main = "Outbreak Duration", ylab = "Time (Days)")
```

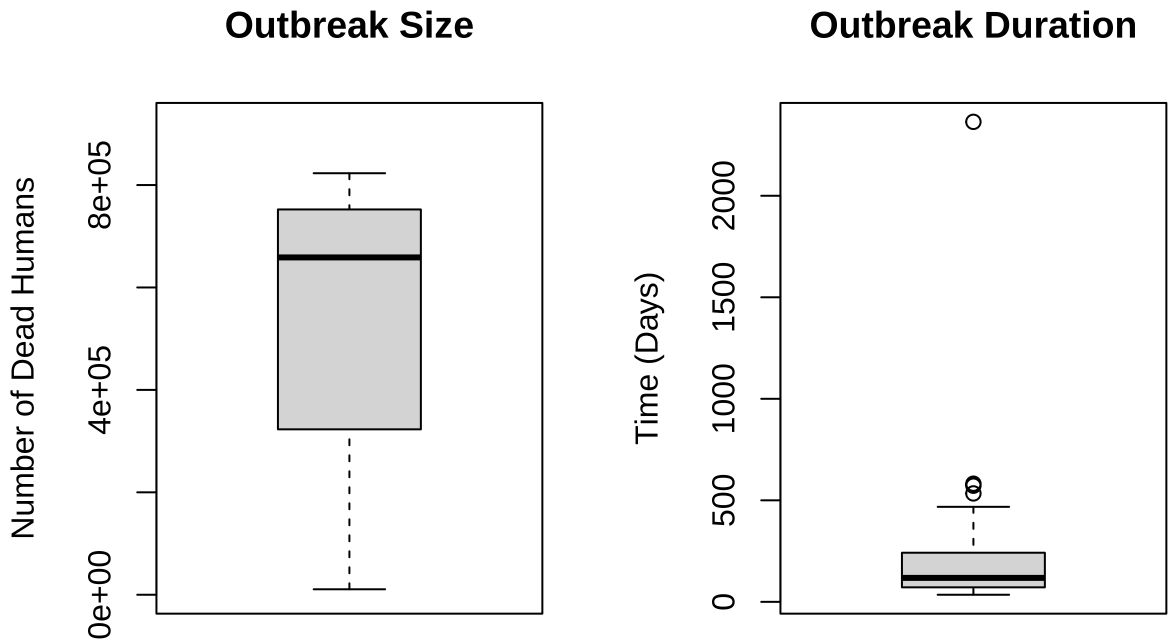


```r
bonferroni.alpha <- 0.05/length(parameters)
prcc_size <- pcc(bSEIRrK[, 1:length(parameters)], bSEIRrK$MaxInf, nboot = niter,
    rank = TRUE, conf = 1 - bonferroni.alpha)
prcc_duration <- pcc(bSEIRrK[, 1:length(parameters)], bSEIRrK$Thresh100, nboot = niter,
    rank = TRUE, conf = 1 - bonferroni.alpha)

# plot correlation coefficients and confidence intervals for epidemic size and
# duration
size <- prcc_size$PRCC
size$param <- rownames(size)
colnames(size)[4:5] <- c("maxCI", "minCI")
size$maxCI[which(size$maxCI > 1)] <- 1
size$maxCI[which(size$maxCI < -1)] <- -1
size$minCI[which(size$minCI > 1)] <- 1
size$minCI[which(size$minCI < -1)] <- -1

duration <- prcc_duration$PRCC
duration$param <- rownames(duration)
colnames(duration)[4:5] <- c("maxCI", "minCI")
duration$maxCI[which(duration$maxCI > 1)] <- 1
duration$maxCI[which(duration$maxCI < -1)] <- -1
duration$minCI[which(duration$minCI > 1)] <- 1
duration$minCI[which(duration$minCI < -1)] <- -1


C <- ggplot(size, aes(x = param, y = original)) + geom_point(size = 4) + geom_errorbar(aes(ymax = maxCI
```

```
    ymin = minCI)) + ggtitle("C") + xlab("Parameters") + ylab("Partial Rank Correlation Coefficients") [illegible]
    scale_x_discrete(labels = c(r_r = expression(r[r]), d_r = expression(d[r]), K_r = expression(K[r]),
        p_r = expression(p[r]), alpha = expression(alpha), beta_h = expression(beta[b]),
        beta_r = expression(beta[r]), sigma_h = expression(sigma[b]), b_h = expression(b[h]),
        d_h = expression(d[h]), d_f = expression(d[f]), gamma_h = expression(gamma[b]),
        gamma_r = expression(gamma[r]), g_h = expression(g[h]), g_r = expression(g[r]),
        K_f = expression(K[f]), r_f = expression(r[f]))) + ylim(-1, 1)

D <- ggplot(duration, aes(x = param, y = original)) + geom_point(size = 4) + geom_errorbar(aes(ymax = m[illegible]
    ymin = minCI)) + ggtitle("D") + xlab("Parameters") + ylab(" ") + scale_x_discrete(labels = c(r_r = [illegible]
    d_r = expression(d[r]), K_r = expression(K[r]), p_r = expression(p[r]), alpha = expression(alpha),
    beta_h = expression(beta[b]), beta_r = expression(beta[r]), sigma_h = expression(sigma[b]),
    b_h = expression(b[h]), d_h = expression(d[h]), d_f = expression(d[f]), gamma_h = expression(gamma[[illegible]
    gamma_r = expression(gamma[r]), g_h = expression(g[h]), g_r = expression(g[r]),
    K_f = expression(K[f]), r_f = expression(r[f]))) + ylim(-1, 1)

tiff("FigS5.tiff", height = 22.23, width = 19.05, units = "cm", compression = "lzw",
    res = 600)
multiplot(A, B, C, D, cols = 2)
dev.off()
```

```
## pdf
##   2
```

Bubonic/Pneumonic SEIR - Figure S6 (Panels A & B)

```
parameters <- c(beta_r = 0.09, alpha = 3/923406, gamma_r = 1/5.15, g_r = 0.1, r_f = 0.0084,
    K_f = 6, d_f = 1/5, beta_b = 0.19, beta_p = 0.45, sigma_b = 1/6, sigma_p = 1/4.3,
    gamma_b = 1/10, gamma_p = 1/2.5, p = 0.2, g_h = 0.34, b_h = 1/(25 * 365), d_h = 1/(25 *
        365))  #you can play with transmission and recovery rates here

par(mfrow = c(1, 2))
plot(bpSEIR$MaxInf ~ bpSEIR$beta_r, main = expression(paste("Effect of ", beta[r],
    " on Size")), xlab = expression(beta[r]), ylab = "Outbreak Size")
plot(bpSEIR$Thresh100 ~ bpSEIR$beta_r, main = expression(paste("Effect of ", beta[r],
    " on Duration")), xlab = expression(beta[r]), ylab = "Detectable Duration (days)")
```

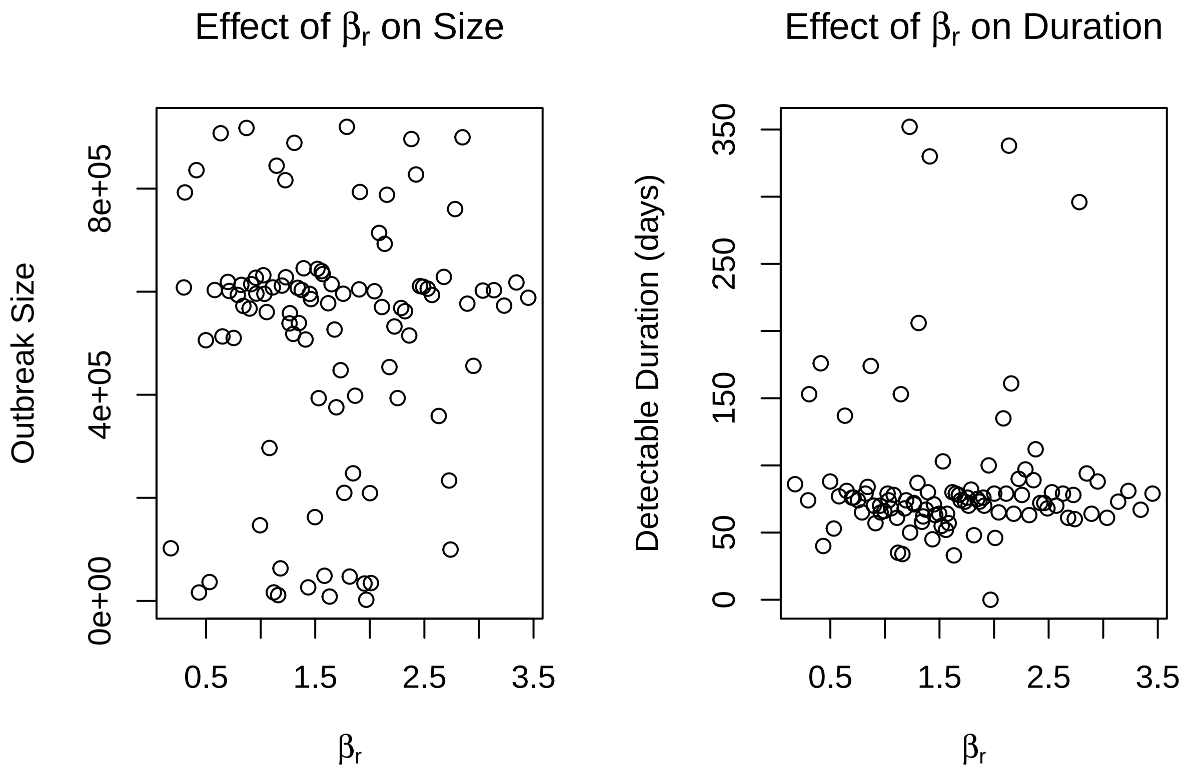


```
plot(bpSEIR$MaxInf ~ bpSEIR$alpha, main = expression(paste("Effect of ", alpha, " on Size")),
    xlab = expression(alpha), ylab = "Outbreak Size")
plot(bpSEIR$Thresh100 ~ bpSEIR$alpha, main = expression(paste("Effect of ", alpha,
    " on Duration")), xlab = expression(alpha), ylab = "Detectable Duration (days)")
```

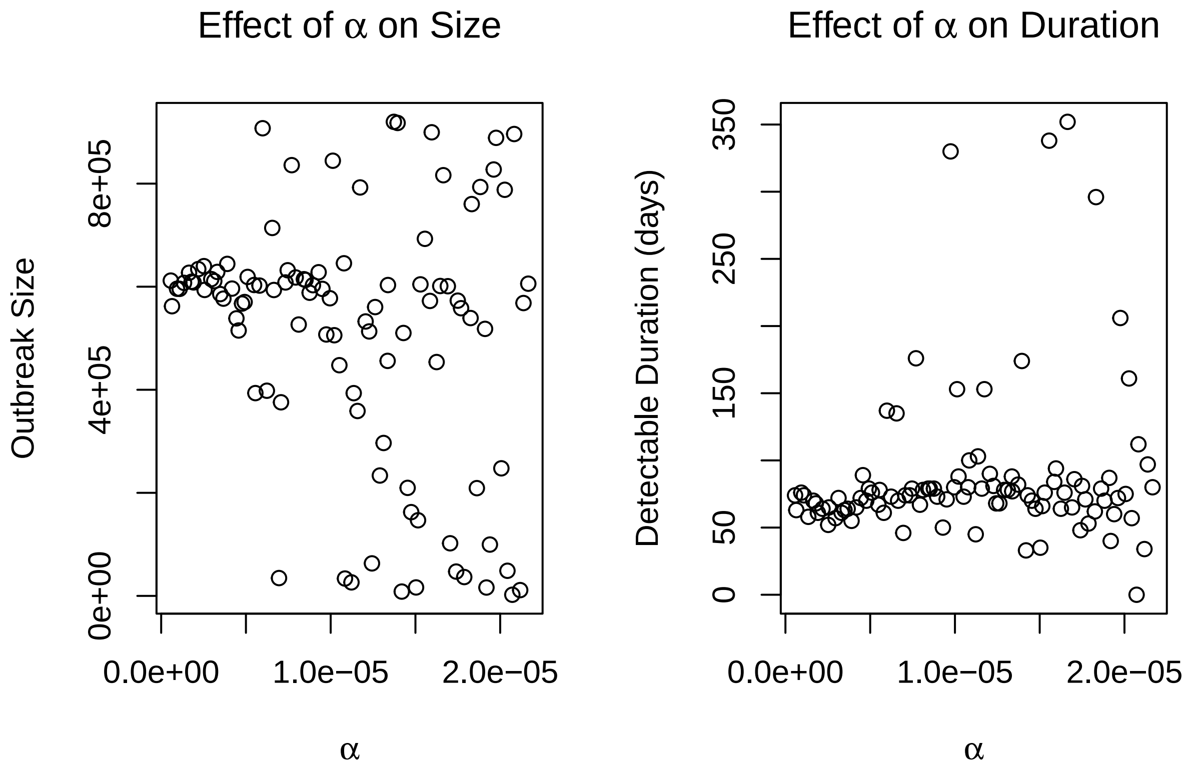


```
plot(bpSEIR$MaxInf ~ bpSEIR$gamma_r, main = expression(paste("Effect of ", gamma[r],
    " on Size")), xlab = expression(gamma[r]), ylab = "Outbreak Size")
plot(bpSEIR$Thresh100 ~ bpSEIR$gamma_r, main = expression(paste("Effect of ", gamma[r],
    " on Duration")), xlab = expression(gamma[r]), ylab = "Detectable Duration (days)")
```

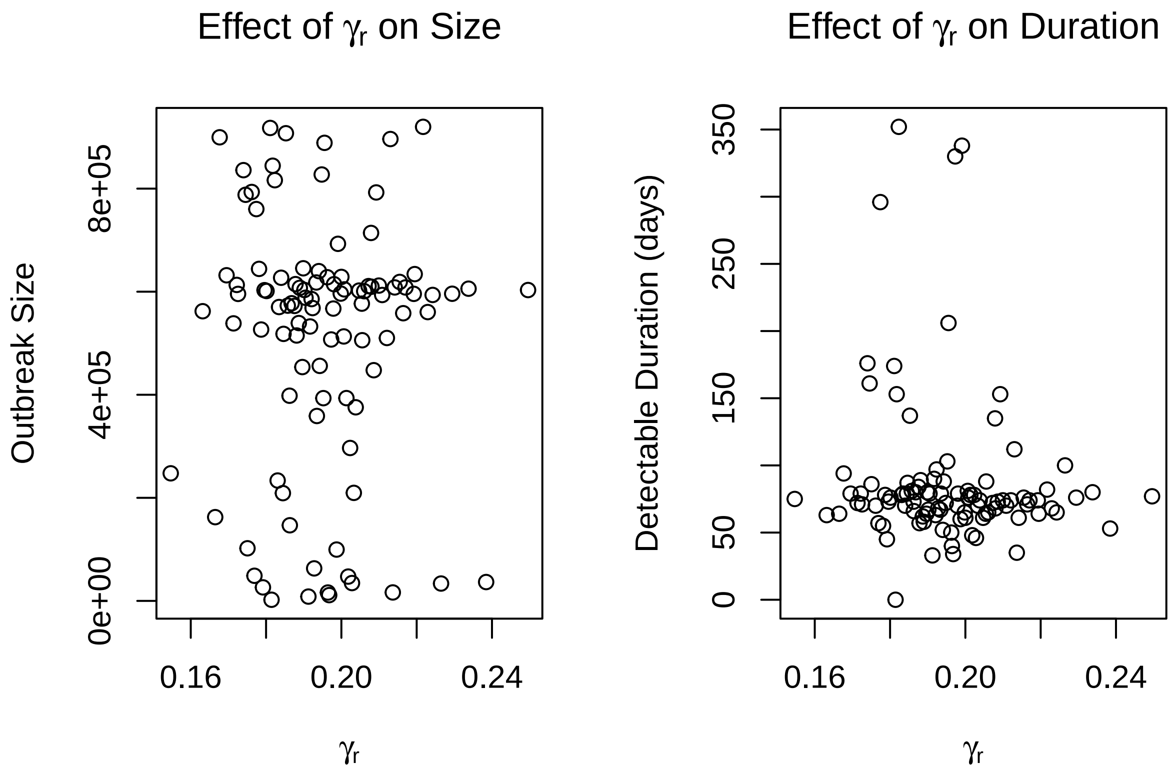


```
plot(bpSEIR$MaxInf ~ bpSEIR$g_r, main = expression(paste("Effect of ", g[r], " on Size")),
    xlab = expression(g[r]), ylab = "Outbreak Size")
plot(bpSEIR$Thresh100 ~ bpSEIR$g_r, main = expression(paste("Effect of ", g[r], " on Duration")),
    xlab = expression(g[r]), ylab = "Detectable Duration (days)")
```

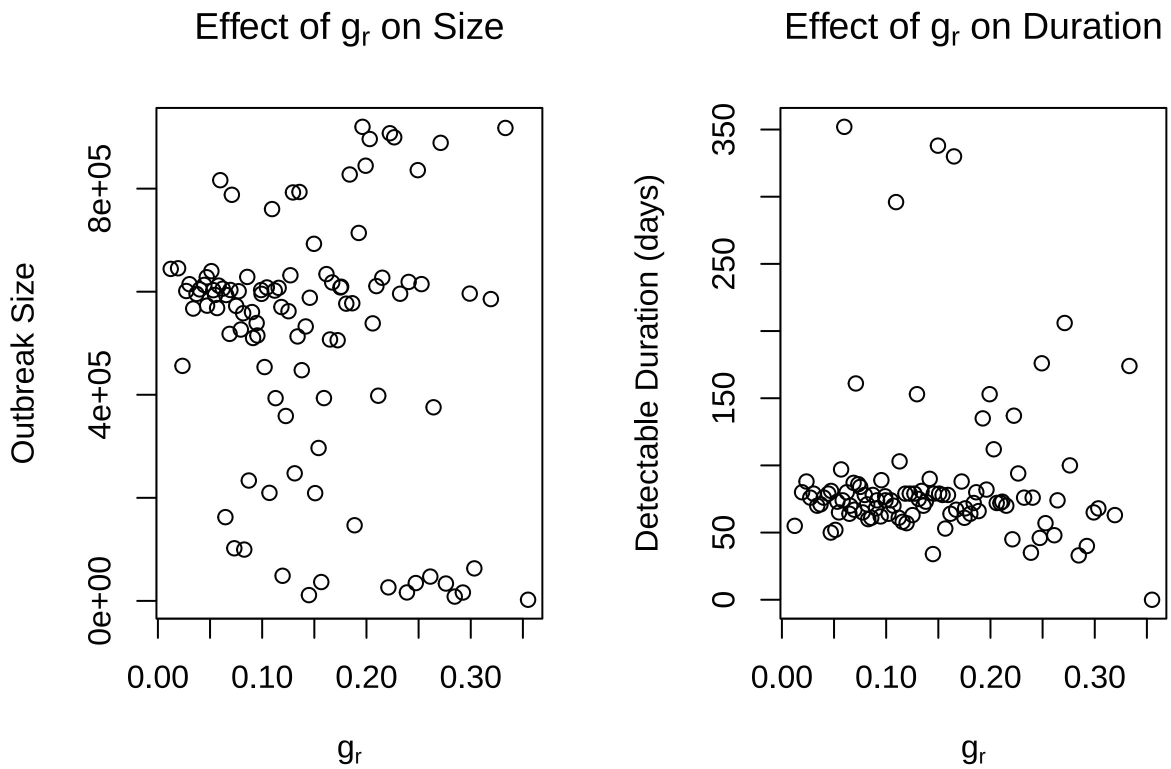


```
plot(bpSEIR$MaxInf ~ bpSEIR$r_f, main = expression(paste("Effect of ", r[f], " on Size")),
    xlab = expression(r[f]), ylab = "Outbreak Size")
plot(bpSEIR$Thresh100 ~ bpSEIR$r_f, main = expression(paste("Effect of ", r[f], " on Duration")),
    xlab = expression(r[f]), ylab = "Detectable Duration (days)")
```

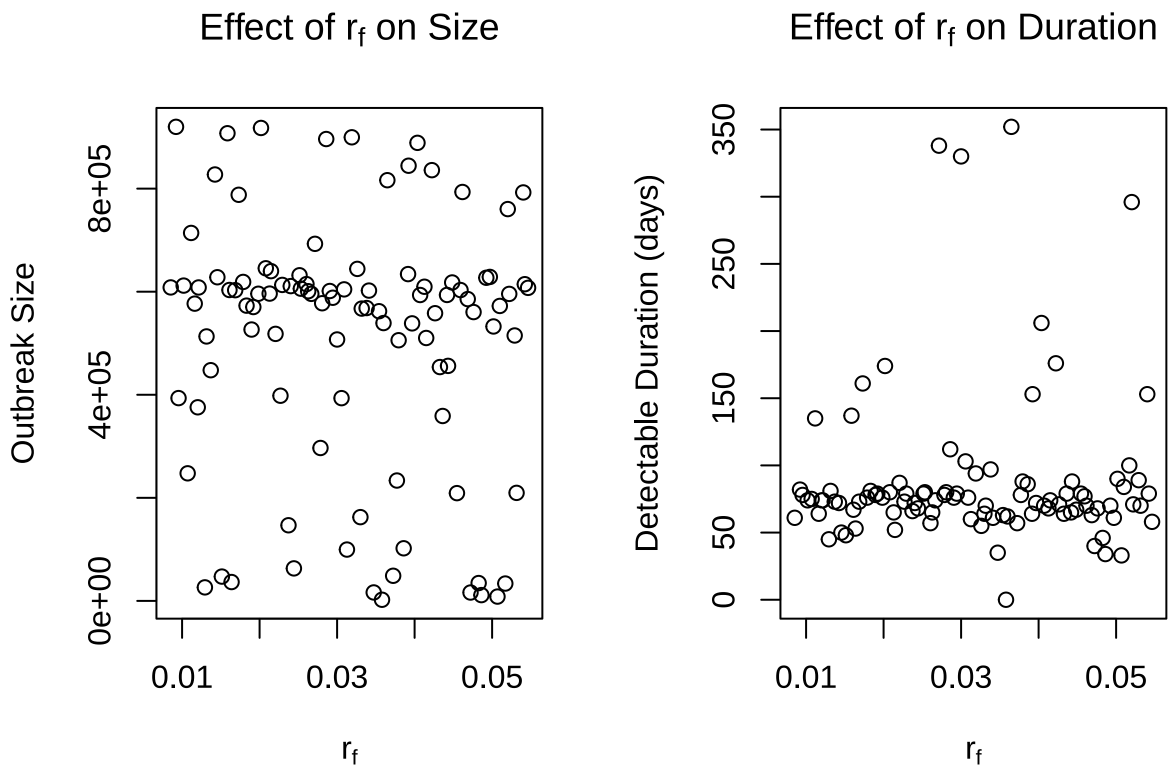


```r
plot(bpSEIR$MaxInf ~ bpSEIR$K_f, main = expression(paste("Effect of ", K[f], " on Size")),
    xlab = expression(K[f]), ylab = "Outbreak Size")
plot(bpSEIR$Thresh100 ~ bpSEIR$K_f, main = expression(paste("Effect of ", K[f], " on Duration")),
    xlab = expression(K[f]), ylab = "Detectable Duration (days)")
```

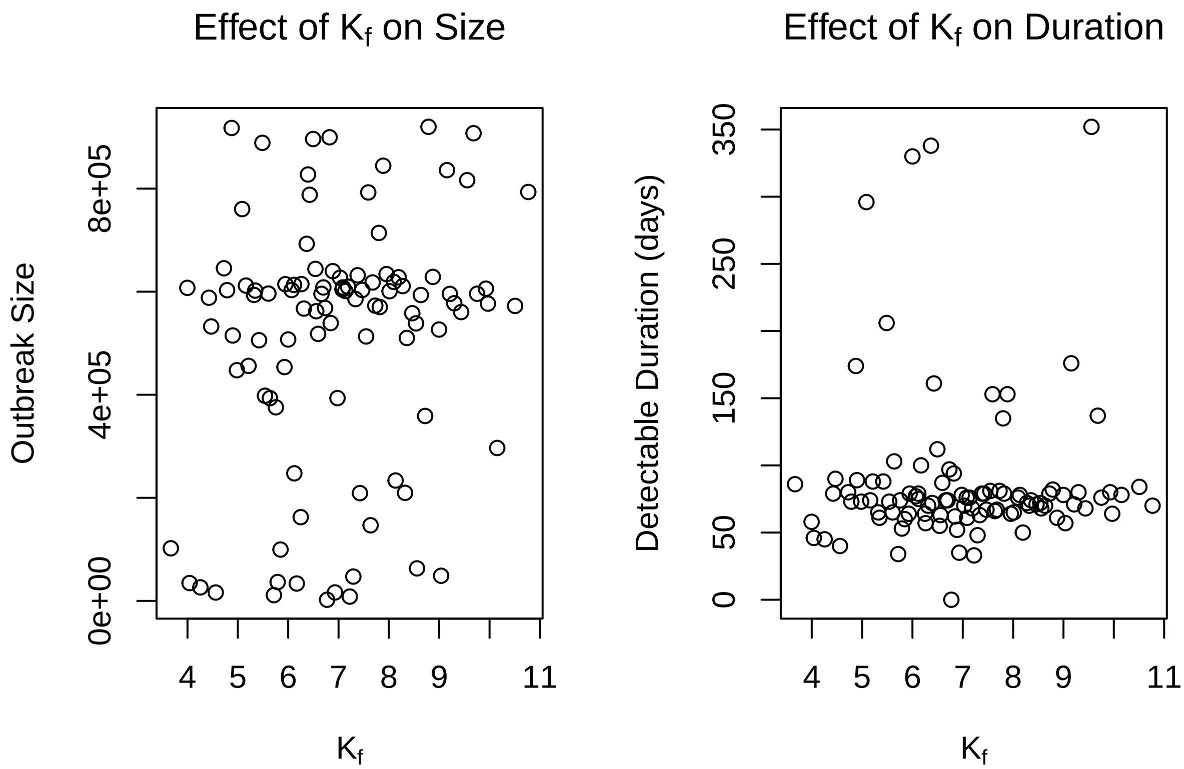


```
plot(bpSEIR$MaxInf ~ bpSEIR$d_f, main = expression(paste("Effect of ", d[f], " on Size")),
    xlab = expression(d[f]), ylab = "Outbreak Size")
plot(bpSEIR$Thresh100 ~ bpSEIR$d_f, main = expression(paste("Effect of ", d[f], " on Duration")),
    xlab = expression(d[f]), ylab = "Detectable Duration (days)")
```

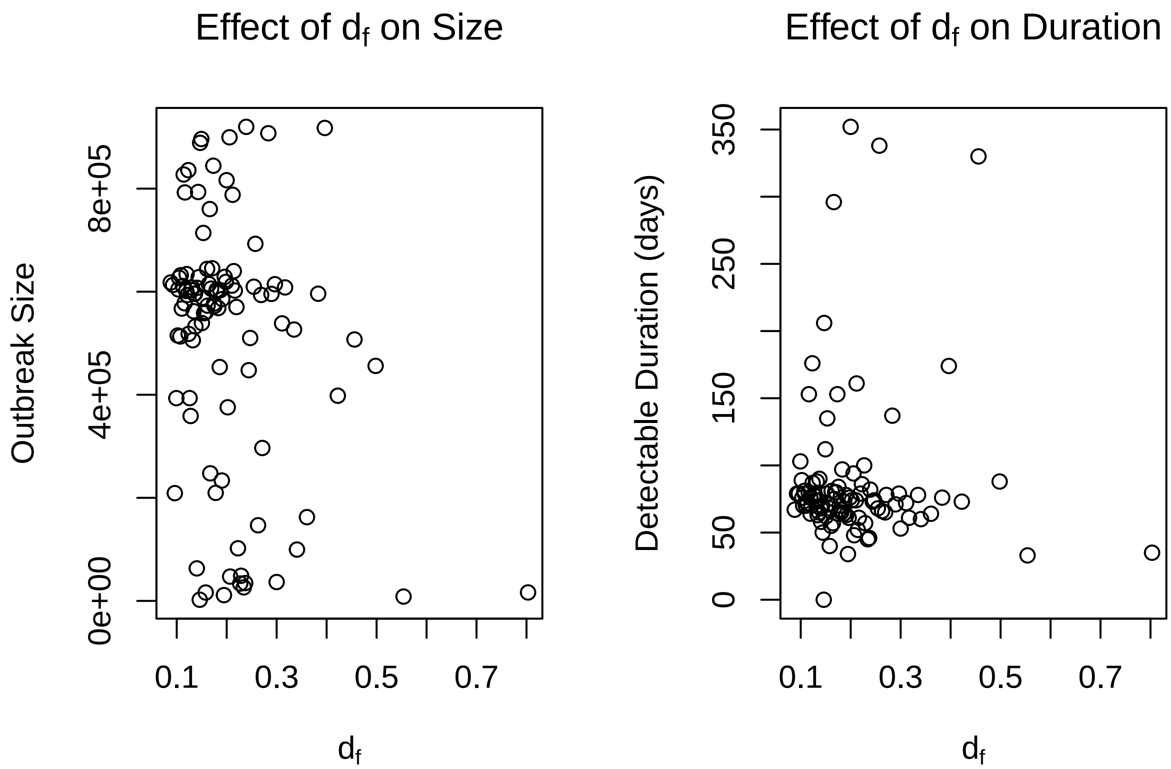


```
plot(bpSEIR$MaxInf ~ bpSEIR$beta_b, main = expression(paste("Effect of ", beta[b],
    " on Size")), xlab = expression(beta[b]), ylab = "Outbreak Size")
plot(bpSEIR$Thresh100 ~ bpSEIR$beta_b, main = expression(paste("Effect of ", beta[b],
    " on Duration")), xlab = expression(beta[b]), ylab = "Detectable Duration (days)")
```

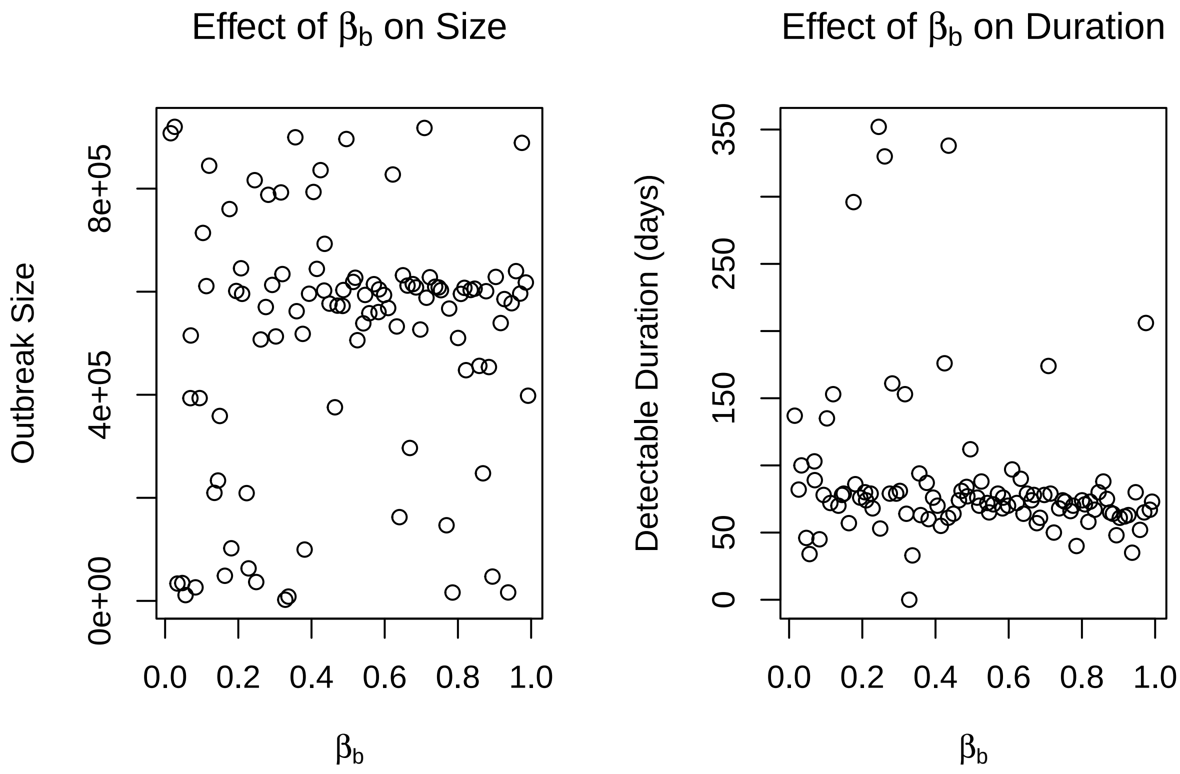


```r
plot(bpSEIR$MaxInf ~ bpSEIR$sigma_b, main = expression(paste("Effect of ", sigma[b],
    " on Size")), xlab = expression(sigma[b]), ylab = "Outbreak Size")
plot(bpSEIR$Thresh100 ~ bpSEIR$sigma_b, main = expression(paste("Effect of ", sigma[b],
    " on Duration")), xlab = expression(sigma[b]), ylab = "Detectable Duration (days)")
```

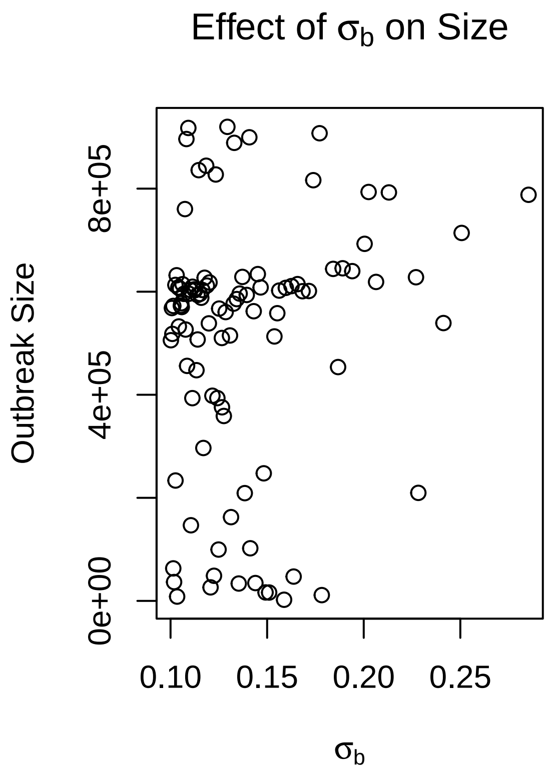

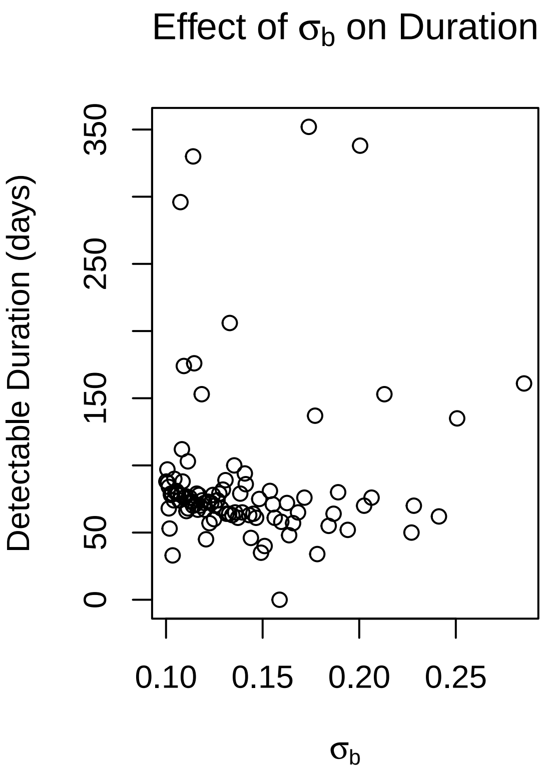


```
plot(bpSEIR$MaxInf ~ bpSEIR$gamma_b, main = expression(paste("Effect of ", gamma[b],
    " on Size")), xlab = expression(gamma[b]), ylab = "Outbreak Size")
plot(bpSEIR$Thresh100 ~ bpSEIR$gamma_b, main = expression(paste("Effect of ", gamma[b],
    " on Duration")), xlab = expression(gamma[b]), ylab = "Detectable Duration (days)")
```

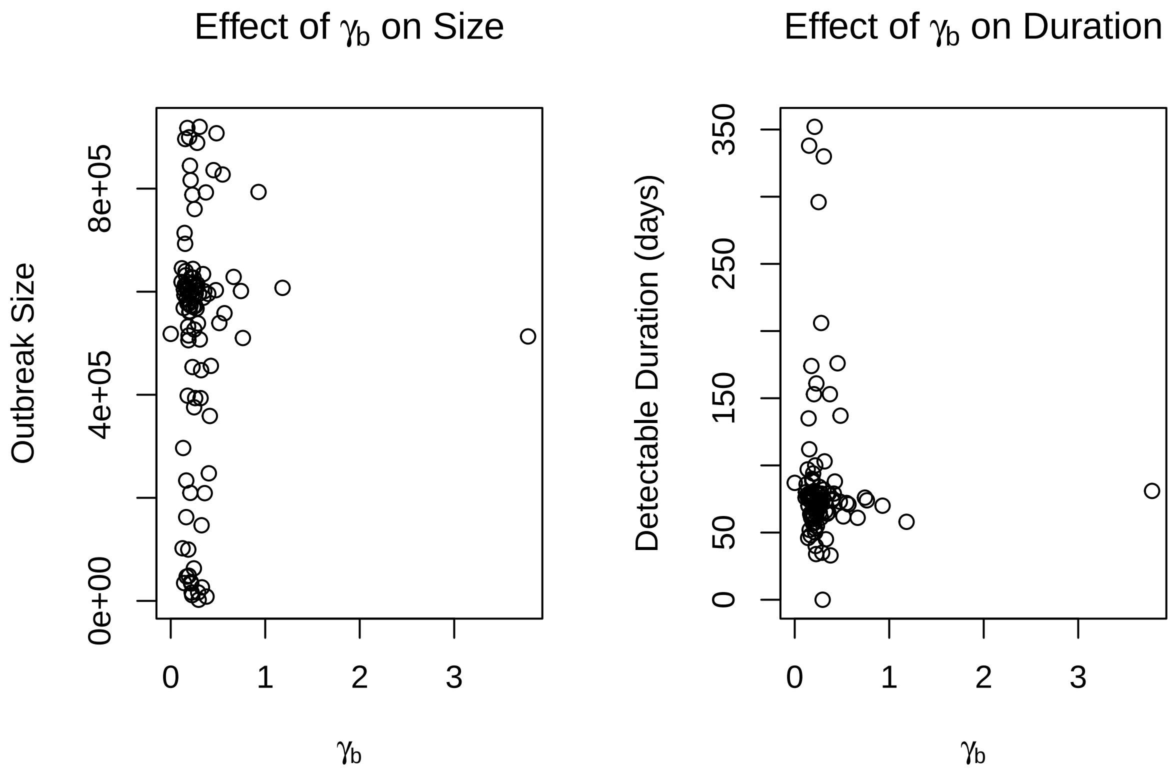


```
plot(bpSEIR$MaxInf ~ bpSEIR$beta_p, main = expression(paste("Effect of ", beta[p],
    " on Size")), xlab = expression(beta[p]), ylab = "Outbreak Size")
plot(bpSEIR$Thresh100 ~ bpSEIR$beta_p, main = expression(paste("Effect of ", beta[p],
    " on Duration")), xlab = expression(beta[p]), ylab = "Detectable Duration (days)")
```

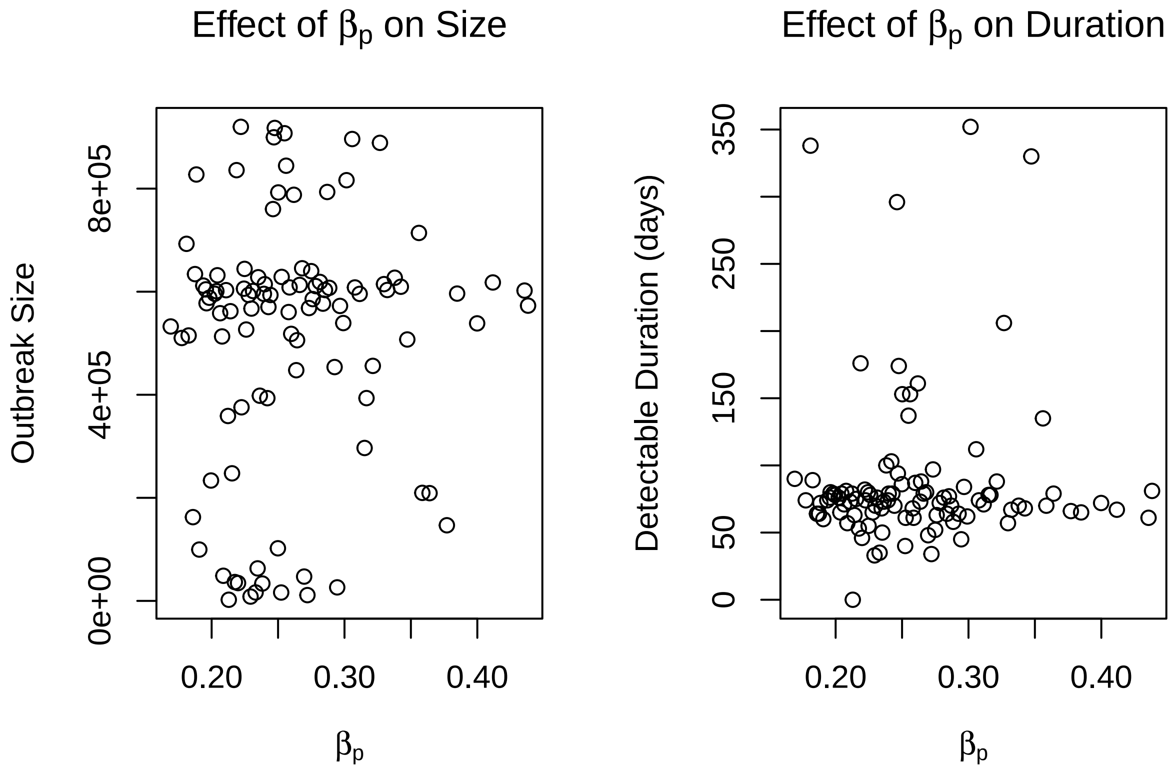


```r
plot(bpSEIR$MaxInf ~ bpSEIR$sigma_p, main = expression(paste("Effect of ", sigma[p],
    " on Size")), xlab = expression(sigma[p]), ylab = "Outbreak Size")
plot(bpSEIR$Thresh100 ~ bpSEIR$sigma_p, main = expression(paste("Effect of ", sigma[p],
    " on Duration")), xlab = expression(sigma[p]), ylab = "Detectable Duration (days)")
```

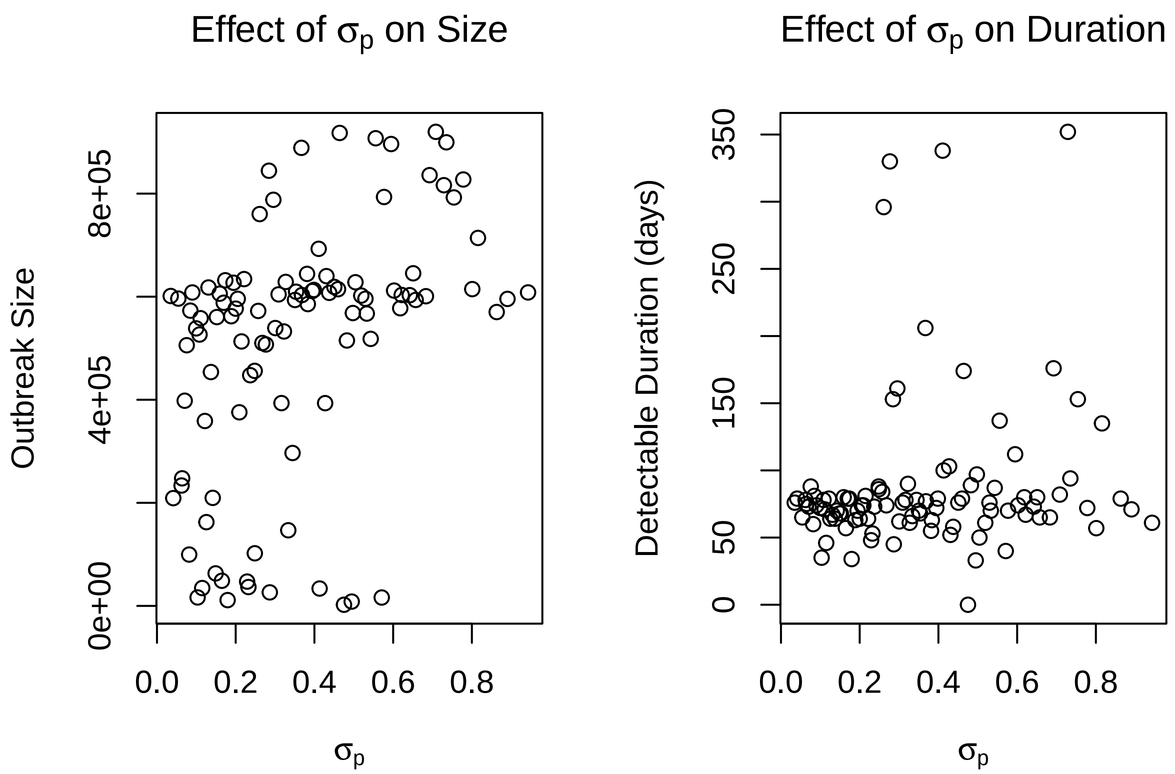


```
plot(bpSEIR$MaxInf ~ bpSEIR$gamma_p, main = expression(paste("Effect of ", gamma[p],
    " on Size")), xlab = expression(gamma[p]), ylab = "Outbreak Size")
plot(bpSEIR$Thresh100 ~ bpSEIR$gamma_p, main = expression(paste("Effect of ", gamma[p],
    " on Duration")), xlab = expression(gamma[p]), ylab = "Detectable Duration (days)")
```

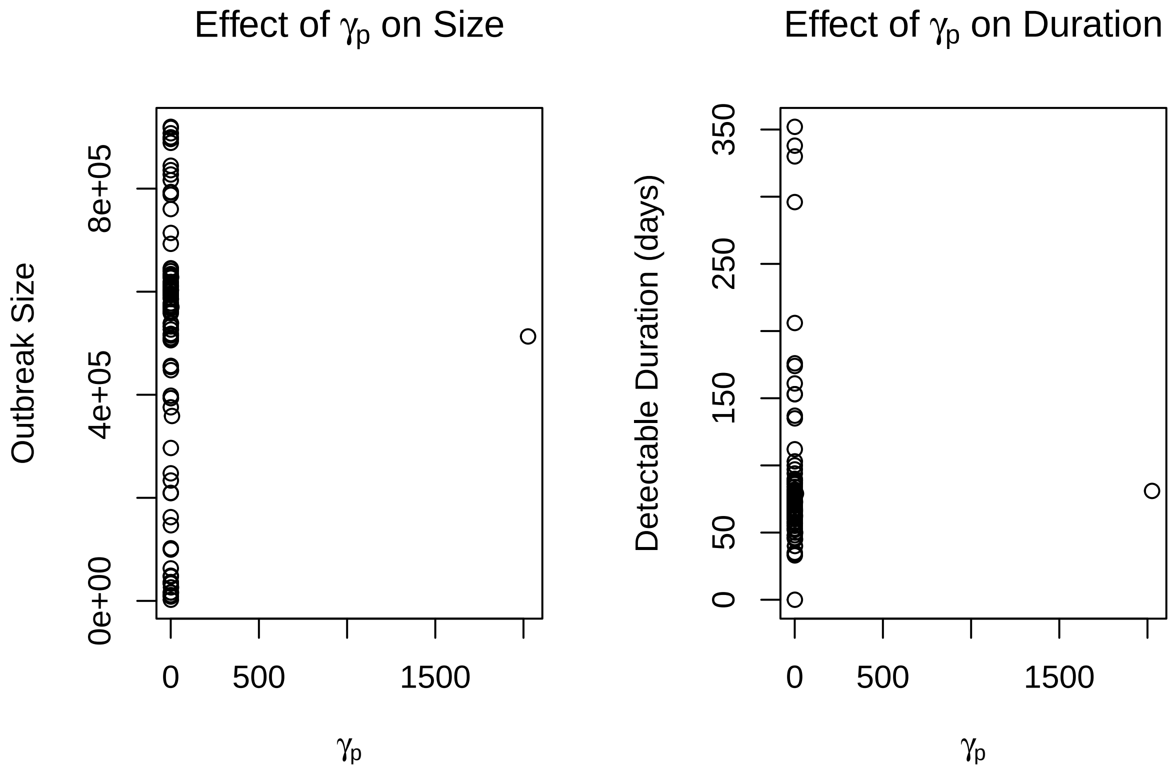


```
plot(bpSEIR$MaxInf ~ bpSEIR$g_h, main = expression(paste("Effect of ", g[h], " on Size")),
    xlab = expression(g[h]), ylab = "Outbreak Size")
plot(bpSEIR$Thresh100 ~ bpSEIR$g_h, main = expression(paste("Effect of ", g[h], " on Duration")),
    xlab = expression(g[h]), ylab = "Detectable Duration (days)")
```

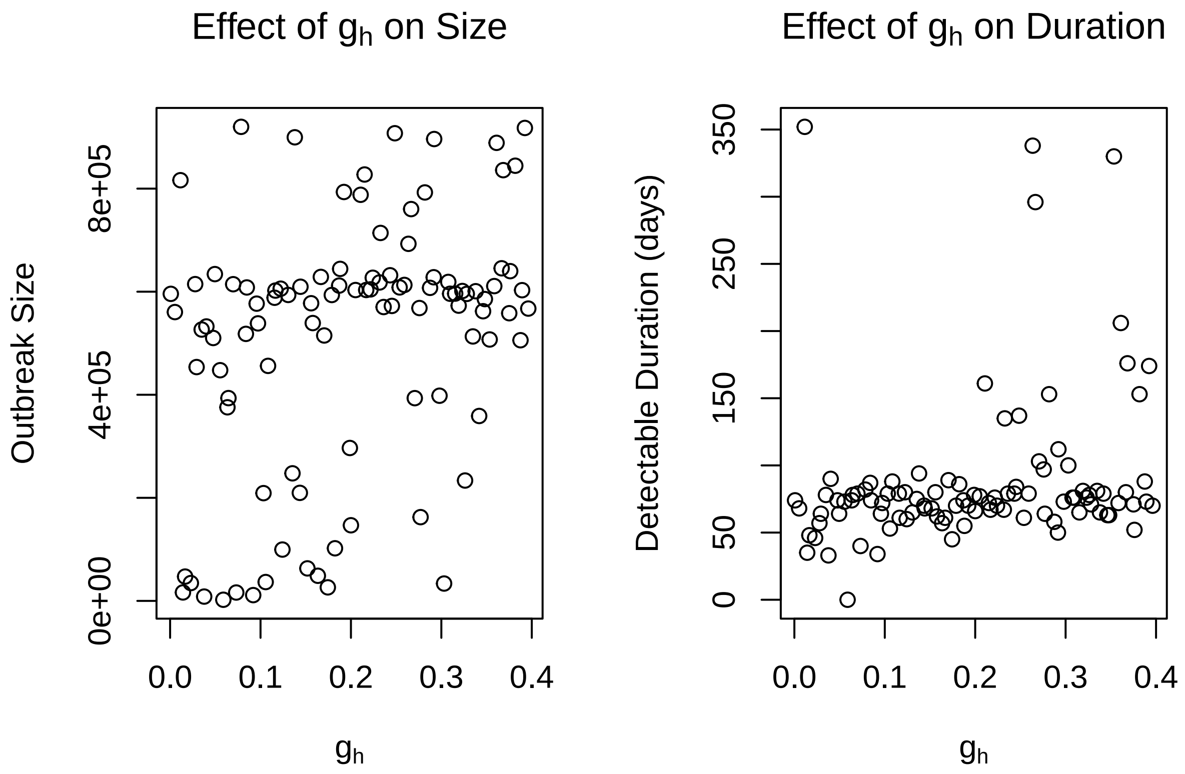


```
plot(bpSEIR$MaxInf ~ bpSEIR$p, main = expression(paste("Effect of ", p, " on Size")),
    xlab = "p", ylab = "Outbreak Size")
plot(bpSEIR$Thresh100 ~ bpSEIR$p, main = expression(paste("Effect of ", p, " on Duration")),
    xlab = "p", ylab = "Detectable Duration (days)")
```

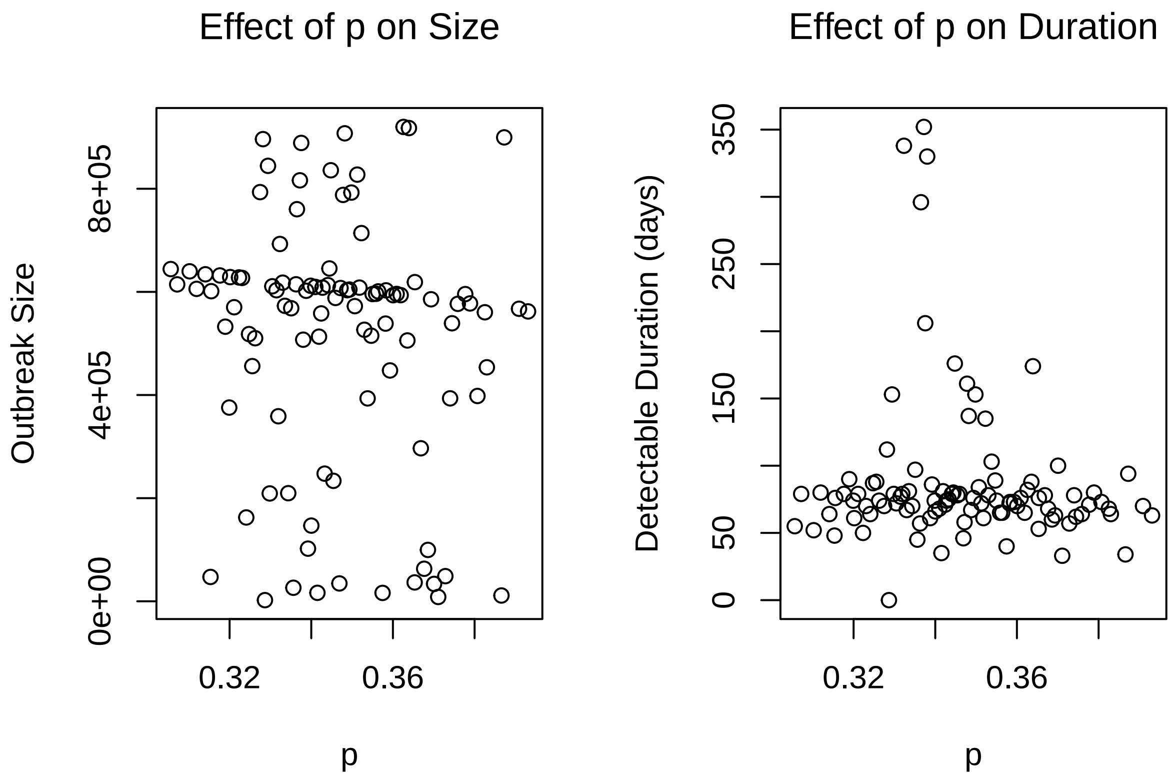


```
plot(bpSEIR$MaxInf ~ bpSEIR$b_h, main = expression(paste("Effect of ", b[h], " on Size")),
    xlab = expression(b[h]), ylab = "Outbreak Size")
plot(bpSEIR$Thresh100 ~ bpSEIR$b_h, main = expression(paste("Effect of ", b[h], " on Duration")),
    xlab = expression(b[h]), ylab = "Detectable Duration (days)")
```

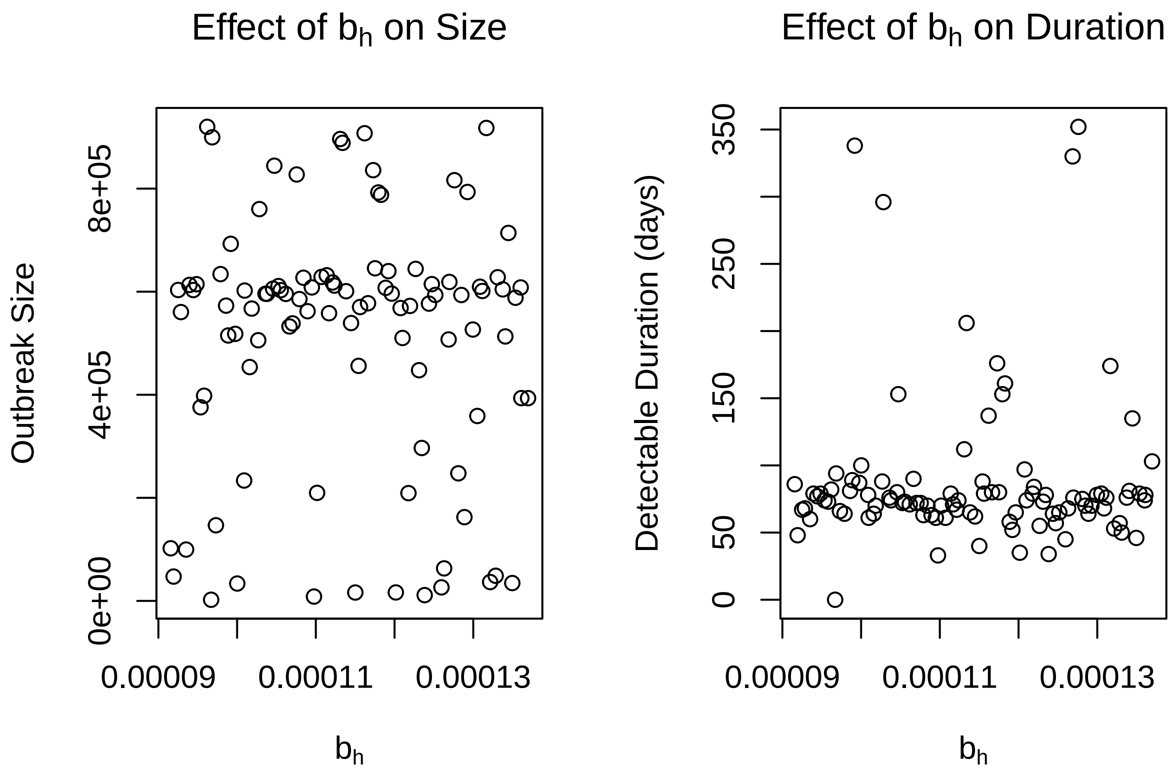


```
plot(bpSEIR$MaxInf ~ bpSEIR$d_h, main = expression(paste("Effect of ", d[h], " on Size")),
    xlab = expression(d[h]), ylab = "Outbreak Size")
plot(bpSEIR$Thresh100 ~ bpSEIR$d_h, main = expression(paste("Effect of ", d[h], " on Duration")),
    xlab = expression(d[h]), ylab = "Detectable Duration (days)")
```

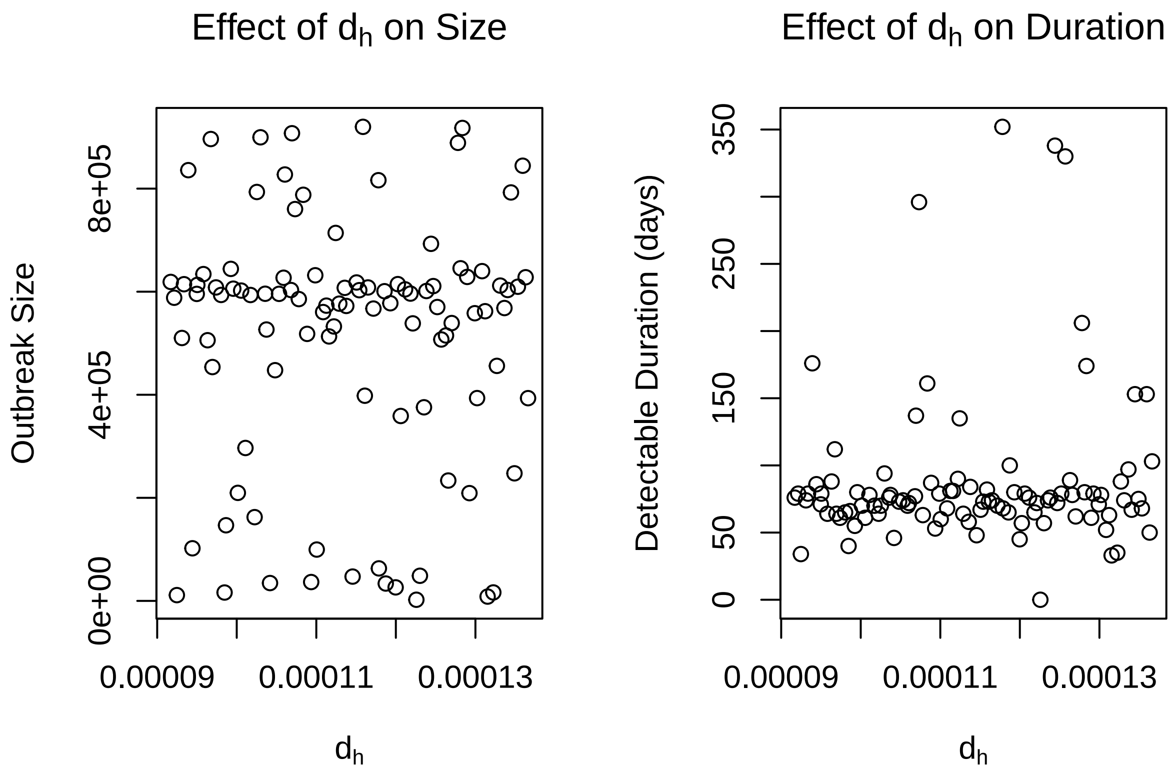


```r
par(mfrow = c(1, 2))
boxplot(bpSEIR$MaxInf, main = "Outbreak Size", ylab = "Number of Dead Humans", ylim = c(0,
    923406))
boxplot(bpSEIR$Thresh100, main = "Outbreak Duration", ylab = "Time (Days)")
```

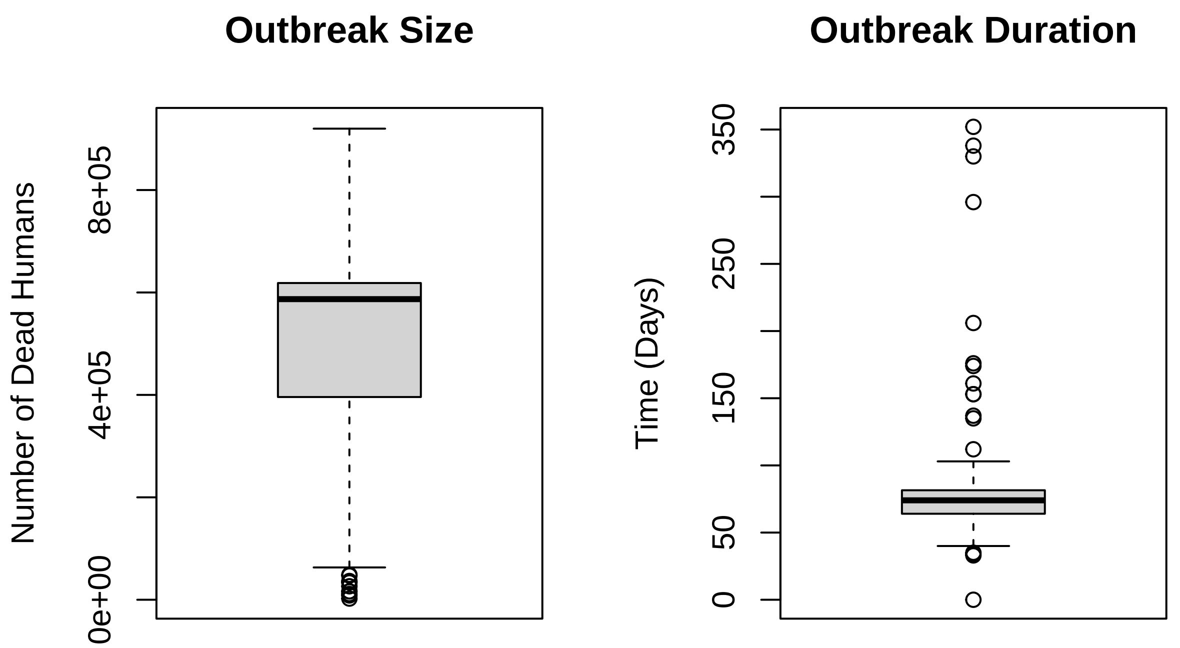


```
bonferroni.alpha <- 0.05/length(parameters)
prcc_size <- pcc(bpSEIR[, 1:length(parameters)], bpSEIR[, length(parameters) + 1],
    nboot = niter, rank = TRUE, conf = 1 - bonferroni.alpha)
prcc_duration <- pcc(bpSEIR[, 1:length(parameters)], bpSEIR[, length(parameters) +
    2], nboot = niter, rank = TRUE, conf = 1 - bonferroni.alpha)

# plot correlation coefficients and confidence intervals for epidemic size and
# duration
size <- prcc_size$PRCC
size$param <- rownames(size)
colnames(size)[4:5] <- c("maxCI", "minCI")
size$maxCI[which(size$maxCI > 1)] <- 1
size$maxCI[which(size$maxCI < -1)] <- -1
size$minCI[which(size$minCI > 1)] <- 1
size$minCI[which(size$minCI < -1)] <- -1

duration <- prcc_duration$PRCC
duration$param <- rownames(duration)
colnames(duration)[4:5] <- c("maxCI", "minCI")
duration$maxCI[which(duration$maxCI > 1)] <- 1
duration$maxCI[which(duration$maxCI < -1)] <- -1
duration$minCI[which(duration$minCI > 1)] <- 1
duration$minCI[which(duration$minCI < -1)] <- -1


A <- ggplot(size, aes(x = param, y = original)) + geom_point(size = 4) + geom_errorbar(aes(ymax = maxCI
```

```
    ymin = minCI)) + ggtitle("A") + xlab("Parameters") + ylab("Partial Rank Correlation Coefficients") +
    scale_x_discrete(labels = c(alpha = expression(alpha), beta_b = expression(beta[b]),
        beta_p = expression(beta[p]), beta_r = expression(beta[r]), b_h = expression(b[h]),
        d_h = expression(d[h]), d_f = expression(d[f]), sigma_b = expression(sigma[b]),
        gamma_b = expression(gamma[b]), sigma_p = expression(sigma[p]), gamma_p = expression(gamma[p]),
        gamma_r = expression(gamma[r]), g_h = expression(g[h]), g_r = expression(g[r]),
        K_f = expression(K[f]), r_f = expression(r[f]))) + ylim(-1, 1)


B <- ggplot(duration, aes(x = param, y = original)) + geom_point(size = 4) + geom_errorbar(aes(ymax = ma
    ymin = minCI)) + ggtitle("B") + xlab("Parameters") + ylab(" ") + scale_x_discrete(labels = c(alpha =
    beta_b = expression(beta[b]), beta_p = expression(beta[p]), beta_r = expression(beta[r]),
    b_h = expression(b[h]), d_h = expression(d[h]), d_f = expression(d[f]), sigma_b = expression(sigma[b
    gamma_b = expression(gamma[b]), sigma_p = expression(sigma[p]), gamma_p = expression(gamma[p]),
    gamma_r = expression(gamma[r]), g_h = expression(g[h]), g_r = expression(g[r]),
    K_f = expression(K[f]), r_f = expression(r[f]))) + ylim(-1, 1)

multiplot(A, B, cols = 2)
```

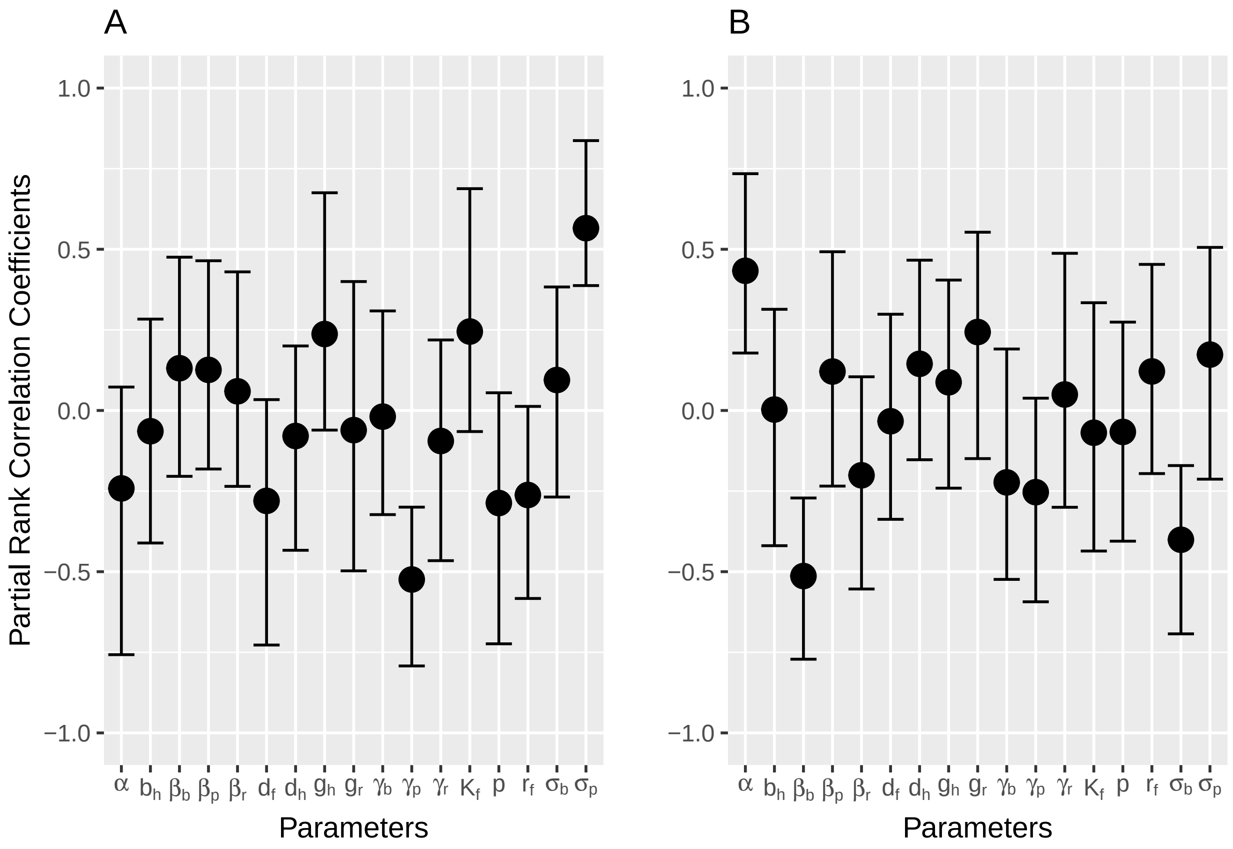


```
tiff("FigS6.tiff", height = 10, width = 19.05, units = "cm", compression = "lzw",
    res = 600)
multiplot(A, B, cols = 2)
dev.off()


## pdf
```

```
## 	2
```

Smallpox SIR - Figure S7 (Panels A & B)

```r
parameters <- c(beta_s = 0.584, gamma_s = 1/9.5, g_s = 0.05, b_h = 1/(25 * 365),
    d_h = 1/(25 * 365))  #you can play with transmission and recovery rates here

# plot scatterplots
par(mfrow = c(1, 2))
plot(sSIR$MaxInf ~ sSIR$beta_s, main = expression(paste("Effect of ", beta[s], " on Size")),
    xlab = expression(beta[s]), ylab = "Outbreak Size")
plot(sSIR$Thresh100 ~ sSIR$beta_s, main = expression(paste("Effect of ", beta[s],
    " on Duration")), xlab = expression(beta[s]), ylab = "Observable Duration (days)")
```

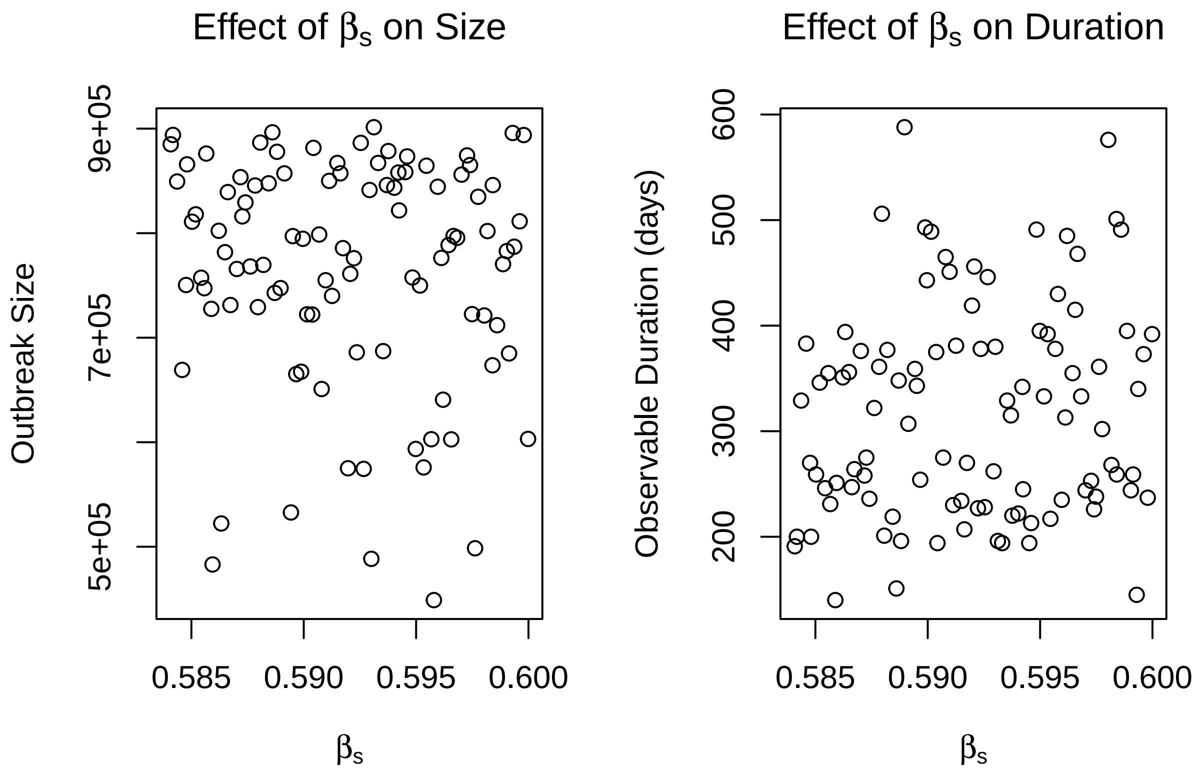


```r
plot(sSIR$MaxInf ~ sSIR$gamma_s, main = expression(paste("Effect of ", gamma[s],
    " on Size")), xlab = expression(gamma[s]), ylab = "Outbreak Size")
plot(sSIR$Thresh100 ~ sSIR$gamma_s, main = expression(paste("Effect of ", gamma[s],
    " on Duration")), xlab = expression(gamma[s]), ylab = "Observable Duration (days)")
```

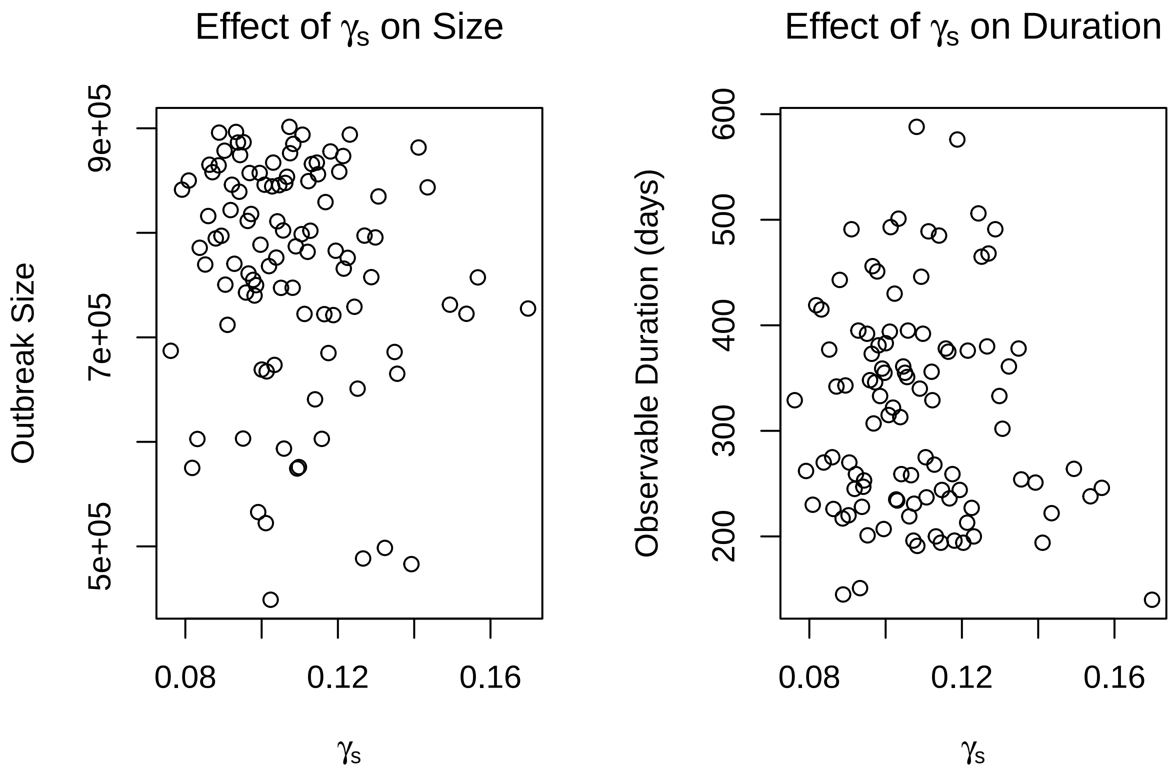


```
plot(sSIR$MaxInf ~ sSIR$g_s, main = expression(paste("Effect of ", g[s], " on Size")),
    xlab = expression(g[s]), ylab = "Outbreak Size")
plot(sSIR$Thresh100 ~ sSIR$g_s, main = expression(paste("Effect of ", g[s], " on Duration")),
    xlab = expression(g[s]), ylab = "Detectable Duration (days)")
```

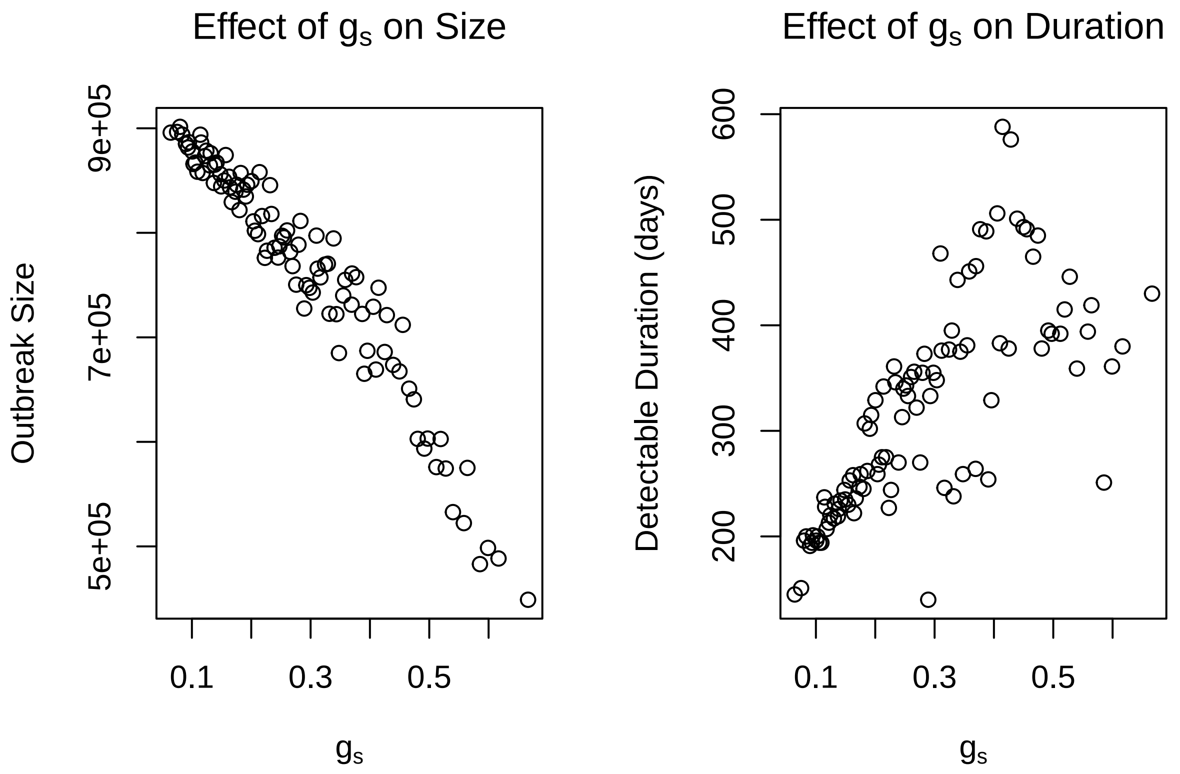


```
plot(sSIR$MaxInf ~ sSIR$b_h, main = expression(paste("Effect of ", b[h], " on Size")),
    xlab = expression(b[h]), ylab = "Outbreak Size")
plot(sSIR$Thresh100 ~ sSIR$b_h, main = expression(paste("Effect of ", b[h], " on Duration")),
    xlab = expression(b[h]), ylab = "Observable Duration (days)")
```

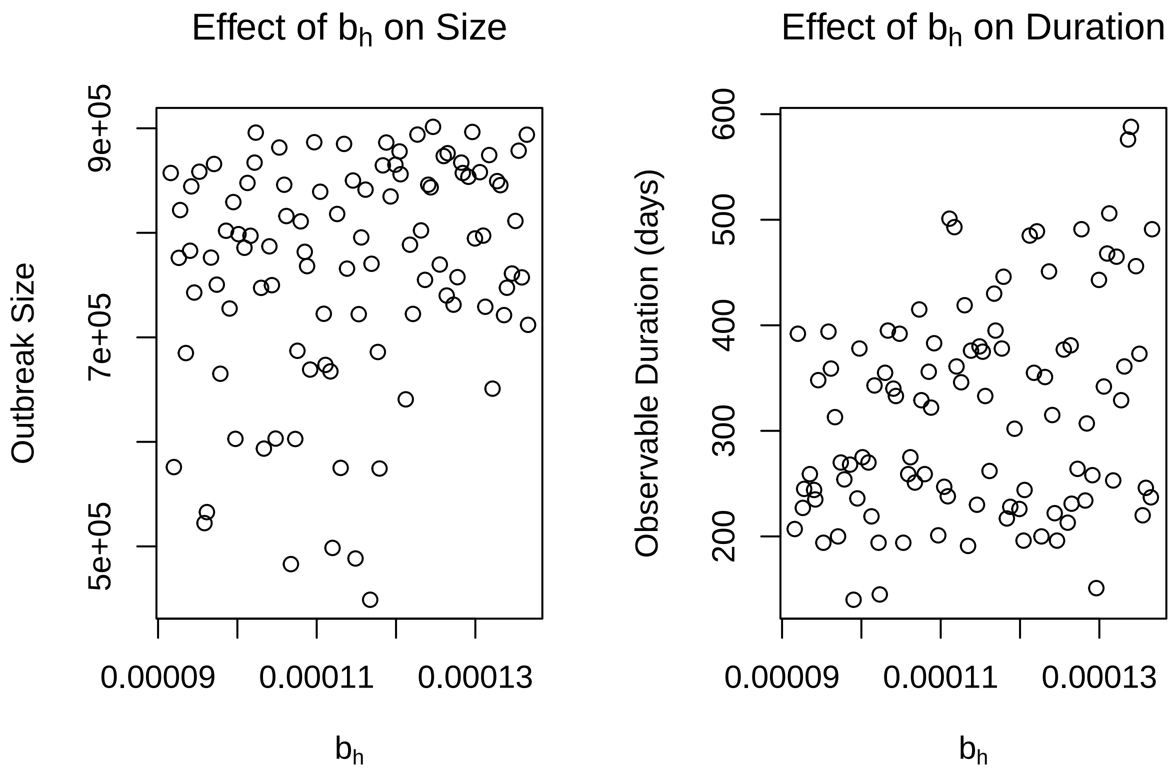


```
plot(sSIR$MaxInf ~ sSIR$d_h, main = expression(paste("Effect of ", d[h], " on Size")),
    xlab = expression(d[h]), ylab = "Outbreak Size")
plot(sSIR$Thresh100 ~ sSIR$d_h, main = expression(paste("Effect of ", d[h], " on Duration")),
    xlab = expression(d[h]), ylab = "Observable Duration (days)")
```

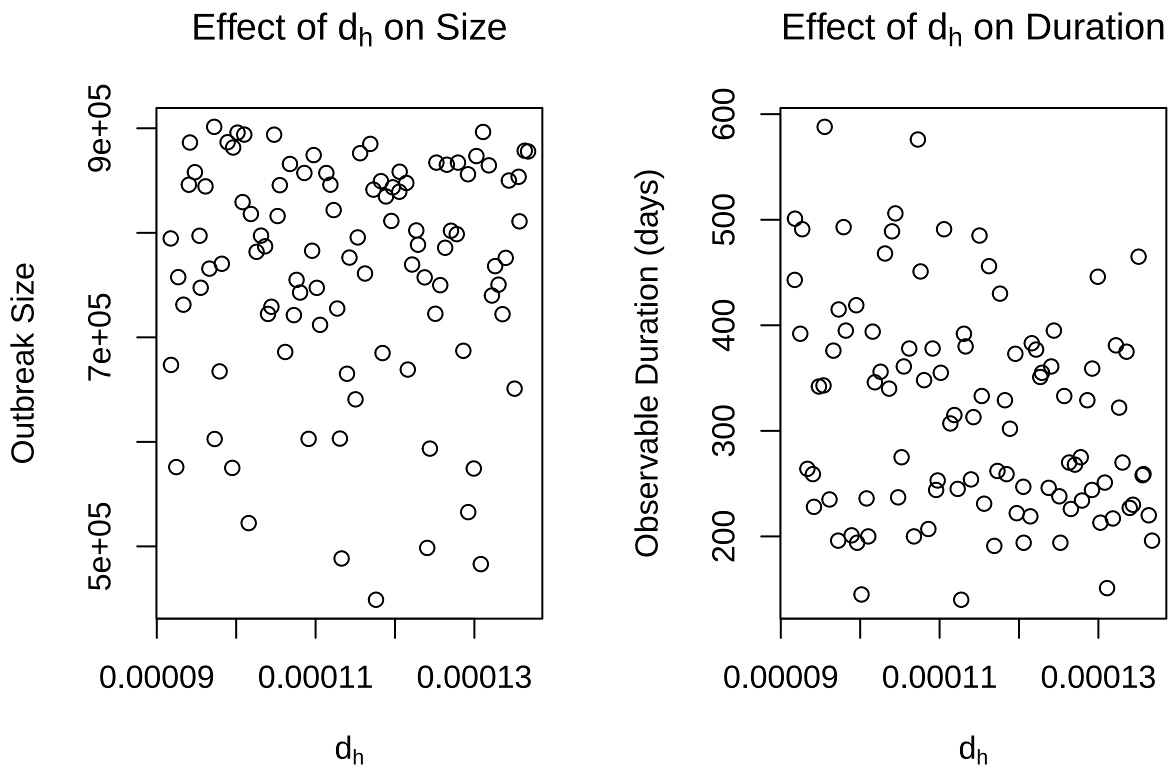


```
par(mfrow = c(1, 2))
boxplot(sSIR$MaxInf, main = "Outbreak Size", ylab = "Number of Dead Humans", ylim = c(0,
    923406))
boxplot(sSIR$Thresh100, main = "Observable Outbreak Duration", ylab = "Time (Days)")
```

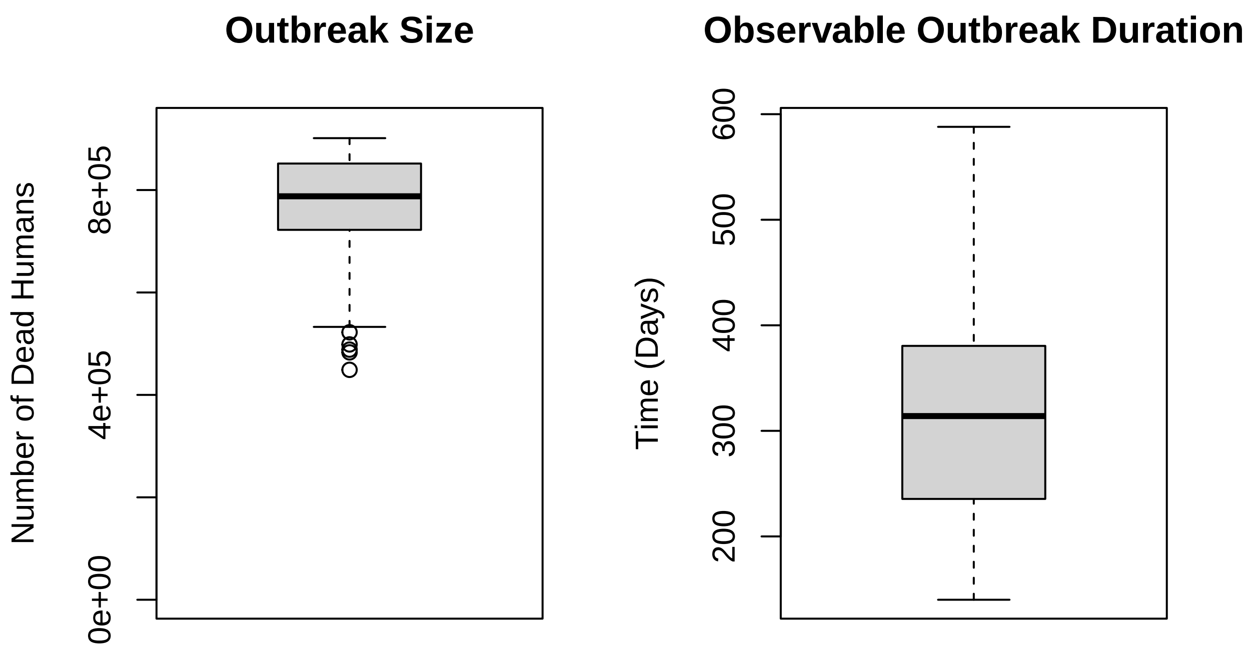


```
bonferroni.alpha <- 0.05/length(parameters)
prcc_size <- pcc(sSIR[, 1:length(parameters)], sSIR$MaxInf, nboot = niter, rank = TRUE,
    conf = 1 - bonferroni.alpha)
prcc_duration <- pcc(sSIR[, 1:length(parameters)], sSIR$Thresh100, nboot = niter,
    rank = TRUE, conf = 1 - bonferroni.alpha)

# plot correlation coefficients and confidence intervals for epidemic size and
# duration
size <- prcc_size$PRCC
size$param <- rownames(size)
colnames(size)[4:5] <- c("maxCI", "minCI")
size$maxCI[which(size$maxCI > 1)] <- 1
size$maxCI[which(size$maxCI < -1)] <- -1
size$minCI[which(size$minCI > 1)] <- 1
size$minCI[which(size$minCI < -1)] <- -1

duration <- prcc_duration$PRCC
duration$param <- rownames(duration)
colnames(duration)[4:5] <- c("maxCI", "minCI")
duration$maxCI[which(duration$maxCI > 1)] <- 1
duration$maxCI[which(duration$maxCI < -1)] <- -1
duration$minCI[which(duration$minCI > 1)] <- 1
duration$minCI[which(duration$minCI < -1)] <- -1


A <- ggplot(size, aes(x = param, y = original)) + geom_point(size = 4) + geom_errorbar(aes(ymax = maxCI
```

```
    ymin = minCI)) + ggtitle("A") + xlab("Parameters") + ylab("Partial Rank Correlation Coefficients") +
    scale_x_discrete(labels = c(beta_s = expression(beta[s]), b_h = expression(b[h]),
        d_h = expression(d[h]), gamma_s = expression(gamma[s]), g_s = expression(g[s]))) +
    ylim(-1, 1)

B <- ggplot(duration, aes(x = param, y = original)) + geom_point(size = 4) + geom_errorbar(aes(ymax = ma
    ymin = minCI)) + ggtitle("B") + xlab("Parameters") + ylab(" ") + scale_x_discrete(labels = c(beta_s
    b_h = expression(b[h]), d_h = expression(d[h]), gamma_s = expression(gamma[s]),
    g_s = expression(g[s]))) + ylim(-1, 1)
```

Smallpox SEIR - Figure S7 (Panels C & D)

```
parameters <- c(beta_s = 0.584, sigma_s = 1/12, gamma_s = 1/9.5, g_s = 0.05, b_h = 1/(25 *
    365), d_h = 1/(25 * 365))  #you can play with transmission and recovery rates here

# plot scatterplots
par(mfrow = c(1, 2))
plot(sSEIR$MaxInf ~ sSEIR$beta_s, main = expression(paste("Effect of ", beta[s],
    " on Size")), xlab = expression(beta[s]), ylab = "Outbreak Size")
plot(sSEIR$Thresh100 ~ sSEIR$beta_s, main = expression(paste("Effect of ", beta[s],
    " on Duration")), xlab = expression(beta[s]), ylab = "Observable Duration (days)")
```

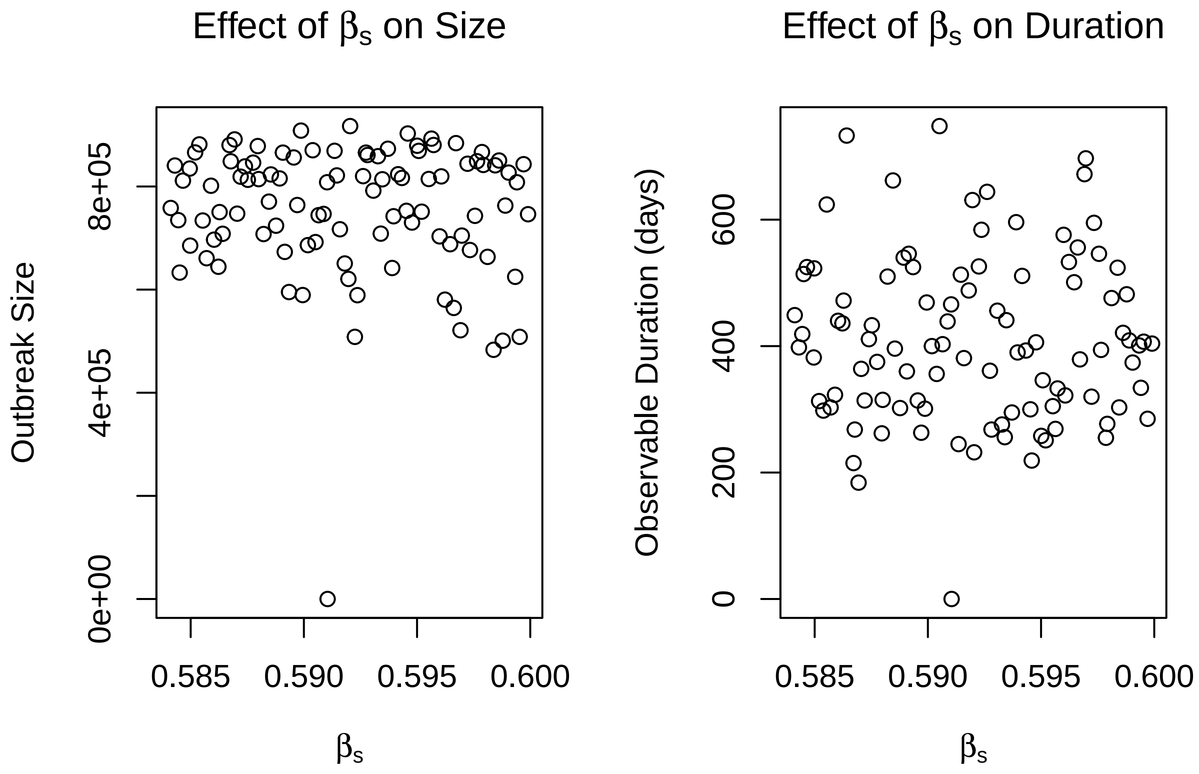

```
plot(sSEIR$MaxInf ~ sSEIR$sigma_s, main = expression(paste("Effect of ", sigma[s],
    " on Size")), xlab = expression(sigma[s]), ylab = "Outbreak Size")
plot(sSEIR$Thresh100 ~ sSEIR$sigma_s, main = expression(paste("Effect of ", sigma[s],
    " on Duration")), xlab = expression(sigma[s]), ylab = "Detectable Outbreak Duration (days)")
```

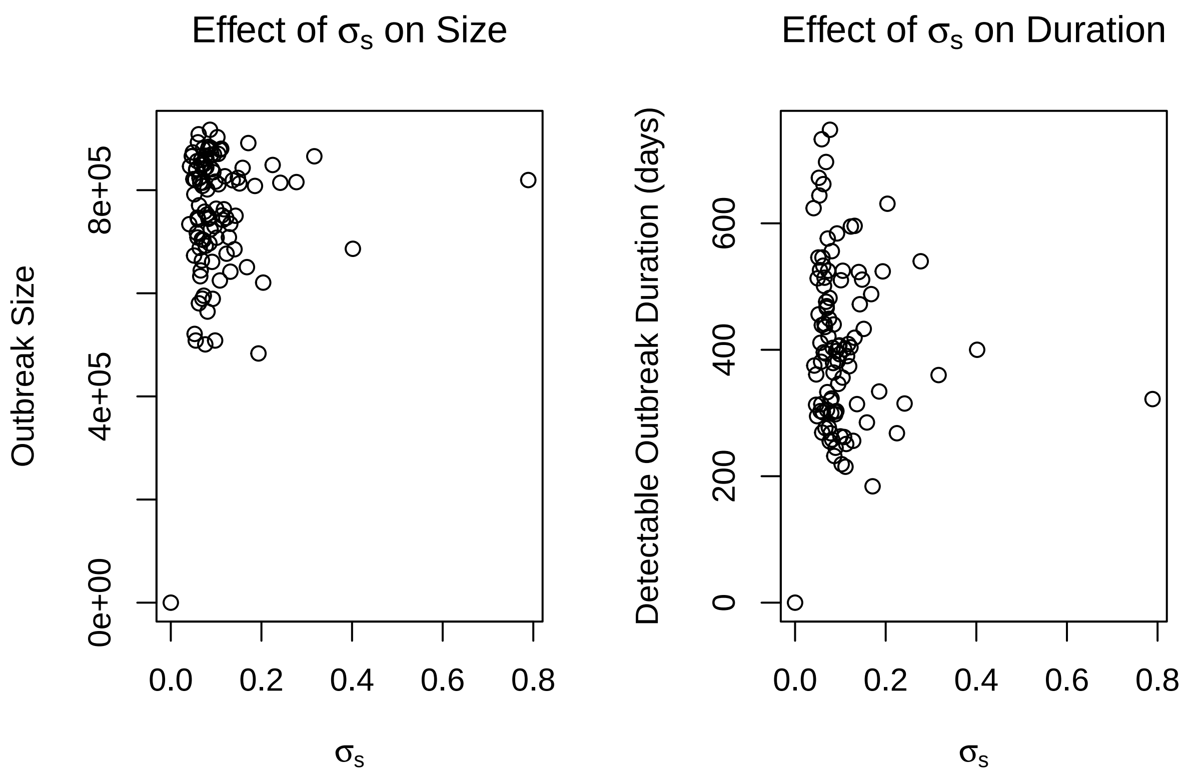


```
plot(sSEIR$MaxInf ~ sSEIR$gamma_s, main = expression(paste("Effect of ", gamma[s],
    " on Size")), xlab = expression(gamma[s]), ylab = "Outbreak Size")
plot(sSEIR$Thresh100 ~ sSEIR$gamma_s, main = expression(paste("Effect of ", gamma[s],
    " on Duration")), xlab = expression(gamma[s]), ylab = "Observable Duration (days)")
```

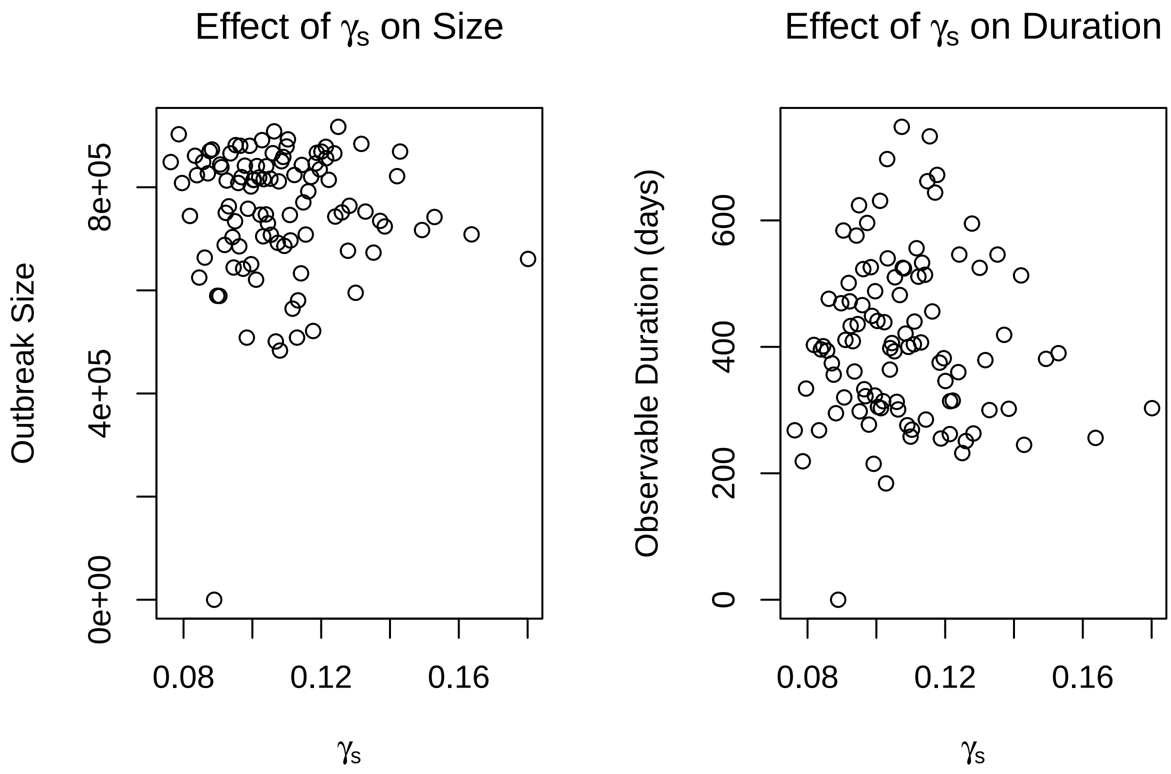


```
plot(sSEIR$MaxInf ~ sSEIR$g_s, main = expression(paste("Effect of ", g[s], " on Size")),
    xlab = expression(g[s]), ylab = "Outbreak Size")
plot(sSEIR$Thresh100 ~ sSEIR$g_s, main = expression(paste("Effect of ", g[s], " on Duration")),
    xlab = expression(g[s]), ylab = "Detectable Duration (days)")
```

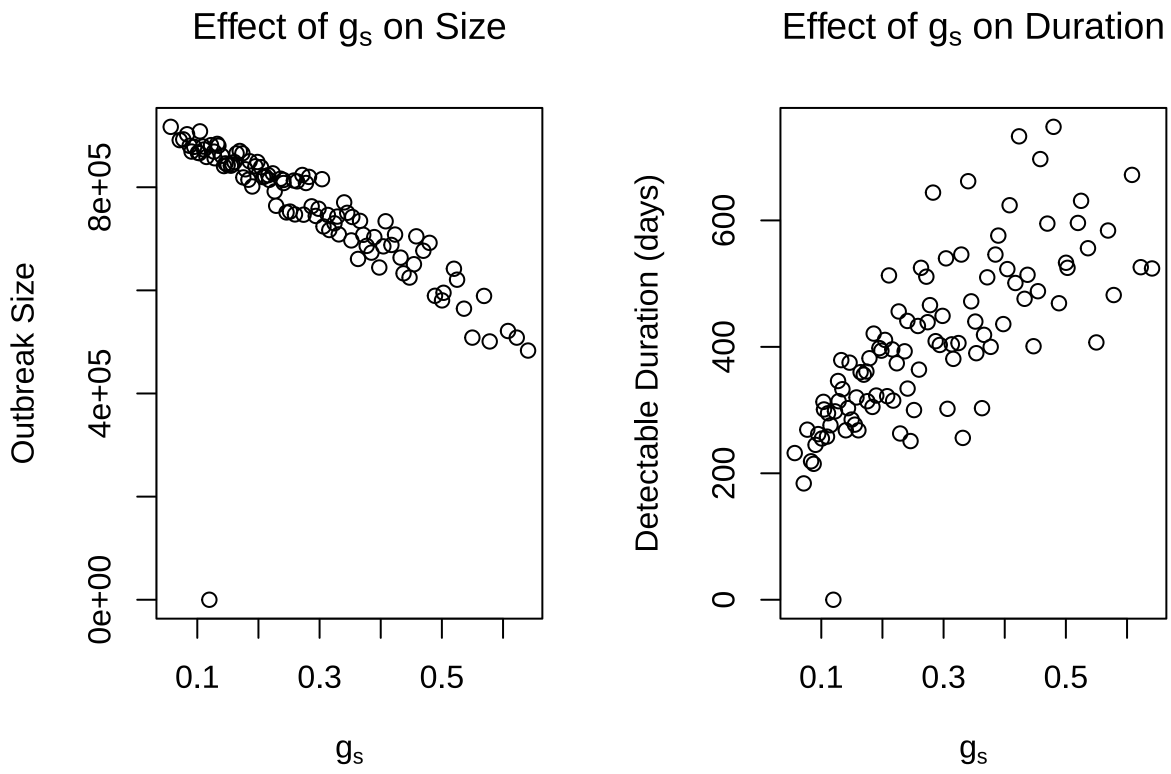


```
plot(sSEIR$MaxInf ~ sSEIR$b_h, main = expression(paste("Effect of ", b[h], " on Size")),
    xlab = expression(b[h]), ylab = "Outbreak Size")
plot(sSEIR$Thresh100 ~ sSEIR$b_h, main = expression(paste("Effect of ", b[h], " on Duration")),
    xlab = expression(b[h]), ylab = "Observable Duration (days)")
```

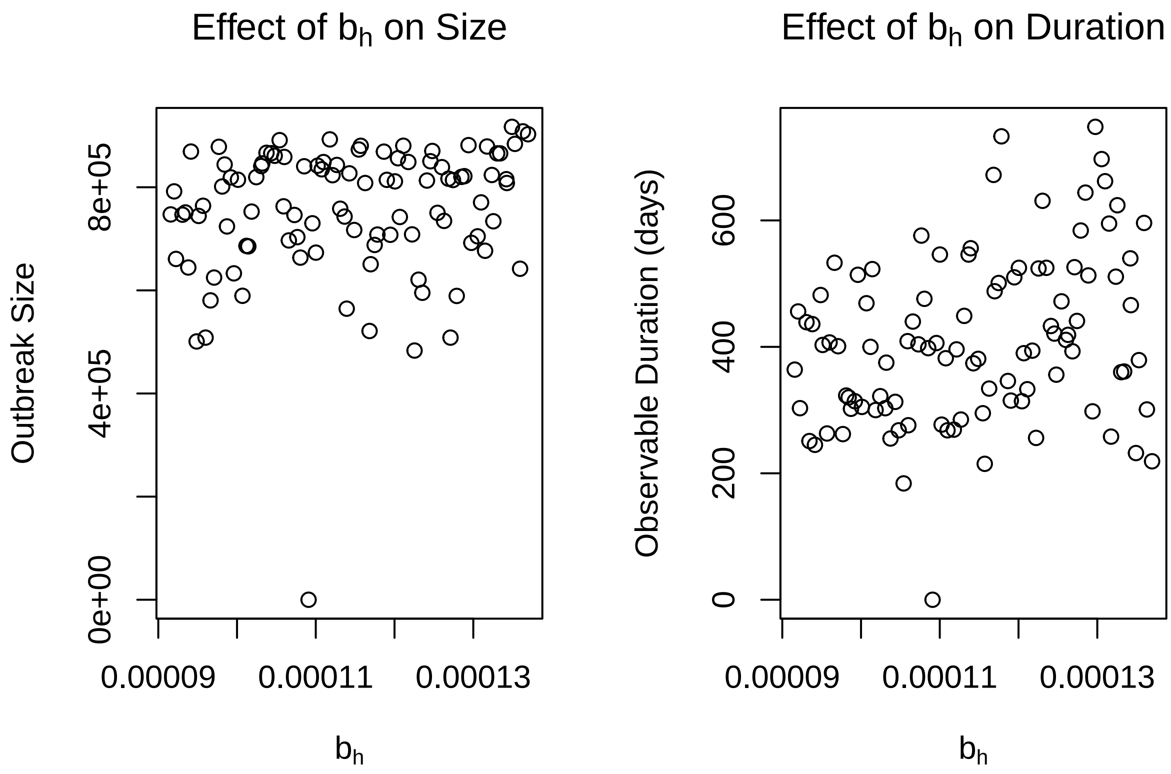


```
plot(sSEIR$MaxInf ~ sSEIR$d_h, main = expression(paste("Effect of ", d[h], " on Size")),
    xlab = expression(d[h]), ylab = "Outbreak Size")
plot(sSEIR$Thresh100 ~ sSEIR$d_h, main = expression(paste("Effect of ", d[h], " on Duration")),
    xlab = expression(d[h]), ylab = "Observable Duration (days)")
```

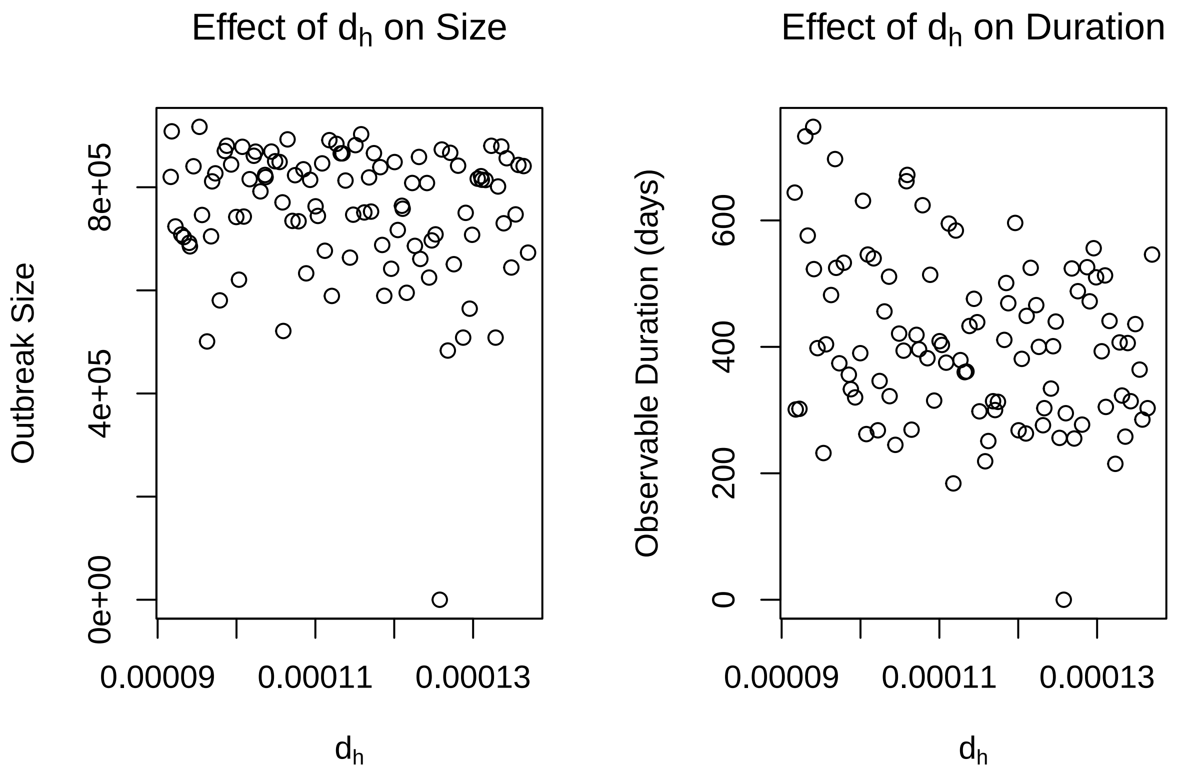


```
par(mfrow = c(1, 2))
boxplot(sSEIR$MaxInf, main = "Outbreak Size", ylab = "Number of Dead Humans", ylim = c(0,
    923406))
boxplot(sSEIR$Thresh100, main = "Outbreak Duration", ylab = "Time (Days)")
```

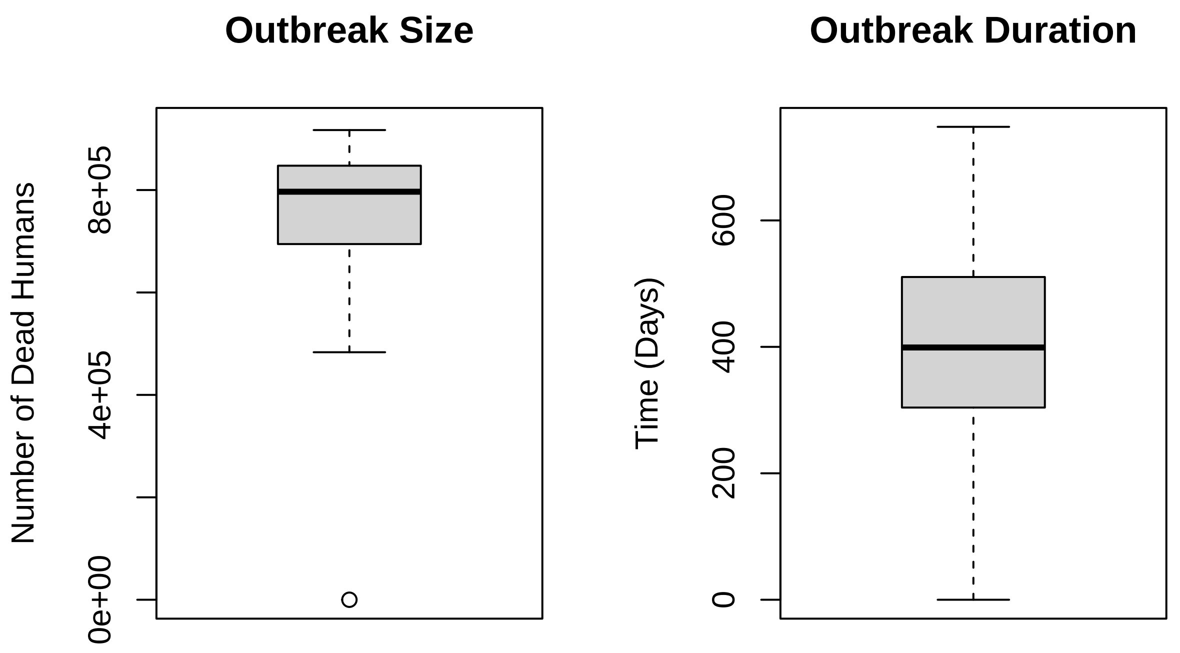


```
bonferroni.alpha <- 0.05/length(parameters)
prcc_size <- pcc(sSEIR[, 1:length(parameters)], sSEIR$MaxInf, nboot = niter, rank = TRUE,
    conf = 1 - bonferroni.alpha)
prcc_duration <- pcc(sSEIR[, 1:length(parameters)], sSEIR$Thresh100, nboot = niter,
    rank = TRUE, conf = 1 - bonferroni.alpha)

# plot correlation coefficients and confidence intervals for epidemic size and
# duration
size <- prcc_size$PRCC
size$param <- rownames(size)
colnames(size)[4:5] <- c("maxCI", "minCI")
size$maxCI[which(size$maxCI > 1)] <- 1
size$maxCI[which(size$maxCI < -1)] <- -1
size$minCI[which(size$minCI > 1)] <- 1
size$minCI[which(size$minCI < -1)] <- -1

duration <- prcc_duration$PRCC
duration$param <- rownames(duration)
colnames(duration)[4:5] <- c("maxCI", "minCI")
duration$maxCI[which(duration$maxCI > 1)] <- 1
duration$maxCI[which(duration$maxCI < -1)] <- -1
duration$minCI[which(duration$minCI > 1)] <- 1
duration$minCI[which(duration$minCI < -1)] <- -1

C <- ggplot(size, aes(x = param, y = original)) + geom_point(size = 4) + geom_errorbar(aes(ymax = maxCI
    ymin = minCI)) + ggtitle("C") + xlab("Parameters") + ylab("Partial Rank Correlation Coefficients")
```

```r
    scale_x_discrete(labels = c(beta_s = expression(beta[s]), b_h = expression(b[h]),
        d_h = expression(d[h]), gamma_s = expression(gamma[s]), g_s = expression(g[s]),
        sigma_s = expression(sigma[s]))) + ylim(-1, 1)

D <- ggplot(duration, aes(x = param, y = original)) + geom_point(size = 4) + geom_errorbar(aes(ymax = ma
    ymin = minCI)) + ggtitle("D") + xlab("Parameters") + ylab(" ") + scale_x_discrete(labels = c(beta_s
    b_h = expression(b[h]), d_h = expression(d[h]), gamma_s = expression(gamma[s]),
    g_s = expression(g[s]), sigma_s = expression(sigma[s]))) + ylim(-1, 1)

tiff("FigS7.tiff", height = 22.23, width = 19.05, units = "cm", compression = "lzw",
    res = 600)
multiplot(A, B, C, D, cols = 2)
dev.off()
```

```
## pdf
##   2
```

Measles SIR - Figure S8 (Panels A & B)

```r
parameters <- c(beta_m = 1.175, gamma_m = 1/13, g_m = 0.7, b_h = 1/(25 * 365), d_h = 1/(25 *
    365))  #you can play with transmission and recovery rates here

# plot scatterplots
par(mfrow = c(1, 2))
plot(mSIR$MaxInf ~ mSIR$beta_m, main = expression(paste("Effect of ", beta[m], " on Size")),
    xlab = expression(beta[m]), ylab = "Outbreak Size")
plot(mSIR$Thresh100 ~ mSIR$beta_m, main = expression(paste("Effect of ", beta[m],
    " on Duration")), xlab = expression(beta[m]), ylab = "Observable Duration (days)")
```

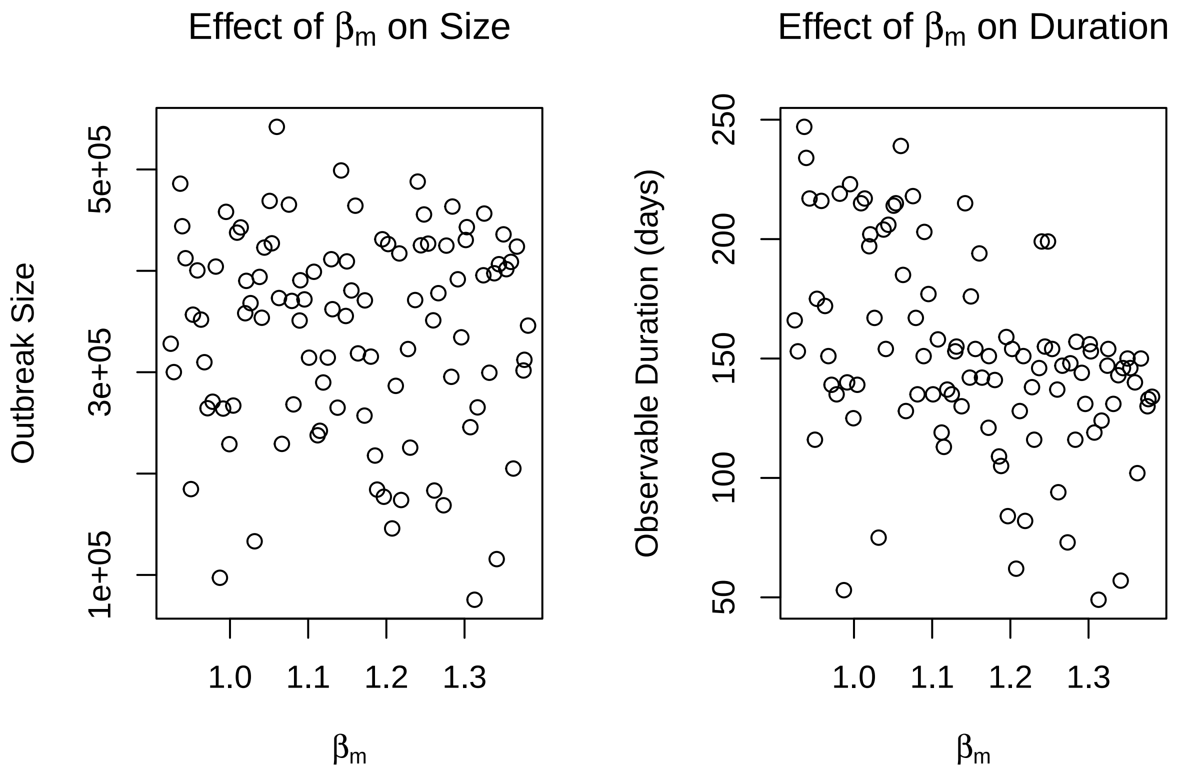


```
plot(mSIR$MaxInf ~ mSIR$gamma_m, main = expression(paste("Effect of ", gamma[m],
    " on Size")), xlab = expression(gamma[m]), ylab = "Outbreak Size")
plot(mSIR$Thresh100 ~ mSIR$gamma_m, main = expression(paste("Effect of ", gamma[m],
    " on Duration")), xlab = expression(gamma[m]), ylab = "Observable Duration (days)")
```

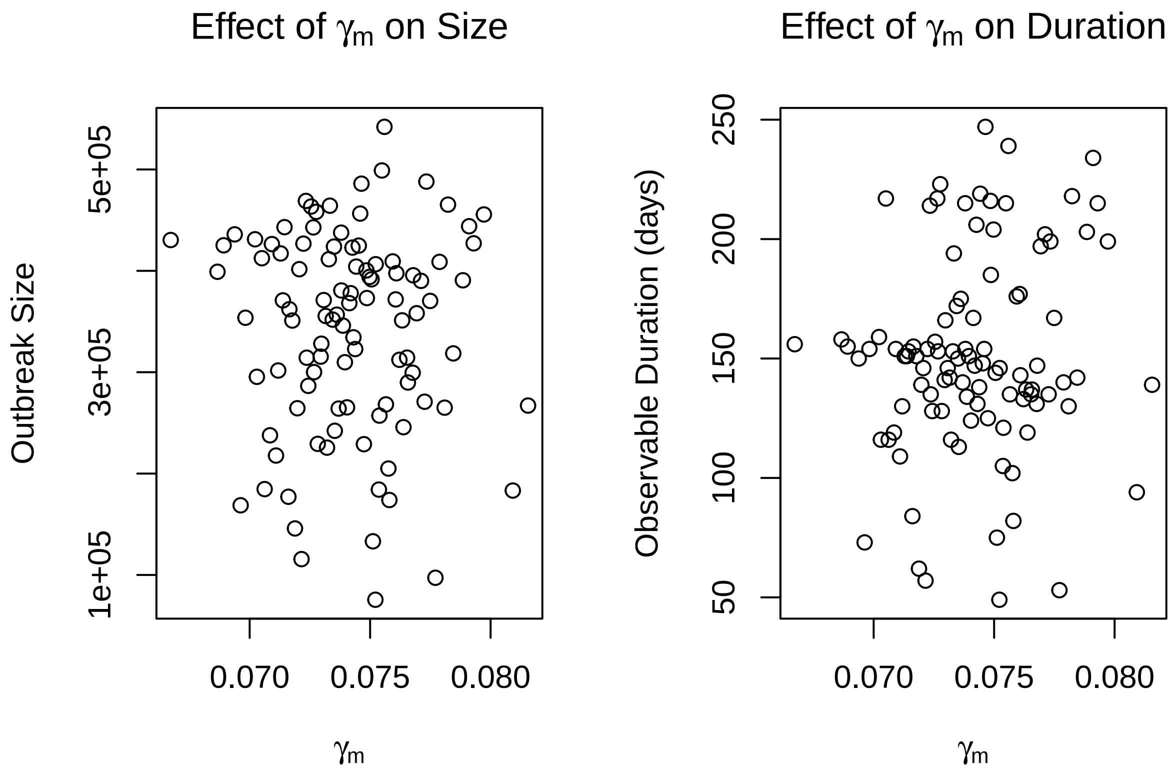


```
plot(mSIR$MaxInf ~ mSIR$g_m, main = expression(paste("Effect of ", g[m], " on Size")),
    xlab = expression(g[m]), ylab = "Outbreak Size")
plot(mSIR$Thresh100 ~ mSIR$g_m, main = expression(paste("Effect of ", g[m], " on Duration")),
    xlab = expression(g[m]), ylab = "Detectable Duration (days)")
```

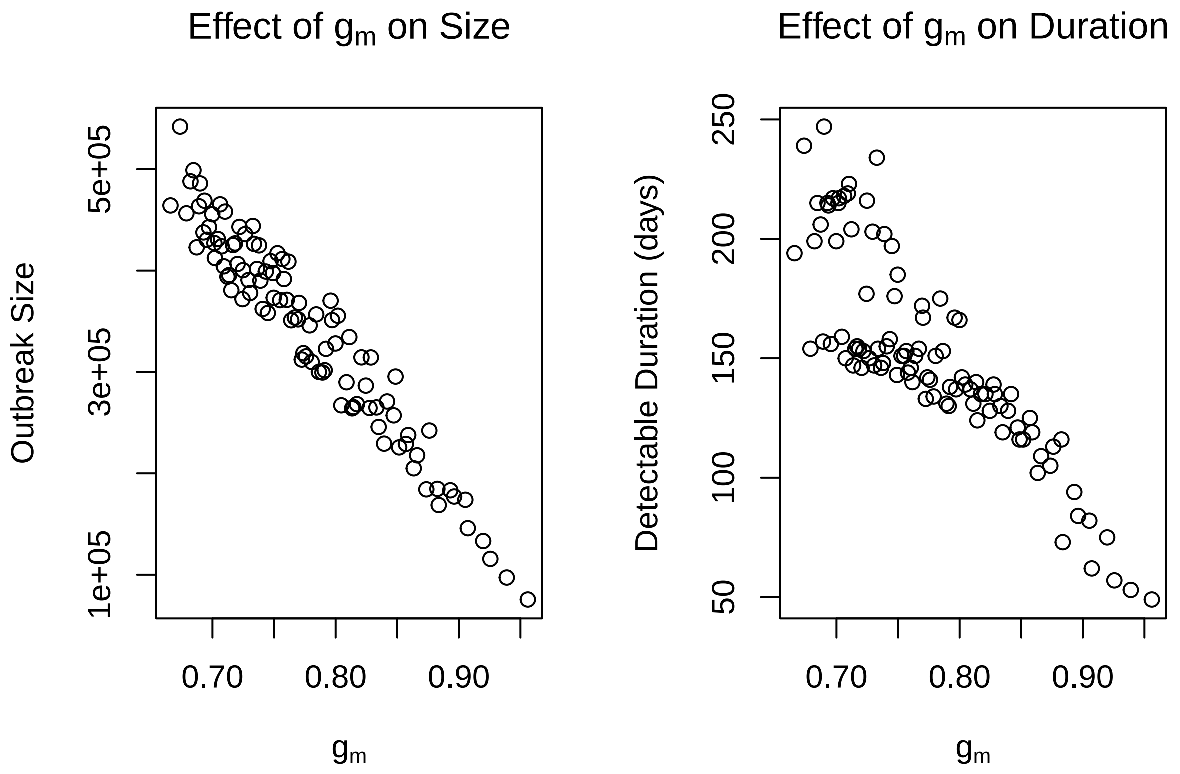


```
plot(mSIR$MaxInf ~ mSIR$b_h, main = expression(paste("Effect of ", b[h], " on Size")),
    xlab = expression(b[h]), ylab = "Outbreak Size")
plot(mSIR$Thresh100 ~ mSIR$b_h, main = expression(paste("Effect of ", b[h], " on Duration")),
    xlab = expression(b[h]), ylab = "Observable Duration (days)")
```

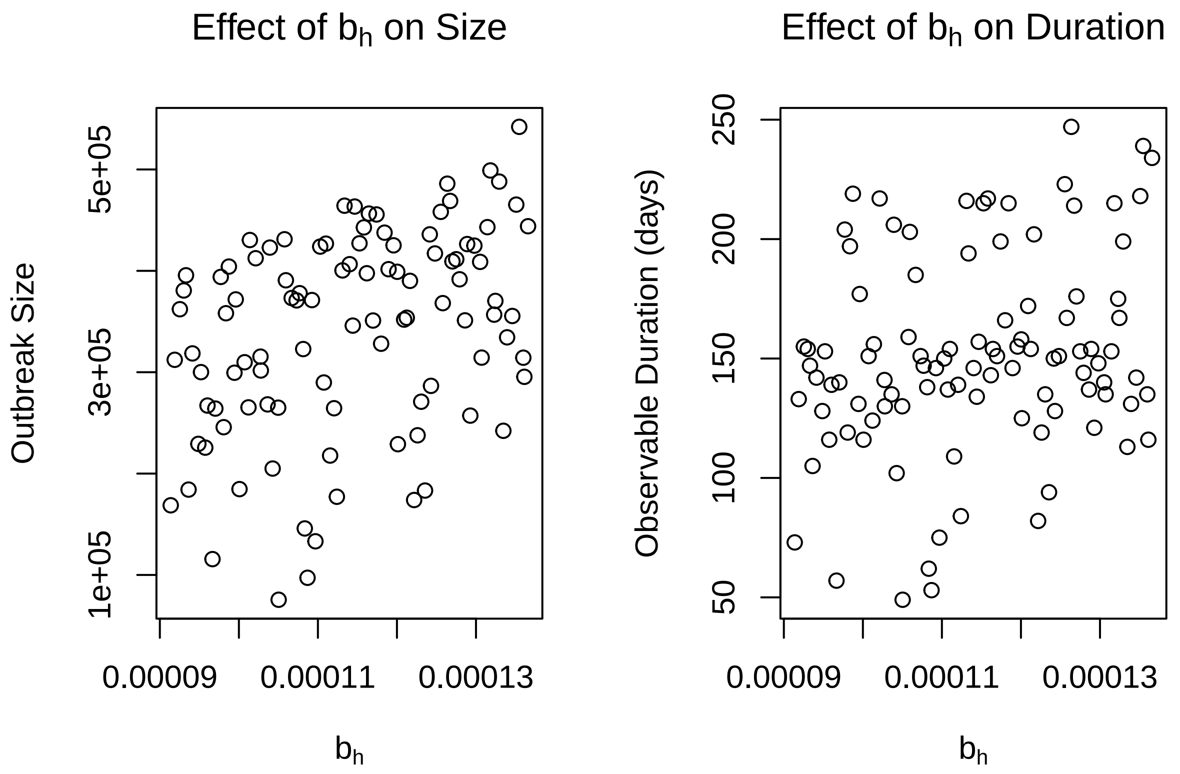


```
plot(mSIR$MaxInf ~ mSIR$d_h, main = expression(paste("Effect of ", d[h], " on Size")),
    xlab = expression(d[h]), ylab = "Outbreak Size")
plot(mSIR$Thresh100 ~ mSIR$d_h, main = expression(paste("Effect of ", d[h], " on Duration")),
    xlab = expression(d[h]), ylab = "Observable Duration (days)")
```

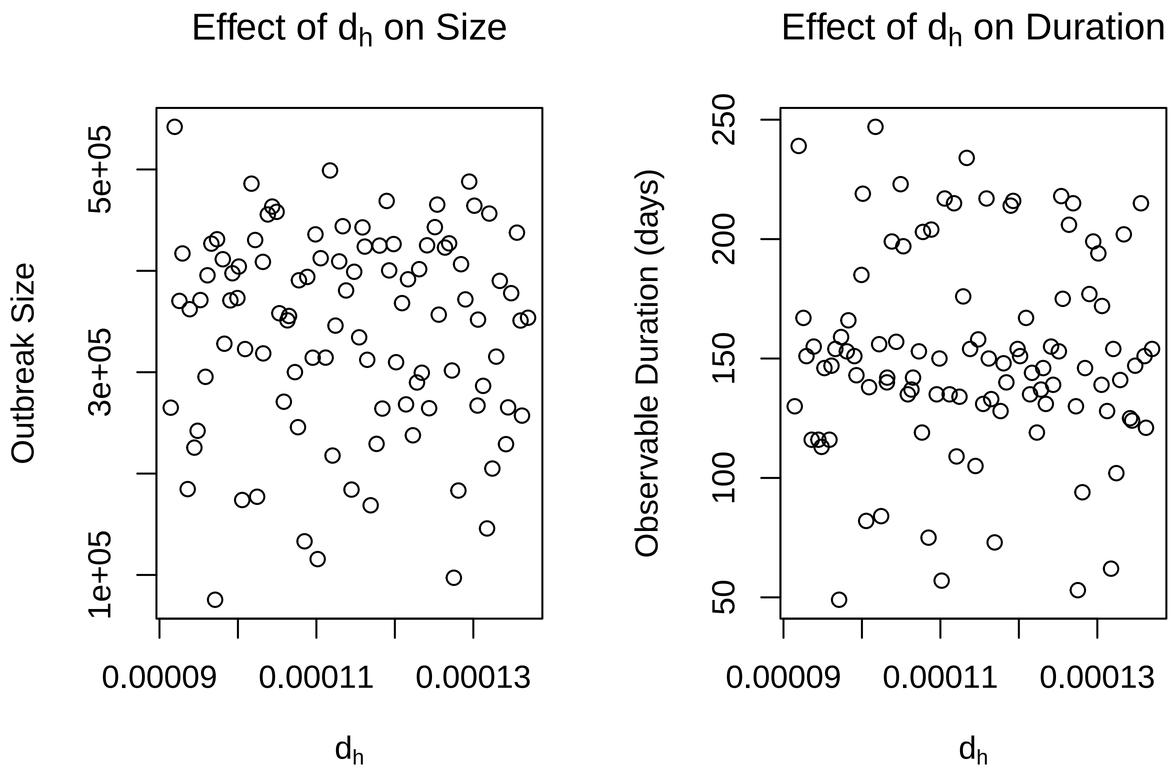


```r
par(mfrow = c(1, 2))
boxplot(mSIR$MaxInf, main = "Outbreak Size", ylab = "Number of Dead Humans", ylim = c(0,
    923406))
boxplot(mSIR$Thresh100, main = "Observable Outbreak Duration", ylab = "Time (Days)")
```

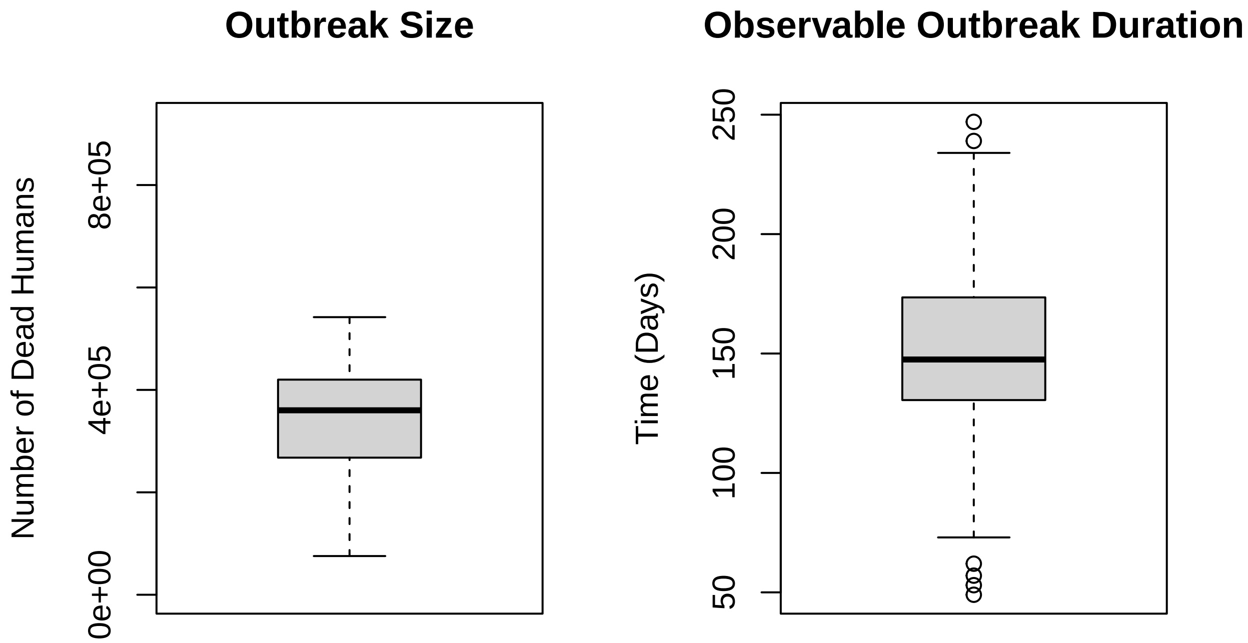


```
bonferroni.alpha <- 0.05/length(parameters)
prcc_size <- pcc(mSIR[, 1:length(parameters)], mSIR$MaxInf, nboot = niter, rank = TRUE,
    conf = 1 - bonferroni.alpha)
prcc_duration <- pcc(mSIR[, 1:length(parameters)], mSIR$Thresh100, nboot = niter,
    rank = TRUE, conf = 1 - bonferroni.alpha)

# plot correlation coefficients and confidence intervals for epidemic size and
# duration
size <- prcc_size$PRCC
size$param <- rownames(size)
colnames(size)[4:5] <- c("maxCI", "minCI")
size$maxCI[which(size$maxCI > 1)] <- 1
size$maxCI[which(size$maxCI < -1)] <- -1
size$minCI[which(size$minCI > 1)] <- 1
size$minCI[which(size$minCI < -1)] <- -1

duration <- prcc_duration$PRCC
duration$param <- rownames(duration)
colnames(duration)[4:5] <- c("maxCI", "minCI")
duration$maxCI[which(duration$maxCI > 1)] <- 1
duration$maxCI[which(duration$maxCI < -1)] <- -1
duration$minCI[which(duration$minCI > 1)] <- 1
duration$minCI[which(duration$minCI < -1)] <- -1

A <- ggplot(size, aes(x = param, y = original)) + geom_point(size = 4) + geom_errorbar(aes(ymax = maxCI
    ymin = minCI)) + ggtitle("A") + xlab("Parameters") + ylab("Partial Rank Correlation Coefficients") +
```

```
    scale_x_discrete(labels = c(beta_m = expression(beta[m]), b_h = expression(b[h]),
        d_h = expression(d[h]), gamma_m = expression(gamma[m]), g_m = expression(g[m]))) +
    ylim(-1, 1)

B <- ggplot(duration, aes(x = param, y = original)) + geom_point(size = 4) + geom_errorbar(aes(ymax = ma
    ymin = minCI)) + ggtitle("B") + xlab("Parameters") + ylab(" ") + scale_x_discrete(labels = c(beta_m
    b_h = expression(b[h]), d_h = expression(d[h]), gamma_m = expression(gamma[m]),
    g_m = expression(g[m]))) + ylim(-1, 1)
```

Measles SEIR - Figure S8 (Panels C & D)

```
parameters <- c(beta_m = 1.175, sigma_m = 1/10, gamma_m = 1/13, g_m = 0.7, b_h = 1/(25 *
    365), d_h = 1/(25 * 365))  #you can play with transmission and recovery rates here

# plot scatterplots
par(mfrow = c(1, 2))
plot(mSEIR$MaxInf ~ mSEIR$beta_m, main = expression(paste("Effect of ", beta[m],
    " on Size")), xlab = expression(beta[m]), ylab = "Outbreak Size")
plot(mSEIR$Thresh100 ~ mSEIR$beta_m, main = expression(paste("Effect of ", beta[m],
    " on Duration")), xlab = expression(beta[m]), ylab = "Observable Duration (days)")
```

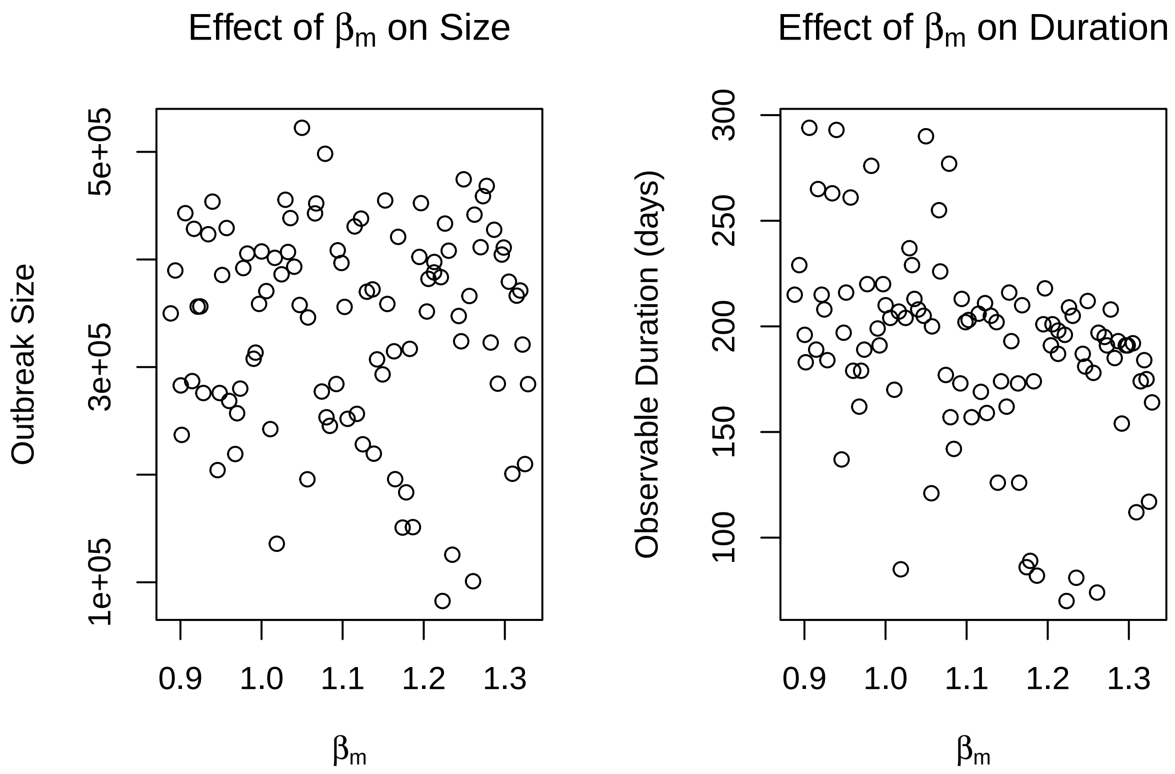

```
plot(mSEIR$MaxInf ~ mSEIR$sigma_m, main = expression(paste("Effect of ", sigma[m],
    " on Size")), xlab = expression(sigma[m]), ylab = "Outbreak Size")
plot(mSEIR$Thresh100 ~ mSEIR$sigma_m, main = expression(paste("Effect of ", sigma[m],
    " on Duration")), xlab = expression(sigma[m]), ylab = "Detectable Outbreak Duration (days)")
```

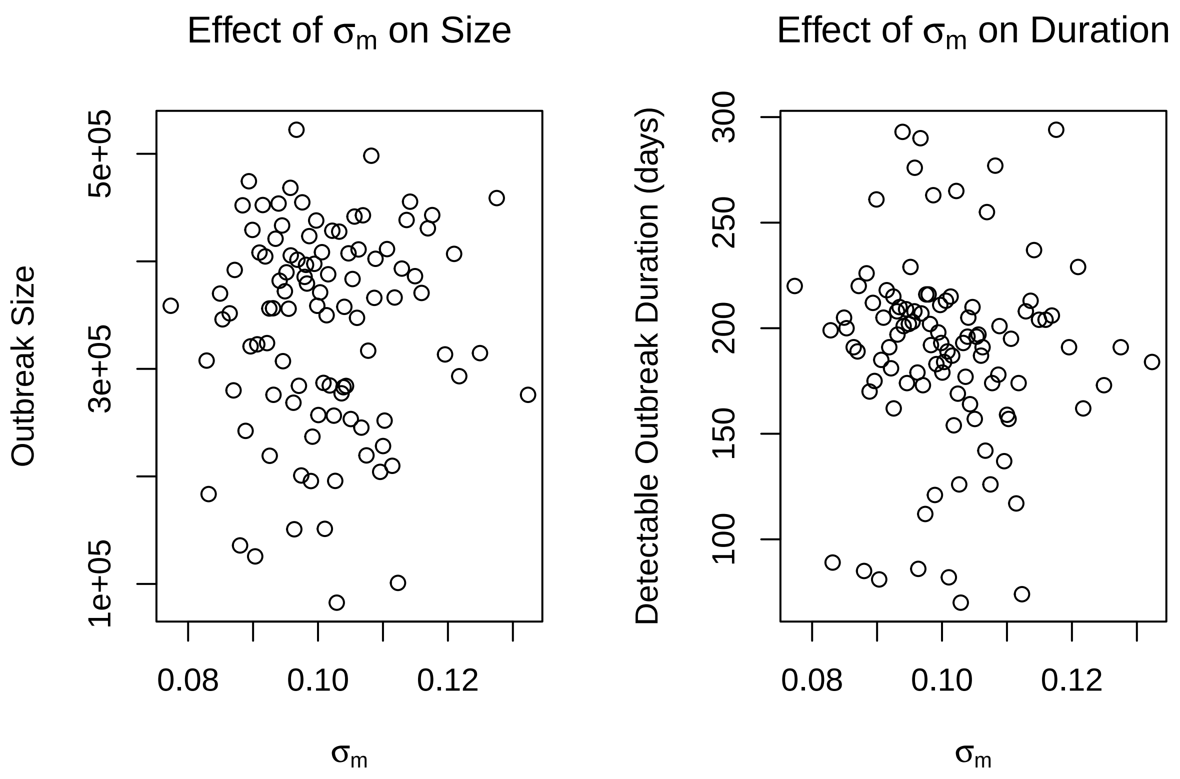


```
plot(mSEIR$MaxInf ~ mSEIR$gamma_m, main = expression(paste("Effect of ", gamma[m],
    " on Size")), xlab = expression(gamma[m]), ylab = "Outbreak Size")
plot(mSEIR$Thresh100 ~ mSEIR$gamma_m, main = expression(paste("Effect of ", gamma[m],
    " on Duration")), xlab = expression(gamma[m]), ylab = "Observable Duration (days)")
```

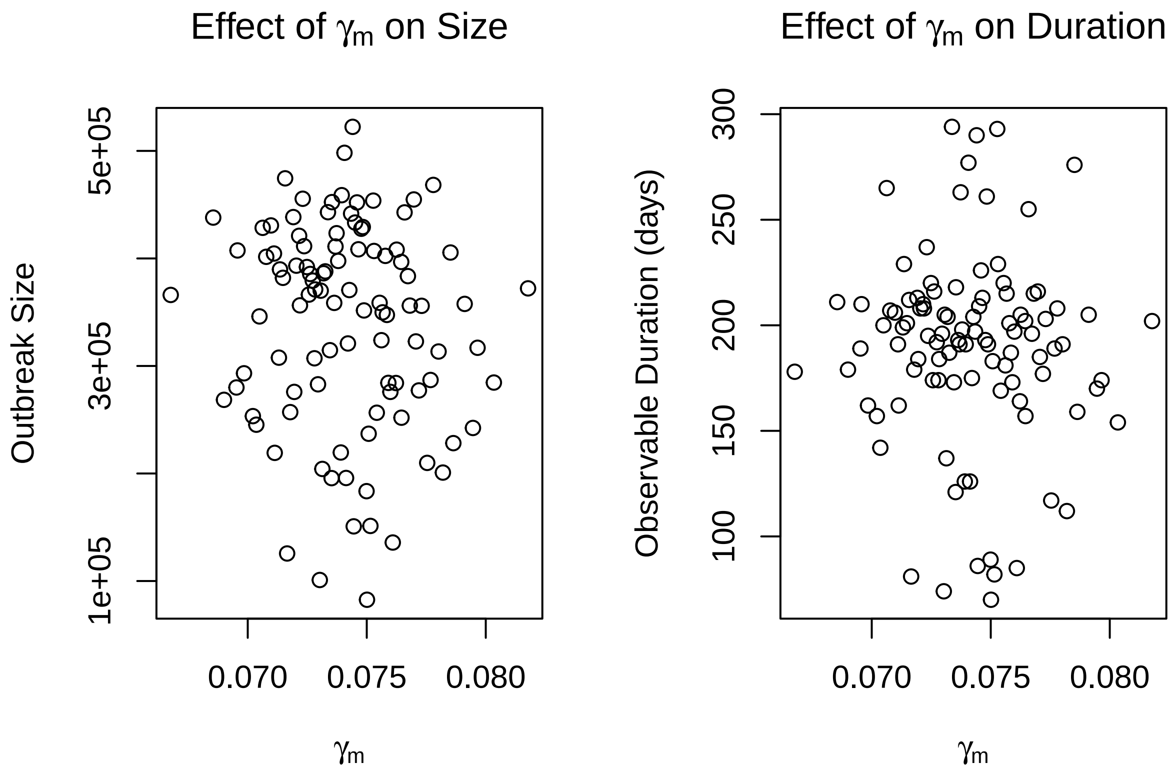


```
plot(mSEIR$MaxInf ~ mSEIR$g_m, main = expression(paste("Effect of ", g[m], " on Size")),
    xlab = expression(g[m]), ylab = "Outbreak Size")
plot(mSEIR$Thresh100 ~ mSEIR$g_m, main = expression(paste("Effect of ", g[m], " on Duration")),
    xlab = expression(g[m]), ylab = "Detectable Duration (days)")
```

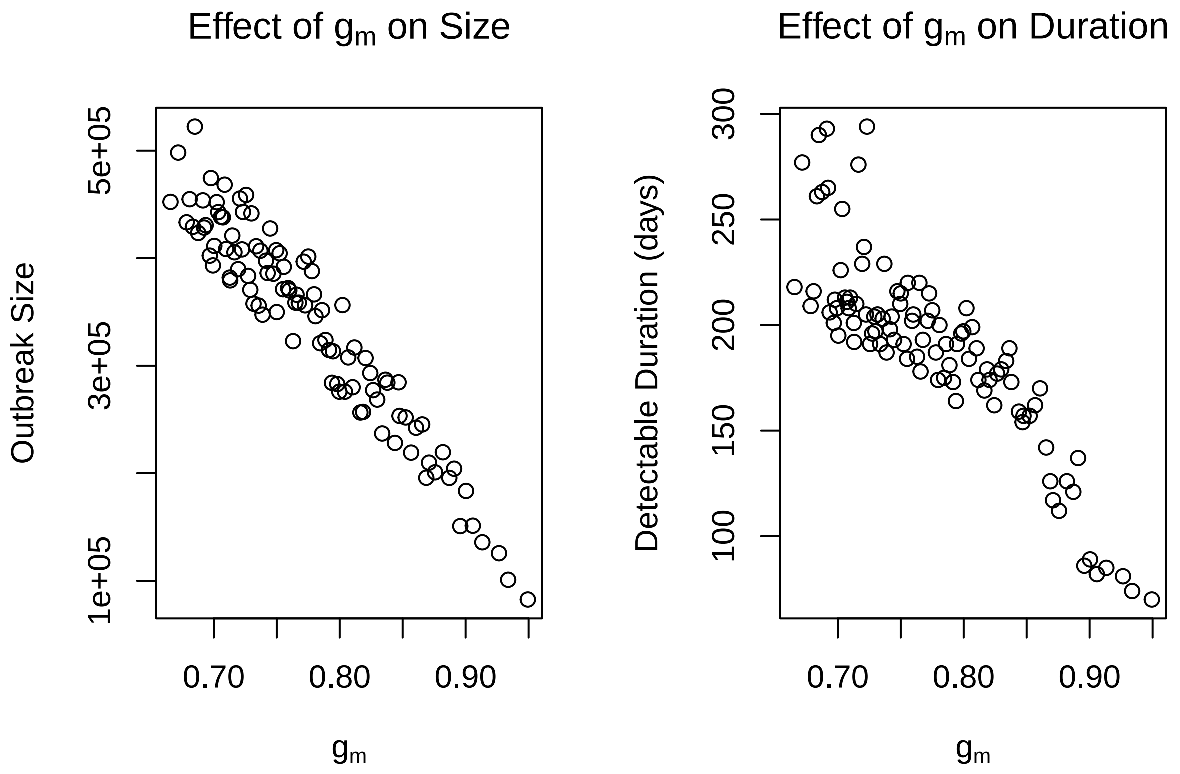


```
plot(mSEIR$MaxInf ~ mSEIR$b_h, main = expression(paste("Effect of ", b[h], " on Size")),
    xlab = expression(b[h]), ylab = "Outbreak Size")
plot(mSEIR$Thresh100 ~ mSEIR$b_h, main = expression(paste("Effect of ", b[h], " on Duration")),
    xlab = expression(b[h]), ylab = "Observable Duration (days)")
```

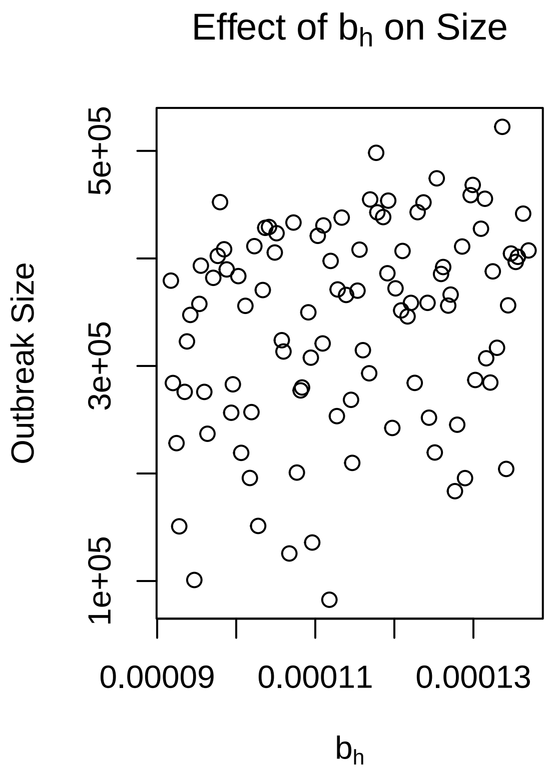

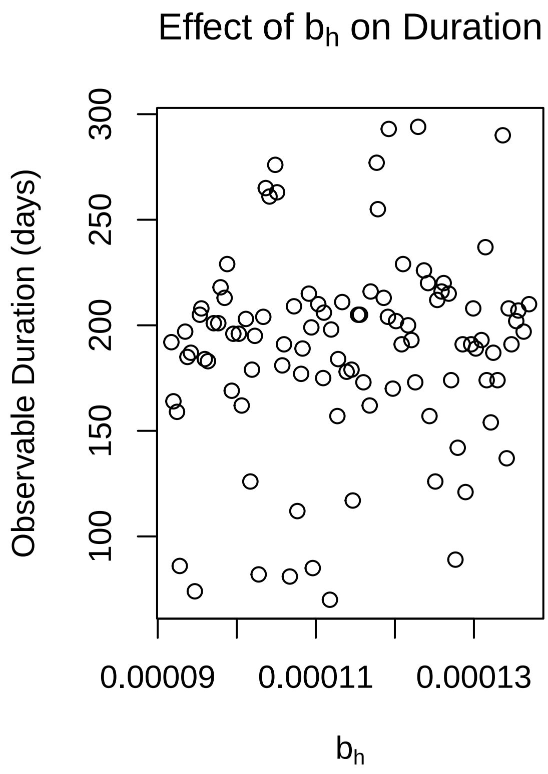


```
plot(mSEIR$MaxInf ~ mSEIR$d_h, main = expression(paste("Effect of ", d[h], " on Size")),
    xlab = expression(d[h]), ylab = "Outbreak Size")
plot(mSEIR$Thresh100 ~ mSEIR$d_h, main = expression(paste("Effect of ", d[h], " on Duration")),
    xlab = expression(d[h]), ylab = "Observable Duration (days)")
```

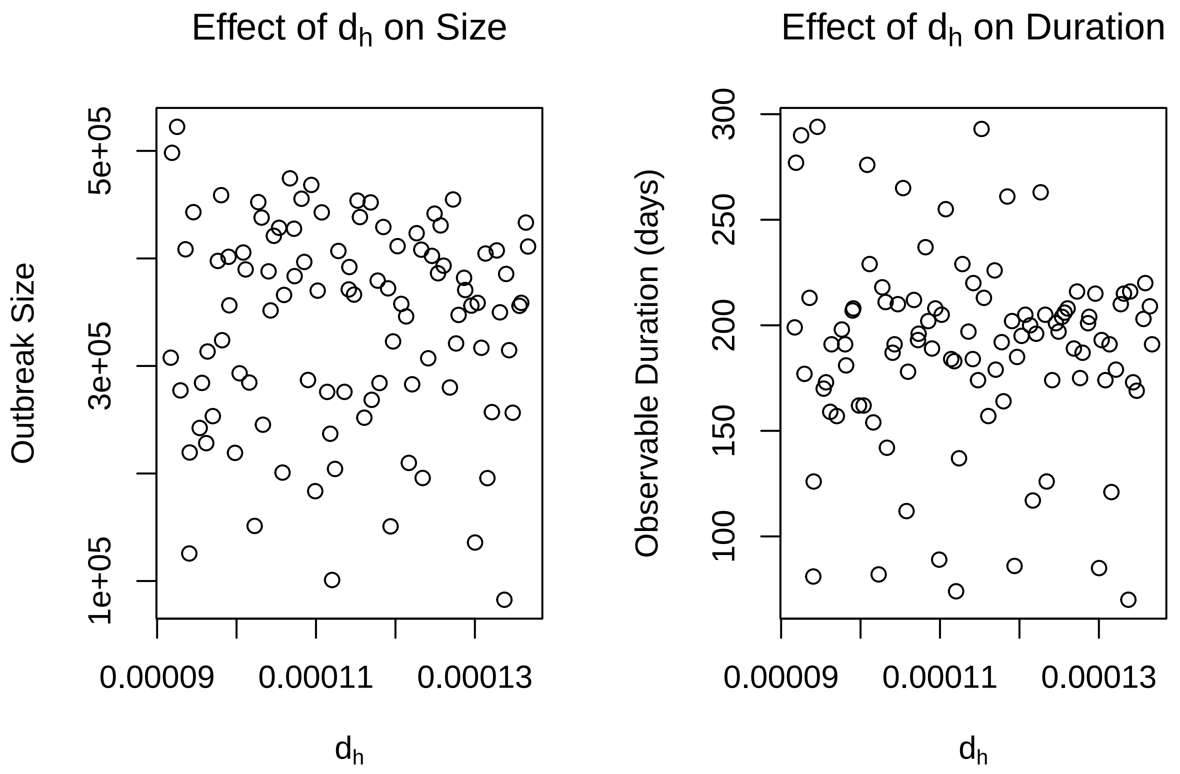


```
par(mfrow = c(1, 2))
boxplot(mSEIR$MaxInf, main = "Outbreak Size", ylab = "Number of Dead Humans", ylim = c(0,
    923406))
boxplot(mSEIR$Thresh100, main = "Outbreak Duration", ylab = "Time (Days)")
```

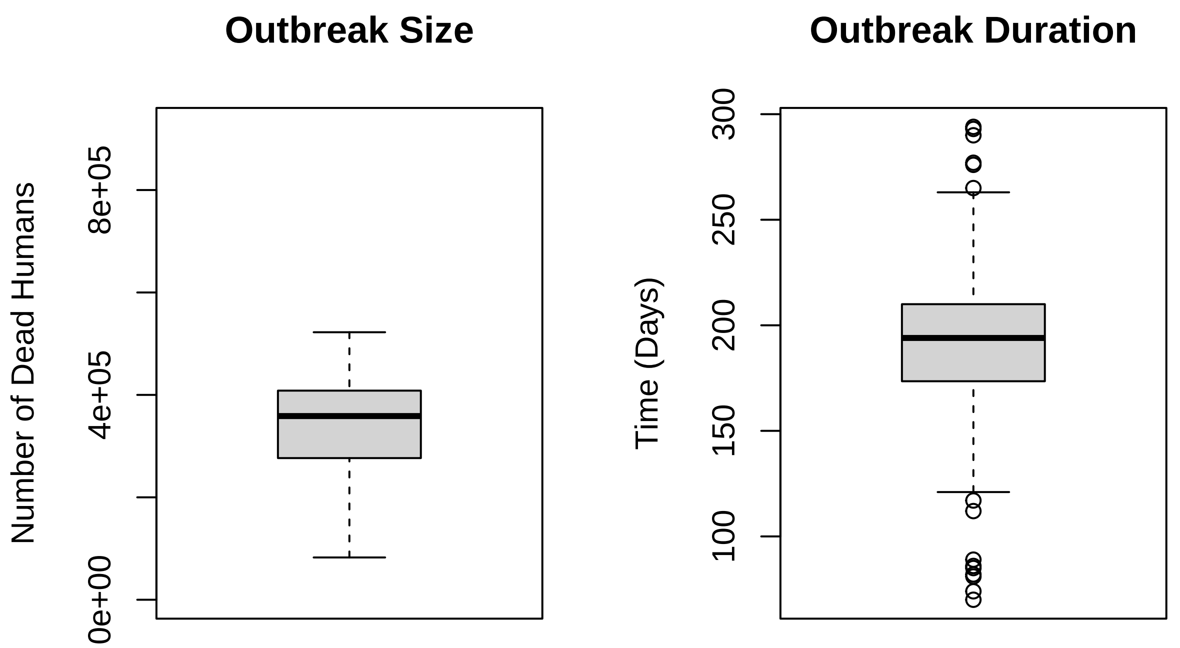


```
bonferroni.alpha <- 0.05/length(parameters)
prcc_size <- pcc(mSEIR[, 1:length(parameters)], mSEIR$MaxInf, nboot = niter, rank = TRUE,
    conf = 1 - bonferroni.alpha)
prcc_duration <- pcc(mSEIR[, 1:length(parameters)], mSEIR$Thresh100, nboot = niter,
    rank = TRUE, conf = 1 - bonferroni.alpha)

# plot correlation coefficients and confidence intervals for epidemic size and
# duration
size <- prcc_size$PRCC
size$param <- rownames(size)
colnames(size)[4:5] <- c("maxCI", "minCI")
size$maxCI[which(size$maxCI > 1)] <- 1
size$maxCI[which(size$maxCI < -1)] <- -1
size$minCI[which(size$minCI > 1)] <- 1
size$minCI[which(size$minCI < -1)] <- -1

duration <- prcc_duration$PRCC
duration$param <- rownames(duration)
colnames(duration)[4:5] <- c("maxCI", "minCI")
duration$maxCI[which(duration$maxCI > 1)] <- 1
duration$maxCI[which(duration$maxCI < -1)] <- -1
duration$minCI[which(duration$minCI > 1)] <- 1
duration$minCI[which(duration$minCI < -1)] <- -1

C <- ggplot(size, aes(x = param, y = original)) + geom_point(size = 4) + geom_errorbar(aes(ymax = maxCI
    ymin = minCI)) + ggtitle("C") + xlab("Parameters") + ylab("Partial Rank Correlation Coefficients")
```

```
    scale_x_discrete(labels = c(beta_m = expression(beta[m]), b_h = expression(b[h]),
        d_h = expression(d[h]), gamma_m = expression(gamma[m]), g_m = expression(g[m]),
        sigma_m = expression(sigma[m]))) + ylim(-1, 1)
D <- ggplot(duration, aes(x = param, y = original)) + geom_point(size = 4) + geom_errorbar(aes(ymax = ma
    ymin = minCI)) + ggtitle("D") + xlab("Parameters") + ylab(" ") + scale_x_discrete(labels = c(beta_m
    b_h = expression(b[h]), d_h = expression(d[h]), gamma_m = expression(gamma[m]),
    g_m = expression(g[m]), sigma_m = expression(sigma[m]))) + ylim(-1, 1)

tiff("FigS8.tiff", height = 22.23, width = 19.05, units = "cm", compression = "lzw",
    res = 600)
multiplot(A, B, C, D, cols = 2)
dev.off()
```

```
## pdf
##   2
```
