# Supplementary material for: Comparative SIR/SEIR modeling of the Antonine Plague in Rome
Source: PLoS One. 2025 Feb 13;20(2):e0313684. doi: 10.1371/journal.pone.0313684 (PMC11824979; doi:10.1371/journal.pone.0313684)
Supplement: Attachment — Submitted filename: PlosOne Review_PONE-D-24-04961.pdf [file pone.0313684.s011.pdf]

Dear Editor,

Dear PLOS ONE Editorial Office,

I would like to thank you for your kind invitation to review the manuscript ID#PONE-D-24-04961 and entitled “Comparative SIR/SEIR modeling of the Antonine Plague in Rome”.

Please find the review report below.

The article is fascinating and addresses developing a series of dynamic ordinary differential equation models of the spread of disease in Rome between 165 and 189 CE for several pathogens. Please consider the comments below to improve the paper further:

1) From my perspective, the abstract section is well handled and conveys clear content about the article to the target readership.
2) The authors are suggested to check the whole manuscript one more time to avoid typing and/or grammatical errors. For example, in Abstract is *“....ancient authors, or (b) the daily deaths in ca.*” correct? What is “ca” here? Is it "circa"?
3) In Introduction, page 2, lines 28-29; is there any problem with the typing of “…in the [r]eligious and cultural history of the 29 [Roman E]mpire as well as [in] its social and political development” (1).”?
4) Abbreviations should be stated with their expansions where they are first mentioned. Just a few examples: in Introduction, page 4, line 90 *(“…we adapted the following ODE (?)…*), line 93 *(“… period, i.e., the SIR (?) and SEIR (?) variants); and (2) bubonic…”)*
5) The omission of the main purpose of the study in the final paragraph of the Introduction is regarded as a significant deficiency
6) The literature review in the Introduction section generally focuses on Antonine Plague studies and as expected relies on relatively outdated references. However, the absence of recent modeling studies based on SIR/SEIR models indicates a deficiency in the literature review. It is strongly recommended to include recent studies on prediction models developed based on SIR/SEIR models in the literature review section to ensure a more comprehensive and up-to-date review. Several relevant studies are listed below.

Tutsoy, O., Çolak, Ş., Polat, A., & Balikci, K. (2020). A novel parametric model for the prediction and analysis of the COVID-19 casualties. *Ieee Access*, *8*, 193898-193906.

Tutsoy, O., Polat, A., Çolak, Ş., & Balikci, K. (2020). Development of a multi-dimensional parametric model with non-pharmacological policies for predicting the COVID-19 pandemic casualties. *Ieee Access*, *8,* 225272-225283.

Tutsoy, O., & Polat, A. (2022). Linear and non-linear dynamics of the epidemics: System identification based parametric prediction models for the pandemic outbreaks. *ISA transactions*, *124,* 90-102.

Maier, B. F., & Brockmann, D. (2020). Effective containment explains subexponential growth in recent confirmed COVID-19 cases in China. *Science*, *368*(6492), 742-746.

Abou-Ismail, A. (2020). Compartmental models of the COVID-19 pandemic for physicians and physician-scientists. *SN comprehensive clinical medicine*, *2*(7), 852-858.

Arenas, A. J., Gonzalez-Parra, G., & Chen-Charpentier, B. M. (2016). Construction of nonstandard finite difference schemes for the SI and SIR epidemic models of fractional order. *Mathematics and Computers in Simulation*, *121*, 48-63.

Chen YC, Lu PE, Chang CS, Liu TH. A time-dependent SIR model for COVID-19 with undetectable infected persons. *IEEE Trans Netw Sci Eng* 2020;7(4):3279–94.

7) What do $\boldsymbol{F}$s stand for in the equations in the lines 141-142?
8) Please check the grammatical mistake in the sentence in the lines 150-152
9) Significant issues with the description of variables in the equations have been noted, such as with r_f, K_f, and d_f in lines 153-154. A thorough review and revision of all equations are necessary to ensure that each variable is clearly and accurately described. While these specific instances have been highlighted, a comprehensive review is recommended to address any other potential omissions or ambiguities.
10) There is a lack of analysis and commentary on how the equations in the bubonic SEIR model, provided in lines 180-185, were developed. It is unclear which effects contributed positively and which contributed negatively to the model. A detailed explanation, including justifications, is necessary. Additionally, similar to comment (8),

many variables are presented without proper definition. Just one more example: What is σ_b? Again, a thorough review and clarification of all variables are required. Although the explanations of these variables and the analyses of the equations are partially provided in subsequent sections, they should be presented initially, at the point where the equations are first introduced, to maintain a logical flow and improve readability.

11) The equations given in lines 198-205 should also be considered as in comment (10).
12) The equations until Table-1 should also be considered as in comment (10).
13) In Table 1, Expected value [Range: min, max] is “3/Sr (t = 0) [0.39 < αKr < 20] rats-1”….etc. In these values, which one is Expected value? Which one is Range and which ones are min, max? In my opinion, this column of the table is presented in an incomprehensible manner. It is overly complicated, making it difficult, if not impossible, for readers to understand.
14) The discussion is generally coherent, and the conclusion effectively highlights the main findings, such as the models' failure to replicate the mortality pattern from primary sources and the possibility that secondary literature may have overstated the impact of the Antonine Plague. To further strengthen the conclusion, it is recommended to add a sentence or paragraph summarizing the overall conclusion of the study. This should encapsulate the key takeaways and the broader implications of the findings for historical interpretations of the plague and for future research directions.
15) In S2Fig., the red line representing the Bubonic & Pneumonic SEIR model does not appear. Although it seems to follow either the blue or green curve (which needs clarification), it would be beneficial to adjust the figure so that the red line is visible. This will ensure clarity and accuracy in the representation of the data.

I wish you success in implementing the suggested improvements.
