# Supplementary material for: Comparative SIR/SEIR modeling of the Antonine Plague in Rome
Source: PLoS One. 2025 Feb 13;20(2):e0313684. doi: 10.1371/journal.pone.0313684 (PMC11824979; doi:10.1371/journal.pone.0313684)
Supplement: Attachment — Submitted filename: ap_epidemiology_2021_response_to_reviewers.docx [file pone.0313684.s012.docx]

Dear Editor-in-Chief, dear Reviewers,

We sincerely appreciate the thorough review of our manuscript. We have addressed each comment to the best of our ability, providing additional details where necessary. Below, we provide a point-by-point response to the feedback received:

- Response to editor’s comment 1: We are glad to hear (read) that.
- Response to editor’s comment 2: Reviewed. “Ca.” indeed means circa.
- Response to editor’s comment 3: This is typographically correct (the square brackets indicate our clarifying edits to the cited text).
- Response to editor’s comment 4: Indeed, fixed.
- Response to editor’s comment 5: Purpose statement added.
- Response to editor’s comment 6: Done.
- Response to editor’s comment 7: Added explanation of *F* to lines 146-147 of the reviewed submission. The *F*s are explained below on line 151 of the original submission (line 159 of the reviewed submission), in place where the behavior of the variables *F* and *H* is discussed. Since each subpopulation variable is discussed before the equation describing it is introduced, we consider the current approach of explanation more consistent and appropriate.
- Response to editor’s comment 8: There was indeed a mistake, fixed.
- Response to editor’s comment 9: Fixed.
- Response to editor’s comment 10, 11, and 12: Fixed. The analysis of which parameters and to what extent affected the progress of the modeled outbreaks is provided in the *Results* section (specifically the *Sensitivity analysis: Model outcome variability and parameter influence* part) because it is based on the results of the models.
- Response to editor’s comment 13: Fixed by adding explanatory notes to unclear rows. All the cells in the “Expected value [Range: min, max]” column follow the same structure. I.e., contain the most likely value of the given variable based on literature followed by the potential minimal and maximal values the variable can range over.
- Response to editor’s comment 14: Conclusion enhanced.
- Response to editor’s comment 15: Fixed.
- Response to comment by Reviewer #1: Addressed at the end of the *Discussion* section.
- Response to Journal Requirement 1: Done.
- Response to Journal Requirement 2: The funders had no role in study design, data collection and analysis, decision to publish, or preparation of the manuscript.
- Response to Journal Requirement 3: Fixed.
- Response to Journal Requirement 4: Fixed.

We greatly appreciate the detailed review of our manuscript and hope that we have met the expectations of both the reviewers and the editor.

Sincerely,

Anestis Karasaridis^1,2^ (Ph.D. Student), Aleš Chalupa^1,2^ (Assistant Professor)

^1^ Department for the Study of Religions, Faculty of Arts, Masaryk University, Brno, Czech Republic

^2^ Centre for the Digital Research of Religion (CEDRR), Masaryk University, Brno, Czech Republic
